# A benchmark study of aromaticity indexes for benzene, pyridine and the diazines – I. Ground state aromaticity†

**DOI:** 10.1039/d2ra00093h

**Published:** 2022-01-19

**Authors:** Jacob Pedersen, Kurt V. Mikkelsen

**Affiliations:** Department of Chemistry, University of Copenhagen Copenhagen DK-2100 Denmark kmi@chem.ku.dk

## Abstract

Five different aromaticity indexes are benchmarked for benzene, pyridine and the diazines in their ground states. A basis set study was performed using the Pople style, Karlsruhe and Dunning's correlation consistent basis sets. Ten different DFT functionals, including LSDA, PBE, PBE0, B3LYP, CAM-B3LYP, wB97XD, M06-2X, SOGGA11X, M11 and MN15 were benchmarked by comparison with CCSD, CASSCF and MP2. Large out-of-plane imaginary frequencies were observed for some of the optimized structures at the correlated wavefunction level of theory. It was found that the DFT functionals in general predict the *para*-delocalization index, multicenter index and aromatic fluctuation index to be approximately 70%, 50% and 45% larger, respectively, compared to the CCSD method. Comparisons of the DFT functionals showed that the wB97XD, CAM-B3LYP and M06-2X functionals performed the best. Furthermore, the basis set dependence of the DFT functionals was found to be large for the electron sharing indexes. Based on these findings, it is recommended to perform ground state calculations of aromaticity indexes at the wB97XD, CAM-B3LYP or M06-2X level of theory utilizing a simple basis set of triple-*ζ* quality.

## Abstract 2

Five different aromaticity indexes are benchmarked for benzene, pyridine and the diazines in their ground states.

## Graphical abstract

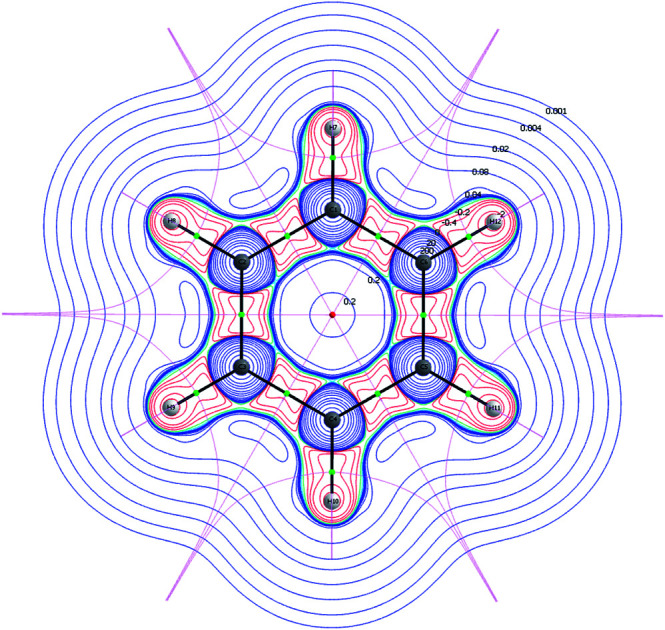

## 1 Introduction

The concept of aromaticity is cumbersome, because no general accepted definition of it exists. However, the concept is widely used and provides various application options in chemical engineering.^1,2^ The latest one being in MOlecular Solar Thermal (MOST) energy storage systems,^3–5^ in which aromaticity reversal between different electronic states potentially can extend the storage lifetime and enhance the switching properties of the organic photoswitches.^6,7^

Aromaticity is usually associated with a set of rather characteristic physicochemical properties, including cyclic electron delocalization, energetic stabilization, bond length equalization, exalted magnetic susceptibility, *etc.*^8^ As a consequence, aromaticity cannot be uniquely quantified nor measured directly. Instead, various indexes are designed to scale aromaticity by means of some of the characteristic physicochemical properties, and these are used as a measure for the amount of aromaticity in a given molecule. Hence, in order to get a reliable measure of the aromaticity in a molecule, multiple aromaticity indexes are required, since the properties are not necessarily are present simultaneously or related to each other.^9–11^

Molecular electronic structure calculations were performed in this study in order to calculate the aromaticity indexes (further description in the computational approach section). The accuracy of the aromaticity indexes is then directly related to the accuracy of the electronic structure theory used in the calculations. Highly correlated wavefunction methods are believed to yield very accurate results in general, but at a high cost.^12^ The molecules examined in this study is rather small, however, these molecules represent the set of the most common unit-structures, that constitutes many polycyclic arenes and heteroarenes. Thus, the behavior of the aromaticity indexes is believed to be encapsulated by this set of molecules.

Kohn–Sham Density Functional Theory (KS–DFT) is a different approach, in which the KS Fock operator is approximated. The challenge with KS–DFT is, that the exchange-correlation (XC) potential is unknown and cannot be approximated systematically, meaning that no lower or upper bound for the system exists.^13^ The variational principle provides a lower bound for the wavefunction methods, which can be approached systematically by including more Slater determinants, thereby recovering more electron correlation.^14^ Consequently, the DFT functionals must be benchmarked by comparison with either experimental data or results obtained from highly correlated wavefunction methods, in order to check the accuracy of the calculated properties. However, the DFT functionals may perform differently for different properties, thereby requiring a separate benchmark study for each property. This benchmark study is for the aromaticity indexes; HOMA,^15,16^ PDI,^17^ MCI,^18^ AV1245 ^19^ and FLU^19^ (see Section 2).†

### 1.1 Quantum theory of atoms in molecules

If the molecular volume is divided into vector subspaces, each containing one atom of the molecule, one can define the molecular properties in terms of atomic contributions. In Quantum Theory of Atoms In Molecules (QTAIM), the molecular wavefunction is partitioned in either Hilbert space or in real space, hence allowing for a topological analysis of the electron density in each vector subspace. The wavefunction partition is preferable performed in real space, since the choice of basis set dictates the accuracy of the partition in Hilbert space.^20,21^

The electron density is uniquely defined and exists in all space, guaranteed by the principles of quantum mechanics.^22,23^ The uniqueness of the wavefunction partition is then ensured by taking the electron density as central variable. The one electron density *γ*^(1)^ is defined in terms of the Reduced Density Matrix (RDM) method.^19^ For convenience, the electron pair density *γ*^(2)^ is similarly stated here:1
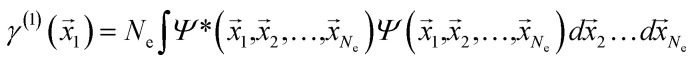
2
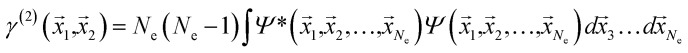
*N*_e_ is the number of electrons, and *x⃑* denotes the combined spatial and spin coordinate of the electron. Information about the dynamics of the electron density is then obtained by examination of the vector fields in each vector subspace. Each subspace satisfy the ‘quantum condition’, which states that the subspace must be bounded by a surface *S*, defined such that the inner product of the electron density gradient ∇*γ*^(1)^ and any vector *n*(*x⃑*), perpendicular to the surface *S*, is zero at all points on the surface:

3 ∇*γ*^(1)^(*x⃑*) · *n*(*x⃑*) = 0, ∀*x⃑* ∈ *S*(*x⃑*)

Any such surfaces are in the framework of QTAIM called zero-flux surfaces.^21,24^ Evaluation of the electron density gradient at different initial points in the vector fields allows one to trace out gradient paths and construct contour maps or phase portraits, depending on which space is used for the partitioning. Assuming real space, the critical points (*ω*, *σ*) are characterized by the rank of the Hessian (*ω*) and the signature of its eigenvalues (*σ* = *λ*_1_ + *λ*_2_ + *λ*_3_).^24^

**Fig. 1:**
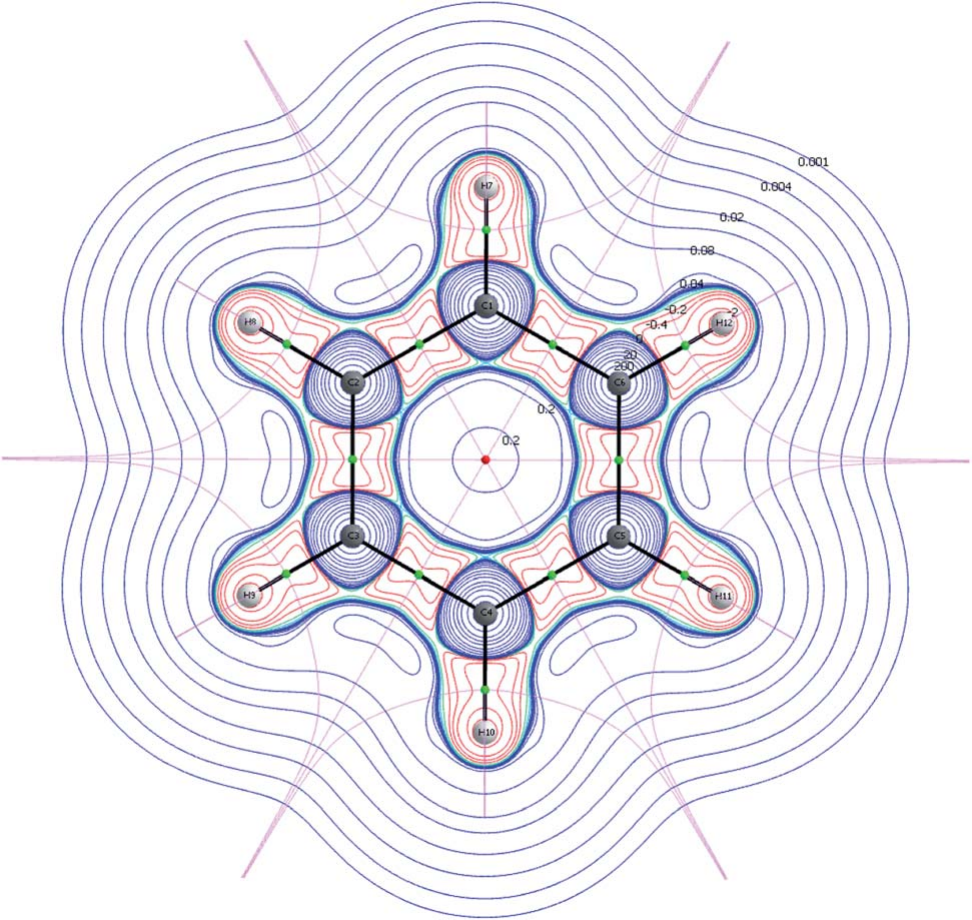
2D visualization of the wavefunction partition of benzene calculated at MN15/6-31+G(d). The RCP (red dot), BCPs (green dots), NCPs (medium spheres) and molecular graph (black solid lines) are illustrated. The contour map is colored by the value of the Hessian, and the pink lines are the zero-flux surfaces.

A Nuclear Critical Point (NCP) is defined as a local maximum (3,−3) of the electron density and indicates presence of a nucleus. Bond Critical Points (BCP)s are saddle points (3,−1), and they are located between two NCPs. It is noted, that the unstable manifolds (defined as the set of initial points; *x⃑*(*t*) → x⃑* as − *t* → *∞*, with *t* being the time) connect two NCPs, whereas the stable manifolds separate the basins assigned to each of the NCPs. A basin is defined as the region, in which all of the initial points move towards the attracting NCP. Hence, unstable manifolds indicate movement of electrons between two basins, *i.e.* electron sharing, and they are in the framework of QTAIM called atomic interaction lines or bond paths. A molecular graph is a collection of multiple bond paths. The stable manifolds satisfy the quantum condition and are the zero-flux surfaces. A Ring Critical Point (RCP) is defined as (3,1) and is found in the interior of ring structures (Fig. 1).^21,24^

## 2 Aromaticity indexes

The Harmonic Oscillator Model of Aromaticity (HOMA), defined by J. Kruszewski and T. M. Krygowski, is a geometrical aromaticity index.^15,16^4
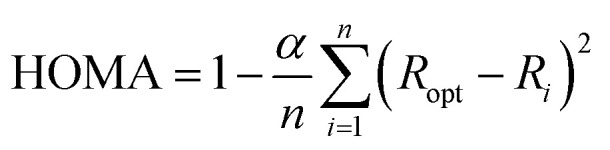


The empirical constant *α* is chosen, such that the HOMA index is equal to one for an aromatic molecule with equalized bond lengths and zero for the molecule in its Kekulé structure, *i.e.* with alternating bond lengths. The number of bonds with π-electrons is denoted *n*, and *R*_*i*_ is the bond length of the *i*-th bond. The bond-specific optimal bond length *R*_opt_ is calculated by minimization of the energy caused by forced steric strain in the molecule, also called deformation energy.^16^

Aromaticity indexes based on π-electron delocalization are commonly called Electron Sharing Indexes (ESI)s, and they are defined in terms of quantities from the QTAIM formalism.^19^ The difference between two independent one electron densities and the corresponding electron pair density is the exchange-correlation density (XCD) *γ*_*xc*_(*x⃑*_1_,*x⃑*_2_).

5 *γ*_*xc*_(*x⃑*_1_, *x⃑*_2_) = *γ*^(1)^(*x⃑*_1_)*γ*^(1)^(*x⃑*_2_) − *γ*^(2)^(*x⃑*_1_, *x⃑*_2_)

Integration of the XCD with respect to two different basins of attraction gives the delocalization index (DI):^19^6
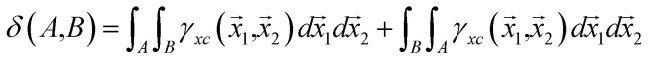


The atoms in a ring are ordered by the string 
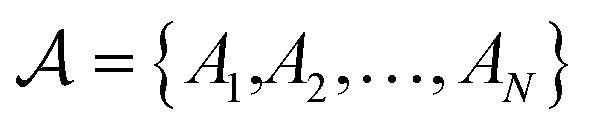
, where *N* is the number of atoms and by definition; *A*_1_ = *A*_*N*+1_. The *Para*–Delocalization Index (PDI), defined for six-membered rings only by J. Poater *et al.*, is the average value of the DI for all *para*-related atoms.^17^7
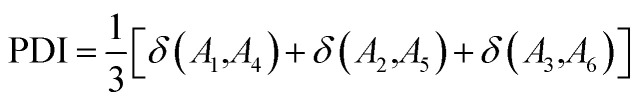


P. Butlinck *et al.* defined the Multicenter Index^18^ (MCI) in terms of the *I*_ring_ index, which was developed by M. Giambiagi *et al.*^25^ six years earlier.8
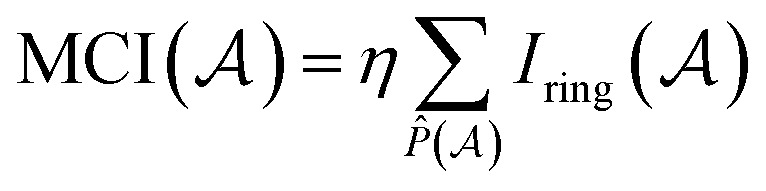
9
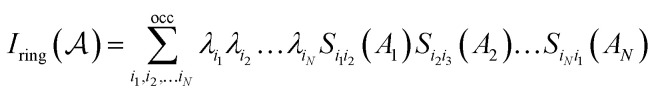


The normalization constant has been identified as *η* = 1/(2*N*).^19^ The permutation operator 
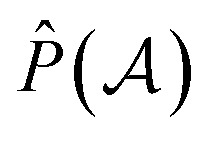
 works on the string 
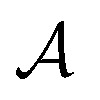
, thereby producing *N*! permutations. The atomic overlap matrix is the overlap between the two natural orbitals *ϕ*^NO^_*i*_ and *ϕ*^NO^_*j*_ on atom A.10
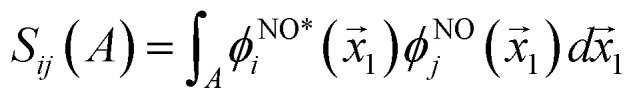


The natural orbitals and occupation numbers are the eigenvectors and the corresponding eigenvalues of the diagonal electron density matrix:11
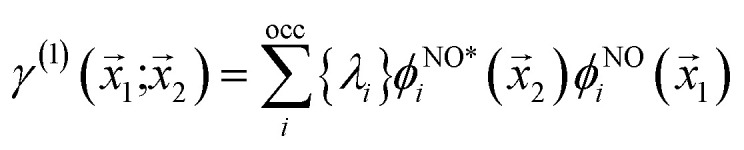


Delocalization of π-electrons between *para*-related carbon atoms has been observed to be larger than between the corresponding *meta*-related.^17^ Based on these findings, E. Matito *et al.* introduced the AV1245 index, which measures the delocalization between atoms of the positional relationship 1, 2, 4 and 5. The AV1245 index is defined as the mean of the MCI values of these atoms.^19^12
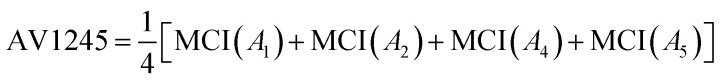


The aromatic fluctuation (FLU) index, also defined by E. Matito *et al.*,^26^ quantifies the delocalization–fluctuation over all adjacent atoms.13
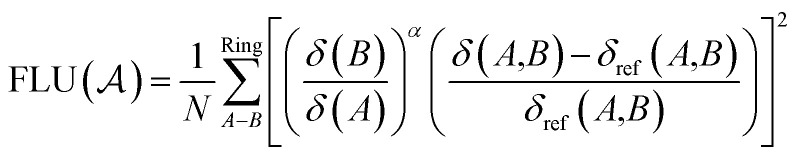
14
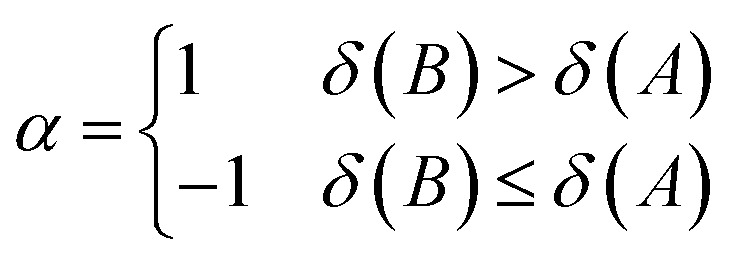


The atomic valence *δ*(*A*) can be shown to equal the difference between the electron population in a basin and half of the XCD integrated over the same basin twice. *δ*_ref_(*A*,*B*) is the reference DI of atom *A* and *B* from a chosen reference molecule.^19^

## 3 Computational approach

**Fig. 2:**
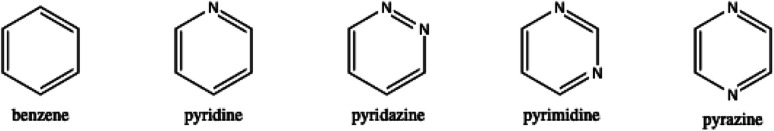
Kekulé structures of the molecules considered in this study.

The aromaticity indexes were calculated for the molecules (Fig. 2) in their ground states according to the procedure in Scheme S1 (located in the ESI).† The molecules were geometrical optimized in vacuum prior to the wavefunction calculations, and the frequencies were calculated to validate, that the optimized structures were local minima. Each step in the wavefunction preparation has been calculated at the same level of theory. All wavefunction calculations were performed in the Gaussian 16 (Rev A.03) program.^27^ (See ESI† for route section and *z*-matrices).

Single (or multiple) large out-of-plane imaginary frequencies were observed in some of the correlated wavefunction optimization and frequency calculations performed in Gaussian 16. Previous studies have shown similar results; optimized planar arenes turned out to be transitions states.^28,29^ In this study, the predicted transition structures were re-optimized in the Dalton (2016.1) program.^30^ The frequencies were similarly re-calculated in Dalton (when possible, or else in Gaussian 16), and for one of the calculations (pyridine at CASSCF(8,8)/6-31+G(d)), the frequencies confirmed, that the re-optimized structure now was a local minimum. The wavefunction for the re-optimized structure was then re-calculated in Gaussian 16. In a previous study, D. Asturiol *et al.* were able to turn all encountered transition structures into local minima by introducing a counterpoise correction.^29^ However, since only a few of the optimized structures were transition structures, no further corrections were performed. Further discussion of the imaginary frequencies and its consequences is given in the results and discussion section.

### 3.1 Electronic structure calculation

Second-order Møller-Plesset perturbation theory^31–36^ (MP2), Complete Active Space Self-Consistent Field^37–39^ (CASSCF) and Coupled Cluster including singles and doubles^40–43^ (CCSD) have been used to benchmark the ten DFT functionals; LSDA,^44^ PBE,^45^ PBE0,^46^ B3LYP,^47^ CAM-B3LYP,^48^ wB97XD,^49^ M06-2X,^50^ SOGGA11X,^51^ M11 ^52^ and MN15.^53^ (Table 1). The results calculated at the CCSD level of theory are used as reference. Concerning the CASSCF calculations, the number of orbitals in the active space was six for benzene and eight for the rest of the molecules. The number of electrons was chosen to match the number of orbitals used in the active space. However, it was not possible to calculate the frequencies of pyridine at the CASSCF(8,8)/6-311+G(d) and -/Def2SVP level of theory in Gaussian 16. This problem was solved by reducing the active space to (6,6).

List of electronic structure methods and basis sets used in this study

**Table:** 

Electronic structure methods		
Wavefunction based		Reference
CCSD		40–43
CASSCF		37–39
MP2		31–36

**KS-DFT**	**Description/note** ^66^	
LSDA	Local spin density approximation	44
PBE	Generalized gradient approximation	45
PBE0	Correlation corrected PBE	46
B3LYP	Correlation from LYP + VWN III	47
CAM-B3LYP	Long-range corrected	48
wB97XD	Long-range corrected + dispersion	49
M06-2X	Minnesota functional	50
SOGGA11X	Minnesota functional	51
M11	Minnesota functional	52
MN15	Minnesota functional	53

**Table:** 

Basis sets		
Type	Name	Reference
Pople	6-31+G(d), 6-31++G(d,p)	54 and 55
	6-311+G(d), 6-311++G(d,p)	56–58
Karlsruhe	Def2SVP, Def2TZVP	67
	Def2SVPD, Def2TZVPD	59
Dunning's correlation	cc-pVDZ, cc-pVTZ	60
Consistent	aug-cc-pVDZ, aug-cc-pVTZ	61

Pople style,^54–58^ Karlsruhe^59^ and Dunning's correlation consistent^60,61^ (cc) basis sets in both double-*ζ* and triple-*ζ* quality have been utilized for each of the electronic structure methods. Previous studies have shown, that the Pople style basis sets work very well with one set of diffuse and polarized functions added to the heavy atoms.^62,63^ Hence, in this study, the double-*ζ* and triple-*ζ* Pople style basis sets refer to the 6-31+G(d) and 6-311+G(d) basis sets. The Karlsruhe and Dunning's cc basis sets have furthermore been augmented with diffuse functions, and one additional set of diffuse and polarized functions have been added to the hydrogen atoms in the Pople style basis sets (Table 1). It was not possible to perform the CASSCF calculations with the augmented basis sets due to linear dependence, thus they have been neglected.

The electronic structure theory has been specified in the wavefunction files for CCSD, MP2, CASSCF, LSDA, PBE, PBE0, B3LYP and M06-2X.^64^

### 3.2 Wavefunction analysis

The wavefunction partition and topological analysis were performed in the AIMAll software package.^65^ The AIMExt program were used to localize and characterize the critical points, and the AIMInt program were used for the numerical integration of the atomic basins.^65^ All AIMAll-calculations were performed with the same settings.

The output of the numerical integrations were then used in the ESI-3D program^68^ to obtain the aromaticity indexes (see ESI† for method-specification in the AIMAll calculations and for generic input-file in the ESI-3D calculations).

## 4 Results and discussion

In this section, the imaginary frequencies and its consequences are initially discussed, followed by an assessment of the basis set performance. The DFT functionals are then benchmarked by comparison with the results obtained by the correlated wavefunction methods, and the aromaticity in the molecules from this study is finally assessed.

The electronic energy, zero point energy (ZPE) and sum of electronic and zero point energy are obtained in the process of calculating the aromaticity indexes. For future reference, these energetics are reported together with the aromaticity indexes in the ESI,† but they will not be further discussed.

### 4.1 Transition structures

Concerning the predicted transition structures, the observed imaginary frequencies and the associated level of theory used in the calculations are listed in Table 2.

**Table:** List of imaginary frequencies and associated level of theory observed in this study

Transition structures		
Molecule	Level of theory	Frequency
Benzene	CCSD/6-31++G(d,p)	741.75*i*
	-/6-311+G(d)	137.53*i*
	-/6-311++G(d,p)	949.02*i*
	-/Def2SVPD	1245.53*i*
	MP2/6-31++G(d,p)	956.42*i*
	-/6-311+G(d,p)	466.57*i*
	-/6-311++G(d,p)	1190.70*i*
	-/Def2SVPD	1377.96*i*
Pyridine	CASSCF(8,8)/6-31+G(d)	1528.84*i*, 1387.88*i*, 1052.72*i*, 645.89*i*
	-/6-311+G(d,p)	2045.66*i*, 1462.91*i*, 1254.60*i*, 882.73*i*
	-/Def2TZVP	2009.55*i*, 966.02*i*, 589.62*i*, 374.49*i*
	-/cc-pVTZ	952.56*i*, 470.91*i*, 465.01*i*
Pyridazine	MP2/6-311++G(d,p)	223.22*i*
Pyrimidine	CASSCF(8,8)/cc-pVTZ	890.18*i*, 561.23*i*

It is noted, that transition states only were predicted at the correlated wavefunction level of theory and not for the single-determinant based DFT functionals. All the imaginary frequencies are rather large, and simulations in GaussView 6.0.16 ^69^ revealed, that the associated vibrations are out-of-plane. The majority of these surprising results occurred at the MP2 level of theory using the Pople style basis sets. These findings are in agreement with previous studies.^28,29^

Most of the transition structures were predicted for benzene, and it is observed, that those from CCSD and MP2 calculations have in common, that the used basis sets contain diffuse functions. Moreover, all of the transition structures at the CASSCF level of theory were calculated with basis sets of triple-*ζ* quality, expect for the double-*ζ* Pople style basis set. In contrast, a mix of both double-*ζ* and triple-*ζ* basis sets resulted in transition states for the CCSD and MP2 calculations.

Properties calculated utilizing truncated basis sets might be different from the complete basis sets limit. This difference is known as the Basis Set Incompleteness Error (BSIE).^70^ Basis Set Superposition Error (BSSE) effects are introduced, when atom-centered basis functions overlap the function space of another atom, thereby increasing the number of basis functions used for the description of the other atom. This artificial improvement of the basis set counteracts the BSIE and helps compensate for the basis set deficiencies.^71^ BSSE effects are frequently encountered, when two or more molecules are studied. The analog of BSSE effects in single molecules (intramolecular BSSE effects) have similarly been reported.^72^ Both BSIE and the BSSE effects affect the potential energy surface, which introduces artificial effects into energy-based properties, such as frequencies.^29^

The BSIE and BSSE effects are highly entangled, however, it has been proposed that the intramolecular BSSE effects are the cause of the imaginary frequencies in aromatic planar arenes.^29,70^ Treating the imaginary frequencies as caused by intramolecular BSSE effects, the problem can be fixed by introducing a counterpoise correction.^71^ As mentioned in the computational approach section, D. Asturiol *et al.* presented an effective way to partition systems, thereby enabling for counterpoise corrections.^29^

The calculated aromaticity indexes are expected to be of comparable size. If the results obtained at one level of theory are notably different from the general tendencies, it might be an attribute of the intramolecular BSSE effects or an error. However, no such distinct results are observed (see the next two subsections). Hence, it is concluded, that the intramolecular BSSE effects have no or at least small enough effect not to noted in the aromaticity indexes.

### 4.2 Basis set analysis

Only the basis set dependence of the aromaticity indexes are examined in this section. The performance of the basis sets is discussed for each aromaticity index, starting with benzene and then extended to include pyridine and the diazines.

#### 4.2.1 HOMA index

**Fig. 3:**
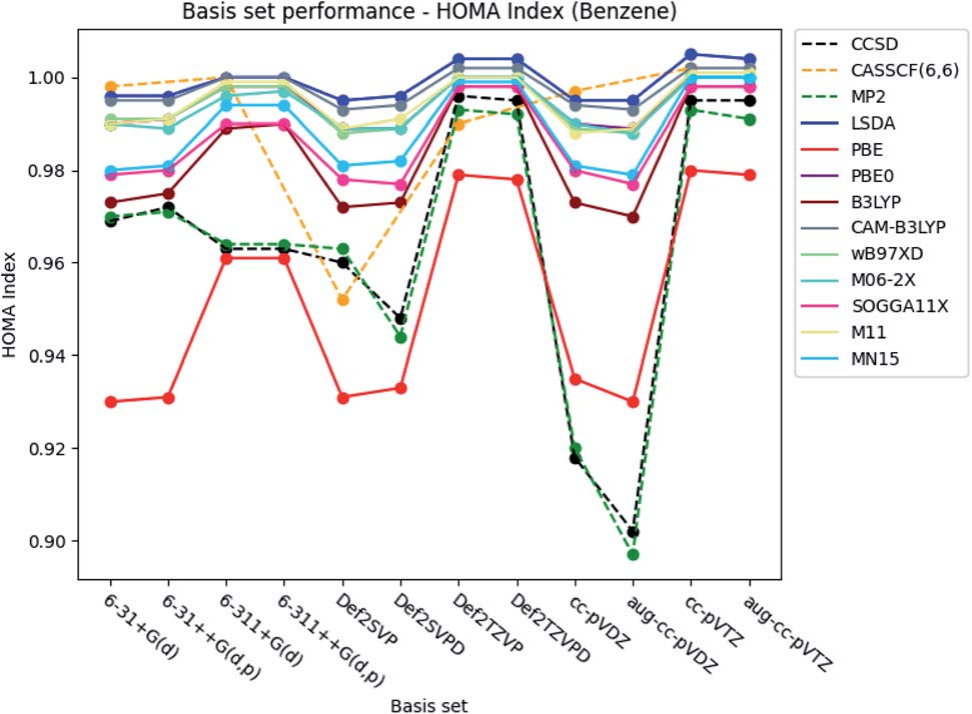
Basis set dependence of the HOMA index for benzene. The basis sets are listed on the horizontal axis, and the HOMA index is given along the vertical axis.

The basis set dependence of the HOMA index for the different electronic structure methods for benzene is illustrated in Fig. 3. A significant increase in the index is in general observed, when the larger triple-*ζ* basis sets are used compared to the corresponding double-*ζ* basis sets. However, the opposite is true for the Pople style basis sets at the CCSD and MP2 level of theory, in which the index is lowered (−∼0.01). The increase in the index is much more significant (+∼0.05) for the Karlsruhe and Dunning's cc basis sets than for the Pople style basis sets (+∼0.015). Exceptions are noted for LSDA, in which the increase, concerning the Pople style basis sets, is of comparable size with the one observed for the other basis sets. In addition, the index is only slightly increased at the CASSCF level of theory for the Pople style and Dunning's cc basis sets, when the larger basis sets are utilized.

Augmentation with diffuse functions for the Karlsruhe and Dunning's cc-basis sets is seen to make no notable difference compared to the values obtained by the ‘bare’ basis sets. The double-*ζ* Karlsruhe and Dunning's cc basis sets at the CCSD and MP2 level of theory are exceptions. In these cases, the index is lowered (−∼0.02), when diffuse functions are included in the basis sets. Addition of diffuse and polarized functions to the hydrogens for the Pople style basis sets is noted to have a negligible effect on the index.

The basis set dependence of the HOMA index is similarly plotted for pyridine and the diazines. The figures are available in the ESI.† Overall, the performance of the basis sets is consistent with the observations done for benzene.

#### 4.2.2 PDI, MCI and AV1245 index

**Fig. 4:**
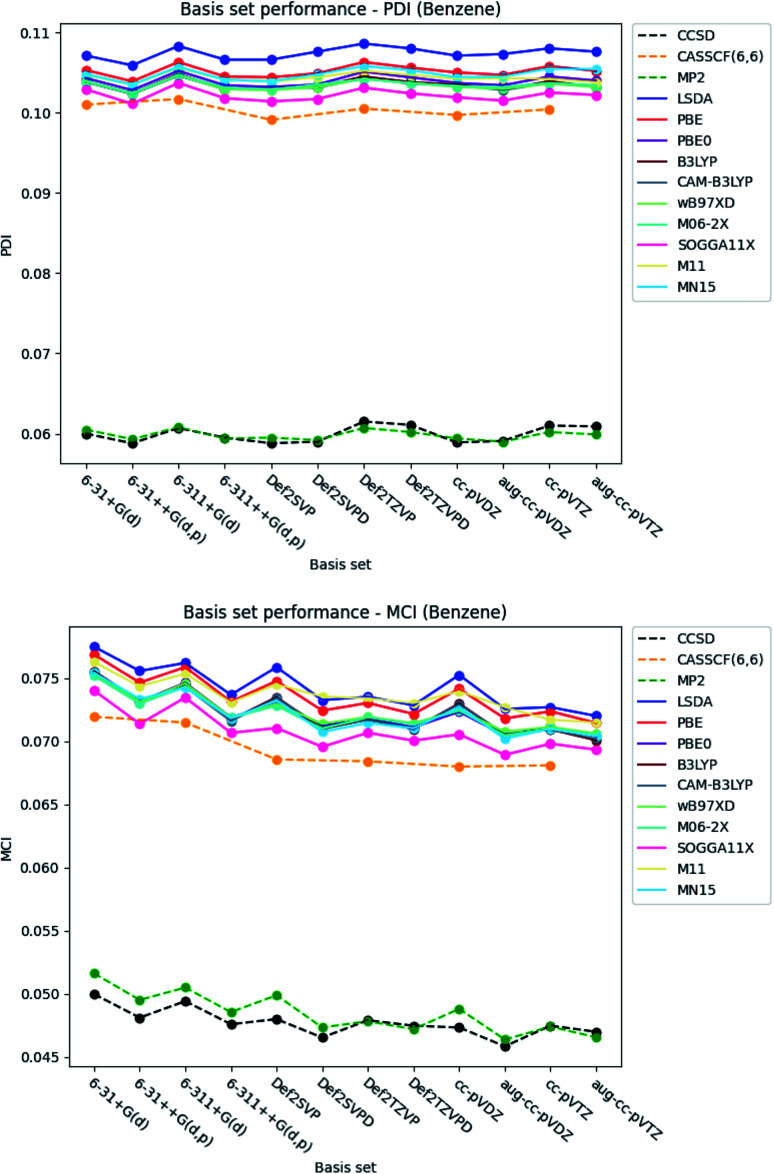
Basis set dependence of the PDI (top) and MCI (bottom) for benzene. The basis sets are listed on the horizontal axis, and the associated property is given along the vertical axis.

**Fig. 5:**
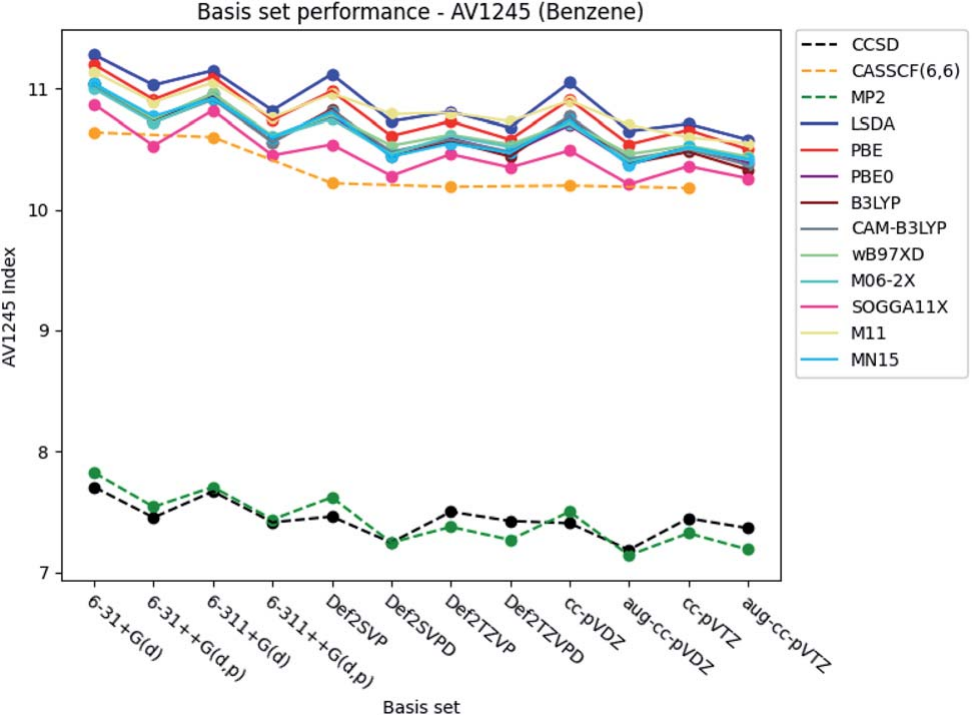
Basis set dependence of the AV1245 index for benzene. The basis sets are listed on the horizontal axis, and the AV1245 index is given along the vertical axis.

It is seen from Fig. 4 and 5, that the basis set dependence of the PDI, MCI and AV1245 index are quite similar to each other. However, the relative change in the index values are different. The basis set dependence of the indexes is the most prominent in the AV1245 index (±∼1), minor in the MCI (±∼0.005) and the least in the PDI (±∼0.002). It is noted, that the index values obtained by the larger triple-*ζ* basis sets are larger than those obtained with the double-*ζ* basis sets for the PDI. The opposite is true for the MCI and AV1245 index. The basis set dependence of the MCI and AV1245 index is expected to be similar, since the AV1245 index is defined in term of the MCI. The PDI, MCI and AV1245 index have in common, that inclusion of diffuse functions to the Karlsruhe and Dunning's cc basis sets lowers the index values. A similar reduction of the indexes occurs when diffuse and polarized functions are added to the hydrogens for the Pople style basis sets. For the Pople style basis sets, this reduction is of comparable size with the change that occurred, when the larger triple-*ζ* basis sets were used. The reduction is rather small for the double-*ζ* Karlsruhe and Dunning's cc basis sets and negligible for the corresponding triple-*ζ* basis sets. The Pople style basis sets do in general predict larger values than the Karlsruhe and Dunning's cc basis sets.

The basis set dependence of the PDI, MCI and AV1245 index for pyridine and the diazines are available in the ESI.† Comparison of the basis set dependencies reveals similar tendencies as described for benzene. Furthermore, it is observed that the PDI tends to converge towards larger values, as each type of basis sets become larger. In contrast, the indexes goes towards smaller values for the MCI and AV1245 index, as each type of basis sets are enlarged.

#### 4.2.3 FLU index

**Fig. 6:**
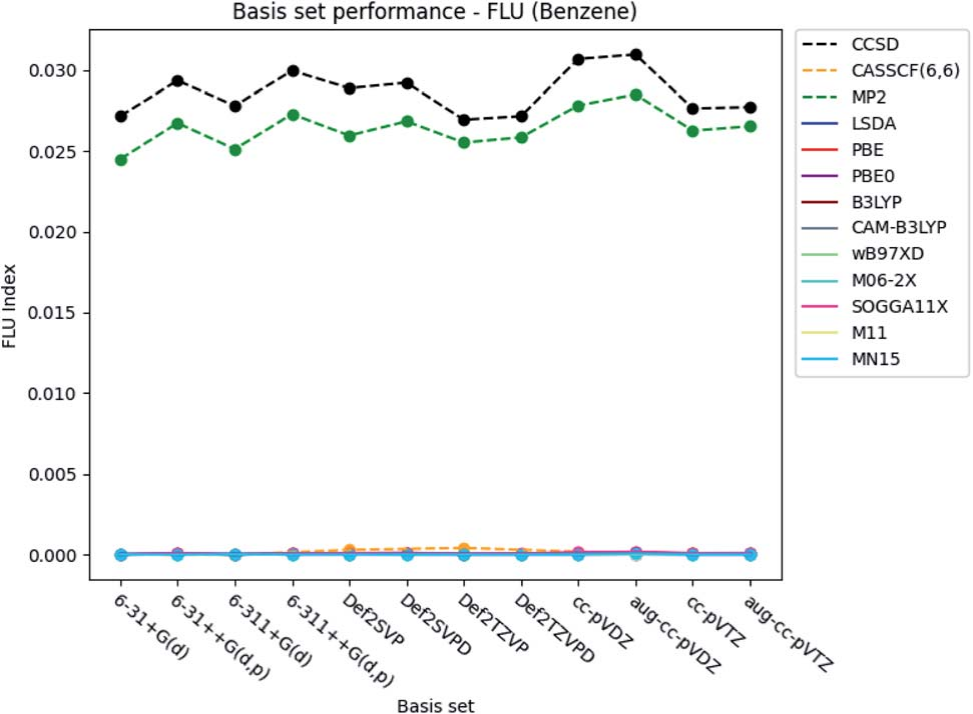
Basis set dependence of the FLU index for benzene. The basis sets are listed on the horizontal axis, and the FLU index is given along the vertical axis.

Fig. 6 shows, that the FLU index is almost independent of the choice of basis set, when the DFT functionals and CASSCF are used for the calculations of benzene. Comparison of the indexes obtained by CCSD and MP2 reveals, that slightly larger values are obtained, when the larger triple-*ζ* basis sets are used compared to the corresponding double-*ζ* Pople style basis sets, and *vice versa* for the Karlsruhe and Dunning's cc basis sets. The changes in the index values obtained by the DFT functionals and CASSCF are negligibly small and are therefore not discussed.

The basis set dependence of the FLU index is available for pyridine and the diazines in the ESI.† It is noted, that the DFT-calculated FLU index is much more basis set dependent for the other molecules than for benzene. In general, the basis set performance for the wavefunction methods are in agreement with the observations for benzene, expect for the increasement in the index when utilizing the Pople style triple-*ζ* basis sets. The index is generally slightly lowered, similarly as for the Karlsruhe and Dunning's cc basis sets. However, concerning the DFT functionals, the index is increased by ∼0.003, when the larger triple-*ζ* Pople style and Karlsruhe basis sets are used. The increasement observed for the Dunning's cc basis sets is negligible. Addition of diffuse and polarized functions to the hydrogens for the Pople style basis sets is seen to increase the index for the wavefunction methods but not for the DFT functionals. A slight increasement is, however, observed for all methods for the double-*ζ* Karlsruhe and Dunning's cc basis sets, when diffuse functions are added.

### 4.3 Benchmarking of DFT functionals

Under the assumption that the aromaticity indexes calculated at the CCSD level of theory are the most accurate, these results can be used as reference. Thus, the results from the DFT functionals are benchmarked by comparison with those from the correlated wavefunction methods.

#### 4.3.1 HOMA index

**Fig. 7:**
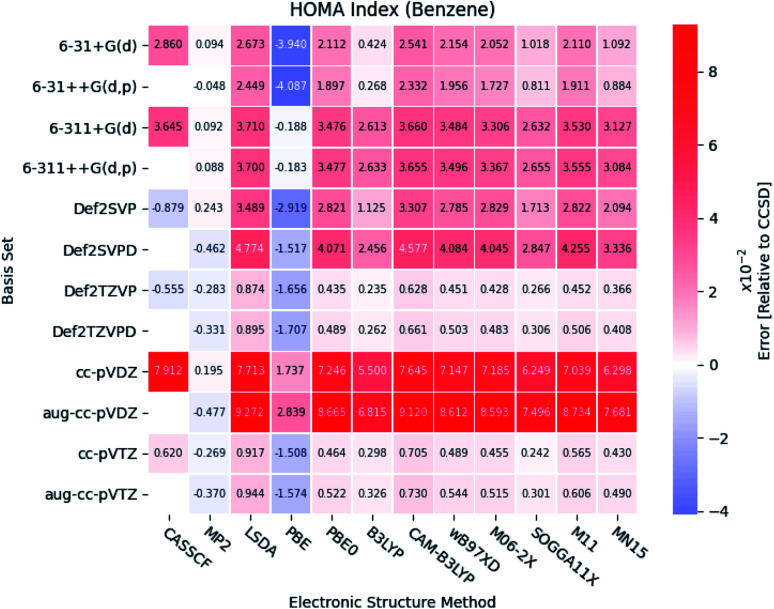
The errors of the calculated HOMA indexes relative to CCSD for benzene. The basis sets and electronic structure methods are listed on the vertical and horizontal axis, respectively. The errors are colored by size.

The relative errors of the HOMA indexes for benzene are illustrated in Fig. 7. It is seen, that the indexes calculated at the MP2 level of theory are very close to the reference results, especially when the Pople style basis sets are utilized. Rather large errors are observed at the CASSCF level of theory, except for the Karlsruhe basis sets. This is highly unexpected. In this study, only one correlated orbital is used per electron in the active space for the CASSCF calculations, similarly as in a previous study.^73^ These large errors and fluctuating behavior of the aromaticity indexes may be a consequence of a too small active space. Aiming for a systematic extension of the active space is unrealistic; previous studies have shown that the convergence is too slow.^74^ The CASSCF calculations were only included in this study, because it, contrary to CCSD and MP2, can provide the molecular orbital coefficients for excited states, which is needed in future studies of excited state aromaticity.

Large errors are similarly observed for all the DFT functionals, when Dunning's double-*ζ* cc basis sets are used. It is noted, that the pure DFT functional, PBE, significantly underestimates the HOMA index, except in combination with the triple-*ζ* Pople style basis sets.

In general, small errors are observed for the hybrid functionals, when the triple-*ζ* Karlsruhe and Dunning's cc basis sets are used. Comparison of the different DFT functionals reveals, that the results from B3LYP are in best agreement with the reference. Next follows the two Minnesota functionals SOGGA11X and MN15. Relative differences between the HOMA indexes for pyridine and the diazines are available in the ESI.† The tendencies found for the other molecules are in agreement with the ones observed for benzene.

A statistical summary of the aromaticity indexes are given in the Tables 41–45 (located in the ESI†). From the tables, it is seen that the DFT functionals in general overestimates the HOMA index, expect for PBE. This is in agreement with the relative placement of the electronic structure methods in Fig. 3. The overestimation stems from the fact, that the DFT functionals in general predict smaller bond lengths than CCSD, which translate into larger HOMA indexes.

The standard deviation observed within CCSD and MP2 is high compared to the DFT functionals, meaning that the choice of basis set is crucial for the accuracy of the HOMA index. However, the standard deviation of the DFT functionals is in general small. This leads to a favoring of the computationally less demanding double-*ζ* basis sets, since the effect of using the more elaborate basis sets is negligible, whereas the computational saving is significant.

#### 4.3.2 ESI

The relative errors of the PDI, MCI, AV1245 index and FLU index are available in the ESI† for all five molecules. It is noted, that all the indexes obtained at the MP2 level of theory are in good agreement with the reference. However, CASSCF and the DFT functionals tend to predict a larger amount of aromaticity in each case. This means, that the PDI, MCI and AV1245 index are larger than the corresponding indexes obtained at the CCSD level of theory, and *vice versa* for the FLU index. The relative errors of the MCI, AV1245 index and FLU index, from the CASSCF calculations of pyridazine, are of comparable size with the errors from the MP2 calculations. The errors of the CASSCF calculations are in general large, in agreement with the observation for the HOMA index. Based on these findings, it is not recommended to use the CASSCF level of theory for proper description of aromaticity. Comparison of the mean values of the DFT-calculated indexes from the Tables 41–45,† and the corresponding indexes obtained at the CCSD/aug-cc-pVTZ level of theory shows, that the DFT functionals in general overestimates the PDI with ∼70%, the MCI with ∼50% and the AV1245 index with ∼45% relative to CCSD. The DFT-predicted FLU indexes are three orders of magnitude smaller than the reference.

**Fig. 8:**
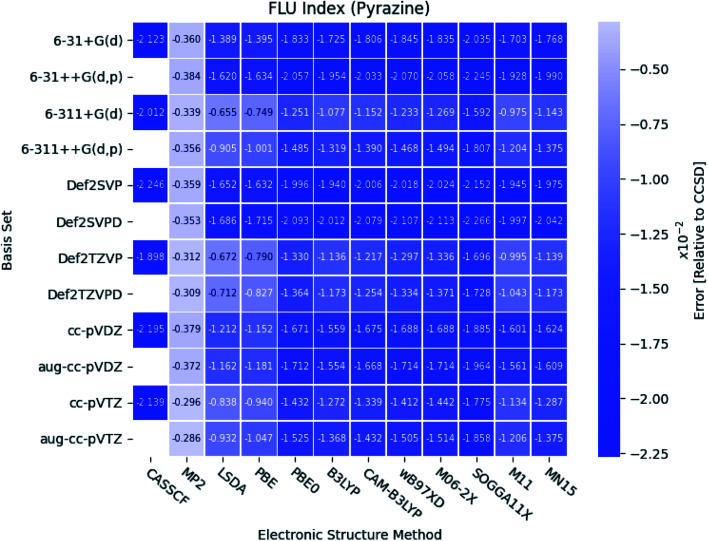
The errors of the calculated FLU indexes relative to CCSD for pyrazine. The basis sets and electronic structure methods are listed on the vertical and horizontal axis, respectively. The errors are colored by size.

As seen in Fig. 8, the FLU indexes calculated with the triple-*ζ* basis sets are in better agreement with the references than the corresponding double-*ζ* basis sets. This trend is only prominent for the diazines, especially in pyrazine. Furthermore, for pyridine and the diazines, the relative errors for the two pure DFT functionals, LSDA and PBE, are observed to be small compared to the other more elaborate DFT functionals. In addition, comparable results are obtained with the B3LYP, M11, MN15 and the two long-range corrected CAM-B3LYP and wB97XD functionals.

With respect to the rest of the ESIs, it is seen that the SOGGA11X functional provides results slightly closer to those from CCSD than the other DFT functionals, closely followed by the M06-2X functional for the MCI and AV1245 index. Since the standard deviation for the MCI, AV1245 index and FLU index is large for the DFT functionals, and because the triple-*ζ* basis sets were found to predict the better results (see also next subsection), it is recommended to use basis sets of triple-*ζ* quality for the calculations of the ESIs.

### 4.4 Assessment of aromaticity

**Scheme 1:**
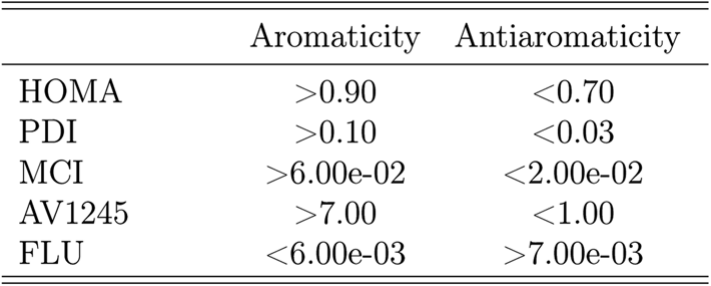
Interpretation scheme used to assign the values of the aromaticity indexes as either aromatic or antiaromatic.

In the following assessment of aromaticity, the aromaticity indexes are interpreted in terms of Scheme 1.^10,75^ According to Hückel's rule,^76^ all five molecules are aromatic in their ground states.

The aromaticity indexes for the five molecules obtained at the CCSD/aug-cc-pVTZ level of theory are listed and interpreted in Table 3. From the table, it is seen, that the HOMA indexes imply, that all five molecules are aromatic, in agreement with Hückel's rule. In contrast, the FLU indexes indicate, that the molecules are antiaromatic. Concerning the PDI, MCI and AV1245 index, only the latter clearly indicates that benzene and pyrazine are aromatic. However, it is noted, that these values are only just large enough to indicate aromaticity. The rest of the indexes can be assigned slightly to both properties.

**Table:** The aromaticity indexes of the five molecules calculated at the CCSD/aug-cc-pVTZ level of theory. The color green indicates aromaticity, the color red indicates antiaromaticity and the color yellow is used for the values that can be assigned to both properties

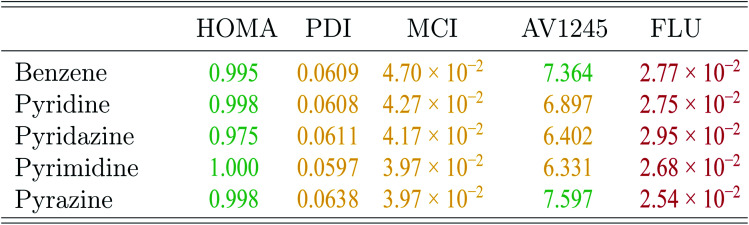

These findings are quite surprising and unexpected. Unfortunately, examination of all calculated aromaticity indexes reveals, that similarly sized index values are obtained in all correlated wavefunction calculations, except in CASSCF calculations due to reasons discussed earlier. The PDI and FLU index of benzene, pyridine and pyrimidine have been calculated at the CISD/6-31G(d) level of theory in a previous study. Comparison of these values with the ones obtained in this study by CCSD and MP2 shows, that the PDI values, in this study, in general is 20% smaller than those previously obtained. Similarly, the FLU indexes, calculated by wavefunction methods in this study, are one order of magnitude larger than those calculated in the previous study.^19^

However, if the attention is directed towards the results obtained by the DFT functionals, then almost all of the calculated aromaticity indexes indicate aromaticity. Only a few of the calculated PDI, MCI and FLU index values are dissimilar in their interpretation, and it is noted, that the majority of them are only just below the threshold of predicting aromaticity. Exceptions are observed for the FLU indexes of the diazines, in which some of the values indicate antiaromaticity. For both the PDI and FLU index, it is seen, that the two pure DFT functionals in combination with the double-*ζ* Pople style and Karlsruhe basis sets in particular predict antiaromatic character. The SOGGA11X functional is similarly observed to predict dissimilar MCI and FLU index values, however, this is expected, since the benchmark study showed, that the results obtained at the SOGGA11X level of theory were in best agreement with the results from CCSD calculations.

**Scheme 2:**
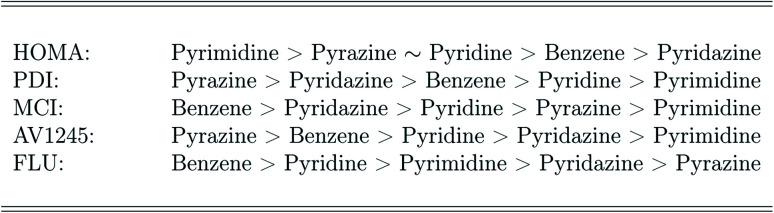
Ranking of the amount of aromaticity in the molecules based on each aromaticity index.

The ranking of the relative amount of aromaticity in the molecules is based on the averaged aromaticity indexes from Tables 41–45,† and it follows from Scheme 2. It is clear from Scheme 2, that the amount of aromaticity relative to each other differ depending on which aromaticity index is used to measure the amount of aromaticity. This is in perfect agreement with previous studies; it is extremely difficult to find a consensus among the aromaticity indexes.^77^ The ability to undergo electrophilic aromatic substitution reactions has previously been used to rank the relative amount of aromaticity between molecules, and a similar ranking, as proposed by the FLU index, has been reported.^78^

It is problematic, that the correlated wavefunction calculations only agree with the DFT functionals in the case of the HOMA index, and that their interpretation of the FLU index is completely different compared to the one based on the DFT calculations. Benzene, pyridine and the diazines are aromatic molecules,^79–85^ meaning that this benchmark study unequivocally demonstrates, that the correlated wavefunction methods in combination with the ESIs fail to describe the aromaticity of the studied molecules. This is very surprising, since CCSD is known to be an extremely accurate and well-balanced method that usually never fails.^74^

The recommend DFT functionals are therefore not the ones in best agreement with the correlated wavefunction methods, but the DFT functionals; wB97XD, CAM-B3LYP and M06-2X, which have been observed to perform very consistently and to predict an appropriate amount of aromaticity throughout this study. In addition, based on these DFT functionals, the relative amount of aromaticity (based on each individual aromaticity index) is in agreement with the averaged ordering observed for the FLU index and the previously reported ranking.^78^

## 5 Conclusion

The amount of aromaticity in the molecules; benzene, pyridine and the diazines in their ground states have been assessed by five different aromaticity indexes, including the HOMA index, PDI, MCI, AV1245 index and FLU index. The performance of the Pople style, Karlsruhe and Dunning's cc basis sets in both double-*ζ* and triple-*ζ* quality, with and without diffuse functions added, have been investigated for their performance with respect to calculation of aromaticity indexes. Furthermore, correlated wavefunction methods, including CCSD, CASSCF and MP2 have been used to benchmark the ten DFT functionals; LSDA, PBE, PBE0, B3LYP, CAM-B3LYP, wB97XD, M06-2X, SOGGA11X, M11 and MN15.

A couple of the optimized structures from correlated wavefunction calculations turned out to be transition structures, as large out-of-plane imaginary frequencies were obtained from frequency calculations. These findings will join the group of surprising results observed for several planar arenes at the correlated wavefunction level of theory, as reported in previous studies.^28,29^

The basis set study showed, that the larger amount of aromaticity were predicted for the HOMA index and the PDI, when the larger triple-*ζ* basis sets were used compared to the corresponding double-*ζ* basis sets. The opposite were observed for the MCI, AV1245 index and FLU index. Addition of diffuse functions to the Karlsruhe and Dunning's cc basis sets were in general observed to lower the amount of aromaticity in the molecules. Similar effects were noted for the inclusion of diffuse and polarized functions to the hydrogens for the Pople style basis sets. However, the effects were often negligible, though mostly appearing for the double-*ζ* Pople style and Karlsruhe basis sets. Based on these findings, it is recommend to save the extra computational time and not include additional diffuse functions in the basis sets.

Comparison of the aromaticity indexes obtained by the different electronic structure methods revealed, that the CASSCF method with only one correlated orbital per electron in the active space performed poorly. In addition, the DFT functionals were found to predict the PDI, MCI and AV1245 index to be ∼70%, ∼50% and ∼45% larger, respectively, compared to CCSD. The DFT-calculated FLU indexes were observed to be three orders of magnitude smaller than the corresponding indexes obtained at correlated wavefunction level of theory. The correlated wavefunction methods and DFT functionals only predicted results of comparable size in their calculations of the HOMA index. Overall, wB97XD, CAM-B3LYP and M06-2X were found to perform the best for the calculations of aromaticity indexes. The pure DFT functionals, LSDA and PBE were conversely found to perform the worst, except for calculation of the FLU index. Examination of the DFT functionals showed a large basis set dependence for the ESIs. It is recommended to use the wB97XD, CAM-B3LYP or M06-2X functional in combination with a simple basis set of triple-*ζ* quality, where ‘simple’ means no inclusion of additional diffuse functions, since their effect was found to be negligible.

Assessment of the aromaticity indexes showed, that the DFT functionals in general predicted all five molecules to be aromatic regardless of which aromaticity index used. Concerning the correlated wavefunction methods, aromaticity was only clearly indicated by the HOMA indexes. Hence, the correlated wavefunction methods in combination with the ESIs failed to describe the aromaticity of the studied molecules, which was highly unexpected.

In order to proceed with the computational studies concerning the importance and effects of build-in aromaticity in photoisomerization reactions,^6,7^ this benchmark study clearly needs to be extended to include excited states. The next papers in this series will be a benchmark study of the aromaticity indexes for the first excited singlet and triplet states and investigations of the effects of solvents on the aromaticity indexes using electronic structure methods including the coupling to the surrounding solvent.^86–89^ When an accurate (and preferable cheap) description of ground state and excited state aromaticity is determined, the amount of aromaticity must be determined for a variety of molecules, allowing for hierarchical ranking of aromatic molecules.

## Conflicts of interest

The authors declare no conflict of interest.

## Supplementary Material

RA-012-D2RA00093H-s001
