# Supplementary material for: A benchmark study of aromaticity indexes for benzene, pyridine and the diazines – I. Ground state aromaticity
Source: RSC Adv. 2022 Jan 19;12(5):2830–42. doi: 10.1039/d2ra00093h (PMC8979194; doi:10.1039/d2ra00093h)
Supplement: RA-012-D2RA00093H-s001 [file RA-012-D2RA00093H-s001.pdf]

Electronic Supplementary Material (ESI) for RSC Advances.
This journal is © The Royal Society of Chemistry 2022

# - Supporting Information -

A benchmark study of aromaticity indexes for benzene, pyridine and the diazines - I. Ground state aromaticity

Jacob Pedersen† and Kurt V. Mikkelsen†*

Table of Contents

A Aromaticity Indexes . . . 2
A.1 Benzene . . . 3
A.1.1 DFT . . . 3
A.1.2 CCSD . . . 8
A.1.3 CASSCF(6,6) . . . 8
A.1.4 MP2 . . . 9
A.2 Pyridine . . . 10
A.2.1 DFT . . . 10
A.2.2 CCSD . . . 15
A.2.3 CASSCF(8,8) . . . 15
A.2.4 MP2 . . . 16
A.3 Pyridazine . . . 17
A.3.1 DFT . . . 17
A.3.2 CCSD . . . 22
A.3.3 CASSCF(8,8) . . . 22
A.3.4 MP2 . . . 23
A.4 Pyrimidine . . . 24
A.4.1 DFT . . . 24
A.4.2 CCSD . . . 29
A.4.3 CASSCF(8,8) . . . 29
A.4.4 MP2 . . . 30
A.5 Pyrazine . . . 31
A.5.1 DFT . . . 31

A.5.2 CCSD 36
A.5.3 CASSCF(8,8) 36
A.5.4 MP2 37
B Statistical Summary of the Aromaticity Indexes 38
C Basis Set Performance Plots 41
C.1 HOMA Index 41
C.2 PDI 44
C.3 MCI 47
C.4 AV1245 Index 50
C.5 FLU Index 53
C.6 Electronic Energy 56
C.7 Zero Point Energy 59
C.8 Sum of Electronic Energy and ZPE 62
D Benchmark Plots 65
D.1 HOMA Index 65
D.2 PDI 68
D.3 MCI 71
D.4 AV1245 Index 74
D.5 FLU Index 77
D.6 Electronic Energy 80
D.7 Zero Point Energy 83
D.8 Sum of Electronic Energy and ZPE 86
E Internal Coordinate Representations 89
F Route Section and Calculation Setup 91

A Aromaticity Indexes

All the calculated aromaticity indexes as well as the electronic energies, zero point energies and sums of electronic and zero point energy for all five molecules in their ground states are listed in the following pages. The aromaticity indexes are all unitless, whereas all energetics are in atomic units. 'Energy' refers to the electronic energy, 'ZPE' refers to the zero point energy and 'Energy+ZPE' refers to the sum of electronic and zero-point energy. The associated molecule is specified in the table label in top of each table, and the level of theory used to obtain the results are specified above each set of results. The MCI and FLU index values are given in scientific notation, where 'Xe-Y' = '$X\cdot 10^{-Y}$'.

A.1 Benzene

A.1.1 DFT

Table 1: Molecule: Benzene

Level of Theory: LSDA

Basis set	HOMA	PDI	MCI	AV1245	FLU	Energy (a.u.)	ZPE (a.u.)	Energy+ZPE (a.u.)
6-31+G(d)	0.996	0.1071	7.75e-02	11.28	3.20e-05	-230.912412	0.098374	-230.814038
6-31++G(d,p)	0.996	0.1059	7.56e-02	11.03	0.00e+00	-230.920585	0.098183	-230.822402
6-311+G(d)	1.000	0.1083	7.62e-02	11.15	3.90e-05	-230.955363	0.097907	-230.857456
6-311++G(d,p)	1.000	0.1066	7.37e-02	10.82	0.00e+00	-230.965773	0.097898	-230.867875
Def2SVP	0.995	0.1066	7.59e-02	11.12	8.00e-06	-230.743074	0.098398	-230.644676
Def2SVPD	0.996	0.1076	7.33e-02	10.73	0.00e+00	-230.756202	0.098321	-230.657881
Def2TZVP	1.004	0.1086	7.35e-02	10.81	3.00e-06	-230.992565	0.097973	-230.894592
Def2TZVPD	1.004	0.1080	7.29e-02	10.68	1.00e-06	-230.993556	0.097942	-230.895614
cc-pVDZ	0.995	0.1071	7.53e-02	11.05	1.00e-06	-230.908588	0.098081	-230.810507
aug-cc-pVDZ	0.995	0.1073	7.26e-02	10.65	1.10e-05	-230.920365	0.097882	-230.822483
cc-pVTZ	1.005	0.1080	7.27e-02	10.71	0.00e+00	-230.991771	0.098116	-230.893655
aug-cc-pVTZ	1.004	0.1076	7.20e-02	10.58	1.00e-06	-230.993461	0.098017	-230.895444

Level of Theory: PBE

Basis set	HOMA	PDI	MCI	AV1245	FLU	Energy (a.u.)	ZPE (a.u.)	Energy+ZPE (a.u.)
6-31+G(d)	0.930	0.1053	7.69e-02	11.20	2.00e-06	-231.944980	0.097945	-231.847035
6-31++G(d,p)	0.931	0.1039	7.47e-02	10.91	5.50e-05	-231.953977	0.097815	-231.856162
6-311+G(d)	0.961	0.1063	7.59e-02	11.10	0.00e+00	-231.984303	0.097500	-231.886803
6-311++G(d,p)	0.961	0.1045	7.32e-02	10.74	5.80e-05	-231.994889	0.097496	-231.897393
Def2SVP	0.931	0.1044	7.48e-02	10.98	3.50e-05	-231.773819	0.098023	-231.675796
Def2SVPD	0.933	0.1049	7.24e-02	10.61	7.10e-05	-231.783717	0.097961	-231.685756
Def2TZVP	0.979	0.1063	7.30e-02	10.73	2.90e-05	-232.019082	0.097433	-231.921649
Def2TZVPD	0.978	0.1056	7.22e-02	10.58	4.40e-05	-232.020148	0.097604	-231.922544
cc-pVDZ	0.935	0.1050	7.42e-02	10.91	8.20e-05	-231.951734	0.097793	-231.853941
aug-cc-pVDZ	0.930	0.1047	7.18e-02	10.54	1.29e-04	-231.963053	0.097395	-231.865658
cc-pVTZ	0.980	0.1058	7.24e-02	10.66	5.60e-05	-232.014668	0.097680	-231.916988
aug-cc-pVTZ	0.979	0.1052	7.15e-02	10.50	7.10e-05	-232.017133	0.097630	-231.919503

Table 2: Molecule: Benzene

Level of Theory: PBE0

Basis set	HOMA	PDI	MCI	AV1245	FLU	Energy (a.u.)	ZPE (a.u.)	Energy+ZPE (a.u.)
6-31+G(d)	0.990	0.1043	7.55e-02	11.04	0.00e+00	-231.972105	0.101367	-231.870738
6-31++G(d,p)	0.991	0.1028	7.31e-02	10.74	3.90e-05	-231.981305	0.101181	-231.880124
6-311+G(d)	0.998	0.1052	7.46e-02	10.96	2.00e-06	-232.009242	0.100850	-231.908392
6-311++G(d,p)	0.998	0.1034	7.19e-02	10.60	3.60e-05	-232.019816	0.100764	-231.919052
Def2SVP	0.989	0.1032	7.30e-02	10.77	2.80e-05	-231.802063	0.101251	-231.700812
Def2SVPD	0.989	0.1035	7.12e-02	10.47	4.90e-05	-231.809265	0.100792	-231.708473
Def2TZVP	1.000	0.1051	7.18e-02	10.59	1.30e-05	-232.045113	0.100687	-231.944426
Def2TZVPD	1.000	0.1044	7.11e-02	10.47	2.20e-05	-232.045901	0.100846	-231.945055
cc-pVDZ	0.990	0.1037	7.24e-02	10.70	6.90e-05	-231.982133	0.101040	-231.881093
aug-cc-pVDZ	0.989	0.1034	7.05e-02	10.39	9.70e-05	-231.990694	0.100622	-231.890072
cc-pVTZ	1.000	0.1045	7.12e-02	10.52	3.30e-05	-232.040612	0.100889	-231.939723
aug-cc-pVTZ	1.000	0.1040	7.04e-02	10.39	4.10e-05	-232.042469	0.100818	-231.941651

Level of Theory: B3LYP

Basis set	HOMA	PDI	MCI	AV1245	FLU	Energy (a.u.)	ZPE (a.u.)	Energy+ZPE (a.u.)
6-31+G(d)	0.973	0.1039	7.56e-02	11.04	1.00e-06	-232.258917	0.100566	-232.158351
6-31++G(d,p)	0.975	0.1024	7.32e-02	10.73	5.50e-05	-232.268439	0.100408	-232.168031
6-311+G(d)	0.989	0.1048	7.45e-02	10.93	0.00e+00	-232.300696	0.100111	-232.200585
6-311++G(d,p)	0.990	0.1029	7.17e-02	10.56	5.90e-05	-232.311295	0.100060	-232.211235
Def2SVP	0.972	0.1030	7.35e-02	10.83	3.20e-05	-232.084722	0.100531	-231.984191
Def2SVPD	0.973	0.1032	7.10e-02	10.44	6.90e-05	-232.094944	0.100688	-231.994256
Def2TZVP	0.998	0.1045	7.17e-02	10.57	2.60e-05	-232.337354	0.100166	-232.237188
Def2TZVPD	0.998	0.1038	7.10e-02	10.44	3.90e-05	-232.338200	0.100339	-232.237861
cc-pVDZ	0.973	0.1035	7.30e-02	10.77	7.80e-05	-232.262946	0.100277	-232.162669
aug-cc-pVDZ	0.970	0.1029	7.05e-02	10.38	1.25e-04	-232.274578	0.099964	-232.174614
cc-pVTZ	0.998	0.1039	7.10e-02	10.48	5.70e-05	-232.333367	0.100385	-232.232982
aug-cc-pVTZ	0.998	0.1032	7.01e-02	10.33	7.00e-05	-232.335567	0.100346	-232.235221

Table 3: Molecule: Benzene

Level of Theory: CAM-B3LYP

Basis set	HOMA	PDI	MCI	AV1245	FLU	Energy (a.u.)	ZPE (a.u.)	Energy+ZPE (a.u.)
6-31+G(d)	0.995	0.1038	7.54e-02	11.02	2.00e-06	-232.114658	0.101784	-232.012874
6-31++G(d,p)	0.995	0.1023	7.31e-02	10.72	2.20e-05	-232.123887	0.101586	-232.022301
6-311+G(d)	1.000	0.1046	7.43e-02	10.91	5.00e-06	-232.157234	0.101327	-232.055907
6-311++G(d,p)	1.000	0.1029	7.16e-02	10.56	2.50e-05	-232.167396	0.101212	-232.066184
Def2SVP	0.993	0.1030	7.34e-02	10.82	9.00e-06	-231.940710	0.101626	-231.839084
Def2SVPD	0.994	0.1031	7.11e-02	10.48	2.80e-05	-231.950770	0.101745	-231.849025
Def2TZVP	1.002	0.1043	7.17e-02	10.58	5.00e-06	-232.193957	0.101306	-232.092651
Def2TZVPD	1.002	0.1036	7.11e-02	10.48	1.00e-05	-232.194686	0.101423	-232.093263
cc-pVDZ	0.994	0.1034	7.29e-02	10.77	3.50e-05	-232.118155	0.101400	-232.016755
aug-cc-pVDZ	0.993	0.1028	7.06e-02	10.42	6.50e-05	-232.129697	0.101117	-232.028580
cc-pVTZ	1.002	0.1037	7.09e-02	10.50	2.20e-05	-232.189902	0.101501	-232.088401
aug-cc-pVTZ	1.002	0.1032	7.02e-02	10.37	2.80e-05	-232.192054	0.101431	-232.090623

Level of Theory: wB97XD

Basis set	HOMA	PDI	MCI	AV1245	FLU	Energy (a.u.)	ZPE (a.u.)	Energy+ZPE (a.u.)
6-31+G(d)	0.991	0.1039	7.54e-02	11.04	0.00e+00	-232.172008	0.101777	-232.070231
6-31++G(d,p)	0.991	0.1024	7.31e-02	10.75	3.20e-05	-232.180851	0.101587	-232.079264
6-311+G(d)	0.998	0.1047	7.46e-02	10.96	2.00e-06	-232.211000	0.101184	-232.109816
6-311++G(d,p)	0.998	0.1029	7.19e-02	10.61	3.20e-05	-232.221024	0.101118	-232.119906
Def2SVP	0.988	0.1028	7.30e-02	10.78	2.40e-05	-232.001441	0.101538	-231.899903
Def2SVPD	0.989	0.1032	7.14e-02	10.53	4.00e-05	-232.008588	0.101376	-231.907212
Def2TZVP	1.000	0.1042	7.19e-02	10.62	1.00e-05	-232.246592	0.101256	-232.145336
Def2TZVPD	1.000	0.1037	7.14e-02	10.54	1.60e-05	-232.247198	0.101310	-232.145888
cc-pVDZ	0.989	0.1032	7.24e-02	10.72	5.90e-05	-232.180421	0.101355	-232.079066
aug-cc-pVDZ	0.988	0.1030	7.08e-02	10.46	7.90e-05	-232.188905	0.101029	-232.087876
cc-pVTZ	1.000	0.1036	7.11e-02	10.53	3.30e-05	-232.241920	0.101422	-232.140498
aug-cc-pVTZ	1.000	0.1032	7.06e-02	10.44	3.80e-05	-232.243844	0.101310	-232.142534

Table 4: Molecule: Benzene

Level of Theory: M06-2X

Basis set	HOMA	PDI	MCI	AV1245	FLU	Energy (a.u.)	ZPE (a.u.)	Energy+ZPE (a.u.)
6-31+G(d)	0.990	0.1039	7.52e-02	11.00	0.00e+00	-232.144424	0.101498	-232.042926
6-31++G(d,p)	0.989	0.1024	7.30e-02	10.72	4.60e-05	-232.152083	0.101259	-232.050824
6-311+G(d)	0.996	0.1047	7.44e-02	10.92	0.00e+00	-232.190265	0.101258	-232.089007
6-311++G(d,p)	0.997	0.1031	7.19e-02	10.60	4.10e-05	-232.198410	0.101083	-232.097327
Def2SVP	0.989	0.1029	7.28e-02	10.75	2.80e-05	-231.968791	0.101413	-231.867378
Def2SVPD	0.989	0.1035	7.10e-02	10.46	5.00e-05	-231.976309	0.101183	-231.875126
Def2TZVP	1.000	0.1042	7.19e-02	10.61	1.80e-05	-232.224623	0.101182	-232.123441
Def2TZVPD	1.000	0.1037	7.14e-02	10.52	2.50e-05	-232.225137	0.101182	-232.123955
cc-pVDZ	0.990	0.1033	7.25e-02	10.71	7.10e-05	-232.162454	0.101086	-232.061368
aug-cc-pVDZ	0.988	0.1032	7.05e-02	10.40	1.01e-04	-232.172150	0.100875	-232.071275
cc-pVTZ	1.000	0.1037	7.10e-02	10.51	4.60e-05	-232.222506	0.101292	-232.121214
aug-cc-pVTZ	1.000	0.1034	7.05e-02	10.41	5.20e-05	-232.224448	0.100994	-232.123454

Level of Theory: SOGGA11X

6-31+G(d)	0.979	0.1029	7.40e-02	10.87	7.00e-06	-232.194690	0.102571	-232.092119
6-31++G(d,p)	0.980	0.1011	7.14e-02	10.53	1.01e-04	-232.205014	0.102326	-232.102688
6-311+G(d)	0.990	0.1037	7.35e-02	10.82	1.00e-06	-232.232397	0.101934	-232.130463
6-311++G(d,p)	0.990	0.1018	7.07e-02	10.45	7.40e-05	-232.242878	0.101810	-232.141068
Def2SVP	0.978	0.1014	7.11e-02	10.54	8.70e-05	-232.021342	0.102332	-231.919010
Def2SVPD	0.977	0.1017	6.96e-02	10.28	1.08e-04	-232.026458	0.101882	-231.924576
Def2TZVP	0.998	0.1031	7.07e-02	10.46	4.30e-05	-232.267630	0.102023	-232.165607
Def2TZVPD	0.998	0.1024	7.01e-02	10.35	5.80e-05	-232.268319	0.102114	-232.166205
cc-pVDZ	0.980	0.1019	7.06e-02	10.49	1.49e-04	-232.209889	0.102124	-232.107765
aug-cc-pVDZ	0.977	0.1015	6.89e-02	10.21	1.71e-04	-232.216717	0.101726	-232.114991
cc-pVTZ	0.998	0.1025	6.98e-02	10.36	8.80e-05	-232.260782	0.102093	-232.158689
aug-cc-pVTZ	0.998	0.1022	6.93e-02	10.26	9.50e-05	-232.263097	0.101850	-232.161247

Table 5: Molecule: Benzene

Level of Theory: M11

Basis set	HOMA	PDI	MCI	AV1245	FLU	Energy (a.u.)	ZPE (a.u.)	Energy+ZPE (a.u.)
6-31+G(d)	0.990	0.1047	7.63e-02	11.14	1.20e-05	-232.093462	0.100747	-231.992715
6-31++G(d,p)	0.991	0.1035	7.44e-02	10.89	4.00e-06	-232.100978	0.100506	-232.000472
6-311+G(d)	0.999	0.1056	7.54e-02	11.05	1.50e-05	-232.138216	0.100167	-232.038049
6-311++G(d,p)	0.999	0.1041	7.31e-02	10.76	5.00e-06	-232.146296	0.100094	-232.046202
Def2SVP	0.989	0.1039	7.45e-02	10.96	0.00e+00	-231.911444	0.100494	-231.810950
Def2SVPD	0.991	0.1044	7.36e-02	10.79	1.00e-06	-231.918549	0.099649	-231.818900
Def2TZVP	1.000	0.1052	7.34e-02	10.80	1.00e-06	-232.179587	0.100692	-232.078895
Def2TZVPD	1.000	0.1047	7.30e-02	10.73	0.00e+00	-232.180698	0.100409	-232.080289
cc-pVDZ	0.988	0.1042	7.40e-02	10.90	1.00e-05	-232.101479	0.100198	-232.001281
aug-cc-pVDZ	0.989	0.1043	7.27e-02	10.70	1.60e-05	-232.110377	0.099885	-232.010492
cc-pVTZ	1.001	0.1042	7.17e-02	10.60	1.20e-05	-232.180997	0.101176	-232.079821
aug-cc-pVTZ	1.001	0.1038	7.15e-02	10.55	1.40e-05	-232.185019	0.100110	-232.084909

Level of Theory: MN15

6-31+G(d)	0.980	0.1048	7.54e-02	11.04	4.00e-06	-231.959035	0.100983	-231.858052
6-31++G(d,p)	0.981	0.1035	7.33e-02	10.77	1.30e-05	-231.966445	0.100739	-231.865706
6-311+G(d)	0.994	0.1057	7.43e-02	10.92	8.00e-06	-231.993678	0.100631	-231.893047
6-311++G(d,p)	0.994	0.1041	7.18e-02	10.60	1.40e-05	-232.002359	0.100605	-231.901754
Def2SVP	0.981	0.1039	7.32e-02	10.80	7.00e-06	-231.750141	0.101266	-231.648875
Def2SVPD	0.982	0.1048	7.08e-02	10.44	2.50e-05	-231.762763	0.101292	-231.661471
Def2TZVP	0.999	0.1058	7.15e-02	10.55	5.00e-06	-232.028399	0.100680	-231.927719
Def2TZVPD	0.999	0.1053	7.10e-02	10.47	9.00e-06	-232.029440	0.100432	-231.929008
cc-pVDZ	0.981	0.1044	7.27e-02	10.74	3.10e-05	-231.958821	0.100790	-231.858031
aug-cc-pVDZ	0.979	0.1045	7.02e-02	10.37	5.90e-05	-231.973033	0.100277	-231.872756
cc-pVTZ	1.000	0.1055	7.10e-02	10.52	1.40e-05	-232.025170	0.100805	-231.924365
aug-cc-pVTZ	1.000	0.1054	7.04e-02	10.42	1.70e-05	-232.030289	0.100598	-231.929691

A.1.2 CCSD

Table 6: Molecule: Benzene

Basis set	HOMA	PDI	MCI	AV1245	FLU	Energy (a.u.)	ZPE (a.u.)	Energy+ZPE (a.u.)
6-31+G(d)	0.969	0.0600	5.00e-02	7.705	2.72e-02	-231.507671	0.099626	-231.408045
6-31++G(d,p)	0.972	0.0588	4.81e-02	7.451	2.94e-02	-231.555888	0.098974	-231.456914
6-311+G(d)	0.963	0.0607	4.94e-02	7.668	2.78e-02	-231.573622	0.097728	-231.475894
6-311++G(d,p)	0.963	0.0595	4.76e-02	7.413	3.00e-02	-231.618261	0.097625	-231.520636
Def2SVP	0.960	0.0588	4.80e-02	7.460	2.89e-02	-231.356875	0.100942	-231.255933
Def2SVPD	0.948	0.0590	4.66e-02	7.244	2.92e-02	-231.390207	0.097484	-231.292723
Def2TZVP	0.996	0.0615	4.79e-02	7.499	2.69e-02	-231.743063	0.100486	-231.642577
Def2TZVPD	0.995	0.0611	4.75e-02	7.424	2.72e-02	-231.747027	0.100248	-231.646779
cc-pVDZ	0.918	0.0589	4.74e-02	7.405	3.07e-02	-231.546045	0.100510	-231.445535
aug-cc-pVDZ	0.902	0.0591	4.59e-02	7.184	3.10e-02	-231.576264	0.099348	-231.476916
cc-pVTZ	0.995	0.0610	4.75e-02	7.446	2.76e-02	-231.753923	0.101074	-231.652849
aug-cc-pVTZ	0.995	0.0609	4.70e-02	7.364	2.77e-02	-231.766868	0.100488	-231.666380

A.1.3 CASSCF(6,6)

Table 7: Molecule: Benzene

Basis set	HOMA	PDI	MCI	AV1245	FLU	Energy (a.u.)	ZPE (a.u.)	Energy+ZPE (a.u.)
6-31+G(d)	0.998	0.1010	7.20e-02	10.64	6.00e-06	-230.750885	0.107340	-230.643545
6-311+G(d)	1.000	0.1017	7.15e-02	10.60	0.00e+00	-230.785806	0.106602	-230.679204
Def2SVP	0.952	0.0991	6.86e-02	10.22	2.90e-04	-230.601590	0.105609	-230.495981
Def2TZVP	0.990	0.1005	6.84e-02	10.19	4.16e-04	-230.829820	0.114912	-230.714908
cc-pVDZ	0.997	0.0997	6.80e-02	10.20	1.75e-04	-230.762620	0.106609	-230.656011
cc-pVTZ	1.002	0.1004	6.81e-02	10.18	6.20e-05	-230.820313	0.107310	-230.713003

A.1.4 MP2

Table 8: Molecule: Benzene

Basis set	HOMA	PDI	MCI	AV1245	FLU	Energy (a.u.)	ZPE (a.u.)	Energy+ZPE (a.u.)
6-31+G(d)	0.970	0.0605	5.16e-02	7.824	2.45e-02	-231.472025	0.099213	-231.372812
6-31++G(d,p)	0.971	0.0593	4.95e-02	7.540	2.67e-02	-231.519692	0.101550	-231.418142
6-311+G(d)	0.964	0.0608	5.05e-02	7.705	2.51e-02	-231.540477	0.097788	-231.442689
6-311++G(d,p)	0.964	0.0594	4.86e-02	7.435	2.73e-02	-231.584255	0.097160	-231.487095
Def2SVP	0.963	0.0595	4.99e-02	7.619	2.60e-02	-231.316557	0.100834	-231.215723
Def2SVPD	0.944	0.0592	4.74e-02	7.244	2.68e-02	-231.354127	0.097276	-231.256851
Def2TZVP	0.993	0.0607	4.78e-02	7.375	2.55e-02	-231.715589	0.100004	-231.615585
Def2TZVPD	0.992	0.0602	4.72e-02	7.267	2.59e-02	-231.720163	0.099840	-231.620323
cc-pVDZ	0.920	0.0594	4.88e-02	7.500	2.78e-02	-231.506454	0.100524	-231.405930
aug-cc-pVDZ	0.897	0.0590	4.64e-02	7.139	2.85e-02	-231.540220	0.099262	-231.440958
cc-pVTZ	0.993	0.0602	4.74e-02	7.322	2.63e-02	-231.729479	0.100719	-231.628760
aug-cc-pVTZ	0.991	0.0599	4.66e-02	7.187	2.65e-02	-231.744661	0.100086	-231.644575

A.2 Pyridine

A.2.1 DFT

Table 9: Molecule: Pyridine

Level of Theory: LSDA

Basis set	HOMA	PDI	MCI	AV1245	FLU	Energy (a.u.)	ZPE (a.u.)	Energy+ZPE (a.u.)
6-31+G(d)	0.998	0.1064	7.14e-02	10.57	5.19e-03	-246.885495	0.086960	-246.798535
6-31++G(d,p)	0.999	0.1055	7.01e-02	10.39	4.99e-03	-246.891944	0.086770	-246.805174
6-311+G(d)	1.001	0.1079	7.20e-02	10.62	7.77e-03	-246.933708	0.086531	-246.847177
6-311++G(d,p)	1.001	0.1067	7.01e-02	10.38	7.37e-03	-246.941846	0.086415	-246.855431
Def2SVP	0.996	0.1059	6.99e-02	10.44	4.46e-03	-246.699945	0.086892	-246.613053
Def2SVPD	0.997	0.1075	6.76e-02	10.14	4.76e-03	-246.716639	0.086837	-246.629802
Def2TZVP	1.006	0.1086	6.94e-02	10.32	7.95e-03	-246.970576	0.086639	-246.883937
Def2TZVPD	1.006	0.1083	6.89e-02	10.23	7.85e-03	-246.971797	0.086594	-246.885203
cc-pVDZ	0.997	0.1068	7.05e-02	10.49	5.99e-03	-246.881141	0.086566	-246.794575
aug-cc-pVDZ	0.998	0.1075	6.84e-02	10.18	6.53e-03	-246.895940	0.086467	-246.809473
cc-pVTZ	1.006	0.1081	6.86e-02	10.24	7.40e-03	-246.968158	0.086603	-246.881555
aug-cc-pVTZ	1.006	0.1079	6.80e-02	10.14	7.19e-03	-246.970731	0.086551	-246.884180

Level of Theory: PBE

Basis set	HOMA	PDI	MCI	AV1245	FLU	Energy (a.u.)	ZPE (a.u.)	Energy+ZPE (a.u.)
6-31+G(d)	0.954	0.1050	7.16e-02	10.56	5.26e-03	-247.978616	0.086436	-247.892180
6-31++G(d,p)	0.955	0.1039	7.00e-02	10.35	5.07e-03	-247.985807	0.086288	-247.899519
6-311+G(d)	0.975	0.1062	7.22e-02	10.61	7.38e-03	-248.023275	0.086044	-247.937231
6-311++G(d,p)	0.974	0.1049	7.01e-02	10.34	7.02e-03	-248.031580	0.085943	-247.945637
Def2SVP	0.952	0.1042	7.00e-02	10.41	4.70e-03	-247.790780	0.086358	-247.704422
Def2SVPD	0.956	0.1053	6.77e-02	10.11	4.77e-03	-247.804249	0.086238	-247.718011
Def2TZVP	0.987	0.1065	6.94e-02	10.29	7.46e-03	-248.057518	0.086083	-247.971435
Def2TZVPD	0.986	0.1061	6.88e-02	10.18	7.38e-03	-248.058750	0.086090	-247.972660
cc-pVDZ	0.954	0.1051	7.05e-02	10.45	6.39e-03	-247.984586	0.086121	-247.898465
aug-cc-pVDZ	0.953	0.1052	6.85e-02	10.14	6.58e-03	-247.999091	0.085982	-247.913109
cc-pVTZ	0.988	0.1061	6.88e-02	10.23	7.03e-03	-248.051184	0.086084	-247.965100
aug-cc-pVTZ	0.987	0.1057	6.79e-02	10.09	6.76e-03	-248.054583	0.086049	-247.968534

Table 10: Molecule: Pyridine

Level of Theory: PBE0

Basis set	HOMA	PDI	MCI	AV1245	FLU	Energy (a.u.)	ZPE (a.u.)	Energy+ZPE (a.u.)
6-31+G(d)	0.996	0.1030	6.79e-02	10.20	2.83e-03	-247.997355	0.089632	-247.907723
6-31++G(d,p)	0.996	0.1018	6.63e-02	9.979	2.73e-03	-248.004762	0.089427	-247.915335
6-311+G(d)	0.999	0.1043	6.90e-02	10.30	4.69e-03	-248.040069	0.089188	-247.950881
6-311++G(d,p)	0.999	0.1029	6.70e-02	10.03	4.42e-03	-248.048394	0.089028	-247.959366
Def2SVP	0.993	0.1021	6.64e-02	10.04	2.71e-03	-247.810696	0.089450	-247.721246
Def2SVPD	0.994	0.1032	6.44e-02	9.786	2.71e-03	-247.821605	0.089219	-247.732386
Def2TZVP	1.001	0.1044	6.62e-02	9.967	4.63e-03	-248.075727	0.089124	-247.986603
Def2TZVPD	1.001	0.1040	6.57e-02	9.881	4.57e-03	-248.076639	0.089131	-247.987508
cc-pVDZ	0.995	0.1029	6.68e-02	10.06	3.65e-03	-248.007126	0.089227	-247.917899
aug-cc-pVDZ	0.995	0.1030	6.51e-02	9.807	3.80e-03	-248.018834	0.089042	-247.929792
cc-pVTZ	1.001	0.1040	6.56e-02	9.907	4.40e-03	-248.069461	0.089122	-247.980339
aug-cc-pVTZ	1.001	0.1037	6.49e-02	9.795	4.20e-03	-248.072159	0.089084	-247.983075

Level of Theory: B3LYP

Basis set	HOMA	PDI	MCI	AV1245	FLU	Energy (a.u.)	ZPE (a.u.)	Energy+ZPE (a.u.)
6-31+G(d)	0.985	0.1029	6.91e-02	10.29	3.51e-03	-248.295790	0.088872	-248.206918
6-31++G(d,p)	0.986	0.1018	6.74e-02	10.07	3.38e-03	-248.303433	0.088704	-248.214729
6-311+G(d)	0.995	0.1042	6.99e-02	10.37	5.67e-03	-248.342933	0.088455	-248.254478
6-311++G(d,p)	0.995	0.1028	6.78e-02	10.01	5.36e-03	-248.351256	0.088327	-248.262929
Def2SVP	0.983	0.1022	6.76e-02	10.16	3.09e-03	-248.105301	0.088717	-248.016584
Def2SVPD	0.985	0.1030	6.52e-02	9.849	3.22e-03	-248.118865	0.088631	-248.030234
Def2TZVP	0.999	0.1043	6.72e-02	10.05	5.71e-03	-248.378996	0.088602	-248.290394
Def2TZVPD	0.999	0.1038	6.66e-02	9.957	5.63e-03	-248.380004	0.088610	-248.291394
cc-pVDZ	0.984	0.1030	6.82e-02	10.21	4.35e-03	-248.299525	0.088465	-248.211060
aug-cc-pVDZ	0.983	0.1030	6.62e-02	9.899	4.72e-03	-248.314153	0.088340	-248.225813
cc-pVTZ	1.000	0.1037	6.64e-02	9.975	5.31e-03	-248.373197	0.088608	-248.284589
aug-cc-pVTZ	1.000	0.1033	6.57e-02	9.849	5.10e-03	-248.376249	0.088579	-248.287670

Table 11: Molecule: Pyridine

Level of Theory: CAM-B3LYP

Basis set	HOMA	PDI	MCI	AV1245	FLU	Energy (a.u.)	ZPE (a.u.)	Energy+ZPE (a.u.)
6-31+G(d)	0.998	0.1024	6.82e-02	10.21	3.02e-03	-248.154012	0.090031	-248.063981
6-31++G(d,p)	0.998	0.1013	6.66e-02	9.994	2.89e-03	-248.161429	0.089831	-248.071598
6-311+G(d)	1.000	0.1037	6.92e-02	10.29	5.23e-03	-248.202043	0.089603	-248.112440
6-311++G(d,p)	1.000	0.1024	6.72e-02	10.04	4.92e-03	-248.210037	0.089435	-248.120602
Def2SVP	0.996	0.1018	6.70e-02	10.10	2.68e-03	-247.963862	0.089789	-247.874073
Def2SVPD	0.997	0.1026	6.48e-02	9.817	2.81e-03	-247.977610	0.089676	-247.887934
Def2TZVP	1.004	0.1037	6.66e-02	9.997	5.24e-03	-248.238405	0.089677	-248.148728
Def2TZVPD	1.004	0.1033	6.61e-02	9.924	5.17e-03	-248.239367	0.089664	-248.149703
cc-pVDZ	0.998	0.1025	6.74e-02	10.13	3.67e-03	-248.157257	0.089558	-248.067699
aug-cc-pVDZ	0.997	0.1024	6.56e-02	9.855	4.07e-03	-248.172047	0.089431	-248.082616
cc-pVTZ	1.004	0.1032	6.59e-02	9.930	4.89e-03	-248.232515	0.089679	-248.142836
aug-cc-pVTZ	1.004	0.1028	6.52e-02	9.819	4.69e-03	-248.235578	0.089635	-248.145943

Level of Theory: wB97XD

6-31+G(d)	0.996	0.1025	6.81e-02	10.21	2.79e-03	-248.203755	0.089935	-248.113820
6-31++G(d,p)	0.996	0.1014	6.65e-02	9.998	2.68e-03	-248.210836	0.089739	-248.121097
6-311+G(d)	0.999	0.1037	6.92e-02	10.31	4.80e-03	-248.248069	0.089420	-248.158649
6-311++G(d,p)	1.000	0.1024	6.72e-02	10.06	4.52e-03	-248.255939	0.089293	-248.166646
Def2SVP	0.993	0.1017	6.65e-02	10.05	2.61e-03	-248.016503	0.089686	-247.926817
Def2SVPD	0.994	0.1026	6.48e-02	9.834	2.66e-03	-248.027224	0.089487	-247.937737
Def2TZVP	1.001	0.1035	6.66e-02	10.01	4.81e-03	-248.283477	0.089529	-248.193948
Def2TZVPD	1.001	0.1032	6.62e-02	9.954	4.74e-03	-248.284282	0.089509	-248.194773
cc-pVDZ	0.995	0.1024	6.70e-02	10.08	3.59e-03	-248.211971	0.089456	-248.122515
aug-cc-pVDZ	0.995	0.1024	6.55e-02	9.868	3.82e-03	-248.223463	0.089298	-248.134165
cc-pVTZ	1.001	0.1031	6.59e-02	9.940	4.51e-03	-248.277037	0.089541	-248.187496
aug-cc-pVTZ	1.001	0.1028	6.54e-02	9.857	4.32e-03	-248.279786	0.089487	-248.190299

Table 12: Molecule: Pyridine

Level of Theory: M06-2X

Basis set	HOMA	PDI	MCI	AV1245	FLU	Energy (a.u.)	ZPE (a.u.)	Energy+ZPE (a.u.)
6-31+G(d)	0.995	0.1024	6.78e-02	10.18	2.75e-03	-248.180065	0.089812	-248.090253
6-31++G(d,p)	0.995	0.1013	6.62e-02	9.977	2.66e-03	-248.186270	0.089571	-248.096699
6-311+G(d)	0.999	0.1036	6.88e-02	10.27	4.51e-03	-248.231506	0.089551	-248.141955
6-311++G(d,p)	0.999	0.1024	6.70e-02	10.04	4.29e-03	-248.237941	0.089316	-248.148625
Def2SVP	0.993	0.1017	6.62e-02	10.03	2.50e-03	-247.988567	0.089654	-247.898913
Def2SVPD	0.994	0.1028	6.42e-02	9.785	2.56e-03	-247.999628	0.089443	-247.910185
Def2TZVP	1.000	0.1034	6.62e-02	9.982	4.50e-03	-248.265660	0.089506	-248.176154
Def2TZVPD	1.000	0.1032	6.58e-02	9.916	4.44e-03	-248.266398	0.089479	-248.176919
cc-pVDZ	0.995	0.1024	6.68e-02	10.08	3.48e-03	-248.199250	0.089370	-248.109880
aug-cc-pVDZ	0.994	0.1026	6.51e-02	9.837	3.72e-03	-248.211517	0.089270	-248.122247
cc-pVTZ	1.000	0.1030	6.55e-02	9.902	4.26e-03	-248.262435	0.089507	-248.172928
aug-cc-pVTZ	1.000	0.1029	6.49e-02	9.821	4.16e-03	-248.265211	0.089407	-248.175804

Level of Theory: SOGGA11X

6-31+G(d)	0.990	0.1010	6.52e-02	9.902	1.75e-03	-248.221828	0.090632	-248.131196
6-31++G(d,p)	0.990	0.0997	6.33e-02	9.654	1.77e-03	-248.230194	0.090365	-248.139829
6-311+G(d)	0.995	0.1023	6.65e-02	10.02	2.91e-03	-248.264975	0.090111	-248.174864
6-311++G(d,p)	0.996	0.1009	6.45e-02	9.757	2.77e-03	-248.273222	0.089913	-248.183309
Def2SVP	0.987	0.1001	6.37e-02	9.738	1.93e-03	-248.030670	0.090430	-247.940240
Def2SVPD	0.988	0.1009	6.19e-02	9.502	1.85e-03	-248.039505	0.090098	-247.949407
Def2TZVP	0.999	0.1020	6.35e-02	9.686	2.73e-03	-248.300093	0.090220	-248.209873
Def2TZVPD	0.999	0.1016	6.30e-02	9.606	2.69e-03	-248.300891	0.090224	-248.210667
cc-pVDZ	0.990	0.1008	6.41e-02	9.760	2.55e-03	-248.236484	0.090199	-248.146285
aug-cc-pVDZ	0.988	0.1007	6.24e-02	9.518	2.51e-03	-248.246368	0.089973	-248.156395
cc-pVTZ	0.999	0.1015	6.28e-02	9.604	2.66e-03	-248.291498	0.090159	-248.201339
aug-cc-pVTZ	0.999	0.1013	6.22e-02	9.510	2.51e-03	-248.294603	0.090073	-248.204530

Table 13: Molecule: Pyridine

Level of Theory: M11

Basis set	HOMA	PDI	MCI	AV1245	FLU	Energy (a.u.)	ZPE (a.u.)	Energy+ZPE (a.u.)
6-31+G(d)	0.996	0.1029	6.88e-02	10.29	3.37e-03	-248.134960	0.089214	-248.045746
6-31++G(d,p)	0.996	0.1020	6.74e-02	10.11	3.23e-03	-248.141012	0.088992	-248.052020
6-311+G(d)	0.999	0.1043	7.00e-02	10.41	5.95e-03	-248.185737	0.088688	-248.097049
6-311++G(d,p)	0.999	0.1032	6.84e-02	10.20	5.67e-03	-248.192112	0.088555	-248.103557
Def2SVP	0.993	0.1024	6.75e-02	10.19	2.82e-03	-247.936181	0.088957	-247.847224
Def2SVPD	0.994	0.1034	6.62e-02	10.03	3.04e-03	-247.947610	0.088703	-247.858907
Def2TZVP	1.002	0.1042	6.81e-02	10.19	6.19e-03	-248.227139	0.089106	-248.138033
Def2TZVPD	1.001	0.1040	6.77e-02	10.14	6.04e-03	-248.228777	0.088998	-248.139779
cc-pVDZ	0.994	0.1029	6.79e-02	10.21	3.82e-03	-248.142678	0.088680	-248.053998
aug-cc-pVDZ	0.995	0.1032	6.68e-02	10.05	4.35e-03	-248.155263	0.088542	-248.066721
cc-pVTZ	1.003	0.1035	6.69e-02	10.06	5.76e-03	-248.226584	0.089316	-248.137268
aug-cc-pVTZ	1.003	0.1033	6.66e-02	10.00	5.64e-03	-248.231697	0.089006	-248.142691

Level of Theory: MN15

6-31+G(d)	0.990	0.1036	6.87e-02	10.28	3.22e-03	-247.986874	0.089356	-247.897518
6-31++G(d,p)	0.990	0.1026	6.73e-02	10.08	3.11e-03	-247.992735	0.089139	-247.903596
6-311+G(d)	0.998	0.1049	6.94e-02	10.33	5.29e-03	-248.026645	0.089001	-247.937644
6-311++G(d,p)	0.998	0.1037	6.77e-02	10.10	5.01e-03	-248.033390	0.088871	-247.944519
Def2SVP	0.989	0.1029	6.72e-02	10.13	2.85e-03	-247.762762	0.089467	-247.673295
Def2SVPD	0.990	0.1043	6.50e-02	9.845	3.01e-03	-247.778580	0.089358	-247.689222
Def2TZVP	1.000	0.1053	6.69e-02	10.04	5.62e-03	-248.062328	0.089117	-247.973211
Def2TZVPD	1.000	0.1051	6.66e-02	9.981	5.56e-03	-248.063583	0.088997	-247.974586
cc-pVDZ	0.990	0.1037	6.76e-02	10.16	3.94e-03	-247.988216	0.089086	-247.899130
aug-cc-pVDZ	0.989	0.1042	6.57e-02	9.878	4.37e-03	-248.004811	0.088853	-247.915958
cc-pVTZ	1.000	0.1050	6.63e-02	9.990	5.14e-03	-248.056754	0.089073	-247.967681
aug-cc-pVTZ	1.000	0.1051	6.57e-02	9.902	4.97e-03	-248.062281	0.088978	-247.973303

A.2.2 CCSD

Table 14: Molecule: Pyridine

Basis set	HOMA	PDI	MCI	AV1245	FLU	Energy (a.u.)	ZPE (a.u.)	Energy+ZPE (a.u.)
6-31+G(d)	0.982	0.0597	4.52e-02	7.159	2.73e-02	-247.527516	0.088744	-247.438772
6-31++G(d,p)	0.983	0.0587	4.38e-02	6.967	2.95e-02	-247.567324	0.088828	-247.478496
6-311+G(d)	0.980	0.0605	4.56e-02	7.218	2.64e-02	-247.599571	0.088032	-247.511539
6-311++G(d,p)	0.979	0.0595	4.43e-02	7.027	2.85e-02	-247.636356	0.087800	-247.548556
Def2SVP	0.977	0.0587	4.40e-02	7.025	2.91e-02	-247.350720	0.089581	-247.261139
Def2SVPD	0.971	0.0594	4.23e-02	6.811	2.97e-02	-247.390290	0.089014	-247.301276
Def2TZVP	0.999	0.0612	4.37e-02	7.020	2.63e-02	-247.770551	0.089352	-247.681199
Def2TZVPD	0.999	0.0610	4.33e-02	6.966	2.66e-02	-247.775009	0.089235	-247.685774
cc-pVDZ	0.950	0.0591	4.42e-02	7.038	2.87e-02	-247.557630	0.089032	-247.468598
aug-cc-pVDZ	0.941	0.0592	4.26e-02	6.821	2.92e-02	-247.593916	0.088529	-247.505387
cc-pVTZ	0.999	0.0609	4.33e-02	6.979	2.72e-02	-247.778043	0.089556	-247.688487
aug-cc-pVTZ	0.998	0.0608	4.27e-02	6.897	2.75e-02	-	-	-

A.2.3 CASSCF(8,8)

Table 15: Molecule: Pyridine

Basis set	HOMA	PDI	MCI	AV1245	FLU	Energy (a.u.)	ZPE (a.u.)	Energy+ZPE (a.u.)
6-31+G(d)	0.990	0.0988	6.22e-02	9.698	9.18e-04	-246.800885	0.092623	-246.708262
6-311+G(d)	0.997	0.0994	6.18e-02	9.592	1.27e-03	-246.819317	0.086129	-246.733188
Def2SVP	0.982	0.0977	6.20e-02	9.623	1.98e-03	-246.617886	0.092658	-246.525228
Def2TZVP	1.002	0.0988	5.85e-02	9.232	1.29e-03	-246.859006	0.087685	-246.771321
cc-pVDZ	0.996	0.0977	5.92e-02	9.303	1.55e-03	-246.788726	0.090492	-246.698234
cc-pVTZ	1.002	0.0985	5.81e-02	9.198	1.41e-03	-246.852624	0.092328	-246.760296

A.2.4 MP2

Table 16: Molecule: Pyridine

Basis set	HOMA	PDI	MCI	AV1245	FLU	Energy (a.u.)	ZPE (a.u.)	Energy+ZPE (a.u.)
6-31+G(d)	0.980	0.0611	4.73e-02	7.379	2.39e-02	-247.498389	0.088698	-247.409691
6-31++G(d,p)	0.982	0.0601	4.58e-02	7.182	2.59e-02	-247.537474	0.088253	-247.449221
6-311+G(d)	0.978	0.0617	4.74e-02	7.399	2.31e-02	-247.573326	0.087932	-247.485394
6-311++G(d,p)	0.978	0.0607	4.60e-02	7.200	2.50e-02	-247.609267	0.087155	-247.522112
Def2SVP	0.978	0.0603	4.63e-02	7.277	2.55e-02	-247.316620	0.089532	-247.227088
Def2SVPD	0.969	0.0607	4.37e-02	6.942	2.64e-02	-247.360523	0.088978	-247.271545
Def2TZVP	0.997	0.0616	4.46e-02	7.071	2.38e-02	-247.749979	0.089004	-247.660975
Def2TZVPD	0.996	0.0613	4.41e-02	6.990	2.41e-02	-247.755070	0.088879	-247.666191
cc-pVDZ	0.949	0.0606	4.63e-02	7.259	2.52e-02	-247.524428	0.089039	-247.435389
aug-cc-pVDZ	0.935	0.0604	4.39e-02	6.933	2.60e-02	-247.564377	0.088453	-247.475924
cc-pVTZ	0.996	0.0613	4.42e-02	7.032	2.46e-02	-247.760122	0.089218	-247.670904
aug-cc-pVTZ	0.996	0.0611	4.34e-02	6.906	2.51e-02	-247.777517	0.088986	-247.688531

A.3 Pyridazine

A.3.1 DFT

Table 17: Molecule: Pyridazine

Level of Theory: LSDA

Basis set	HOMA	PDI	MCI	AV1245	FLU	Energy (a.u.)	ZPE (a.u.)	Energy+ZPE (a.u.)
6-31+G(d)	0.992	0.1083	7.19e-02	10.08	7.65e-03	-262.824970	0.074602	-262.750368
6-31++G(d,p)	0.992	0.1076	7.10e-02	9.966	7.38e-03	-262.830041	0.074369	-262.755672
6-311+G(d)	0.993	0.1095	7.31e-02	10.18	1.05e-02	-262.877345	0.074177	-262.803168
6-311++G(d,p)	0.993	0.1087	7.17e-02	10.03	9.89e-03	-262.883743	0.073940	-262.809803
Def2SVP	0.995	0.1079	7.12e-02	10.07	6.92e-03	-262.621723	0.074615	-262.547108
Def2SVPD	0.996	0.1097	6.87e-02	9.738	6.73e-03	-262.641073	0.074554	-262.566519
Def2TZVP	0.997	0.1104	7.06e-02	9.930	1.03e-02	-262.915968	0.074384	-262.841584
Def2TZVPD	0.998	0.1104	7.01e-02	9.853	1.01e-02	-262.917505	0.074336	-262.843169
cc-pVDZ	0.993	0.1089	7.19e-02	10.14	8.61e-03	-262.821376	0.074252	-262.747124
aug-cc-pVDZ	0.993	0.1098	6.98e-02	9.826	8.75e-03	-262.839199	0.074246	-262.764953
cc-pVTZ	0.996	0.1101	6.99e-02	9.876	9.65e-03	-262.912172	0.074341	-262.837831
aug-cc-pVTZ	0.996	0.1101	6.93e-02	9.763	9.37e-03	-262.915568	0.074257	-262.841311

Level of Theory: PBE

6-31+G(d)	0.943	0.1071	7.25e-02	10.13	8.32e-03	-263.980813	0.073968	-263.906845
6-31++G(d,p)	0.944	0.1063	7.13e-02	9.992	8.00e-03	-263.986491	0.073778	-263.912713
6-311+G(d)	0.961	0.1081	7.35e-02	10.22	1.07e-02	-264.029838	0.073577	-263.956261
6-311++G(d,p)	0.961	0.1072	7.20e-02	10.04	1.01e-02	-264.036374	0.073368	-263.963006
Def2SVP	0.953	0.1065	7.16e-02	10.11	7.62e-03	-263.775347	0.073982	-263.701365
Def2SVPD	0.957	0.1079	6.92e-02	9.771	7.14e-03	-263.791338	0.073903	-263.717435
Def2TZVP	0.974	0.1087	7.08e-02	9.936	1.03e-02	-264.065304	0.073700	-263.991604
Def2TZVPD	0.974	0.1085	7.03e-02	9.848	1.01e-02	-264.066806	0.073705	-263.993101
cc-pVDZ	0.944	0.1075	7.22e-02	10.16	9.54e-03	-263.987451	0.073675	-263.913776
aug-cc-pVDZ	0.944	0.1079	7.01e-02	9.836	9.23e-03	-264.005033	0.073607	-263.931426
cc-pVTZ	0.972	0.1084	7.03e-02	9.897	9.83e-03	-264.057378	0.073687	-263.983691
aug-cc-pVTZ	0.973	0.1082	6.95e-02	9.762	9.46e-03	-264.061513	0.073652	-263.987861

Table 18: Molecule: Pyridazine

Level of Theory: PBE0

Basis set	HOMA	PDI	MCI	AV1245	FLU	Energy (a.u.)	ZPE (a.u.)	Energy+ZPE (a.u.)
6-31+G(d)	0.989	0.1048	6.80e-02	9.695	5.37e-03	-263.989051	0.077025	-263.912026
6-31++G(d,p)	0.989	0.1040	6.68e-02	9.548	5.15e-03	-263.994927	0.076798	-263.918129
6-311+G(d)	0.991	0.1059	6.97e-02	9.844	7.62e-03	-264.036360	0.076593	-263.959767
6-311++G(d,p)	0.991	0.1049	6.82e-02	9.673	7.15e-03	-264.042926	0.076344	-263.966582
Def2SVP	0.991	0.1042	6.74e-02	9.679	5.21e-03	-263.784824	0.076974	-263.707850
Def2SVPD	0.991	0.1054	6.53e-02	9.387	4.92e-03	-263.798420	0.076774	-263.721646
Def2TZVP	0.992	0.1063	6.70e-02	9.553	7.25e-03	-264.073649	0.076619	-263.997030
Def2TZVPD	0.993	0.1061	6.65e-02	9.482	7.10e-03	-264.074772	0.076625	-263.998147
cc-pVDZ	0.990	0.1050	6.78e-02	9.709	6.32e-03	-263.999881	0.076683	-263.923198
aug-cc-pVDZ	0.989	0.1054	6.60e-02	9.433	6.17e-03	-264.014647	0.076532	-263.938115
cc-pVTZ	0.992	0.1060	6.64e-02	9.513	6.98e-03	-264.065901	0.076615	-263.989286
aug-cc-pVTZ	0.992	0.1059	6.58e-02	9.399	6.73e-03	-264.069228	0.076562	-263.992666

Level of Theory: B3LYP

Basis set	HOMA	PDI	MCI	AV1245	FLU	Energy (a.u.)	ZPE (a.u.)	Energy+ZPE (a.u.)
6-31+G(d)	0.973	0.1047	6.91e-02	9.787	6.43e-03	-264.298913	0.076247	-264.222666
6-31++G(d,p)	0.974	0.1039	6.79e-02	9.641	6.17e-03	-264.304957	0.076046	-264.228911
6-311+G(d)	0.981	0.1058	7.06e-02	9.919	9.13e-03	-264.350658	0.075836	-264.274822
6-311++G(d,p)	0.982	0.1048	6.91e-02	9.744	8.59e-03	-264.357210	0.075618	-264.281592
Def2SVP	0.979	0.1041	6.85e-02	9.789	5.89e-03	-264.091312	0.076218	-264.015094
Def2SVPD	0.981	0.1053	6.61e-02	9.453	5.64e-03	-264.107171	0.076159	-264.031012
Def2TZVP	0.986	0.1061	6.80e-02	9.645	8.74e-03	-264.387702	0.076043	-264.311659
Def2TZVPD	0.987	0.1059	6.75e-02	9.568	8.55e-03	-264.388950	0.076048	-264.312902
cc-pVDZ	0.974	0.1050	6.92e-02	9.848	7.36e-03	-264.303977	0.075893	-264.228084
aug-cc-pVDZ	0.973	0.1053	6.72e-02	9.535	7.41e-03	-264.321306	0.075838	-264.245468
cc-pVTZ	0.985	0.1058	6.73e-02	9.592	8.32e-03	-264.380367	0.076043	-264.304324
aug-cc-pVTZ	0.986	0.1055	6.66e-02	9.467	8.01e-03	-264.384047	0.075999	-264.308048

Table 19: Molecule: Pyridazine

Level of Theory: CAM-B3LYP

Basis set	HOMA	PDI	MCI	AV1245	FLU	Energy (a.u.)	ZPE (a.u.)	Energy+ZPE (a.u.)
6-31+G(d)	0.985	0.1039	6.77e-02	9.660	6.14e-03	-264.158803	0.077405	-264.081398
6-31++G(d,p)	0.986	0.1031	6.66e-02	9.520	5.89e-03	-264.164677	0.077185	-264.087492
6-311+G(d)	0.985	0.1050	6.94e-02	9.809	9.03e-03	-264.211453	0.076987	-264.134466
6-311++G(d,p)	0.986	0.1041	6.80e-02	9.647	8.48e-03	-264.217754	0.076750	-264.141004
Def2SVP	0.987	0.1035	6.73e-02	9.688	5.73e-03	-263.951673	0.077297	-263.874376
Def2SVPD	0.988	0.1045	6.50e-02	9.370	5.56e-03	-263.968036	0.077202	-263.890834
Def2TZVP	0.989	0.1053	6.69e-02	9.552	8.62e-03	-264.249110	0.077130	-264.171980
Def2TZVPD	0.990	0.1051	6.65e-02	9.490	8.42e-03	-264.250358	0.077112	-264.173246
cc-pVDZ	0.987	0.1042	6.78e-02	9.725	6.81e-03	-264.163347	0.077004	-264.086343
aug-cc-pVDZ	0.986	0.1045	6.60e-02	9.438	6.98e-03	-264.181104	0.076940	-264.104164
cc-pVTZ	0.989	0.1050	6.63e-02	9.508	8.25e-03	-264.241626	0.077126	-264.164500
aug-cc-pVTZ	0.989	0.1047	6.56e-02	9.394	7.96e-03	-264.245394	0.077066	-264.168328

Level of Theory: wB97XD

6-31+G(d)	0.985	0.1040	6.78e-02	9.677	5.78e-03	-264.201434	0.077294	-264.124140
6-31++G(d,p)	0.985	0.1032	6.66e-02	9.537	5.55e-03	-264.207019	0.077082	-264.129937
6-311+G(d)	0.986	0.1051	6.95e-02	9.834	8.39e-03	-264.250209	0.076807	-264.173402
6-311++G(d,p)	0.987	0.1042	6.81e-02	9.673	7.90e-03	-264.256407	0.076604	-264.179803
Def2SVP	0.987	0.1035	6.71e-02	9.657	5.57e-03	-263.996528	0.077186	-263.919342
Def2SVPD	0.988	0.1046	6.51e-02	9.393	5.36e-03	-264.009859	0.077011	-263.932848
Def2TZVP	0.988	0.1053	6.71e-02	9.580	8.04e-03	-264.287160	0.076970	-264.210190
Def2TZVPD	0.989	0.1051	6.68e-02	9.529	7.88e-03	-264.288213	0.076949	-264.211264
cc-pVDZ	0.985	0.1042	6.76e-02	9.697	6.71e-03	-264.210810	0.076890	-264.133920
aug-cc-pVDZ	0.985	0.1045	6.61e-02	9.454	6.68e-03	-264.225121	0.076771	-264.148350
cc-pVTZ	0.988	0.1050	6.65e-02	9.529	7.73e-03	-264.279217	0.076965	-264.202252
aug-cc-pVTZ	0.988	0.1048	6.59e-02	9.437	7.47e-03	-264.282553	0.076892	-264.205661

Table 20: Molecule: Pyridazine

Level of Theory: M06-2X

Basis set	HOMA	PDI	MCI	AV1245	FLU	Energy (a.u.)	ZPE (a.u.)	Energy+ZPE (a.u.)
6-31+G(d)	0.985	0.1039	6.73e-02	9.678	5.67e-03	-264.180738	0.077222	-264.103516
6-31++G(d,p)	0.985	0.1031	6.62e-02	9.536	5.46e-03	-264.185702	0.076970	-264.108732
6-311+G(d)	0.986	0.1049	6.89e-02	9.816	7.98e-03	-264.236648	0.076918	-264.159730
6-311++G(d,p)	0.986	0.1041	6.76e-02	9.664	7.56e-03	-264.241759	0.076616	-264.165143
Def2SVP	0.986	0.1033	6.65e-02	9.642	5.31e-03	-263.972457	0.077212	-263.895245
Def2SVPD	0.987	0.1047	6.45e-02	9.373	5.06e-03	-263.986269	0.077031	-263.909238
Def2TZVP	0.987	0.1049	6.64e-02	9.544	7.66e-03	-264.272392	0.076963	-264.195429
Def2TZVPD	0.988	0.1049	6.60e-02	9.487	7.51e-03	-264.273395	0.076947	-264.196448
cc-pVDZ	0.986	0.1041	6.72e-02	9.704	6.39e-03	-264.202226	0.076869	-264.125357
aug-cc-pVDZ	0.985	0.1046	6.55e-02	9.455	6.36e-03	-264.217266	0.076780	-264.140486
cc-pVTZ	0.987	0.1047	6.58e-02	9.488	7.39e-03	-264.268161	0.076966	-264.191195
aug-cc-pVTZ	0.987	0.1047	6.52e-02	9.401	7.27e-03	-264.271514	0.076841	-264.194673

Level of Theory: SOGGA11X

Basis set	HOMA	PDI	MCI	AV1245	FLU	Energy (a.u.)	ZPE (a.u.)	Energy+ZPE (a.u.)
6-31+G(d)	0.984	0.1029	6.49e-02	9.370	4.27e-03	-264.215216	0.077953	-264.137263
6-31++G(d,p)	0.984	0.1019	6.35e-02	9.200	4.16e-03	-264.221873	0.077688	-264.144185
6-311+G(d)	0.987	0.1039	6.68e-02	9.546	5.82e-03	-264.263120	0.077459	-264.185661
6-311++G(d,p)	0.988	0.1029	6.53e-02	9.373	5.47e-03	-264.269612	0.077188	-264.192424
Def2SVP	0.985	0.1023	6.45e-02	9.373	4.41e-03	-264.005163	0.077897	-263.927266
Def2SVPD	0.986	0.1033	6.24e-02	9.092	4.09e-03	-264.017043	0.077629	-263.939414
Def2TZVP	0.990	0.1039	6.39e-02	9.235	5.39e-03	-264.299585	0.077597	-264.221988
Def2TZVPD	0.990	0.1037	6.35e-02	9.172	5.28e-03	-264.300569	0.077607	-264.222962
cc-pVDZ	0.985	0.1030	6.48e-02	9.390	5.17e-03	-264.230550	0.077607	-264.152943
aug-cc-pVDZ	0.985	0.1032	6.31e-02	9.121	4.77e-03	-264.243598	0.077400	-264.166198
cc-pVTZ	0.989	0.1036	6.32e-02	9.174	5.36e-03	-264.289415	0.077564	-264.211851
aug-cc-pVTZ	0.989	0.1035	6.26e-02	9.072	5.15e-03	-264.292967	0.077445	-264.215522

Table 21: Molecule: Pyridazine

Level of Theory: M11

Basis set	HOMA	PDI	MCI	AV1245	FLU	Energy (a.u.)	ZPE (a.u.)	Energy+ZPE (a.u.)
6-31+G(d)	0.985	0.1040	6.80e-02	9.731	6.49e-03	-264.141422	0.076811	-264.064611
6-31++G(d,p)	0.985	0.1033	6.70e-02	9.613	6.26e-03	-264.146164	0.076567	-264.069597
6-311+G(d)	0.985	0.1052	6.99e-02	9.924	9.67e-03	-264.197422	0.076308	-264.121114
6-311++G(d,p)	0.986	0.1044	6.87e-02	9.789	9.24e-03	-264.202466	0.076092	-264.126374
Def2SVP	0.985	0.1035	6.74e-02	9.740	5.90e-03	-263.925521	0.076687	-263.848834
Def2SVPD	0.985	0.1047	6.57e-02	9.512	5.92e-03	-263.939764	0.076421	-263.863343
Def2TZVP	0.989	0.1054	6.81e-02	9.739	9.48e-03	-264.240987	0.076749	-264.164238
Def2TZVPD	0.990	0.1053	6.77e-02	9.683	9.31e-03	-264.243110	0.076661	-264.166449
cc-pVDZ	0.985	0.1042	6.78e-02	9.781	6.85e-03	-264.150399	0.076364	-264.074035
aug-cc-pVDZ	0.985	0.1047	6.66e-02	9.576	7.36e-03	-264.166011	0.076270	-264.089741
cc-pVTZ	0.990	0.1050	6.72e-02	9.659	8.99e-03	-264.239150	0.076889	-264.162261
aug-cc-pVTZ	0.989	0.1048	6.68e-02	9.590	9.02e-03	-264.244818	0.076510	-264.168308

Level of Theory: MN15

6-31+G(d)	0.980	0.1050	6.86e-02	9.781	6.09e-03	-263.979809	0.076844	-263.902965
6-31++G(d,p)	0.981	0.1043	6.75e-02	9.655	5.86e-03	-263.984454	0.076612	-263.907842
6-311+G(d)	0.986	0.1062	7.00e-02	9.906	8.72e-03	-264.023671	0.076487	-263.947184
6-311++G(d,p)	0.987	0.1053	6.87e-02	9.759	8.21e-03	-264.028957	0.076265	-263.952692
Def2SVP	0.984	0.1045	6.79e-02	9.774	5.62e-03	-263.739481	0.077025	-263.662456
Def2SVPD	0.985	0.1063	6.56e-02	9.469	5.45e-03	-263.757440	0.076921	-263.680519
Def2TZVP	0.988	0.1069	6.77e-02	9.658	8.75e-03	-264.062223	0.076701	-263.985522
Def2TZVPD	0.989	0.1069	6.73e-02	9.611	8.56e-03	-264.063729	0.076560	-263.987169
cc-pVDZ	0.982	0.1054	6.83e-02	9.806	6.80e-03	-263.983892	0.076611	-263.907281
aug-cc-pVDZ	0.982	0.1063	6.65e-02	9.532	6.95e-03	-264.002947	0.076496	-263.926451
cc-pVTZ	0.988	0.1067	6.70e-02	9.616	8.25e-03	-264.054563	0.076645	-263.977918
aug-cc-pVTZ	0.988	0.1069	6.64e-02	9.520	8.00e-03	-264.060329	0.076569	-263.983760

A.3.2 CCSD

Table 22: Molecule: Pyridazine

Basis set	HOMA	PDI	MCI	AV1245	FLU	Energy (a.u.)	ZPE (a.u.)	Energy+ZPE (a.u.)
6-31+G(d)	0.952	0.0596	4.35e-02	6.580	3.02e-02	-263.512723	0.076120	-263.436603
6-31++G(d,p)	0.954	0.0589	4.25e-02	6.456	3.18e-02	-263.544402	0.076098	-263.468304
6-311+G(d)	0.957	0.0603	4.44e-02	6.697	2.96e-02	-263.590645	0.075440	-263.515205
6-311++G(d,p)	0.957	0.0596	4.35e-02	6.580	3.11e-02	-263.619667	0.074909	-263.544758
Def2SVP	0.962	0.0589	4.32e-02	6.588	3.10e-02	-263.309236	0.076940	-263.232296
Def2SVPD	0.955	0.0597	4.13e-02	6.339	3.14e-02	-263.352850	0.076363	-263.276487
Def2TZVP	0.974	0.0612	4.25e-02	6.507	2.88e-02	-263.764342	0.076678	-263.687664
Def2TZVPD	0.975	0.0611	4.23e-02	6.466	2.89e-02	-263.769185	0.076587	-263.692598
cc-pVDZ	0.932	0.0593	4.34e-02	6.617	3.12e-02	-263.536240	0.076381	-263.459859
aug-cc-pVDZ	0.923	0.0596	4.17e-02	6.370	3.15e-02	-263.578141	0.075888	-263.502253
cc-pVTZ	0.974	0.0610	4.23e-02	6.492	2.94e-02	-263.768528	0.076911	-263.691617
aug-cc-pVTZ	0.975	0.0611	4.17e-02	6.402	2.95e-02	-	-	-

A.3.3 CASSCF(8,8)

Table 23: Molecule: Pyridazine

Basis set	HOMA	PDI	MCI	AV1245	FLU	Energy (a.u.)	ZPE (a.u.)	Energy+ZPE (a.u.)
6-31+G(d)	0.808	0.0879	4.53e-02	7.150	2.52e-02	-262.772173	0.080729	-262.691444
6-311+G(d)	0.792	0.0879	4.55e-02	7.141	3.00e-02	-262.819832	0.080200	-262.739632
Def2SVP	0.822	0.0876	4.54e-02	7.193	2.42e-02	-262.561862	0.080535	-262.481327
Def2TZVP	0.805	0.0880	4.39e-02	6.944	2.74e-02	-262.860026	0.080074	-262.779952
cc-pVDZ	0.809	0.0876	4.47e-02	7.083	2.65e-02	-262.786325	0.080171	-262.706154
cc-pVTZ	0.804	0.0878	4.36e-02	6.926	2.75e-02	-262.852592	0.080069	-262.772523

A.3.4 MP2

Table 24: Molecule: Pyridazine

Basis set	HOMA	PDI	MCI	AV1245	FLU	Energy (a.u.)	ZPE (a.u.)	Energy+ZPE (a.u.)
6-31+G(d)	0.951	0.0597	4.56e-02	6.634	2.64e-02	-263.490960	0.075953	-263.415007
6-31++G(d,p)	0.952	0.0590	4.46e-02	6.512	2.79e-02	-263.522054	0.075400	-263.446654
6-311+G(d)	0.961	0.0602	4.62e-02	6.713	2.60e-02	-263.572043	0.075217	-263.496826
6-311++G(d,p)	0.961	0.0594	4.52e-02	6.597	2.74e-02	-263.600351	0.073843	-263.526508
Def2SVP	0.970	0.0595	4.55e-02	6.706	2.71e-02	-263.282129	0.076793	-263.205336
Def2SVPD	0.961	0.0599	4.29e-02	6.338	2.76e-02	-263.329867	0.076174	-263.253693
Def2TZVP	0.979	0.0604	4.36e-02	6.427	2.59e-02	-263.751883	0.076246	-263.675637
Def2TZVPD	0.978	0.0603	4.32e-02	6.355	2.60e-02	-263.757397	0.076151	-263.681246
cc-pVDZ	0.935	0.0596	4.56e-02	6.710	2.72e-02	-263.510450	0.076279	-263.434171
aug-cc-pVDZ	0.918	0.0595	4.31e-02	6.334	2.78e-02	-263.556218	0.075704	-263.480514
cc-pVTZ	0.978	0.0602	4.33e-02	6.413	2.66e-02	-263.758385	0.076501	-263.681884
aug-cc-pVTZ	0.976	0.0601	4.24e-02	6.271	2.69e-02	-263.777536	0.076090	-263.701446

A.4 Pyrimidine

A.4.1 DFT

Table 25: Molecule: Pyrimidine

Level of Theory: LSDA

Basis set	HOMA	PDI	MCI	AV1245	FLU	Energy (a.u.)	ZPE (a.u.)	Energy+ZPE (a.u.)
6-31+G(d)	1.000	0.1042	6.79e-02	9.854	6.27e-03	-262.860533	0.075527	-262.785006
6-31++G(d,p)	1.000	0.1036	6.70e-02	9.738	6.01e-03	-262.865289	0.075326	-262.789963
6-311+G(d)	1.003	0.1061	6.95e-02	10.06	1.08e-02	-262.914191	0.075145	-262.839046
6-311++G(d,p)	1.003	0.1052	6.82e-02	9.877	1.02e-02	-262.920157	0.074953	-262.845204
Def2SVP	0.998	0.1036	6.66e-02	9.770	5.12e-03	-262.658939	0.075445	-262.583494
Def2SVPD	0.999	0.1056	6.45e-02	9.543	5.50e-03	-262.678876	0.075442	-262.603434
Def2TZVP	1.008	0.1070	6.71e-02	9.808	1.10e-02	-262.951153	0.075165	-262.875988
Def2TZVPD	1.008	0.1069	6.67e-02	9.751	1.08e-02	-262.952574	0.075133	-262.877441
cc-pVDZ	0.999	0.1053	6.80e-02	9.900	7.79e-03	-262.856207	0.075098	-262.781109
aug-cc-pVDZ	1.000	0.1061	6.63e-02	9.684	8.59e-03	-262.873985	0.075095	-262.798890
cc-pVTZ	1.007	0.1065	6.63e-02	9.735	9.92e-03	-262.947196	0.075121	-262.872075
aug-cc-pVTZ	1.007	0.1064	6.57e-02	9.652	9.55e-03	-262.950530	0.075073	-262.875457

Level of Theory: PBE

6-31+G(d)	0.974	0.1031	6.87e-02	9.887	6.62e-03	-264.014383	0.074926	-263.939457
6-31++G(d,p)	0.974	0.1024	6.76e-02	9.748	6.28e-03	-264.019775	0.074753	-263.945022
6-311+G(d)	0.986	0.1047	7.00e-02	10.04	1.04e-02	-264.064572	0.074592	-263.989980
6-311++G(d,p)	0.986	0.1038	6.86e-02	9.865	9.75e-03	-264.070681	0.074412	-263.996269
Def2SVP	0.975	0.1025	6.73e-02	9.803	5.63e-03	-263.810184	0.074812	-263.735372
Def2SVPD	0.979	0.1039	6.51e-02	9.549	5.63e-03	-263.826816	0.074752	-263.752064
Def2TZVP	0.994	0.1052	6.74e-02	9.778	1.03e-02	-264.098641	0.074560	-264.024081
Def2TZVPD	0.993	0.1050	6.69e-02	9.711	1.01e-02	-264.100044	0.074550	-264.025494
cc-pVDZ	0.973	0.1040	6.86e-02	9.922	8.64e-03	-264.020174	0.074547	-263.945627
aug-cc-pVDZ	0.973	0.1042	6.68e-02	9.676	8.79e-03	-264.037614	0.074513	-263.963101
cc-pVTZ	0.994	0.1048	6.68e-02	9.723	9.44e-03	-264.090514	0.074529	-264.015985
aug-cc-pVTZ	0.993	0.1045	6.60e-02	9.617	8.94e-03	-264.094714	0.074503	-264.020211

Table 26: Molecule: Pyrimidine

Level of Theory: PBE0

Basis set	HOMA	PDI	MCI	AV1245	FLU	Energy (a.u.)	ZPE (a.u.)	Energy+ZPE (a.u.)
6-31+G(d)	0.999	0.0995	6.29e-02	9.260	2.67e-03	-264.025395	0.077916	-263.947479
6-31++G(d,p)	0.999	0.0987	6.18e-02	9.115	2.53e-03	-264.031000	0.077697	-263.953303
6-311+G(d)	1.000	0.1013	6.51e-02	9.489	5.42e-03	-264.073861	0.077540	-263.996321
6-311++G(d,p)	1.000	0.1004	6.37e-02	9.318	4.98e-03	-264.080011	0.077316	-264.002695
Def2SVP	0.997	0.0990	6.20e-02	9.216	2.59e-03	-263.822423	0.077757	-263.744666
Def2SVPD	0.997	0.1003	6.00e-02	8.987	2.56e-03	-263.836647	0.077588	-263.759059
Def2TZVP	1.003	0.1016	6.23e-02	9.219	5.22e-03	-264.109687	0.077427	-264.032260
Def2TZVPD	1.003	0.1014	6.19e-02	9.165	5.12e-03	-264.110735	0.077417	-264.033318
cc-pVDZ	0.999	0.1003	6.30e-02	9.305	3.86e-03	-264.035523	0.077503	-263.958020
aug-cc-pVDZ	0.999	0.1005	6.15e-02	9.096	4.01e-03	-264.050107	0.077397	-263.972710
cc-pVTZ	1.003	0.1012	6.17e-02	9.162	4.78e-03	-264.101761	0.077401	-264.024360
aug-cc-pVTZ	1.003	0.1010	6.10e-02	9.069	4.47e-03	-264.105177	0.077373	-264.027804

Level of Theory: B3LYP

Basis set	HOMA	PDI	MCI	AV1245	FLU	Energy (a.u.)	ZPE (a.u.)	Energy+ZPE (a.u.)
6-31+G(d)	0.994	0.1000	6.49e-02	9.466	3.69e-03	-264.335097	0.077181	-264.257916
6-31++G(d,p)	0.994	0.0992	6.37e-02	9.321	3.47e-03	-264.340870	0.076997	-264.263873
6-311+G(d)	0.999	0.1018	6.68e-02	9.676	7.22e-03	-264.387806	0.076813	-264.310993
6-311++G(d,p)	0.999	0.1009	6.53e-02	9.498	6.68e-03	-264.393934	0.076621	-264.317313
Def2SVP	0.993	0.0996	6.38e-02	9.413	3.12e-03	-264.128601	0.077022	-264.051579
Def2SVPD	0.995	0.1007	6.16e-02	9.154	3.25e-03	-264.145199	0.076973	-264.068226
Def2TZVP	1.000	0.1020	6.42e-02	9.417	7.13e-03	-264.423598	0.076882	-264.346716
Def2TZVPD	1.000	0.1018	6.37e-02	9.357	6.99e-03	-264.424783	0.076874	-264.347909
cc-pVDZ	0.993	0.1010	6.51e-02	9.541	5.07e-03	-264.339078	0.076736	-264.262342
aug-cc-pVDZ	0.993	0.1011	6.35e-02	9.305	5.54e-03	-264.356552	0.076715	-264.279837
cc-pVTZ	1.000	0.1016	6.34e-02	9.347	6.36e-03	-264.416096	0.076863	-264.339233
aug-cc-pVTZ	1.000	0.1013	6.27e-02	9.245	5.99e-03	-264.419871	0.076836	-264.343035

Table 27: Molecule: Pyrimidine

Level of Theory: CAM-B3LYP

Basis set	HOMA	PDI	MCI	AV1245	FLU	Energy (a.u.)	ZPE (a.u.)	Energy+ZPE (a.u.)
6-31+G(d)	1.000	0.0989	6.33e-02	9.274	2.91e-03	-264.196152	0.078274	-264.117878
6-31++G(d,p)	1.000	0.0981	6.22e-02	9.135	2.75e-03	-264.201767	0.078066	-264.123701
6-311+G(d)	1.002	0.1007	6.54e-02	9.508	6.32e-03	-264.249832	0.077888	-264.171944
6-311++G(d,p)	1.002	0.0998	6.40e-02	9.343	5.83e-03	-264.255737	0.077672	-264.178065
Def2SVP	0.998	0.0986	6.24e-02	9.257	2.56e-03	-263.990083	0.078056	-263.912027
Def2SVPD	0.999	0.0996	6.03e-02	9.005	2.69e-03	-264.007226	0.077976	-263.929250
Def2TZVP	1.007	0.1008	6.29e-02	9.264	6.25e-03	-264.286189	0.077900	-264.208289
Def2TZVPD	1.007	0.1006	6.25e-02	9.216	6.12e-03	-264.287390	0.077882	-264.209508
cc-pVDZ	1.000	0.0998	6.35e-02	9.359	3.92e-03	-264.199691	0.077790	-264.121901
aug-cc-pVDZ	1.000	0.0998	6.21e-02	9.142	4.45e-03	-264.217597	0.077759	-264.139838
cc-pVTZ	1.007	0.1004	6.22e-02	9.199	5.58e-03	-264.278565	0.077884	-264.200681
aug-cc-pVTZ	1.007	0.1001	6.15e-02	9.106	5.26e-03	-264.282408	0.077848	-264.204560

Level of Theory: wB97XD

6-31+G(d)	0.999	0.0988	6.30e-02	9.250	2.60e-03	-264.238514	0.078157	-264.160357
6-31++G(d,p)	0.999	0.0980	6.19e-02	9.111	2.46e-03	-264.243842	0.077944	-264.165898
6-311+G(d)	1.000	0.1006	6.52e-02	9.490	5.60e-03	-264.288354	0.077728	-264.210626
6-311++G(d,p)	1.000	0.0997	6.38e-02	9.326	5.15e-03	-264.294146	0.077530	-264.216616
Def2SVP	0.998	0.0984	6.20e-02	9.194	2.46e-03	-264.034899	0.077947	-263.956952
Def2SVPD	0.998	0.0994	6.02e-02	8.988	2.48e-03	-264.048906	0.077804	-263.971102
Def2TZVP	1.003	0.1006	6.27e-02	9.250	5.52e-03	-264.323898	0.077742	-264.246156
Def2TZVPD	1.003	0.1004	6.25e-02	9.211	5.41e-03	-264.324910	0.077728	-264.247182
cc-pVDZ	0.999	0.0995	6.31e-02	9.296	3.76e-03	-264.247150	0.077672	-264.169478
aug-cc-pVDZ	0.999	0.0996	6.18e-02	9.114	4.04e-03	-264.261455	0.077602	-264.183853
cc-pVTZ	1.003	0.1002	6.20e-02	9.184	4.98e-03	-264.315787	0.077728	-264.238059
aug-cc-pVTZ	1.003	0.1000	6.15e-02	9.109	4.67e-03	-264.319210	0.077699	-264.241511

Table 28: Molecule: Pyrimidine

Level of Theory: M06-2X

Basis set	HOMA	PDI	MCI	AV1245	FLU	Energy (a.u.)	ZPE (a.u.)	Energy+ZPE (a.u.)
6-31+G(d)	0.999	0.0988	6.26e-02	9.253	2.52e-03	-264.218719	0.078130	-264.140589
6-31++G(d,p)	0.999	0.0981	6.16e-02	9.112	2.41e-03	-264.223463	0.077887	-264.145576
6-311+G(d)	1.000	0.1005	6.47e-02	9.467	4.99e-03	-264.275985	0.077885	-264.198100
6-311++G(d,p)	1.000	0.0997	6.34e-02	9.310	4.63e-03	-264.280775	0.077615	-264.203160
Def2SVP	0.997	0.0984	6.15e-02	9.188	2.34e-03	-264.011663	0.078013	-263.933650
Def2SVPD	0.997	0.0996	5.96e-02	8.976	2.36e-03	-264.025989	0.077855	-263.948134
Def2TZVP	1.002	0.1003	6.22e-02	9.217	4.88e-03	-264.310270	0.077784	-264.232486
Def2TZVPD	1.002	0.1003	6.18e-02	9.173	4.79e-03	-264.311228	0.077768	-264.233460
cc-pVDZ	0.999	0.0996	6.28e-02	9.308	3.47e-03	-264.239642	0.077729	-264.161913
aug-cc-pVDZ	0.999	0.0999	6.14e-02	9.123	3.74e-03	-264.254337	0.077669	-264.176668
cc-pVTZ	1.002	0.1000	6.14e-02	9.137	4.45e-03	-264.306106	0.077781	-264.228325
aug-cc-pVTZ	1.002	0.0999	6.09e-02	9.070	4.30e-03	-264.309536	0.077736	-264.231800

Level of Theory: SOGGA11X

Basis set	HOMA	PDI	MCI	AV1245	FLU	Energy (a.u.)	ZPE (a.u.)	Energy+ZPE (a.u.)
6-31+G(d)	0.997	0.0965	5.89e-02	8.775	1.65e-03	-264.252449	0.078822	-264.173627
6-31++G(d,p)	0.997	0.0955	5.76e-02	8.610	1.74e-03	-264.258847	0.078555	-264.180292
6-311+G(d)	0.999	0.0983	6.13e-02	9.030	2.74e-03	-264.301154	0.078399	-264.222755
6-311++G(d,p)	0.999	0.0973	5.99e-02	8.856	2.54e-03	-264.307235	0.078150	-264.229085
Def2SVP	0.995	0.0962	5.84e-02	8.776	1.89e-03	-264.043777	0.078713	-263.965064
Def2SVPD	0.996	0.0971	5.64e-02	8.538	1.86e-03	-264.056015	0.078435	-263.977580
Def2TZVP	1.001	0.0980	5.83e-02	8.741	2.55e-03	-264.336379	0.078403	-264.257976
Def2TZVPD	1.001	0.0979	5.79e-02	8.690	2.50e-03	-264.337306	0.078397	-264.258909
cc-pVDZ	0.997	0.0973	5.93e-02	8.846	2.34e-03	-264.267122	0.078448	-264.188674
aug-cc-pVDZ	0.996	0.0972	5.77e-02	8.623	2.26e-03	-264.279807	0.078261	-264.201546
cc-pVTZ	1.000	0.0977	5.75e-02	8.664	2.44e-03	-264.326171	0.078344	-264.247827
aug-cc-pVTZ	1.000	0.0975	5.69e-02	8.577	2.29e-03	-264.329883	0.078294	-264.251589

Table 29: Molecule: Pyrimidine

Level of Theory: M11

Basis set	HOMA	PDI	MCI	AV1245	FLU	Energy (a.u.)	ZPE (a.u.)	Energy+ZPE (a.u.)
6-31+G(d)	0.999	0.0990	6.35e-02	9.325	3.25e-03	-264.179130	0.077650	-264.101480
6-31++G(d,p)	0.999	0.0983	6.26e-02	9.207	3.08e-03	-264.183720	0.077427	-264.106293
6-311+G(d)	1.001	0.1010	6.61e-02	9.611	7.27e-03	-264.236162	0.077180	-264.158982
6-311++G(d,p)	1.001	0.1002	6.49e-02	9.470	6.85e-03	-264.240940	0.076991	-264.163949
Def2SVP	0.997	0.0986	6.25e-02	9.291	2.65e-03	-263.963852	0.077452	-263.886400
Def2SVPD	0.997	0.0998	6.10e-02	9.124	2.87e-03	-263.979407	0.077325	-263.902082
Def2TZVP	1.005	0.1011	6.42e-02	9.431	7.59e-03	-264.278055	0.077441	-264.200614
Def2TZVPD	1.005	0.1009	6.38e-02	9.385	7.35e-03	-264.280158	0.077404	-264.202754
cc-pVDZ	0.999	0.0997	6.36e-02	9.385	3.94e-03	-264.187174	0.077189	-264.109985
aug-cc-pVDZ	0.999	0.1000	6.26e-02	9.242	4.67e-03	-264.203305	0.077112	-264.126193
cc-pVTZ	1.005	0.1006	6.33e-02	9.345	6.80e-03	-264.275760	0.077587	-264.198173
aug-cc-pVTZ	1.006	0.1005	6.29e-02	9.297	6.60e-03	-264.281498	0.077458	-264.204040

Level of Theory: MN15

Basis set	HOMA	PDI	MCI	AV1245	FLU	Energy (a.u.)	ZPE (a.u.)	Energy+ZPE (a.u.)
6-31+G(d)	0.997	0.1002	6.41e-02	9.398	3.20e-03	-264.017536	0.077722	-263.939814
6-31++G(d,p)	0.997	0.0996	6.31e-02	9.274	3.05e-03	-264.021887	0.077512	-263.944375
6-311+G(d)	1.000	0.1020	6.59e-02	9.595	6.42e-03	-264.062545	0.077384	-263.985161
6-311++G(d,p)	1.000	0.1012	6.46e-02	9.447	5.98e-03	-264.067452	0.077203	-263.990249
Def2SVP	0.996	0.0998	6.30e-02	9.346	2.77e-03	-263.778407	0.077780	-263.700627
Def2SVPD	0.997	0.1015	6.09e-02	9.107	2.95e-03	-263.797165	0.077735	-263.719430
Def2TZVP	1.001	0.1027	6.37e-02	9.383	6.86e-03	-264.099611	0.077434	-264.022177
Def2TZVPD	1.001	0.1027	6.34e-02	9.347	6.76e-03	-264.101025	0.077352	-264.023673
cc-pVDZ	0.997	0.1011	6.41e-02	9.437	4.30e-03	-264.020899	0.077422	-263.943477
aug-cc-pVDZ	0.997	0.1018	6.25e-02	9.230	4.88e-03	-264.039828	0.077360	-263.962468
cc-pVTZ	1.001	0.1024	6.29e-02	9.320	5.97e-03	-264.091791	0.077397	-264.014394
aug-cc-pVTZ	1.001	0.1024	6.23e-02	9.245	5.70e-03	-264.097587	0.077346	-264.020241

A.4.2 CCSD

Table 30: Molecule: Pyrimidine

Basis set	HOMA	PDI	MCI	AV1245	FLU	Energy (a.u.)	ZPE (a.u.)	Energy+ZPE (a.u.)
6-31+G(d)	0.992	0.0585	4.19e-02	6.558	2.69e-02	-263.550122	0.077419	-263.472703
6-31++G(d,p)	0.992	0.0578	4.10e-02	6.421	2.90e-02	-263.581583	0.077619	-263.503964
6-311+G(d)	0.992	0.0595	4.29e-02	6.681	2.42e-02	-263.628527	0.076863	-263.551664
6-311++G(d,p)	0.992	0.0588	4.20e-02	6.548	2.60e-02	-263.657524	0.076718	-263.580806
Def2SVP	0.992	0.0578	4.13e-02	6.527	2.86e-02	-263.347982	0.078029	-263.269953
Def2SVPD	0.989	0.0587	3.94e-02	6.302	2.96e-02	-263.392828	0.077696	-263.315132
Def2TZVP	1.000	0.0600	4.06e-02	6.450	2.51e-02	-263.801875	0.077698	-263.724177
Def2TZVPD	1.000	0.0599	4.04e-02	6.413	2.53e-02	-263.806607	0.077581	-263.729026
cc-pVDZ	0.976	0.0585	4.21e-02	6.590	2.55e-02	-263.573049	0.077458	-263.495591
aug-cc-pVDZ	0.970	0.0586	4.04e-02	6.366	2.63e-02	-263.614704	0.076991	-263.537713
cc-pVTZ	1.000	0.0597	4.03e-02	6.415	2.62e-02	-263.805947	0.077873	-263.728074
aug-cc-pVTZ	1.000	0.0597	3.97e-02	6.331	2.68e-02	-263.822717	0.077693	-263.745024

A.4.3 CASSCF(8,8)

Table 31: Molecule: Pyrimidine

Basis set	HOMA	PDI	MCI	AV1245	FLU	Energy (a.u.)	ZPE (a.u.)	Energy+ZPE (a.u.)
6-31+G(d)	0.987	0.0942	5.66e-02	8.429	7.80e-04	-262.808880	0.081356	-262.727524
6-311+G(d)	0.987	0.0957	5.87e-02	8.653	4.28e-04	-262.856996	0.080838	-262.776158
Def2SVP	1.000	0.0936	5.52e-02	8.346	1.33e-03	-262.599830	0.081354	-262.518476
Def2TZVP	1.002	0.0947	5.46e-02	8.219	6.34e-04	-262.896124	0.080934	-262.815190
cc-pVDZ	0.997	0.0931	5.49e-02	8.234	1.35e-03	-262.799905	0.081366	-262.718539
cc-pVTZ	1.001	0.0934	5.31e-02	8.036	1.34e-03	-262.866078	0.088727	-262.777351

A.4.4 MP2

Table 32: Molecule: Pyrimidine

Basis set	HOMA	PDI	MCI	AV1245	FLU	Energy (a.u.)	ZPE (a.u.)	Energy+ZPE (a.u.)
6-31+G(d)	0.988	0.0630	4.58e-02	7.148	2.23e-02	-263.526462	0.077349	-263.449113
6-31++G(d,p)	0.989	0.0623	4.48e-02	7.007	2.41e-02	-263.557230	0.077234	-263.479996
6-311+G(d)	0.991	0.0642	4.69e-02	7.286	1.97e-02	-263.608081	0.076772	-263.531309
6-311++G(d,p)	0.991	0.0635	4.59e-02	7.148	2.13e-02	-263.636259	0.076299	-263.559960
Def2SVP	0.992	0.0623	4.52e-02	7.114	2.40e-02	-263.319204	0.077923	-263.241281
Def2SVPD	0.988	0.0632	4.28e-02	6.831	2.50e-02	-263.368184	0.077586	-263.290598
Def2TZVP	0.999	0.0640	4.38e-02	6.952	2.09e-02	-263.787082	0.077287	-263.709795
Def2TZVPD	0.999	0.0639	4.35e-02	6.895	2.12e-02	-263.792447	0.077160	-263.715287
cc-pVDZ	0.974	0.0634	4.61e-02	7.201	2.14e-02	-263.545240	0.077378	-263.467862
aug-cc-pVDZ	0.964	0.0633	4.39e-02	6.914	2.22e-02	-263.590551	0.076828	-263.513723
cc-pVTZ	0.999	0.0638	4.35e-02	6.922	2.19e-02	-263.793436	0.077475	-263.715961
aug-cc-pVTZ	0.999	0.0636	4.27e-02	6.807	2.26e-02	-263.812582	0.077241	-263.735341

A.5 Pyrazine

A.5.1 DFT

Table 33: Molecule: Pyrazine

Level of Theory: LSDA

Basis set	HOMA	PDI	MCI	AV1245	FLU	Energy (a.u.)	ZPE (a.u.)	Energy+ZPE (a.u.)
6-31+G(d)	0.998	0.1109	6.73e-02	11.47	1.18e-02	-262.854290	0.075263	-262.779027
6-31++G(d,p)	0.998	0.1102	6.64e-02	11.32	1.15e-02	-262.859087	0.075075	-262.784012
6-311+G(d)	1.001	0.1124	6.91e-02	11.58	1.70e-02	-262.907897	0.074924	-262.832973
6-311++G(d,p)	1.001	0.1114	6.78e-02	11.37	1.62e-02	-262.913898	0.074754	-262.839144
Def2SVP	0.993	0.1100	6.59e-02	11.29	1.05e-02	-262.653393	0.075271	-262.578122
Def2SVPD	0.993	0.1125	6.36e-02	11.09	1.12e-02	-262.673069	0.075316	-262.597753
Def2TZVP	1.005	0.1135	6.65e-02	11.28	1.75e-02	-262.944993	0.074966	-262.870027
Def2TZVPD	1.005	0.1134	6.61e-02	11.22	1.73e-02	-262.946480	0.074925	-262.871555
cc-pVDZ	0.997	0.1114	6.72e-02	11.36	1.32e-02	-262.850042	0.074830	-262.775212
aug-cc-pVDZ	0.997	0.1126	6.54e-02	11.18	1.45e-02	-262.868203	0.074944	-262.793259
cc-pVTZ	1.005	0.1131	6.58e-02	11.20	1.65e-02	-262.940956	0.074903	-262.866053
aug-cc-pVTZ	1.005	0.1132	6.51e-02	11.14	1.61e-02	-262.944420	0.074858	-262.869562

Level of Theory: PBE

Basis set	HOMA	PDI	MCI	AV1245	FLU	Energy (a.u.)	ZPE (a.u.)	Energy+ZPE (a.u.)
6-31+G(d)	0.966	0.1097	6.81e-02	11.49	1.18e-02	-264.008353	0.074683	-263.933670
6-31++G(d,p)	0.966	0.1088	6.70e-02	11.31	1.13e-02	-264.013788	0.074523	-263.939265
6-311+G(d)	0.981	0.1109	6.97e-02	11.59	1.61e-02	-264.058457	0.074369	-263.984088
6-311++G(d,p)	0.981	0.1098	6.83e-02	11.36	1.53e-02	-264.064600	0.074212	-263.990388
Def2SVP	0.966	0.1086	6.67e-02	11.29	1.07e-02	-263.804738	0.074634	-263.730104
Def2SVPD	0.968	0.1106	6.43e-02	11.08	1.09e-02	-263.821329	0.074667	-263.746662
Def2TZVP	0.990	0.1117	6.69e-02	11.28	1.63e-02	-264.092625	0.074356	-264.018269
Def2TZVPD	0.989	0.1115	6.65e-02	11.21	1.62e-02	-264.094079	0.074338	-264.019741
cc-pVDZ	0.966	0.1099	6.82e-02	11.37	1.38e-02	-264.014187	0.074288	-263.939899
aug-cc-pVDZ	0.965	0.1106	6.61e-02	11.17	1.43e-02	-264.031920	0.074334	-263.957586
cc-pVTZ	0.990	0.1113	6.64e-02	11.21	1.54e-02	-264.084440	0.074315	-264.010125
aug-cc-pVTZ	0.989	0.1112	6.55e-02	11.13	1.50e-02	-264.088718	0.074298	-264.014420

Table 34: Molecule: Pyrazine

Level of Theory: PBE0

Basis set	HOMA	PDI	MCI	AV1245	FLU	Energy (a.u.)	ZPE (a.u.)	Energy+ZPE (a.u.)
6-31+G(d)	0.996	0.1077	6.32e-02	11.13	7.40e-03	-264.018372	0.077679	-263.940693
6-31++G(d,p)	0.996	0.1068	6.20e-02	10.94	7.10e-03	-264.024040	0.077478	-263.946562
6-311+G(d)	0.998	0.1090	6.53e-02	11.28	1.11e-02	-264.066721	0.077321	-263.989400
6-311++G(d,p)	0.998	0.1079	6.39e-02	11.05	1.04e-02	-264.072919	0.077117	-263.995802
Def2SVP	0.992	0.1067	6.21e-02	10.94	7.04e-03	-263.815872	0.077580	-263.738292
Def2SVPD	0.992	0.1086	5.99e-02	10.77	7.10e-03	-263.830145	0.077517	-263.752628
Def2TZVP	1.001	0.1096	6.23e-02	10.96	1.09e-02	-264.102650	0.077234	-264.025416
Def2TZVPD	1.000	0.1095	6.20e-02	10.91	1.08e-02	-264.103749	0.077218	-264.026531
cc-pVDZ	0.996	0.1078	6.30e-02	10.99	8.60e-03	-264.028489	0.077258	-263.951231
aug-cc-pVDZ	0.995	0.1085	6.13e-02	10.83	9.01e-03	-264.043354	0.077184	-263.966170
cc-pVTZ	1.001	0.1093	6.19e-02	10.90	1.05e-02	-264.094673	0.077201	-264.017472
aug-cc-pVTZ	1.000	0.1093	6.11e-02	10.84	1.02e-02	-264.098159	0.077171	-264.020988

Level of Theory: B3LYP

Basis set	HOMA	PDI	MCI	AV1245	FLU	Energy (a.u.)	ZPE (a.u.)	Energy+ZPE (a.u.)
6-31+G(d)	0.989	0.1076	6.49e-02	11.24	8.48e-03	-264.328539	0.076932	-264.251607
6-31++G(d,p)	0.989	0.1067	6.37e-02	11.04	8.13e-03	-264.334354	0.076759	-264.257595
6-311+G(d)	0.996	0.1089	6.69e-02	11.37	1.28e-02	-264.381171	0.076592	-264.304579
6-311++G(d,p)	0.996	0.1078	6.54e-02	11.13	1.21e-02	-264.387337	0.076418	-264.310919
Def2SVP	0.986	0.1066	6.37e-02	11.04	7.59e-03	-264.122694	0.076845	-264.045849
Def2SVPD	0.987	0.1083	6.13e-02	10.84	7.90e-03	-264.139141	0.076866	-264.062275
Def2TZVP	0.999	0.1094	6.41e-02	11.05	1.28e-02	-264.417036	0.076686	-264.340350
Def2TZVPD	0.999	0.1093	6.36e-02	11.00	1.27e-02	-264.418265	0.076666	-264.341599
cc-pVDZ	0.988	0.1077	6.49e-02	11.12	9.72e-03	-264.332670	0.076484	-264.256186
aug-cc-pVDZ	0.988	0.1083	6.31e-02	10.94	1.06e-02	-264.350341	0.076536	-264.273805
cc-pVTZ	0.999	0.1090	6.34e-02	10.97	1.21e-02	-264.409470	0.076655	-264.332815
aug-cc-pVTZ	0.999	0.1089	6.27e-02	10.90	1.17e-02	-264.413324	0.076631	-264.336693

Table 35: Molecule: Pyrazine

Level of Theory: CAM-B3LYP

Basis set	HOMA	PDI	MCI	AV1245	FLU	Energy (a.u.)	ZPE (a.u.)	Energy+ZPE (a.u.)
6-31+G(d)	0.998	0.1071	6.38e-02	11.15	7.66e-03	-264.188970	0.078037	-264.110933
6-31++G(d,p)	0.998	0.1062	6.27e-02	10.96	7.34e-03	-264.194625	0.077838	-264.116787
6-311+G(d)	1.000	0.1083	6.60e-02	11.29	1.20e-02	-264.242553	0.077676	-264.164877
6-311++G(d,p)	1.000	0.1073	6.46e-02	11.07	1.14e-02	-264.248497	0.077476	-264.171021
Def2SVP	0.994	0.1061	6.29e-02	10.98	6.93e-03	-263.983590	0.077895	-263.905695
Def2SVPD	0.994	0.1078	6.06e-02	10.80	7.24e-03	-264.000508	0.077883	-263.922625
Def2TZVP	1.004	0.1088	6.33e-02	11.00	1.20e-02	-264.278966	0.077714	-264.201252
Def2TZVPD	1.004	0.1086	6.29e-02	10.96	1.19e-02	-264.280207	0.077685	-264.202522
cc-pVDZ	0.998	0.1071	6.38e-02	11.03	8.56e-03	-264.192707	0.077555	-264.115152
aug-cc-pVDZ	0.997	0.1077	6.22e-02	10.88	9.44e-03	-264.210744	0.077595	-264.133149
cc-pVTZ	1.004	0.1084	6.27e-02	10.92	1.15e-02	-264.271286	0.077685	-264.193601
aug-cc-pVTZ	1.004	0.1083	6.20e-02	10.87	1.11e-02	-264.275197	0.077652	-264.197545

Level of Theory: wB97XD

6-31+G(d)	0.997	0.1071	6.36e-02	11.16	7.28e-03	-264.231165	0.077944	-264.153221
6-31++G(d,p)	0.997	0.1062	6.24e-02	10.97	6.97e-03	-264.236552	0.077756	-264.158796
6-311+G(d)	0.998	0.1083	6.58e-02	11.30	1.12e-02	-264.280914	0.077511	-264.203403
6-311++G(d,p)	0.999	0.1073	6.44e-02	11.08	1.06e-02	-264.286742	0.077337	-264.209405
Def2SVP	0.993	0.1061	6.24e-02	10.95	6.82e-03	-264.028020	0.077796	-263.950224
Def2SVPD	0.993	0.1078	6.05e-02	10.83	6.95e-03	-264.041955	0.077729	-263.964226
Def2TZVP	1.001	0.1086	6.31e-02	11.01	1.12e-02	-264.316550	0.077563	-264.238987
Def2TZVPD	1.001	0.1085	6.28e-02	10.98	1.11e-02	-264.317606	0.077541	-264.240065
cc-pVDZ	0.996	0.1070	6.34e-02	11.01	8.43e-03	-264.239860	0.077480	-264.162380
aug-cc-pVDZ	0.995	0.1077	6.19e-02	10.90	8.98e-03	-264.254350	0.077441	-264.176909
cc-pVTZ	1.001	0.1083	6.25e-02	10.94	1.07e-02	-264.308394	0.077543	-264.230851
aug-cc-pVTZ	1.001	0.1083	6.20e-02	10.90	1.04e-02	-264.311884	0.077503	-264.234381

Table 36: Molecule: Pyrazine

Level of Theory: M06-2X

Basis set	HOMA	PDI	MCI	AV1245	FLU	Energy (a.u.)	ZPE (a.u.)	Energy+ZPE (a.u.)
6-31+G(d)	0.995	0.1068	6.32e-02	11.06	7.38e-03	-264.211656	0.077824	-264.133832
6-31++G(d,p)	0.995	0.1059	6.20e-02	10.88	7.09e-03	-264.216440	0.077601	-264.138839
6-311+G(d)	0.997	0.1080	6.52e-02	11.21	1.09e-02	-264.268738	0.077603	-264.191135
6-311++G(d,p)	0.997	0.1070	6.39e-02	11.00	1.03e-02	-264.273547	0.077356	-264.196191
Def2SVP	0.991	0.1058	6.19e-02	10.86	6.75e-03	-264.005066	0.077801	-263.927265
Def2SVPD	0.991	0.1076	5.98e-02	10.70	6.89e-03	-264.019443	0.077754	-263.941689
Def2TZVP	0.999	0.1083	6.24e-02	10.93	1.08e-02	-264.303024	0.077526	-264.225498
Def2TZVPD	0.999	0.1082	6.21e-02	10.89	1.07e-02	-264.304030	0.077503	-264.226527
cc-pVDZ	0.995	0.1067	6.31e-02	10.94	8.43e-03	-264.232592	0.077406	-264.155186
aug-cc-pVDZ	0.994	0.1075	6.14e-02	10.79	8.98e-03	-264.247544	0.077402	-264.170142
cc-pVTZ	1.000	0.1080	6.18e-02	10.86	1.04e-02	-264.298675	0.077497	-264.221178
aug-cc-pVTZ	1.000	0.1081	6.13e-02	10.82	1.03e-02	-264.302200	0.077444	-264.224756

Level of Theory: SOGGA11X

Basis set	HOMA	PDI	MCI	AV1245	FLU	Energy (a.u.)	ZPE (a.u.)	Energy+ZPE (a.u.)
6-31+G(d)	0.992	0.1062	6.01e-02	10.89	5.38e-03	-264.244549	0.078645	-264.165904
6-31++G(d,p)	0.992	0.1051	5.87e-02	10.67	5.22e-03	-264.251035	0.078404	-264.172631
6-311+G(d)	0.996	0.1073	6.22e-02	11.06	7.64e-03	-264.293245	0.078211	-264.215034
6-311++G(d,p)	0.996	0.1061	6.07e-02	10.82	7.21e-03	-264.299385	0.077982	-264.221403
Def2SVP	0.989	0.1050	5.92e-02	10.69	5.47e-03	-264.036295	0.078528	-263.957767
Def2SVPD	0.989	0.1067	5.71e-02	10.54	5.36e-03	-264.048648	0.078366	-263.970282
Def2TZVP	0.998	0.1076	5.89e-02	10.72	7.24e-03	-264.328668	0.078264	-264.250404
Def2TZVPD	0.998	0.1074	5.86e-02	10.67	7.16e-03	-264.329652	0.078255	-264.251397
cc-pVDZ	0.993	0.1060	6.00e-02	10.74	6.46e-03	-264.259234	0.078231	-264.181003
aug-cc-pVDZ	0.991	0.1065	5.82e-02	10.60	6.48e-03	-264.272159	0.078067	-264.194092
cc-pVTZ	0.998	0.1072	5.84e-02	10.64	7.09e-03	-264.318325	0.078204	-264.240121
aug-cc-pVTZ	0.998	0.1072	5.78e-02	10.59	6.85e-03	-264.322118	0.078154	-264.243964

Table 37: Molecule: Pyrazine

Level of Theory: M11

Basis set	HOMA	PDI	MCI	AV1245	FLU	Energy (a.u.)	ZPE (a.u.)	Energy+ZPE (a.u.)
6-31+G(d)	0.995	0.1072	6.41e-02	11.20	8.70e-03	-264.171889	0.077348	-264.094541
6-31++G(d,p)	0.995	0.1064	6.32e-02	11.05	8.38e-03	-264.176532	0.077142	-264.099390
6-311+G(d)	0.997	0.1084	6.67e-02	11.36	1.38e-02	-264.228861	0.076923	-264.151938
6-311++G(d,p)	0.998	0.1075	6.55e-02	11.17	1.32e-02	-264.233632	0.076743	-264.156889
Def2SVP	0.991	0.1062	6.30e-02	11.03	7.54e-03	-263.957297	0.077247	-263.880050
Def2SVPD	0.990	0.1081	6.14e-02	10.95	8.05e-03	-263.972510	0.077180	-263.895330
Def2TZVP	1.001	0.1088	6.47e-02	11.14	1.43e-02	-264.270642	0.077196	-264.193446
Def2TZVPD	1.001	0.1088	6.43e-02	11.11	1.40e-02	-264.272870	0.077170	-264.195700
cc-pVDZ	0.994	0.1071	6.39e-02	11.08	9.30e-03	-264.180026	0.076836	-264.103190
aug-cc-pVDZ	0.994	0.1080	6.28e-02	11.01	1.05e-02	-264.196234	0.076779	-264.119455
cc-pVTZ	1.003	0.1082	6.38e-02	11.01	1.35e-02	-264.268362	0.077334	-264.191028
aug-cc-pVTZ	1.002	0.1082	6.34e-02	10.98	1.34e-02	-264.274214	0.077153	-264.197061

Level of Theory: MN15

Basis set	HOMA	PDI	MCI	AV1245	FLU	Energy (a.u.)	ZPE (a.u.)	Energy+ZPE (a.u.)
6-31+G(d)	0.992	0.1080	6.46e-02	11.19	8.05e-03	-264.010491	0.077457	-263.933034
6-31++G(d,p)	0.992	0.1072	6.36e-02	11.02	7.77e-03	-264.014870	0.077258	-263.937612
6-311+G(d)	0.998	0.1092	6.64e-02	11.30	1.21e-02	-264.055452	0.077157	-263.978295
6-311++G(d,p)	0.998	0.1083	6.51e-02	11.09	1.15e-02	-264.060397	0.076993	-263.983404
Def2SVP	0.990	0.1069	6.34e-02	10.99	7.24e-03	-263.772057	0.077619	-263.694438
Def2SVPD	0.991	0.1093	6.11e-02	10.80	7.60e-03	-263.790483	0.077608	-263.712875
Def2TZVP	0.999	0.1101	6.40e-02	11.01	1.28e-02	-264.092610	0.077228	-264.015382
Def2TZVPD	0.999	0.1102	6.37e-02	10.98	1.27e-02	-264.094072	0.077134	-264.016938
cc-pVDZ	0.992	0.1080	6.43e-02	11.04	9.07e-03	-264.014039	0.077163	-263.936876
aug-cc-pVDZ	0.992	0.1092	6.26e-02	10.88	1.00e-02	-264.033154	0.077208	-263.955946
cc-pVTZ	0.999	0.1099	6.33e-02	10.94	1.20e-02	-264.084751	0.077187	-264.007564
aug-cc-pVTZ	0.999	0.1102	6.27e-02	10.90	1.17e-02	-264.090568	0.077129	-264.013439

A.5.2 CCSD

Table 38: Molecule: Pyrazine

Basis set	HOMA	PDI	MCI	AV1245	FLU	Energy (a.u.)	ZPE (a.u.)	Energy+ZPE (a.u.)
6-31+G(d)	0.986	0.0619	4.17e-02	7.762	2.57e-02	-263.542771	0.077052	-263.465719
6-31++G(d,p)	0.987	0.0611	4.07e-02	7.593	2.77e-02	-263.574308	0.077293	-263.497015
6-311+G(d)	0.986	0.0626	4.28e-02	7.846	2.36e-02	-263.621210	0.076531	-263.544679
6-311++G(d,p)	0.985	0.0618	4.18e-02	7.675	2.53e-02	-263.650313	0.076404	-263.573909
Def2SVP	0.984	0.0610	4.11e-02	7.606	2.70e-02	-263.341077	0.077777	-263.263300
Def2SVPD	0.980	0.0624	3.91e-02	7.461	2.80e-02	-263.385834	0.077439	-263.308395
Def2TZVP	0.998	0.0638	4.05e-02	7.685	2.42e-02	-263.794072	0.077370	-263.716702
Def2TZVPD	0.998	0.0638	4.03e-02	7.651	2.44e-02	-263.798944	0.077257	-263.721687
cc-pVDZ	0.966	0.0614	4.18e-02	7.632	2.53e-02	-263.565635	0.077144	-263.488491
aug-cc-pVDZ	0.959	0.0619	4.00e-02	7.492	2.61e-02	-263.607514	0.076596	-263.530918
cc-pVTZ	0.999	0.0636	4.03e-02	7.640	2.48e-02	-263.798215	0.077574	-263.720641
aug-cc-pVTZ	0.998	0.0638	3.97e-02	7.597	2.54e-02	-263.815060	0.077347	-263.737713

A.5.3 CASSCF(8,8)

Table 39: Molecule: Pyrazine

Basis set	HOMA	PDI	MCI	AV1245	FLU	Energy (a.u.)	ZPE (a.u.)	Energy+ZPE (a.u.)
6-31+G(d)	0.989	0.1031	5.75e-02	10.57	4.50e-03	-262.802208	0.080667	-262.721541
6-311+G(d)	0.998	0.1056	5.93e-02	10.76	3.44e-03	-262.830482	0.077451	-262.753031
Def2SVP	0.987	0.1018	5.65e-02	10.35	4.54e-03	-262.594400	0.080491	-262.513909
Def2TZVP	0.994	0.1038	5.59e-02	10.39	5.22e-03	-262.889053	0.080044	-262.809009
cc-pVDZ	0.997	0.1042	5.69e-02	10.41	3.36e-03	-262.796427	0.080321	-262.716106
cc-pVTZ	1.001	0.1053	5.53e-02	10.36	3.45e-03	-262.862211	0.092585	-262.769626

A.5.4 MP2

Table 40: Molecule: Pyrazine

Basis set	HOMA	PDI	MCI	AV1245	FLU	Energy (a.u.)	ZPE (a.u.)	Energy+ZPE (a.u.)
6-31+G(d)	0.983	0.0618	4.27e-02	7.808	2.21e-02	-263.520672	0.077036	-263.443636
6-31++G(d,p)	0.983	0.0610	4.18e-02	7.636	2.38e-02	-263.551548	0.076970	-263.474578
6-311+G(d)	0.984	0.0622	4.35e-02	7.832	2.02e-02	-263.602562	0.076499	-263.526063
6-311++G(d,p)	0.983	0.0613	4.26e-02	7.656	2.17e-02	-263.630882	0.076048	-263.554834
Def2SVP	0.984	0.0612	4.23e-02	7.696	2.34e-02	-263.313739	0.077736	-263.236003
Def2SVPD	0.978	0.0622	3.96e-02	7.427	2.45e-02	-263.362871	0.077380	-263.285491
Def2TZVP	0.997	0.0626	4.06e-02	7.527	2.11e-02	-263.781231	0.077031	-263.704200
Def2TZVPD	0.997	0.0625	4.02e-02	7.473	2.14e-02	-263.786770	0.076904	-263.709866
cc-pVDZ	0.962	0.0615	4.29e-02	7.675	2.15e-02	-263.539511	0.077132	-263.462379
aug-cc-pVDZ	0.951	0.0614	4.04e-02	7.422	2.24e-02	-263.585306	0.076518	-263.508788
cc-pVTZ	0.997	0.0623	4.03e-02	7.481	2.19e-02	-263.787653	0.077248	-263.710405
aug-cc-pVTZ	0.996	0.0623	3.94e-02	7.396	2.26e-02	-263.806950	0.076962	-263.729988

B Statistical Summary of the Aromaticity Indexes

The mean value, standard deviation (Std), maximum value (Max) and minimum value (Min) observed within each electronic structure method for each aromaticity index and molecule are calculated and reported in the following five tables.

Table 41: Statistical summary of the HOMA index

		CCSD	CASSCF	MP2	LSDA	PBE	PBE0	B3LYP	CAM-B3LYP	wB97XD	M06-2X	SOGGA11X	M11	MN15
Benzene	Mean:	0.965	0.994	0.964	0.999	0.953	0.995	0.984	0.998	0.995	0.994	0.987	0.995	0.989
	Std:	0.031	0.014	0.030	0.004	0.023	0.005	0.012	0.004	0.005	0.005	0.009	0.005	0.009
	Max:	0.996	1.002	0.993	1.005	0.980	1.000	0.998	1.002	1.000	1.000	0.998	1.001	1.000
	Min:	0.902	0.952	0.897	0.995	0.930	0.989	0.970	0.993	0.988	0.988	0.977	0.988	0.979
Pyridine	Mean:	0.980	0.992	0.978	1.001	0.968	0.998	0.991	1.000	0.998	0.997	0.993	0.998	0.994
	Std:	0.019	0.006	0.019	0.004	0.016	0.003	0.007	0.003	0.003	0.003	0.005	0.004	0.005
	Max:	0.999	1.002	0.997	1.006	0.988	1.001	1.000	1.004	1.001	1.000	0.999	1.003	1.000
	Min:	0.941	0.982	0.935	0.996	0.952	0.993	0.983	0.996	0.993	0.993	0.987	0.993	0.989
Pyridazine	Mean:	0.958	0.807	0.960	0.995	0.958	0.991	0.980	0.987	0.987	0.986	0.987	0.987	0.985
	Std:	0.017	0.007	0.019	0.002	0.013	0.001	0.005	0.002	0.001	0.001	0.002	0.002	0.003
	Max:	0.975	0.822	0.979	0.998	0.974	0.993	0.987	0.990	0.989	0.988	0.990	0.990	0.989
	Min:	0.923	0.792	0.918	0.992	0.943	0.989	0.973	0.985	0.985	0.985	0.984	0.985	0.980
Pyrimidine	Mean:	0.991	0.991	0.989	1.003	0.983	1.000	0.997	1.002	1.001	1.000	0.998	1.001	0.999
	Std:	0.009	0.006	0.011	0.004	0.009	0.002	0.003	0.003	0.002	0.002	0.002	0.003	0.002
	Max:	1.000	1.002	0.999	1.008	0.994	1.003	1.000	1.007	1.003	1.002	1.001	1.006	1.001
	Min:	0.970	0.987	0.964	0.998	0.973	0.997	0.993	0.998	0.998	0.997	0.995	0.997	0.996
Pyrazine	Mean:	0.986	0.991	0.983	1.000	0.976	0.997	0.993	1.000	0.998	0.996	0.994	0.997	0.995
	Std:	0.013	0.005	0.014	0.005	0.011	0.003	0.005	0.004	0.003	0.003	0.004	0.004	0.004
	Max:	0.999	1.001	0.997	1.005	0.990	1.001	0.999	1.004	1.001	1.000	0.998	1.003	0.999
	Min:	0.959	0.987	0.951	0.993	0.965	0.992	0.986	0.994	0.993	0.991	0.989	0.990	0.990

Table 42: Statistical summary of the PDI

		CCSD	CASSCF	MP2	LSDA	PBE	PBE0	B3LYP	CAM-B3LYP	wB97XD	M06-2X	SOGGA11X	M11	MN15
Benzene	Mean:	0.0599	0.1007	0.0598	0.1074	0.1052	0.1040	0.1035	0.1034	0.1034	0.1035	0.1022	0.1044	0.1048
	Std:	0.0010	0.0007	0.0006	0.0008	0.0007	0.0008	0.0007	0.0007	0.0006	0.0006	0.0008	0.0006	0.0007
	Max:	0.0615	0.1017	0.0608	0.1086	0.1063	0.1052	0.1048	0.1046	0.1047	0.1047	0.1037	0.1056	0.1058
	Min:	0.0588	0.0991	0.0590	0.1059	0.1039	0.1028	0.1024	0.1023	0.1024	0.1024	0.1011	0.1035	0.1035
Pyridine	Mean:	0.0599	0.0986	0.0609	0.1072	0.1053	0.1033	0.1032	0.1027	0.1026	0.1026	0.1011	0.1033	0.1042
	Std:	0.0009	0.0005	0.0005	0.0010	0.0008	0.0008	0.0008	0.0007	0.0007	0.0007	0.0007	0.0007	0.0009
	Max:	0.0612	0.0994	0.0617	0.1086	0.1065	0.1044	0.1043	0.1037	0.1037	0.1036	0.1023	0.1043	0.1053
	Min:	0.0587	0.0977	0.0601	0.1055	0.1039	0.1018	0.1018	0.1013	0.1014	0.1013	0.0997	0.1020	0.1026
Pyridazine	Mean:	0.0600	0.0878	0.0598	0.1093	0.1077	0.1053	0.1052	0.1044	0.1045	0.1043	0.1032	0.1045	0.1059
	Std:	0.0009	0.0001	0.0004	0.0010	0.0008	0.0008	0.0007	0.0007	0.0006	0.0006	0.0006	0.0007	0.0009
	Max:	0.0612	0.0880	0.0604	0.1104	0.1087	0.1063	0.1061	0.1053	0.1053	0.1049	0.1039	0.1054	0.1069
	Min:	0.0589	0.0876	0.0590	0.1076	0.1063	0.1040	0.1039	0.1031	0.1032	0.1031	0.1019	0.1033	0.1043
Pyrimidine	Mean:	0.0590	0.0941	0.0634	0.1056	0.1040	0.1004	0.1009	0.0998	0.0996	0.0996	0.0972	0.1000	0.1014
	Std:	0.0008	0.0006	0.0006	0.0012	0.0009	0.0009	0.0009	0.0009	0.0008	0.0008	0.0008	0.0009	0.0011
	Max:	0.0600	0.0957	0.0642	0.1070	0.1052	0.1016	0.1020	0.1008	0.1006	0.1005	0.0983	0.1011	0.1027
	Min:	0.0578	0.0931	0.0623	0.1036	0.1024	0.0987	0.0992	0.0981	0.0980	0.0981	0.0955	0.0983	0.0996
Pyrazine	Mean:	0.0624	0.1035	0.0619	0.1121	0.1104	0.1084	0.1082	0.1076	0.1076	0.1073	0.1065	0.1077	0.1089
	Std:	0.0011	0.0010	0.0005	0.0012	0.0010	0.0010	0.0010	0.0009	0.0009	0.0009	0.0009	0.0009	0.0012
	Max:	0.0638	0.1056	0.0626	0.1135	0.1117	0.1096	0.1094	0.1088	0.1086	0.1083	0.1076	0.1088	0.1102
	Min:	0.0610	0.1018	0.0610	0.1100	0.1086	0.1067	0.1066	0.1061	0.1061	0.1058	0.1050	0.1062	0.1069

Table 43: Statistical summary of the MCI

		CCSD	CASSCF	MP2	LSDA	PBE	PBE0	B3LYP	CAM-B3LYP	wB97XD	M06-2X	SOGGA11X	M11	MN15
Benzene	Mean:	4.77e-2	7.07e-2	4.85e-2	7.43e-2	7.36e-2	7.22e-2	7.22e-2	7.22e-2	7.23e-2	7.22e-2	7.08e-2	7.36e-2	7.21e-2
	Std:	1.13e-3	1.79e-3	1.64e-3	1.75e-3	1.71e-3	1.59e-3	1.71e-3	1.61e-3	1.49e-3	1.49e-3	1.56e-3	1.39e-3	1.65e-3
	Max:	5.00e-2	7.20e-2	5.16e-2	7.75e-2	7.69e-2	7.55e-2	7.56e-2	7.54e-2	7.54e-2	7.52e-2	7.40e-2	7.63e-2	7.54e-2
	Min:	4.59e-2	6.80e-2	4.64e-2	7.20e-2	7.15e-2	7.04e-2	7.01e-2	7.02e-2	7.06e-2	7.05e-2	6.89e-2	7.15e-2	7.02e-2
Pyridine	Mean:	4.38e-2	6.12e-2	4.53e-2	6.96e-2	6.96e-2	6.63e-2	6.73e-2	6.67e-2	6.66e-2	6.62e-2	6.36e-2	6.77e-2	6.70e-2
	Std:	1.00e-3	1.59e-3	1.43e-3	1.35e-3	1.39e-3	1.31e-3	1.37e-3	1.27e-3	1.22e-3	1.26e-3	1.33e-3	1.07e-3	1.29e-3
	Max:	4.56e-2	6.22e-2	4.74e-2	7.20e-2	7.22e-2	6.90e-2	6.99e-2	6.92e-2	6.92e-2	6.88e-2	6.65e-2	7.00e-2	6.94e-2
	Min:	4.23e-2	5.81e-2	4.34e-2	6.76e-2	6.77e-2	6.44e-2	6.52e-2	6.48e-2	6.48e-2	6.42e-2	6.19e-2	6.62e-2	6.50e-2
Pyridazine	Mean:	4.27e-2	4.50e-2	4.43e-2	7.08e-2	7.11e-2	6.71e-2	6.81e-2	6.69e-2	6.70e-2	6.64e-2	6.40e-2	6.76e-2	6.76e-2
	Std:	9.07e-4	6.44e-4	1.30e-3	1.27e-3	1.30e-3	1.23e-3	1.25e-3	1.18e-3	1.15e-3	1.18e-3	1.26e-3	1.07e-3	1.17e-3
	Max:	4.44e-2	4.55e-2	4.62e-2	7.31e-2	7.35e-2	6.97e-2	7.06e-2	6.94e-2	6.95e-2	6.89e-2	6.68e-2	6.99e-2	7.00e-2
	Min:	4.13e-2	4.36e-2	4.24e-2	6.87e-2	6.92e-2	6.53e-2	6.61e-2	6.50e-2	6.51e-2	6.45e-2	6.24e-2	6.57e-2	6.56e-2
Pyrimidine	Mean:	4.10e-2	5.61e-2	4.46e-2	6.70e-2	6.75e-2	6.22e-2	6.41e-2	6.27e-2	6.25e-2	6.20e-2	5.84e-2	6.34e-2	6.34e-2
	Std:	1.06e-3	1.42e-3	1.40e-3	1.32e-3	1.33e-3	1.31e-3	1.34e-3	1.28e-3	1.27e-3	1.29e-3	1.34e-3	1.29e-3	1.25e-3
	Max:	4.29e-2	5.87e-2	4.69e-2	6.95e-2	7.00e-2	6.51e-2	6.68e-2	6.54e-2	6.52e-2	6.47e-2	6.13e-2	6.61e-2	6.59e-2
	Min:	3.94e-2	5.31e-2	4.27e-2	6.45e-2	6.51e-2	6.00e-2	6.16e-2	6.03e-2	6.02e-2	5.96e-2	5.64e-2	6.10e-2	6.09e-2
Pyrazine	Mean:	4.08e-2	5.72e-2	4.14e-2	6.63e-2	6.70e-2	6.23e-2	6.40e-2	6.31e-2	6.29e-2	6.23e-2	5.92e-2	6.39e-2	6.37e-2
	Std:	1.06e-3	9.95e-4	1.42e-3	1.41e-3	1.43e-3	1.40e-3	1.42e-3	1.36e-3	1.34e-3	1.37e-3	1.40e-3	1.36e-3	1.34e-3
	Max:	4.28e-2	5.93e-2	4.35e-2	6.91e-2	6.97e-2	6.53e-2	6.69e-2	6.60e-2	6.58e-2	6.52e-2	6.22e-2	6.67e-2	6.64e-2
	Min:	3.91e-2	5.53e-2	3.94e-2	6.36e-2	6.43e-2	5.99e-2	6.13e-2	6.06e-2	6.05e-2	5.98e-2	5.71e-2	6.14e-2	6.11e-2

Table 44: Statistical summary of the AV1245 Index

		CCSD	CASSCF	MP2	LSDA	PBE	PBE0	B3LYP	CAM-B3LYP	wB97XD	M06-2X	SOGGA11X	M11	MN15
Benzene	Mean:	7.44	10.49	7.43	10.89	10.79	10.64	10.63	10.64	10.67	10.63	10.47	10.82	10.64
	Std:	0.15	0.22	0.21	0.23	0.23	0.21	0.23	0.21	0.19	0.19	0.21	0.17	0.21
	Max:	7.70	10.64	7.82	11.28	11.20	11.04	11.04	11.02	11.04	11.00	10.87	11.14	11.04
	Min:	7.18	10.18	7.14	10.58	10.50	10.39	10.33	10.37	10.44	10.40	10.21	10.55	10.37
Pyridine	Mean:	6.99	9.57	7.13	10.35	10.31	9.98	10.06	10.01	10.01	9.98	9.69	10.16	10.06
	Std:	0.12	0.20	0.18	0.16	0.17	0.16	0.17	0.15	0.14	0.15	0.16	0.12	0.15
	Max:	7.22	9.70	7.40	10.62	10.61	10.29	10.37	10.29	10.31	10.27	10.02	10.41	10.33
	Min:	6.81	9.20	6.91	10.14	10.09	9.79	9.85	9.82	9.83	9.78	9.50	10.00	9.85
Pyridazine	Mean:	6.51	7.11	6.50	9.96	9.98	9.58	9.67	9.57	9.58	9.57	9.26	9.69	9.67
	Std:	0.11	0.09	0.17	0.15	0.16	0.14	0.15	0.14	0.13	0.13	0.15	0.11	0.13
	Max:	6.70	7.19	6.71	10.18	10.22	9.84	9.92	9.81	9.83	9.82	9.55	9.92	9.91
	Min:	6.34	6.93	6.27	9.74	9.76	9.39	9.45	9.37	9.39	9.37	9.07	9.51	9.47
Pyrimidine	Mean:	6.47	8.37	7.02	9.78	9.78	9.20	9.40	9.23	9.21	9.19	8.73	9.34	9.34
	Std:	0.11	0.15	0.16	0.13	0.14	0.13	0.14	0.13	0.13	0.13	0.14	0.13	0.12
	Max:	6.68	8.65	7.29	10.04	10.04	9.49	9.68	9.51	9.49	9.47	9.03	9.61	9.60
	Min:	6.30	8.04	6.81	9.54	9.55	8.99	9.15	9.01	8.99	8.98	8.54	9.12	9.11
Pyrazine	Mean:	7.64	10.52	7.59	11.29	11.29	10.96	11.05	10.99	11.00	10.91	10.72	11.09	11.01
	Std:	0.10	0.12	0.15	0.14	0.15	0.14	0.15	0.13	0.13	0.13	0.14	0.11	0.13
	Max:	7.85	10.76	7.83	11.58	11.59	11.28	11.37	11.29	11.30	11.21	11.06	11.36	11.30
	Min:	7.46	10.35	7.40	11.09	11.08	10.77	10.84	10.80	10.83	10.70	10.54	10.95	10.80

Table 45: Statistical summary of the FLU Index

		CCSD	CASSCF	MP2	LSDA	PBE	PBE0	B3LYP	CAM-B3LYP	wB97XD	M06-2X	SOGGA11X	M11	MN15
Benzene	Mean:	2.86e-2	8.21e-5	2.64e-2	8.00e-6	5.27e-5	3.57e-5	5.09e-5	2.13e-5	3.04e-5	3.98e-5	8.18e-5	7.50e-6	1.72e-5
	Std:	1.42e-3	1.39e-4	1.13e-3	1.34e-5	3.53e-5	2.74e-5	3.47e-5	1.75e-5	2.28e-5	2.87e-5	5.05e-5	6.27e-6	1.54e-5
	Max:	3.10e-2	4.16e-4	2.85e-2	3.90e-5	1.29e-4	9.70e-5	1.25e-4	6.50e-5	7.90e-5	1.01e-4	1.71e-4	1.60e-5	5.90e-5
	Min:	2.69e-2	0.00e+0	2.45e-2	0.00e+0	0.00e+0	0.00e+0	0.00e+0	2.00e-6	0.00e+0	0.00e+0	1.00e-6	0.00e+0	4.00e-6
Pyridine	Mean:	2.80e-2	1.16e-3	2.49e-2	6.45e-3	6.32e-3	3.78e-3	4.59e-3	4.11e-3	3.82e-3	3.65e-3	2.39e-3	4.66e-3	4.34e-3
	Std:	1.25e-3	3.47e-4	1.01e-3	1.32e-3	1.07e-3	8.23e-4	1.03e-3	1.04e-3	9.16e-4	8.23e-4	4.30e-4	1.33e-3	1.06e-3
	Max:	2.97e-2	1.98e-3	2.64e-2	7.95e-3	7.46e-3	4.69e-3	5.71e-3	5.24e-3	4.81e-3	4.51e-3	2.91e-3	6.19e-3	5.62e-3
	Min:	2.63e-2	9.18e-4	2.31e-2	4.46e-3	4.70e-3	2.71e-3	3.09e-3	2.68e-3	2.61e-3	2.50e-3	1.75e-3	2.82e-3	2.85e-3
Pyridazine	Mean:	3.04e-2	2.60e-2	2.69e-2	8.81e-3	9.18e-3	6.33e-3	7.52e-3	7.32e-3	6.92e-3	6.63e-3	4.95e-3	7.87e-3	7.27e-3
	Std:	1.09e-3	1.60e-3	7.13e-4	1.35e-3	1.14e-3	9.51e-4	1.22e-3	1.27e-3	1.12e-3	1.05e-3	5.81e-4	1.53e-3	1.28e-3
	Max:	3.18e-2	3.00e-2	2.79e-2	1.05e-2	1.07e-2	7.62e-3	9.13e-3	9.03e-3	8.39e-3	7.98e-3	5.82e-3	9.67e-3	8.75e-3
	Min:	2.88e-2	2.42e-2	2.59e-2	6.73e-3	7.14e-3	4.92e-3	5.64e-3	5.56e-3	5.36e-3	5.06e-3	4.09e-3	5.90e-3	5.45e-3
Pyrimidine	Mean:	2.66e-2	8.79e-4	2.22e-2	8.47e-3	8.38e-3	4.02e-3	5.38e-3	4.55e-3	4.09e-3	3.74e-3	2.23e-3	5.24e-3	4.90e-3
	Std:	1.66e-3	2.98e-4	1.52e-3	2.24e-3	1.84e-3	1.15e-3	1.60e-3	1.52e-3	1.30e-3	1.08e-3	3.59e-4	2.00e-3	1.58e-3
	Max:	2.96e-2	1.35e-3	2.50e-2	1.10e-2	1.04e-2	5.42e-3	7.22e-3	6.32e-3	5.60e-3	4.99e-3	2.74e-3	7.59e-3	6.86e-3
	Min:	2.42e-2	4.28e-4	1.97e-2	5.12e-3	5.63e-3	2.53e-3	3.12e-3	2.56e-3	2.46e-3	2.34e-3	1.65e-3	2.65e-3	2.77e-3
Pyrazine	Mean:	2.56e-2	4.29e-3	2.22e-2	1.44e-2	1.39e-2	9.18e-3	1.06e-2	9.76e-3	9.22e-3	9.08e-3	6.46e-3	1.12e-2	1.02e-2
	Std:	1.37e-3	5.66e-4	1.22e-3	2.66e-3	2.17e-3	1.66e-3	2.09e-3	2.10e-3	1.85e-3	1.69e-3	8.79e-4	2.69e-3	2.16e-3
	Max:	2.80e-2	5.22e-3	2.45e-2	1.75e-2	1.63e-2	1.11e-2	1.28e-2	1.20e-2	1.12e-2	1.09e-2	7.64e-3	1.43e-2	1.28e-2
	Min:	2.36e-2	3.36e-3	2.02e-2	1.05e-2	1.07e-2	7.04e-3	7.59e-3	6.93e-3	6.82e-3	6.75e-3	5.22e-3	7.54e-3	7.24e-3

C Basis Set Performance Plots

All basis set performance plots from the 'Basis Set Analysis' section are given in the following pages. The figures are arranged, such that plots of the same property are listed together. The basis sets are listed along the horizontal axis, and the corresponding property is given on the vertical axis. The electronic structure methods are colored, and the color codes are to be found to the right of each plot. The associated molecule is specified in the parentheses in top of each plot as well as the examined property.

C.1 HOMA Index

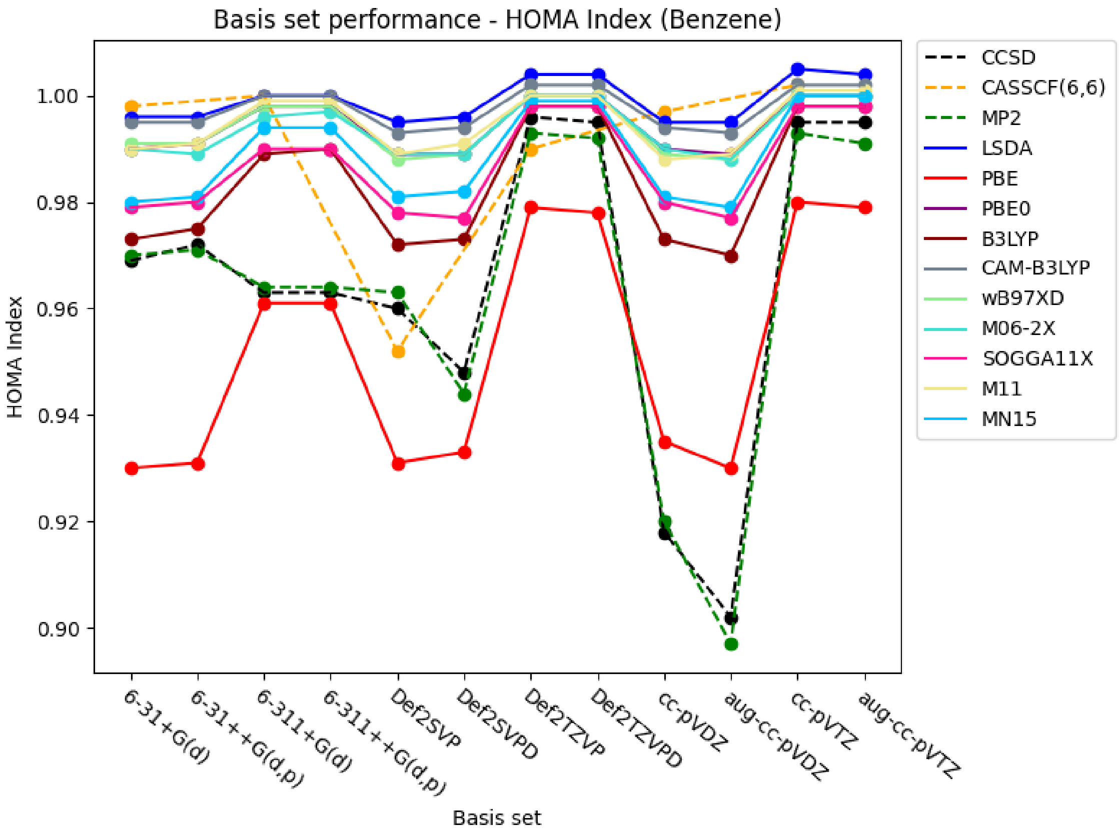

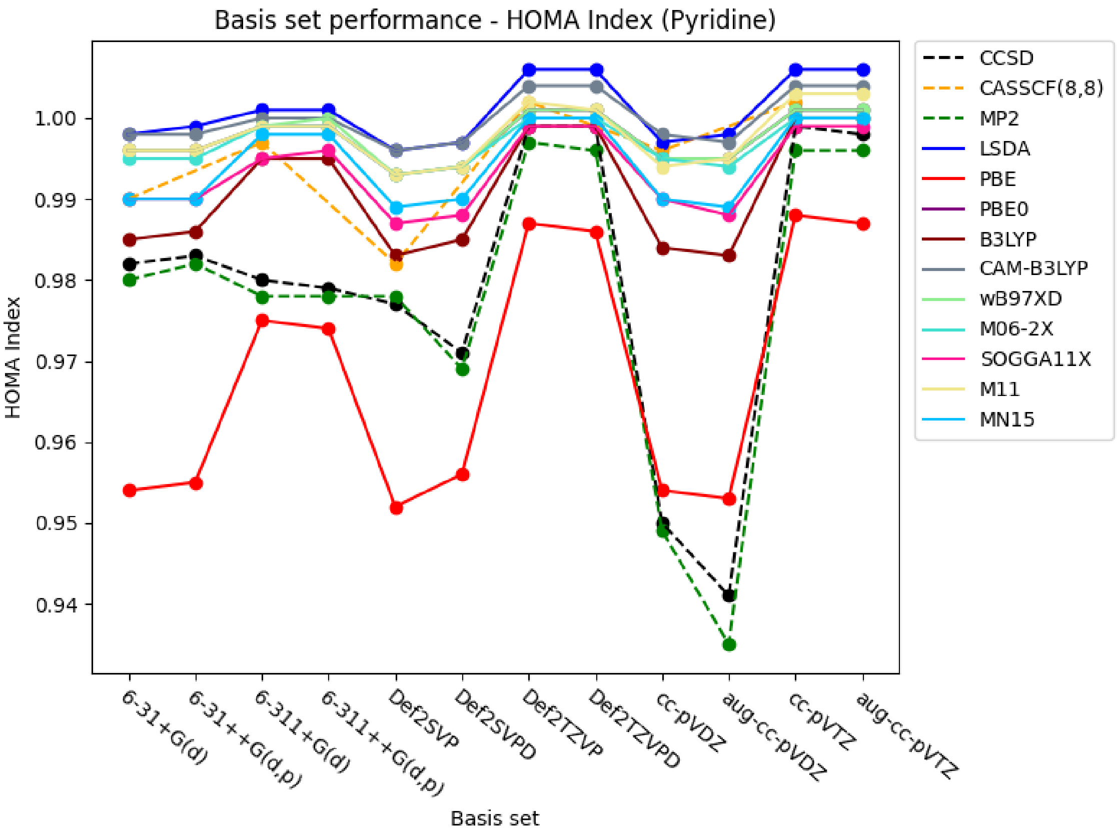
Basis set performance - HOMA Index (Pyridine)
HOMA Index
1.00
0.99
0.98
0.97
0.96
0.95
0.94
6-31+G(d)
6-31++G(d,p)
6-311+G(d)
6-311++G(d,p)
Def2SVP
Def2SVPD
Def2TZVP
Def2TZVPD
cc-pVDZ
aug-cc-pVDZ
cc-pVTZ
aug-cc-pVTZ
Basis set
CCSD
CASSCF(8,8)
MP2
LSDA
PBE
PBE0
B3LYP
CAM-B3LYP
wB97XD
M06-2X
SOGGA11X
M11
MN15

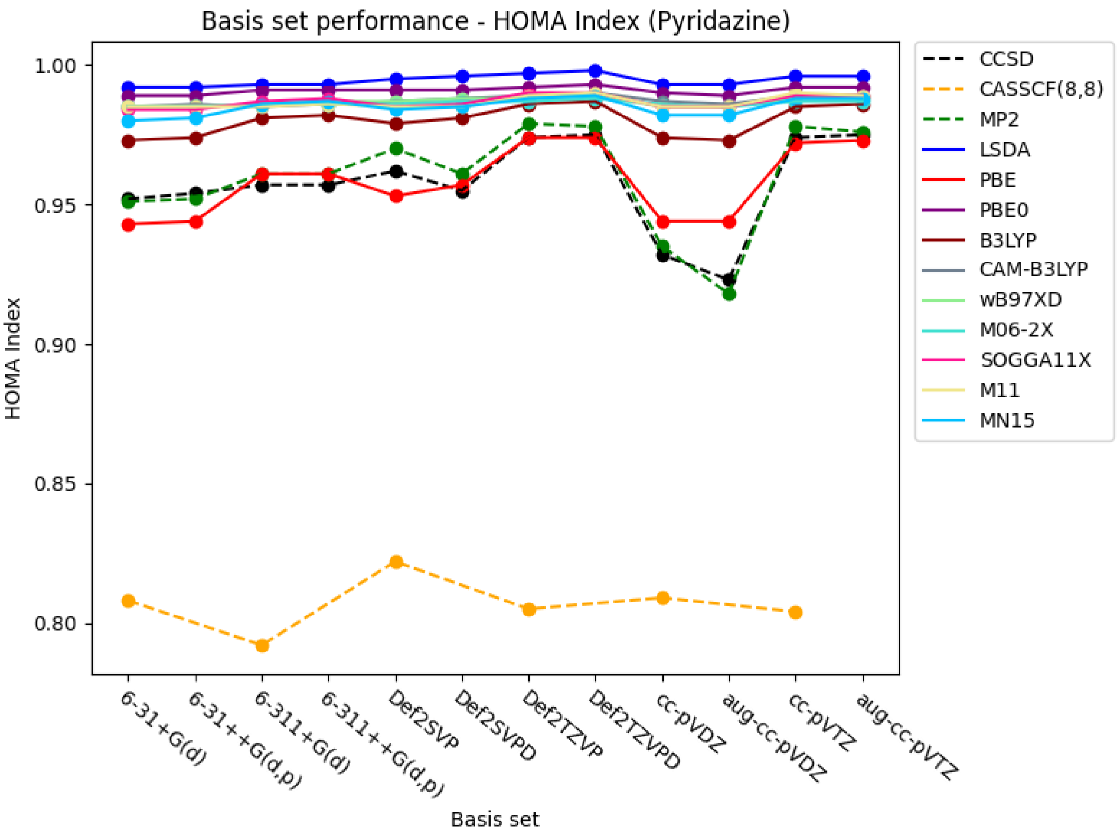
Basis set performance - HOMA Index (Pyridazine)
HOMA Index
1.00
0.95
0.90
0.85
0.80
6-31+G(d)
6-31++G(d,p)
6-311+G(d)
6-311++G(d,p)
Def2SVP
Def2SVPD
Def2TZVP
Def2TZVPD
cc-pVDZ
aug-cc-pVDZ
cc-pVTZ
aug-cc-pVTZ
Basis set
CCSD
CASSCF(8,8)
MP2
LSDA
PBE
PBE0
B3LYP
CAM-B3LYP
wB97XD
M06-2X
SOGGA11X
M11
MN15

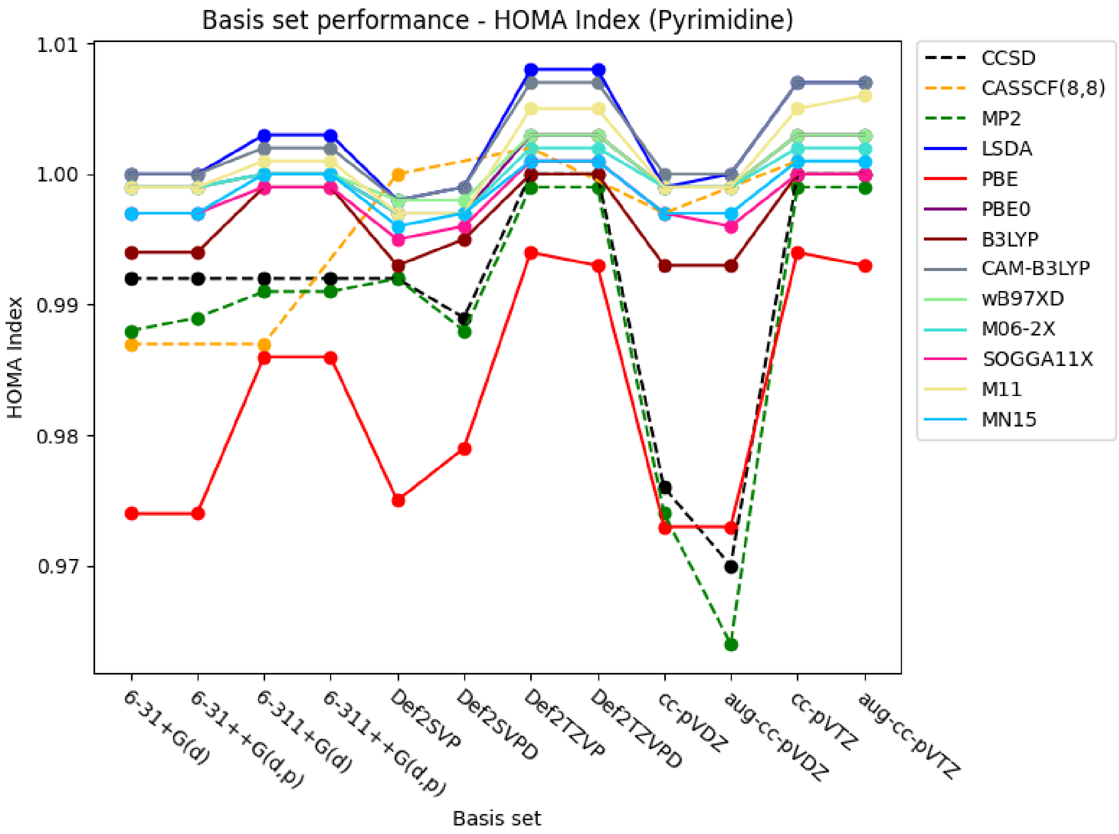
Basis set performance - HOMA Index (Pyrimidine)
HOMA Index
Basis set
1.01
1.00
0.99
0.98
0.97
6-31+G(d)
6-31++G(d,p)
6-311+G(d)
6-311++G(d,p)
Def2SVP
Def2SVPD
Def2TZVP
Def2TZVPD
cc-pVDZ
aug-cc-pVDZ
cc-pVTZ
aug-cc-pVTZ
CCSD
CASSCF(8,8)
MP2
LSDA
PBE
PBE0
B3LYP
CAM-B3LYP
wB97XD
M06-2X
SOGGA11X
M11
MN15

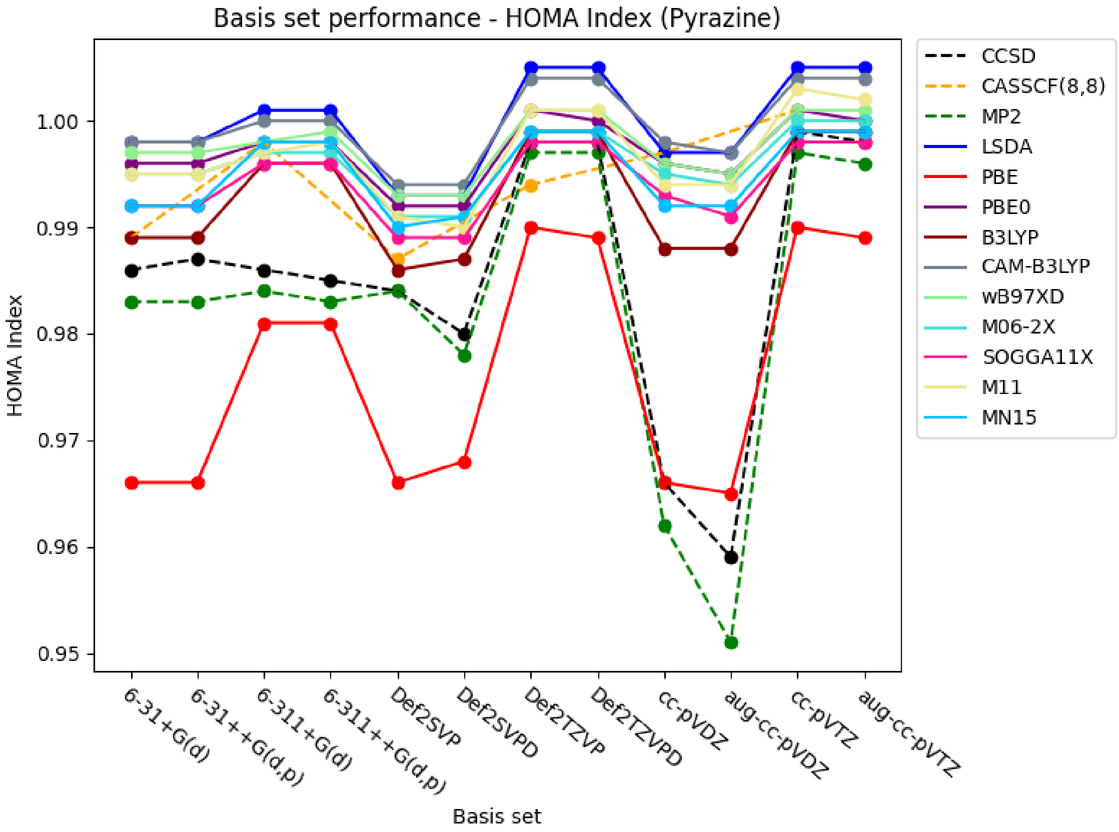
Basis set performance - HOMA Index (Pyrazine)
HOMA Index
Basis set
1.00
0.99
0.98
0.97
0.96
0.95
6-31+G(d)
6-31++G(d,p)
6-311+G(d)
6-311++G(d,p)
Def2SVP
Def2SVPD
Def2TZVP
Def2TZVPD
cc-pVDZ
aug-cc-pVDZ
cc-pVTZ
aug-cc-pVTZ
CCSD
CASSCF(8,8)
MP2
LSDA
PBE
PBE0
B3LYP
CAM-B3LYP
wB97XD
M06-2X
SOGGA11X
M11
MN15

C.2 PDI

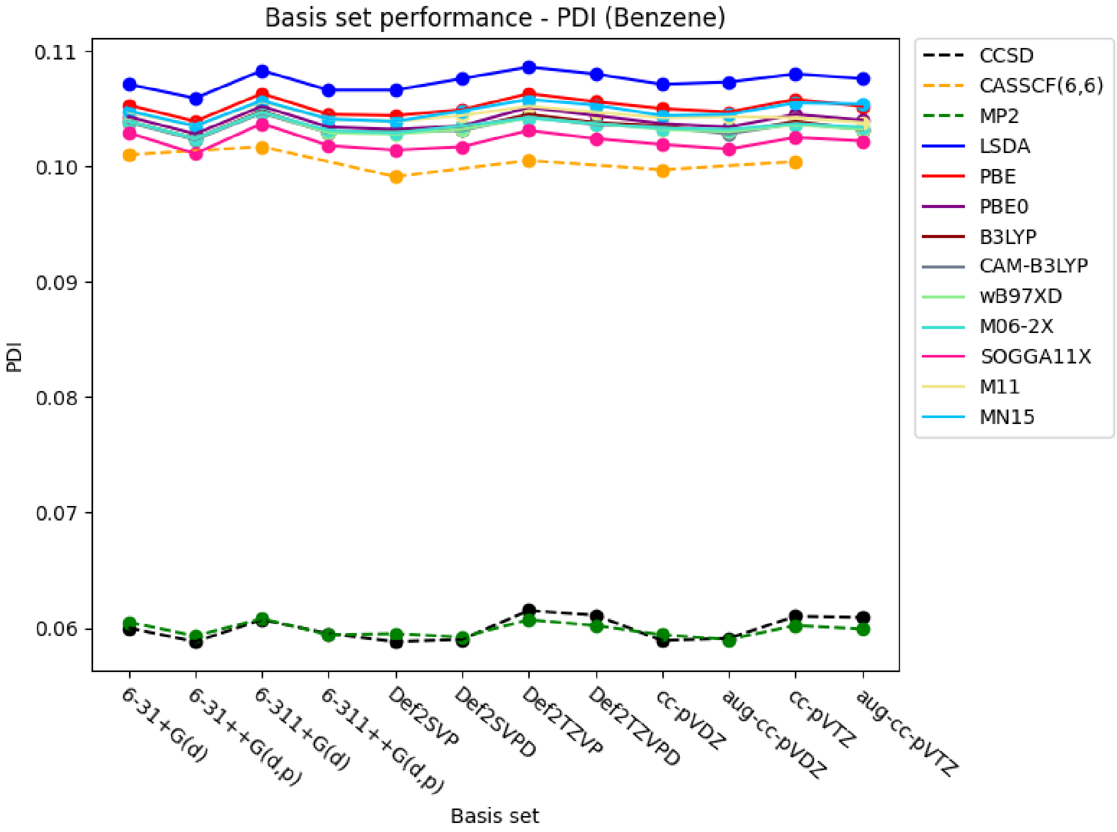

Basis set performance - PDI (Benzene)
0.11
0.10
0.09
0.08
0.07
0.06
PDI
6-31+G(d)
6-31++G(d,p)
6-311+G(d)
6-311++G(d,p)
Def2SVP
Def2SVPD
Def2TZVP
Def2TZVPD
cc-pVDZ
aug-cc-pVDZ
cc-pVTZ
aug-cc-pVTZ
Basis set
CCSD
CASSCF(6,6)
MP2
LSDA
PBE
PBE0
B3LYP
CAM-B3LYP
wB97XD
M06-2X
SOGGA11X
M11
MN15

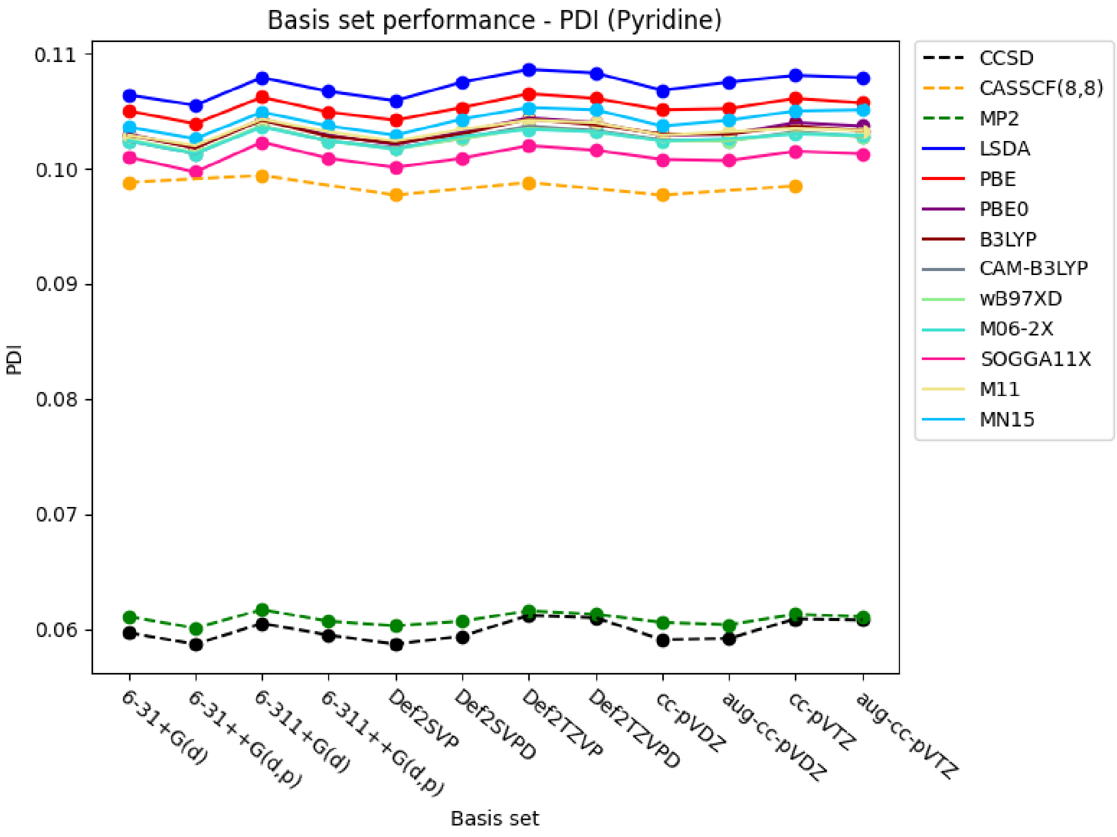
Basis set performance - PDI (Pyridine)
PDI
0.11
0.10
0.09
0.08
0.07
0.06
6-31+G(d)
6-31++G(d,p)
6-311+G(d)
6-311++G(d,p)
Def2SVP
Def2SVPD
Def2TZVP
Def2TZVPD
cc-pVDZ
aug-cc-pVDZ
cc-pVTZ
aug-cc-pVTZ
Basis set
CCSD
CASSCF(8,8)
MP2
LSDA
PBE
PBE0
B3LYP
CAM-B3LYP
wB97XD
M06-2X
SOGGA11X
M11
MN15

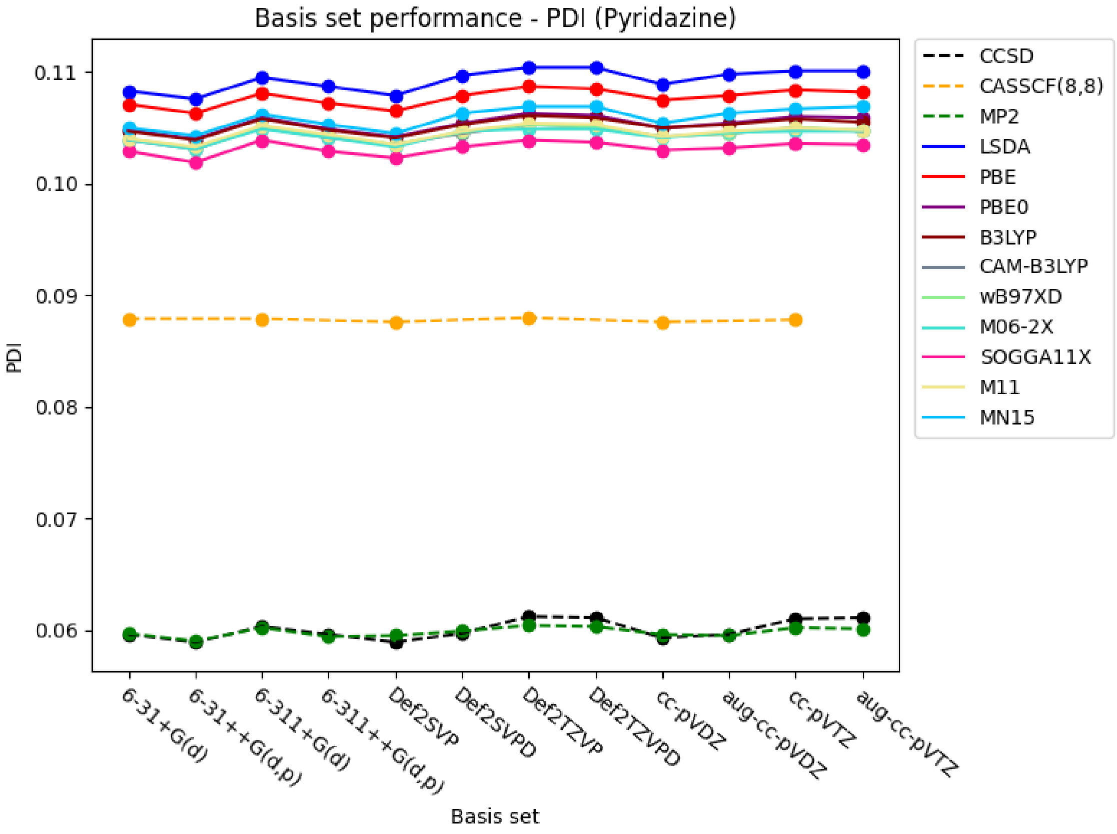
Basis set performance - PDI (Pyridazine)
PDI
0.11
0.10
0.09
0.08
0.07
0.06
6-31+G(d)
6-31++G(d,p)
6-311+G(d)
6-311++G(d,p)
Def2SVP
Def2SVPD
Def2TZVP
Def2TZVPD
cc-pVDZ
aug-cc-pVDZ
cc-pVTZ
aug-cc-pVTZ
Basis set
CCSD
CASSCF(8,8)
MP2
LSDA
PBE
PBE0
B3LYP
CAM-B3LYP
wB97XD
M06-2X
SOGGA11X
M11
MN15

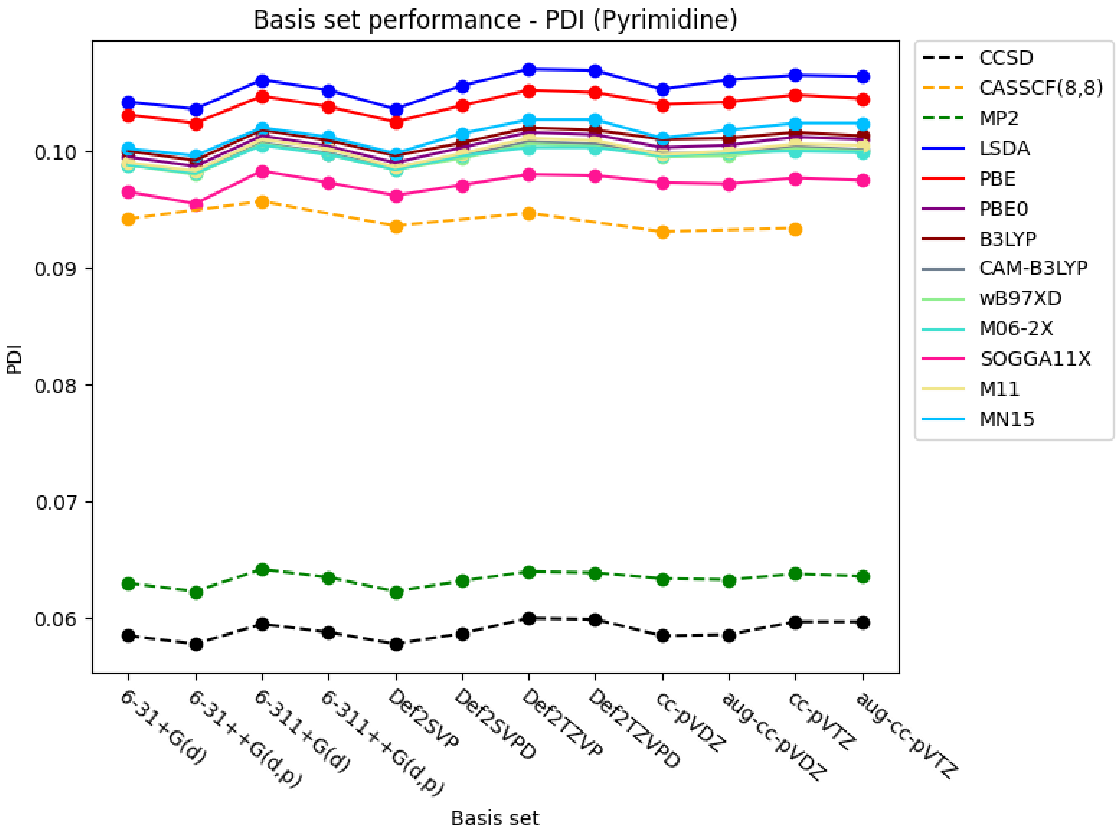
Basis set performance - PDI (Pyrimidine)
PDI
0.10
0.09
0.08
0.07
0.06
6-31+G(d)
6-31++G(d,p)
6-311+G(d)
6-311++G(d,p)
Def2SVP
Def2SVPD
Def2TZVP
Def2TZVPD
cc-pVDZ
aug-cc-pVDZ
cc-pVTZ
aug-cc-pVTZ
Basis set
CCSD
CASSCF(8,8)
MP2
LSDA
PBE
PBE0
B3LYP
CAM-B3LYP
wB97XD
M06-2X
SOGGA11X
M11
MN15

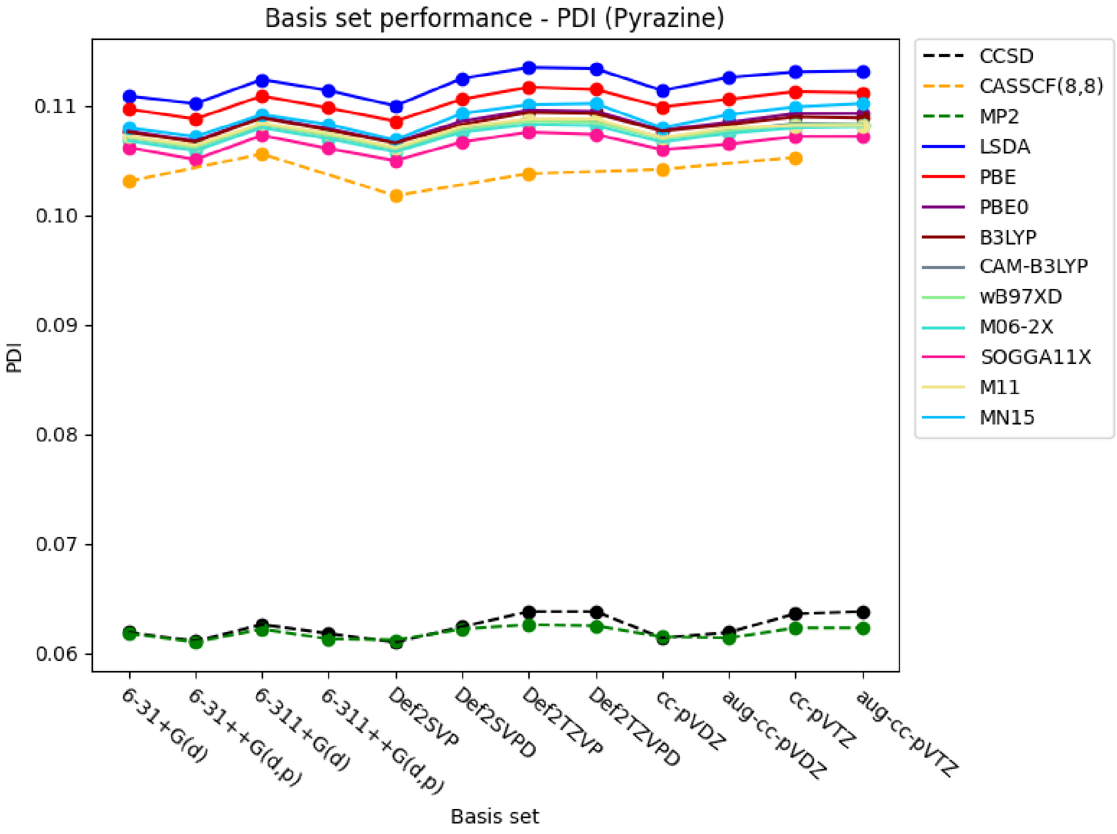
Basis set performance - PDI (Pyrazine)
PDI
0.11
0.10
0.09
0.08
0.07
0.06
6-31+G(d)
6-31++G(d,p)
6-311+G(d)
6-311++G(d,p)
Def2SVP
Def2SVPD
Def2TZVP
Def2TZVPD
cc-pVDZ
aug-cc-pVDZ
cc-pVTZ
aug-cc-pVTZ
Basis set
CCSD
CASSCF(8,8)
MP2
LSDA
PBE
PBE0
B3LYP
CAM-B3LYP
wB97XD
M06-2X
SOGGA11X
M11
MN15

C.3 MCI

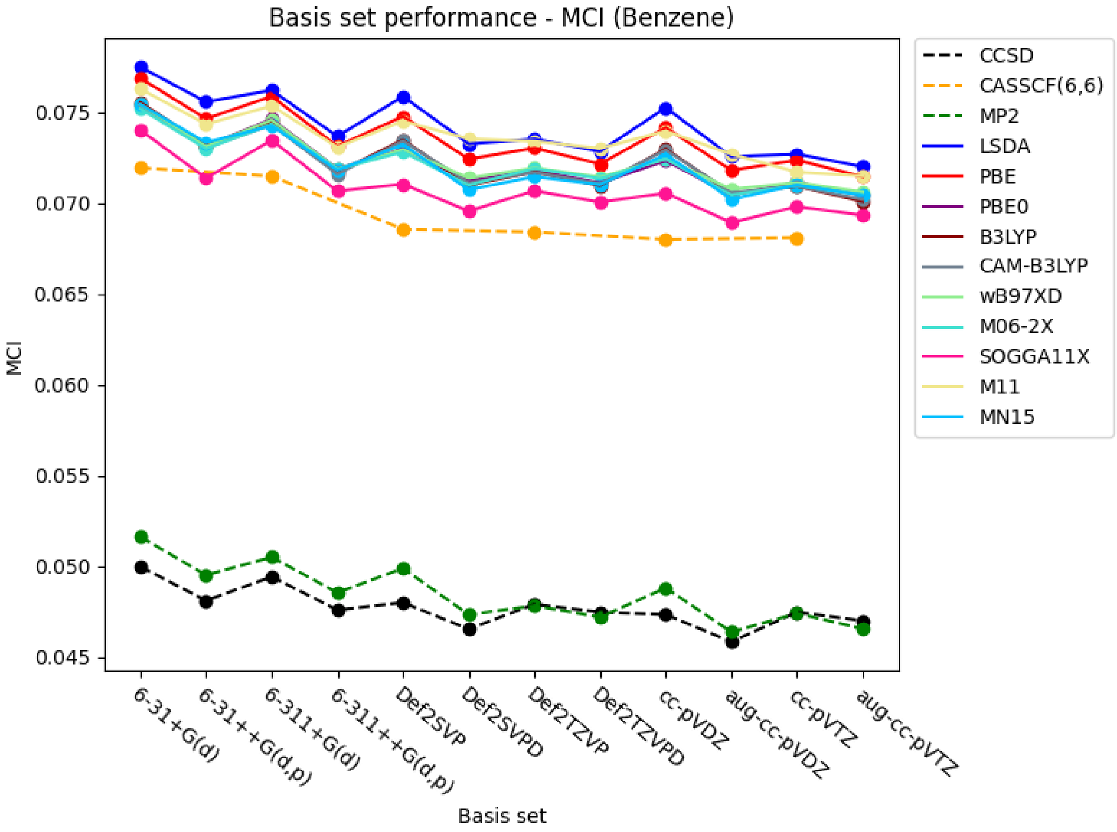

Basis set performance - MCI (Benzene)
MCI
0.075
0.070
0.065
0.060
0.055
0.050
0.045
6-31+G(d)
6-31++G(d,p)
6-311+G(d)
6-311++G(d,p)
Def2SVP
Def2SVPD
Def2TZVP
Def2TZVPD
cc-pVDZ
aug-cc-pVDZ
cc-pVTZ
aug-cc-pVTZ
Basis set
CCSD
CASSCF(6,6)
MP2
LSDA
PBE
PBE0
B3LYP
CAM-B3LYP
wB97XD
M06-2X
SOGGA11X
M11
MN15

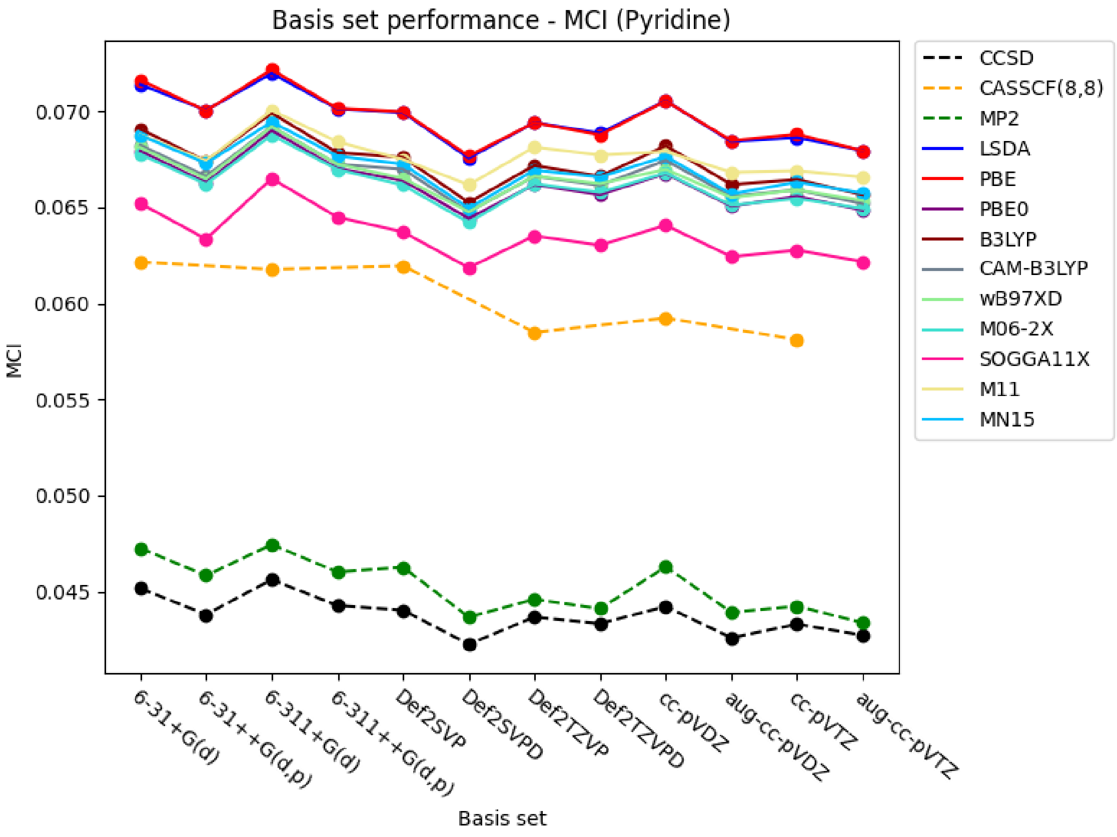
Basis set performance - MCI (Pyridine)
MCI
0.070
0.065
0.060
0.055
0.050
0.045
6-31+G(d)
6-31++G(d,p)
6-311+G(d)
6-311++G(d,p)
Def2SVP
Def2SVPD
Def2TZVP
Def2TZVPD
cc-pVDZ
aug-cc-pVDZ
cc-pVTZ
aug-cc-pVTZ
Basis set
CCSD
CASSCF(8,8)
MP2
LSDA
PBE
PBE0
B3LYP
CAM-B3LYP
wB97XD
M06-2X
SOGGA11X
M11
MN15

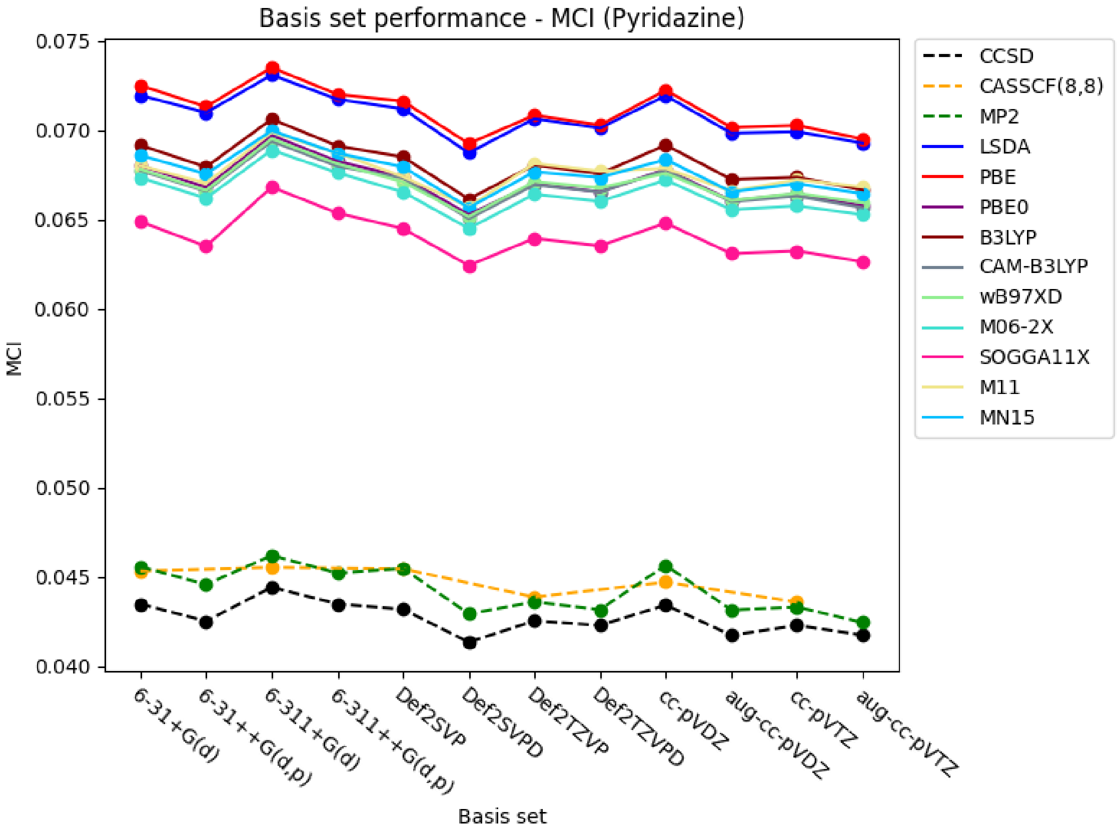
Basis set performance - MCI (Pyridazine)
MCI
0.075
0.070
0.065
0.060
0.055
0.050
0.045
0.040
6-31+G(d)
6-31++G(d,p)
6-311+G(d)
6-311++G(d,p)
Def2SVP
Def2SVPD
Def2TZVP
Def2TZVPD
cc-pVDZ
aug-cc-pVDZ
cc-pVTZ
aug-cc-pVTZ
Basis set
CCSD
CASSCF(8,8)
MP2
LSDA
PBE
PBE0
B3LYP
CAM-B3LYP
wB97XD
M06-2X
SOGGA11X
M11
MN15

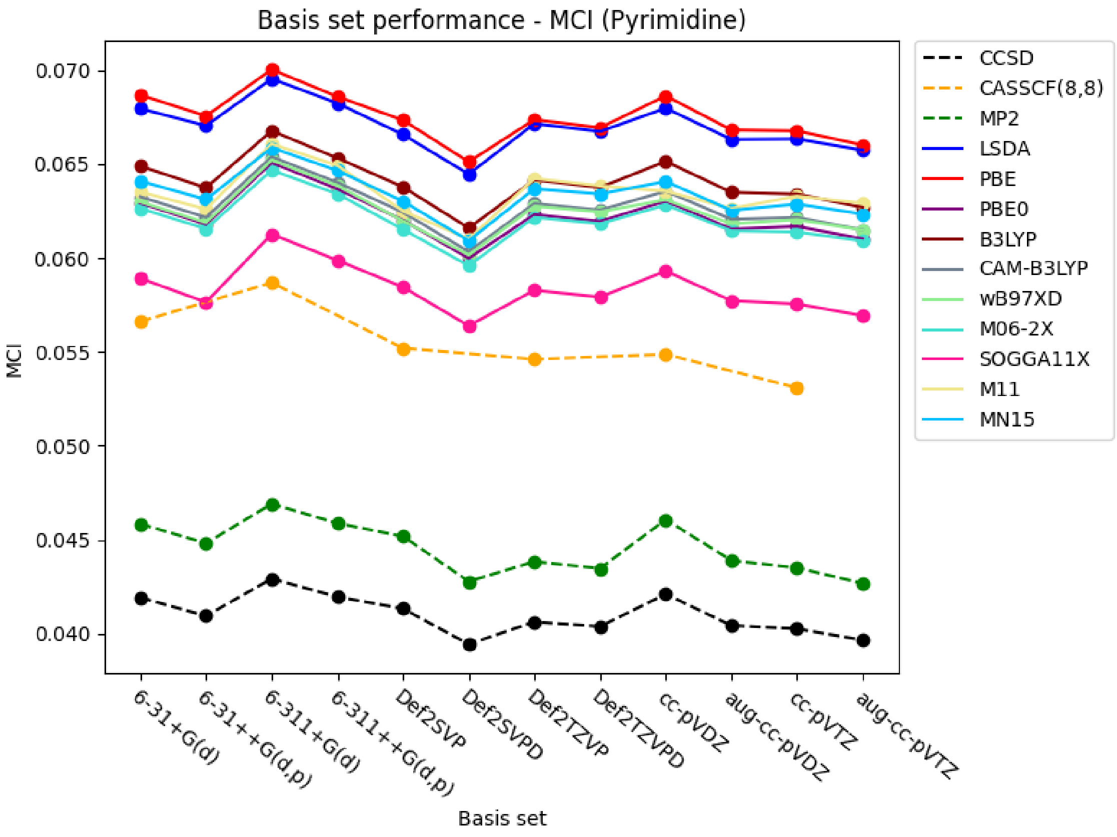

Basis set performance - MCI (Pyrimidine)
MCI
Basis set
0.070
0.065
0.060
0.055
0.050
0.045
0.040
6-31+G(d)
6-31++G(d,p)
6-311+G(d)
6-311++G(d,p)
Def2SVP
Def2SVPD
Def2TZVP
Def2TZVPD
cc-pVDZ
aug-cc-pVDZ
cc-pVTZ
aug-cc-pVTZ
CCSD
CASSCF(8,8)
MP2
LSDA
PBE
PBE0
B3LYP
CAM-B3LYP
wB97XD
M06-2X
SOGGA11X
M11
MN15

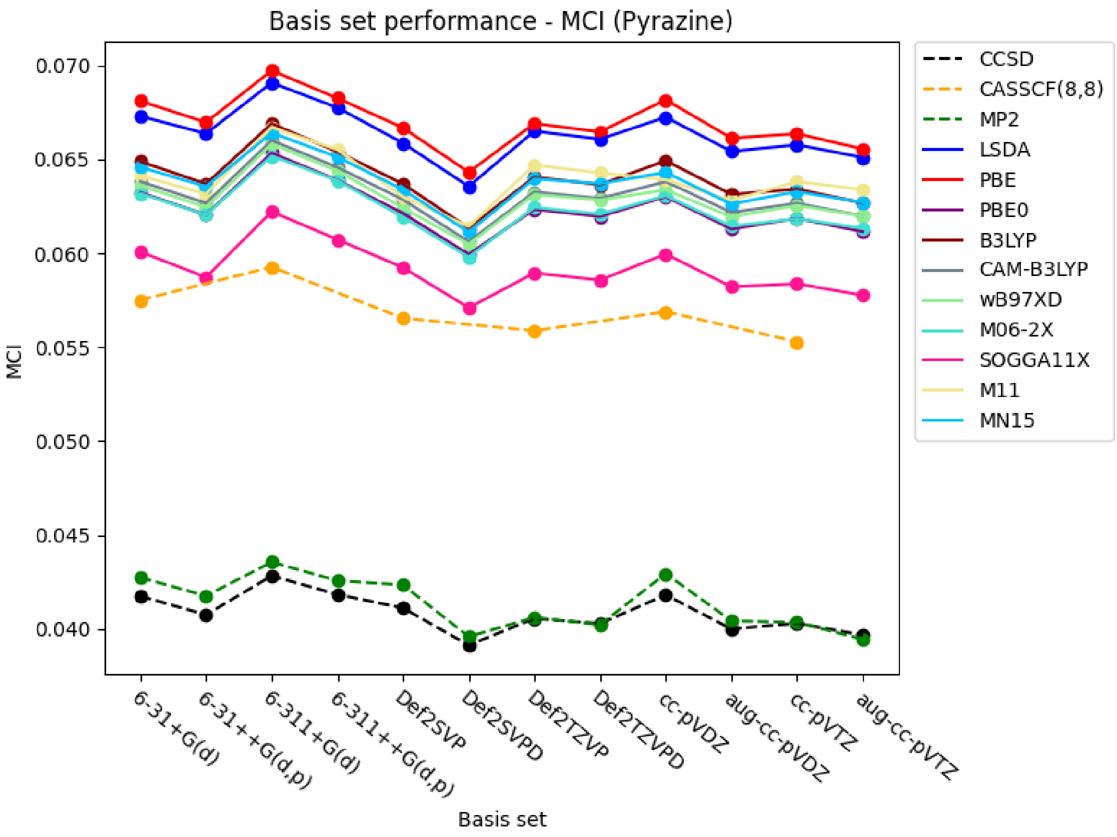

Basis set performance - MCI (Pyrazine)
MCI
Basis set
0.070
0.065
0.060
0.055
0.050
0.045
0.040
6-31+G(d)
6-31++G(d,p)
6-311+G(d)
6-311++G(d,p)
Def2SVP
Def2SVPD
Def2TZVP
Def2TZVPD
cc-pVDZ
aug-cc-pVDZ
cc-pVTZ
aug-cc-pVTZ
CCSD
CASSCF(8,8)
MP2
LSDA
PBE
PBE0
B3LYP
CAM-B3LYP
wB97XD
M06-2X
SOGGA11X
M11
MN15

C.4 AV1245 Index

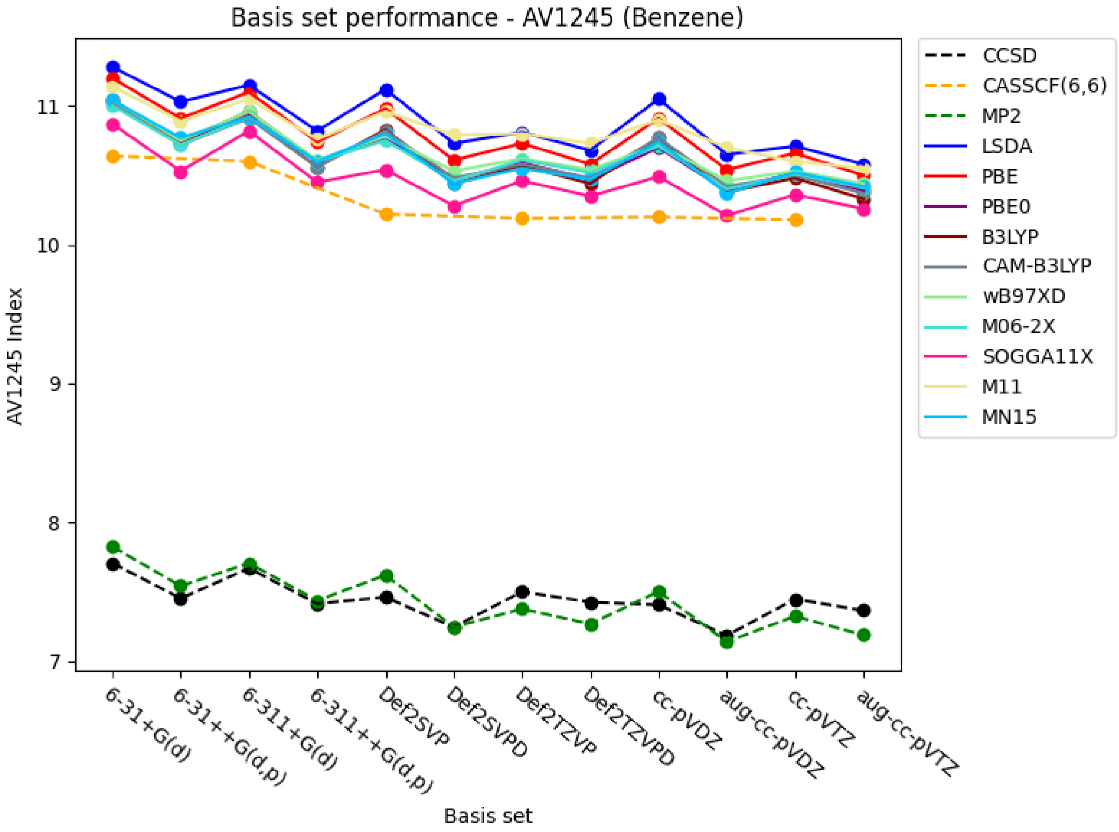

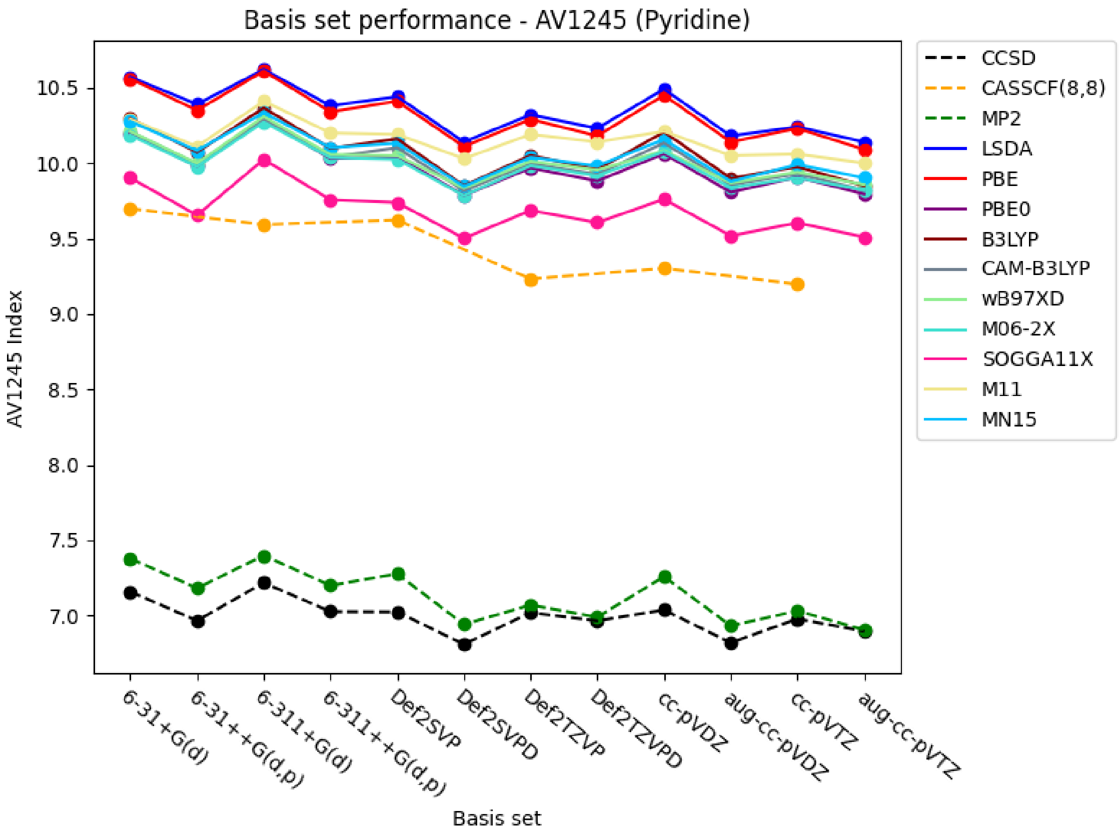
Basis set performance - AV1245 (Pyridine)
AV1245 Index
10.5
10.0
9.5
9.0
8.5
8.0
7.5
7.0
6-31+G(d)
6-31++G(d,p)
6-311+G(d)
6-311++G(d,p)
Def2SVP
Def2SVPD
Def2TZVP
Def2TZVPD
cc-pVDZ
aug-cc-pVDZ
cc-pVTZ
aug-cc-pVTZ
Basis set
CCSD
CASSCF(8,8)
MP2
LSDA
PBE
PBE0
B3LYP
CAM-B3LYP
wB97XD
M06-2X
SOGGA11X
M11
MN15

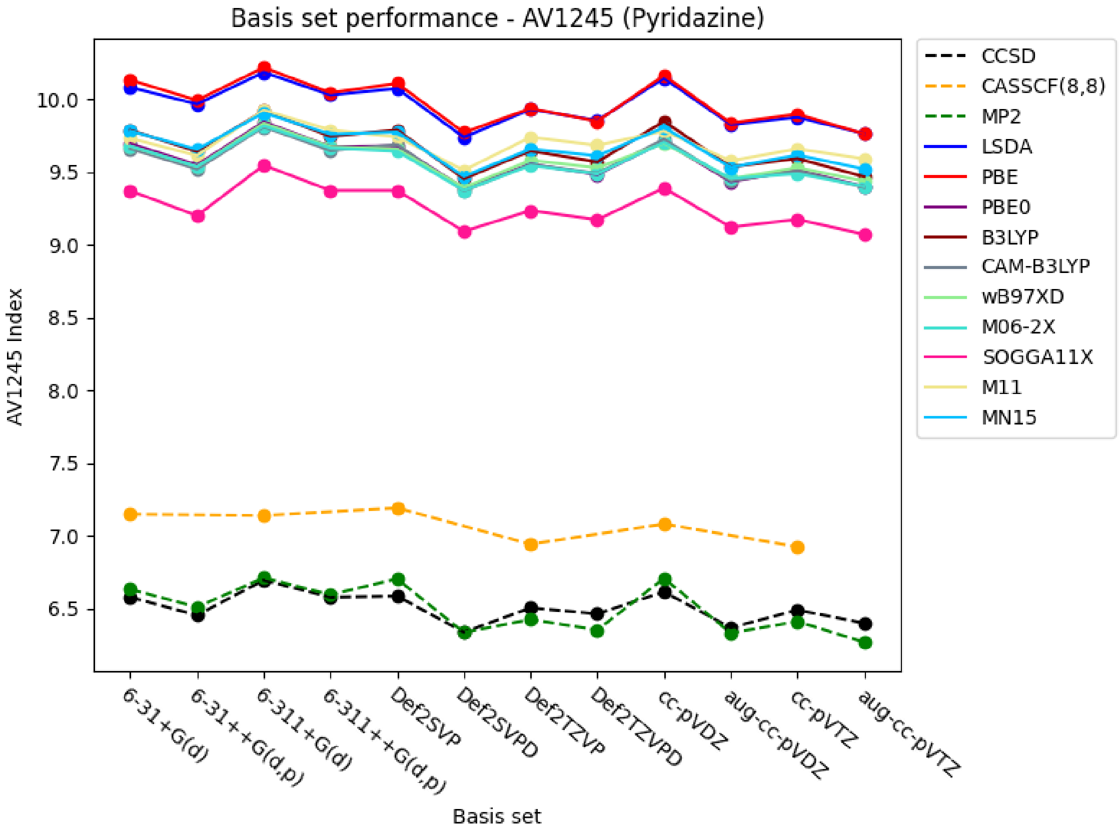
Basis set performance - AV1245 (Pyridazine)
AV1245 Index
10.0
9.5
9.0
8.5
8.0
7.5
7.0
6.5
6-31+G(d)
6-31++G(d,p)
6-311+G(d)
6-311++G(d,p)
Def2SVP
Def2SVPD
Def2TZVP
Def2TZVPD
cc-pVDZ
aug-cc-pVDZ
cc-pVTZ
aug-cc-pVTZ
Basis set
CCSD
CASSCF(8,8)
MP2
LSDA
PBE
PBE0
B3LYP
CAM-B3LYP
wB97XD
M06-2X
SOGGA11X
M11
MN15

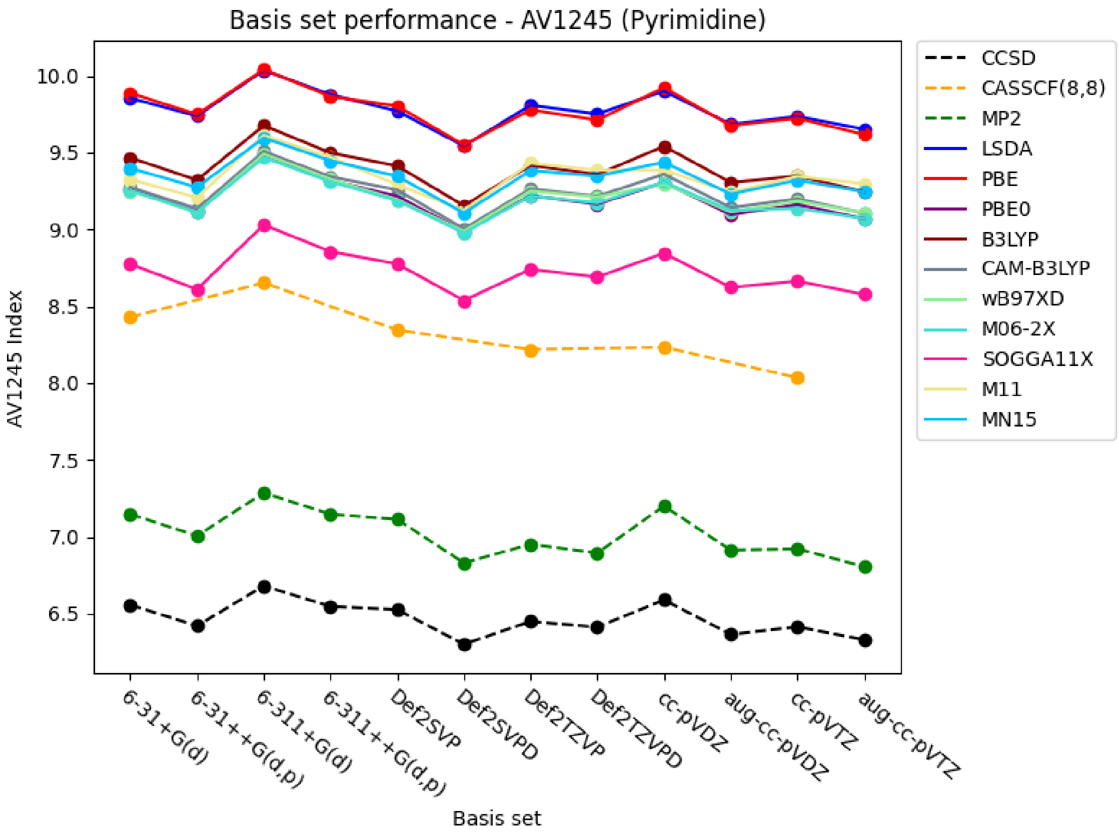
Basis set performance - AV1245 (Pyrimidine)
AV1245 Index
Basis set
CCSD
CASSCF(8,8)
MP2
LSDA
PBE
PBE0
B3LYP
CAM-B3LYP
wB97XD
M06-2X
SOGGA11X
M11
MN15
6-31+G(d)
6-31++G(d,p)
6-311+G(d)
6-311++G(d,p)
Def2SVP
Def2SVPD
Def2TZVP
Def2TZVPD
cc-pVDZ
aug-cc-pVDZ
cc-pVTZ
aug-cc-pVTZ

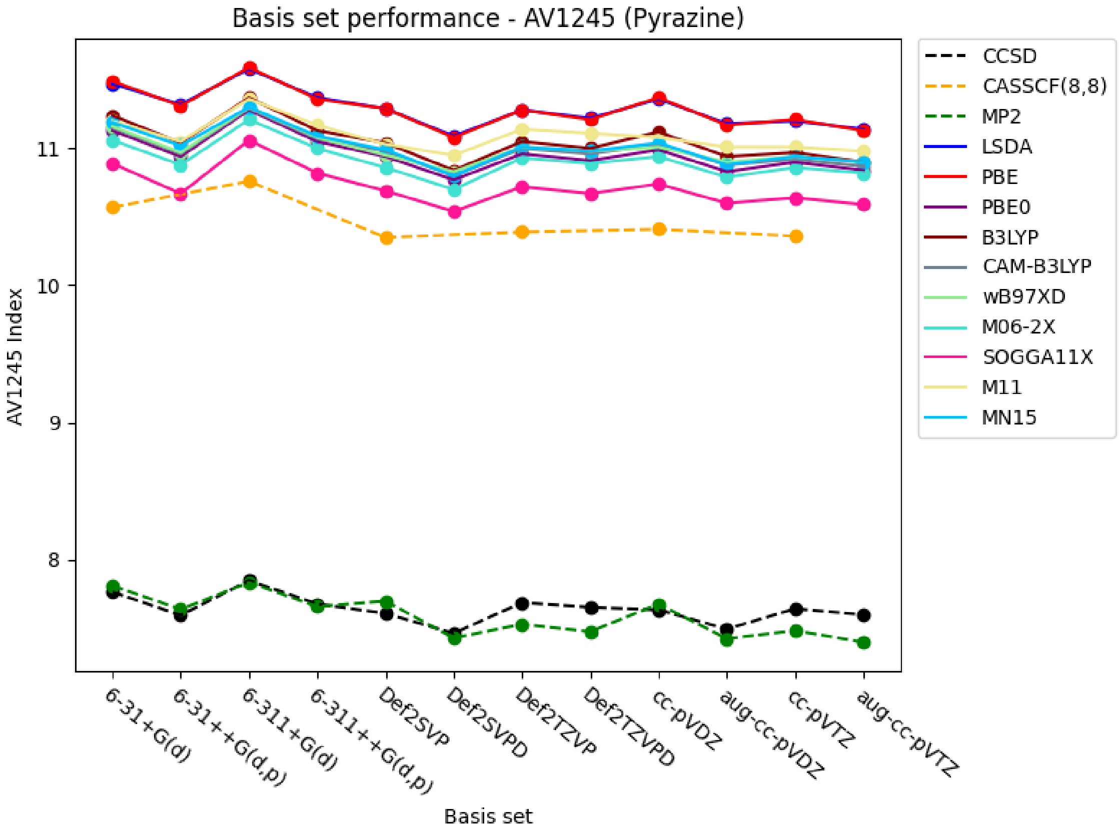
Basis set performance - AV1245 (Pyrazine)
AV1245 Index
Basis set
CCSD
CASSCF(8,8)
MP2
LSDA
PBE
PBE0
B3LYP
CAM-B3LYP
wB97XD
M06-2X
SOGGA11X
M11
MN15
6-31+G(d)
6-31++G(d,p)
6-311+G(d)
6-311++G(d,p)
Def2SVP
Def2SVPD
Def2TZVP
Def2TZVPD
cc-pVDZ
aug-cc-pVDZ
cc-pVTZ
aug-cc-pVTZ

C.5 FLU Index

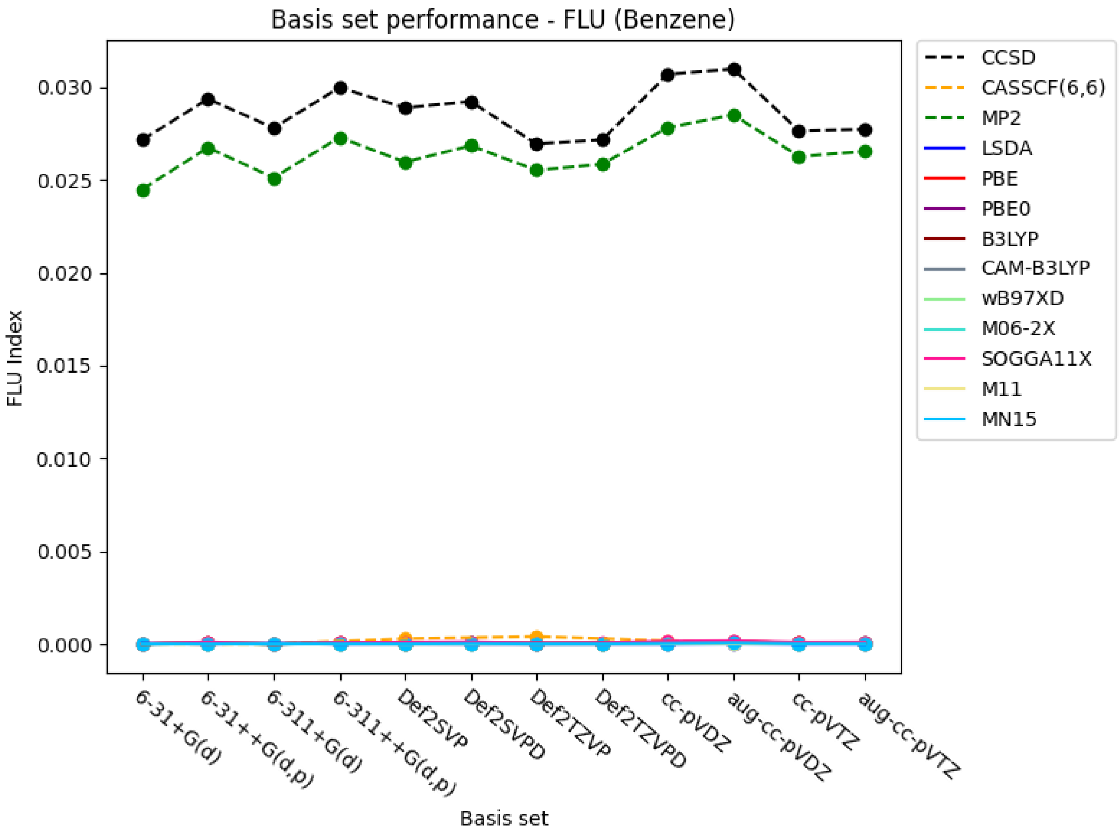

The following is a zoom-in of the figure above:

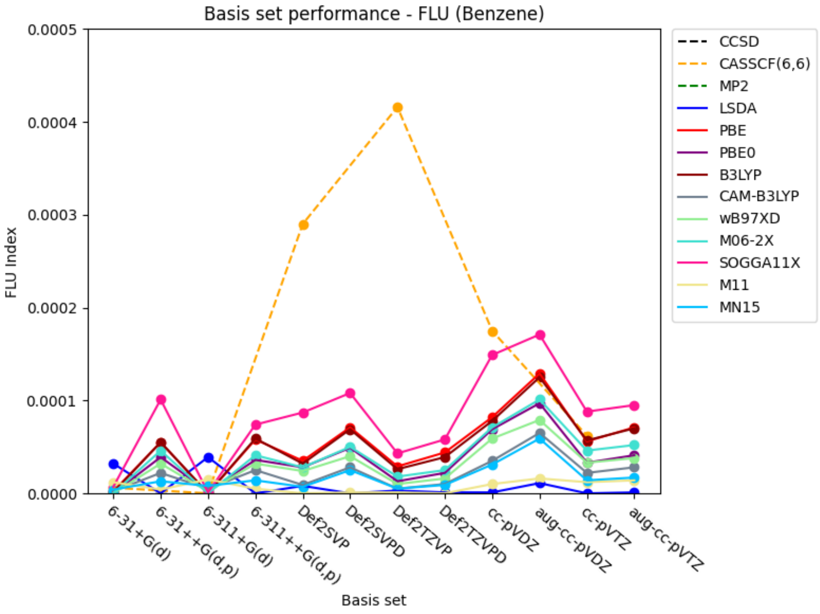

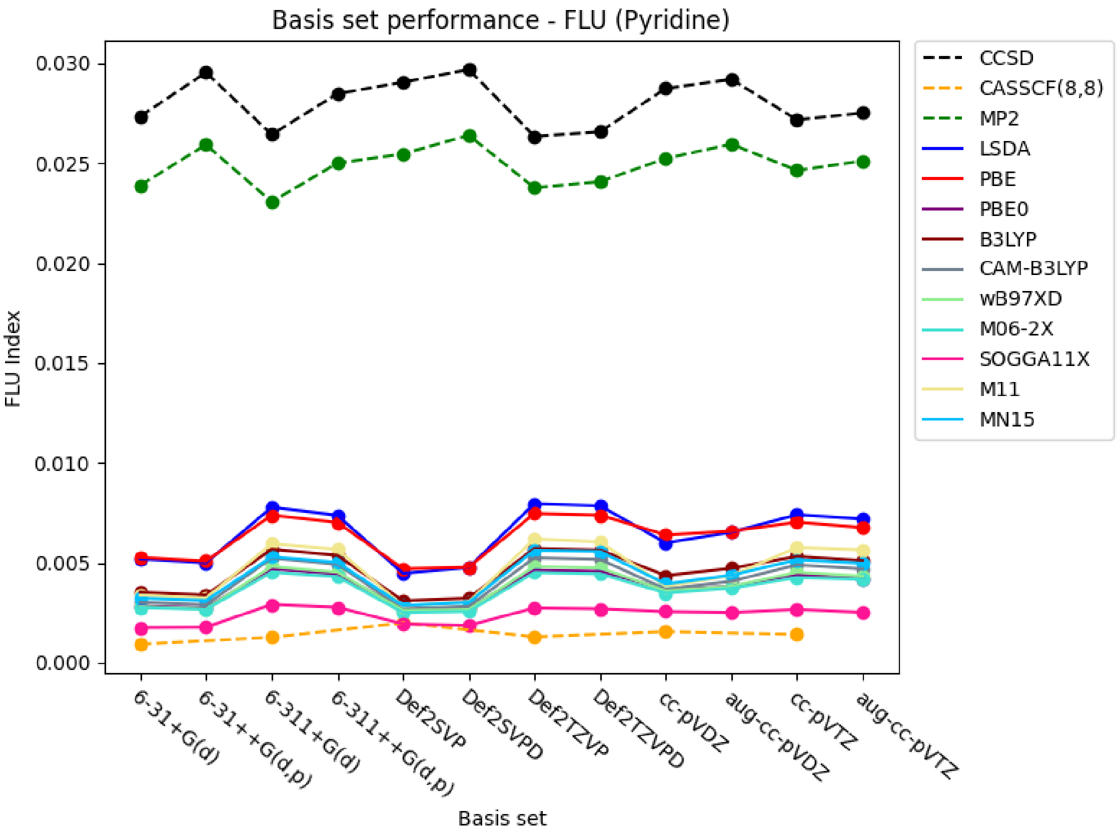
Basis set performance - FLU (Pyridine)
FLU Index
Basis set
0.030
0.025
0.020
0.015
0.010
0.005
0.000
6-31+G(d)
6-31++G(d,p)
6-311+G(d)
6-311++G(d,p)
Def2SVP
Def2SVPD
Def2TZVP
Def2TZVPD
cc-pVDZ
aug-cc-pVDZ
cc-pVTZ
aug-cc-pVTZ
CCSD
CASSCF(8,8)
MP2
LSDA
PBE
PBE0
B3LYP
CAM-B3LYP
wB97XD
M06-2X
SOGGA11X
M11
MN15

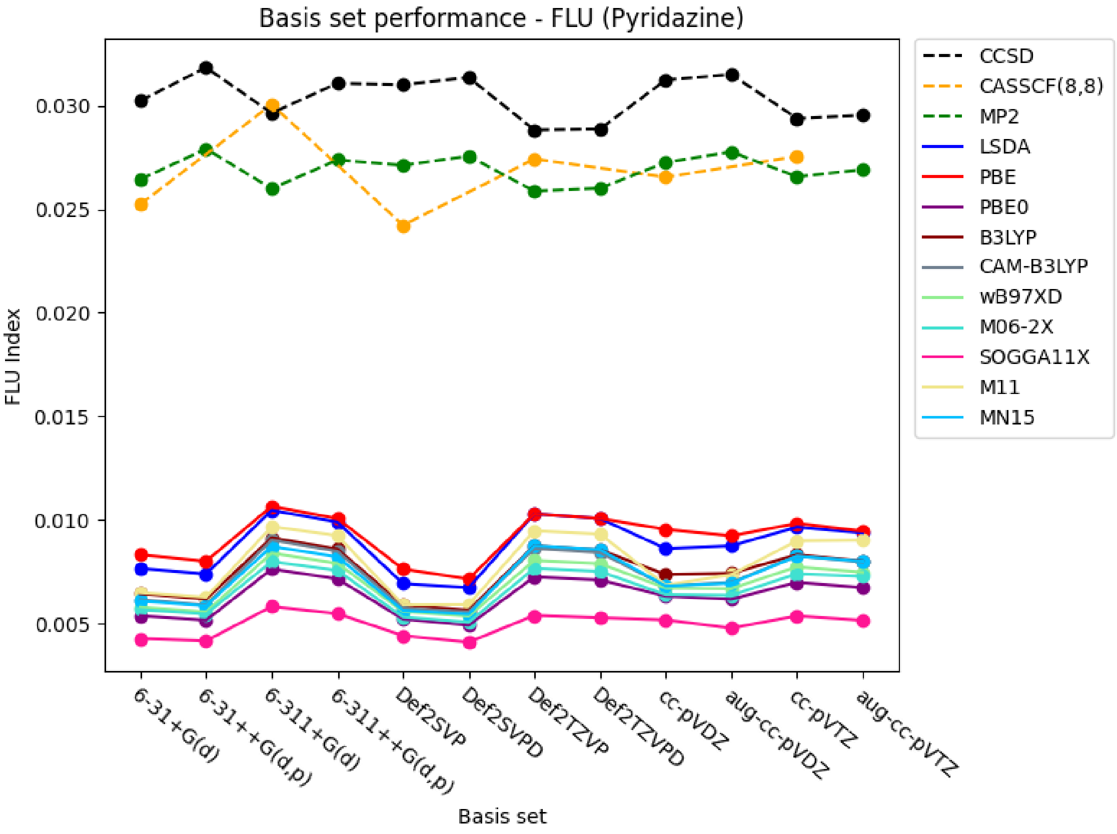
Basis set performance - FLU (Pyridazine)
FLU Index
Basis set
0.030
0.025
0.020
0.015
0.010
0.005
6-31+G(d)
6-31++G(d,p)
6-311+G(d)
6-311++G(d,p)
Def2SVP
Def2SVPD
Def2TZVP
Def2TZVPD
cc-pVDZ
aug-cc-pVDZ
cc-pVTZ
aug-cc-pVTZ
CCSD
CASSCF(8,8)
MP2
LSDA
PBE
PBE0
B3LYP
CAM-B3LYP
wB97XD
M06-2X
SOGGA11X
M11
MN15

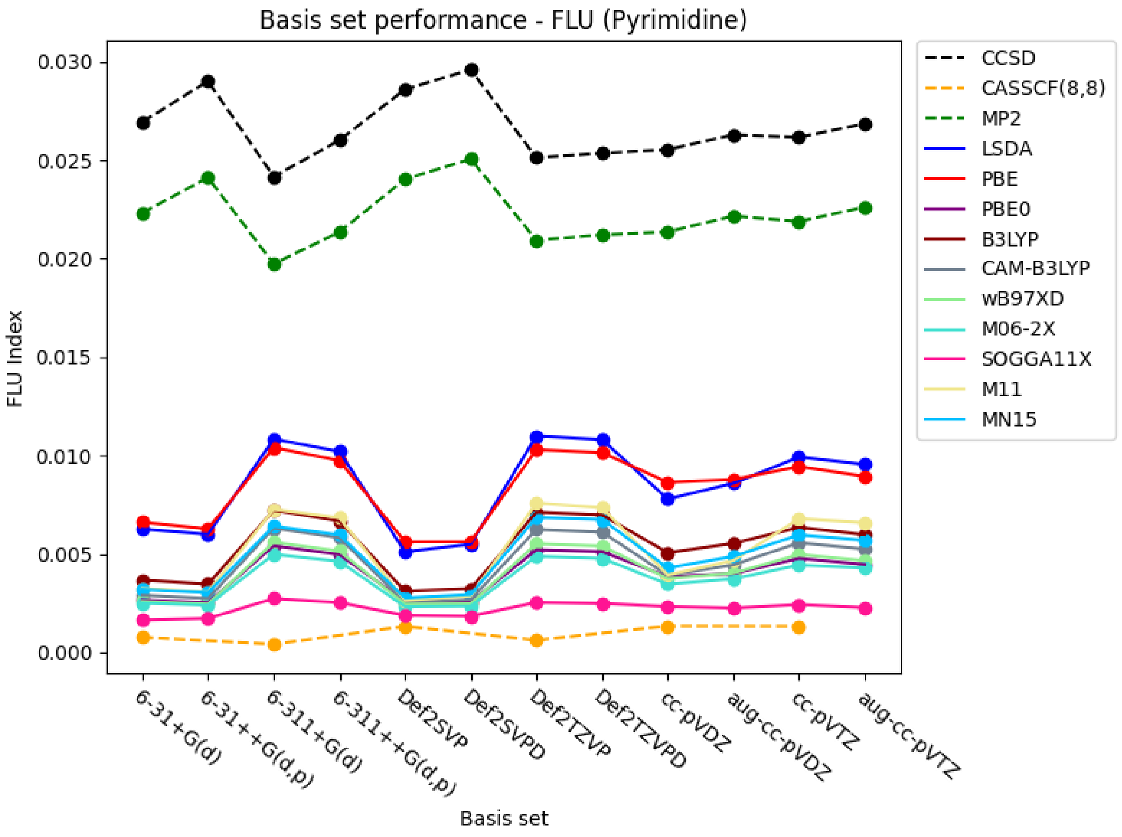
Basis set performance - FLU (Pyrimidine)
FLU Index
0.030
0.025
0.020
0.015
0.010
0.005
0.000
Basis set
6-31+G(d)
6-31++G(d,p)
6-311+G(d)
6-311++G(d,p)
Def2SVP
Def2SVPD
Def2TZVP
Def2TZVPD
cc-pVDZ
aug-cc-pVDZ
cc-pVTZ
aug-cc-pVTZ
CCSD
CASSCF(8,8)
MP2
LSDA
PBE
PBE0
B3LYP
CAM-B3LYP
wB97XD
M06-2X
SOGGA11X
M11
MN15

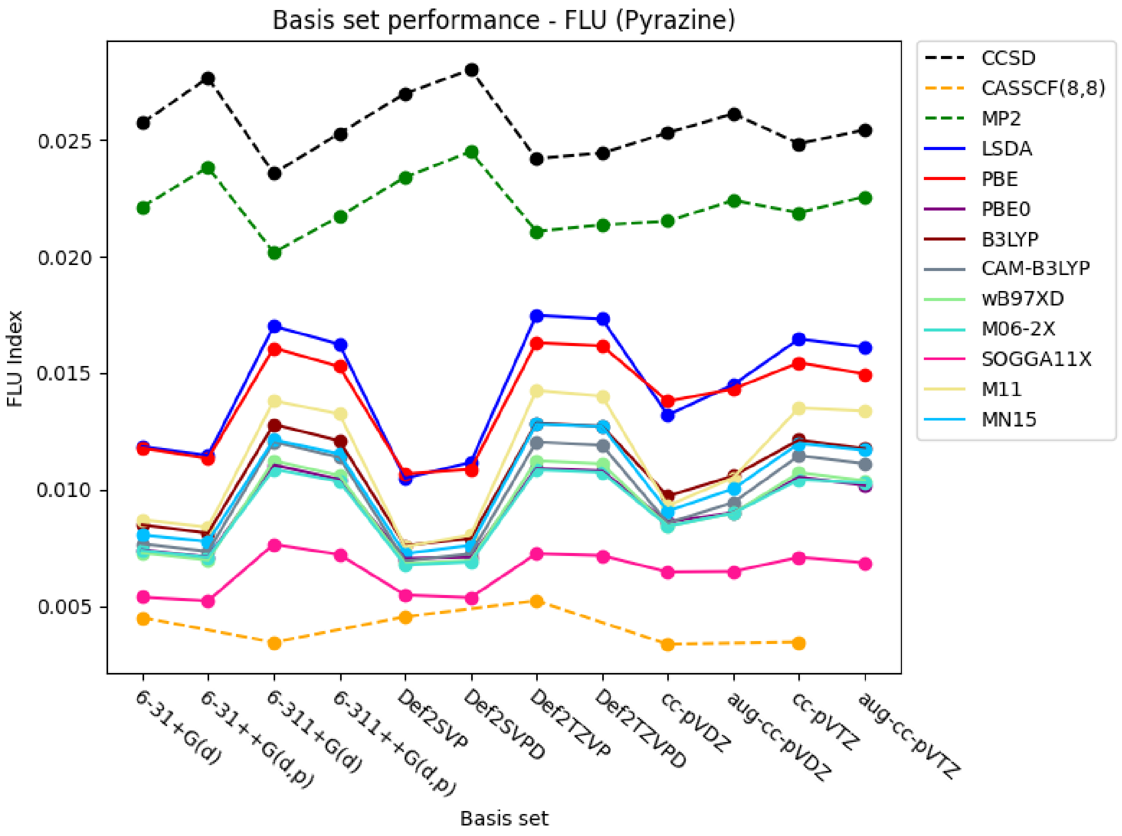
Basis set performance - FLU (Pyrazine)
FLU Index
0.025
0.020
0.015
0.010
0.005
Basis set
6-31+G(d)
6-31++G(d,p)
6-311+G(d)
6-311++G(d,p)
Def2SVP
Def2SVPD
Def2TZVP
Def2TZVPD
cc-pVDZ
aug-cc-pVDZ
cc-pVTZ
aug-cc-pVTZ
CCSD
CASSCF(8,8)
MP2
LSDA
PBE
PBE0
B3LYP
CAM-B3LYP
wB97XD
M06-2X
SOGGA11X
M11
MN15

C.6 Electronic Energy

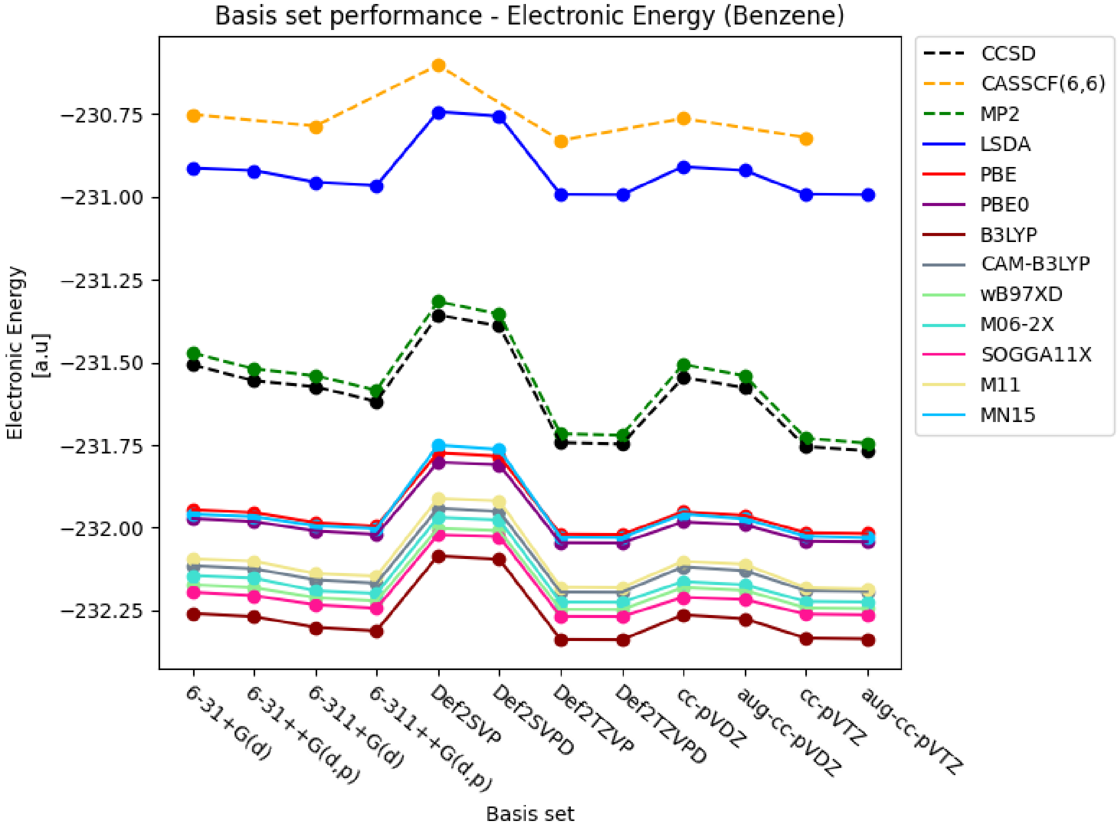

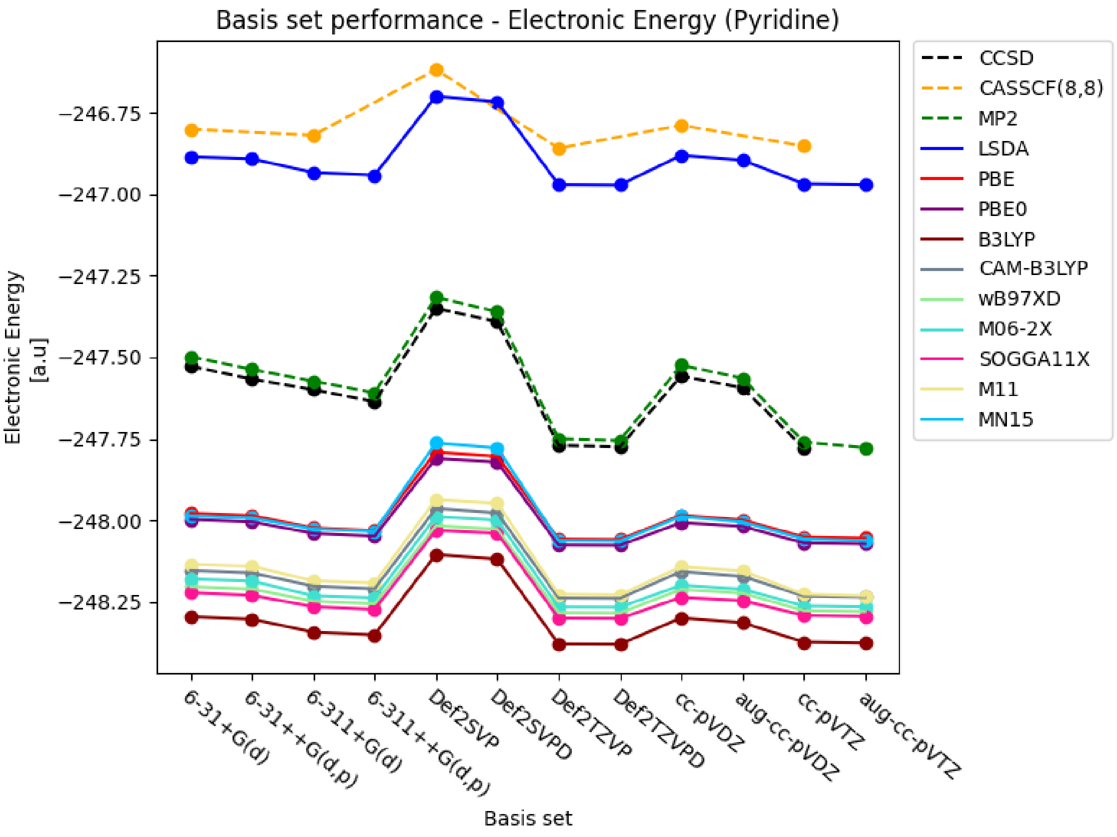
Basis set performance - Electronic Energy (Pyridine)
Electronic Energy
[a.u]
−246.75
−247.00
−247.25
−247.50
−247.75
−248.00
−248.25
6-31+G(d)
6-31++G(d,p)
6-311+G(d)
6-311++G(d,p)
Def2SVP
Def2SVPD
Def2TZVP
Def2TZVPD
cc-pVDZ
aug-cc-pVDZ
cc-pVTZ
aug-cc-pVTZ
Basis set
CCSD
CASSCF(8,8)
MP2
LSDA
PBE
PBE0
B3LYP
CAM-B3LYP
wB97XD
M06-2X
SOGGA11X
M11
MN15

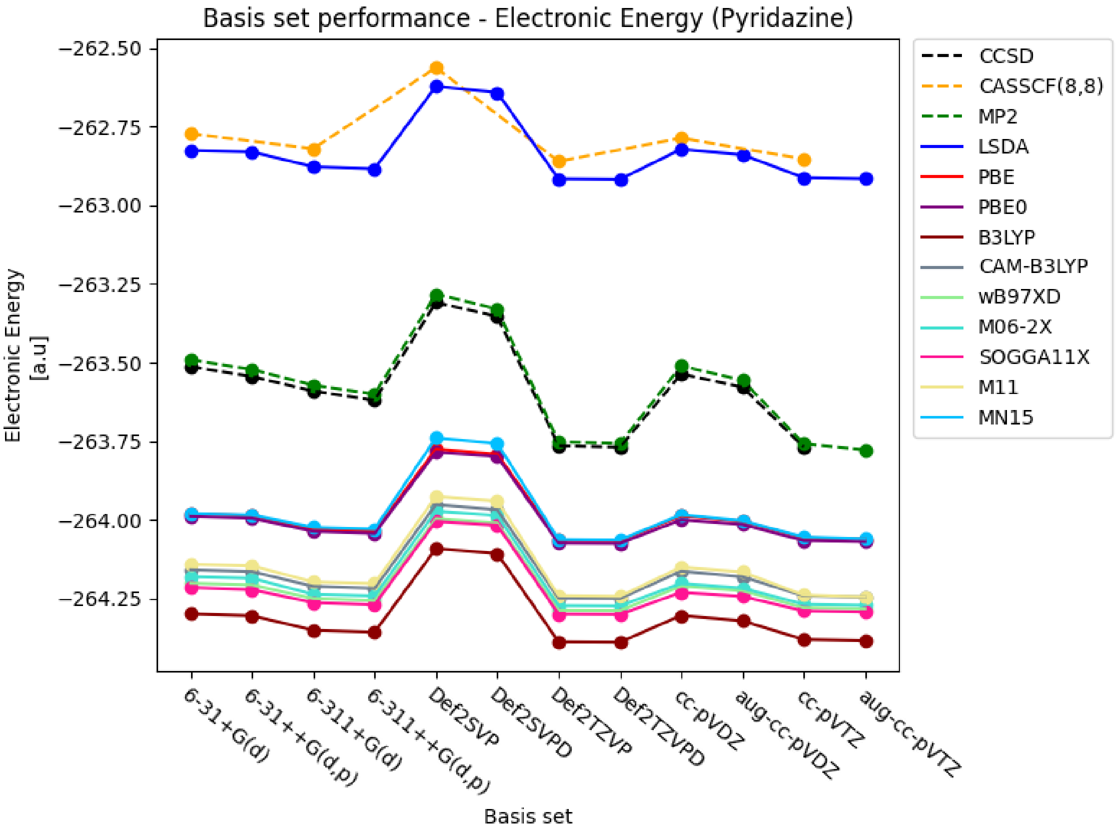
Basis set performance - Electronic Energy (Pyridazine)
Electronic Energy
[a.u]
−262.50
−262.75
−263.00
−263.25
−263.50
−263.75
−264.00
−264.25
6-31+G(d)
6-31++G(d,p)
6-311+G(d)
6-311++G(d,p)
Def2SVP
Def2SVPD
Def2TZVP
Def2TZVPD
cc-pVDZ
aug-cc-pVDZ
cc-pVTZ
aug-cc-pVTZ
Basis set
CCSD
CASSCF(8,8)
MP2
LSDA
PBE
PBE0
B3LYP
CAM-B3LYP
wB97XD
M06-2X
SOGGA11X
M11
MN15

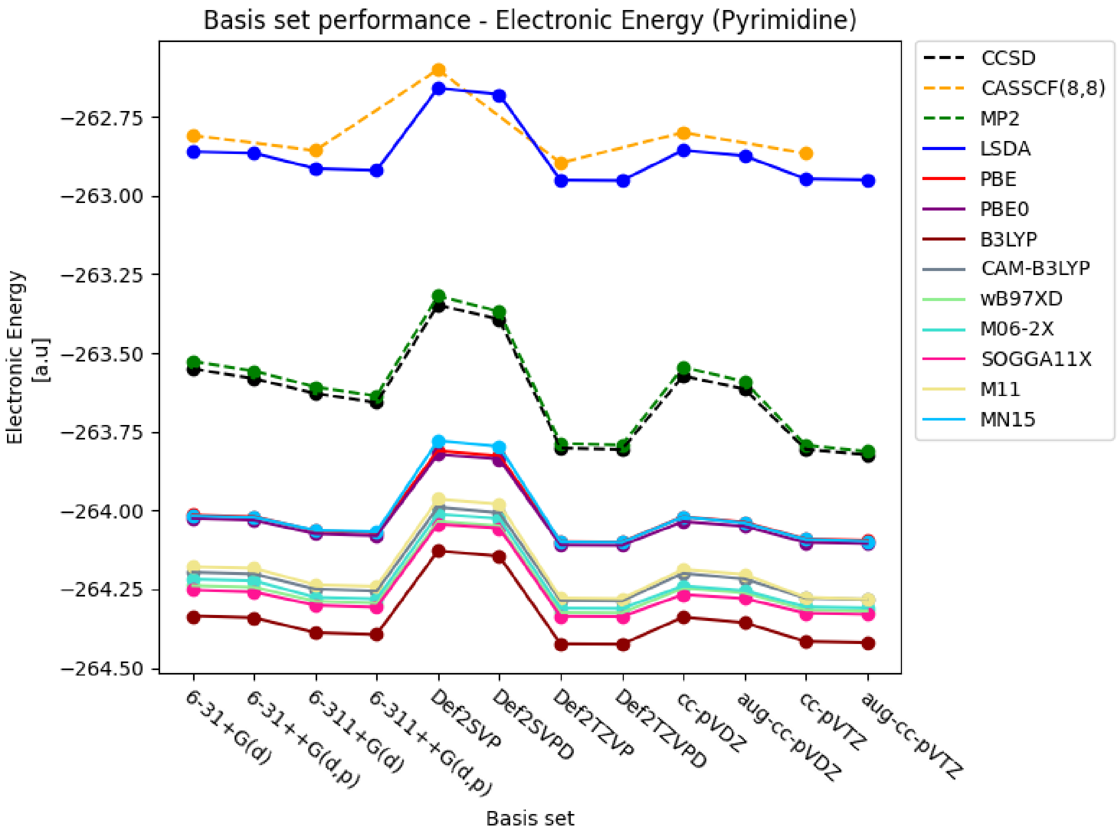

Basis set performance - Electronic Energy (Pyrimidine)
Electronic Energy [a.u]
−262.75
−263.00
−263.25
−263.50
−263.75
−264.00
−264.25
−264.50
6-31+G(d)
6-31++G(d,p)
6-311+G(d)
6-311++G(d,p)
Def2SVP
Def2SVPD
Def2TZVP
Def2TZVPD
cc-pVDZ
aug-cc-pVDZ
cc-pVTZ
aug-cc-pVTZ
Basis set
CCSD
CASSCF(8,8)
MP2
LSDA
PBE
PBE0
B3LYP
CAM-B3LYP
wB97XD
M06-2X
SOGGA11X
M11
MN15

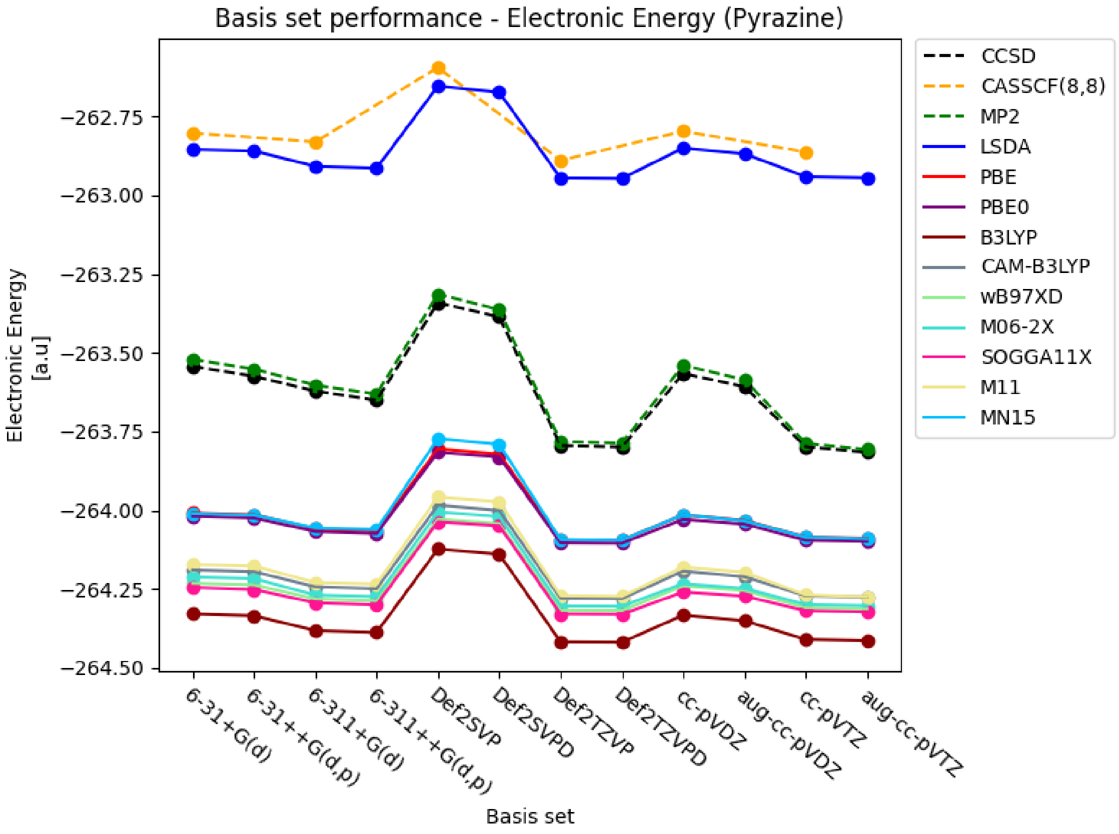

Basis set performance - Electronic Energy (Pyrazine)
Electronic Energy [a.u]
−262.75
−263.00
−263.25
−263.50
−263.75
−264.00
−264.25
−264.50
6-31+G(d)
6-31++G(d,p)
6-311+G(d)
6-311++G(d,p)
Def2SVP
Def2SVPD
Def2TZVP
Def2TZVPD
cc-pVDZ
aug-cc-pVDZ
cc-pVTZ
aug-cc-pVTZ
Basis set
CCSD
CASSCF(8,8)
MP2
LSDA
PBE
PBE0
B3LYP
CAM-B3LYP
wB97XD
M06-2X
SOGGA11X
M11
MN15

C.7 Zero Point Energy

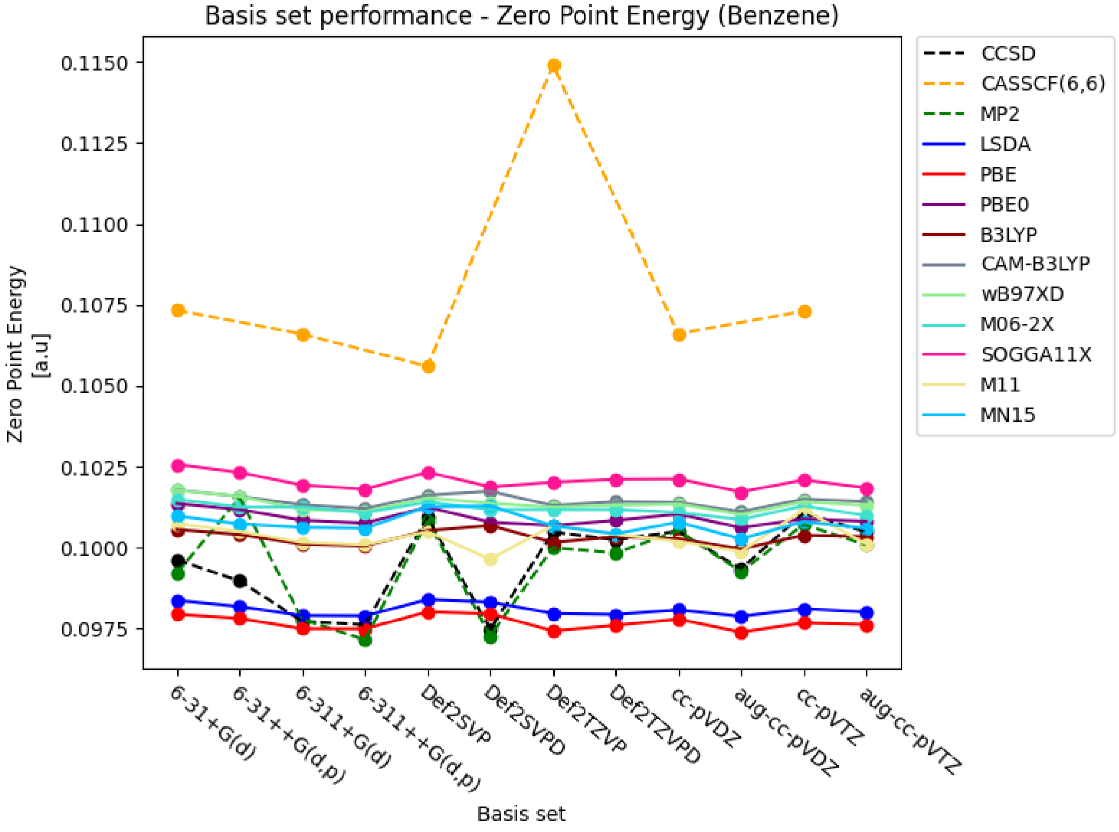

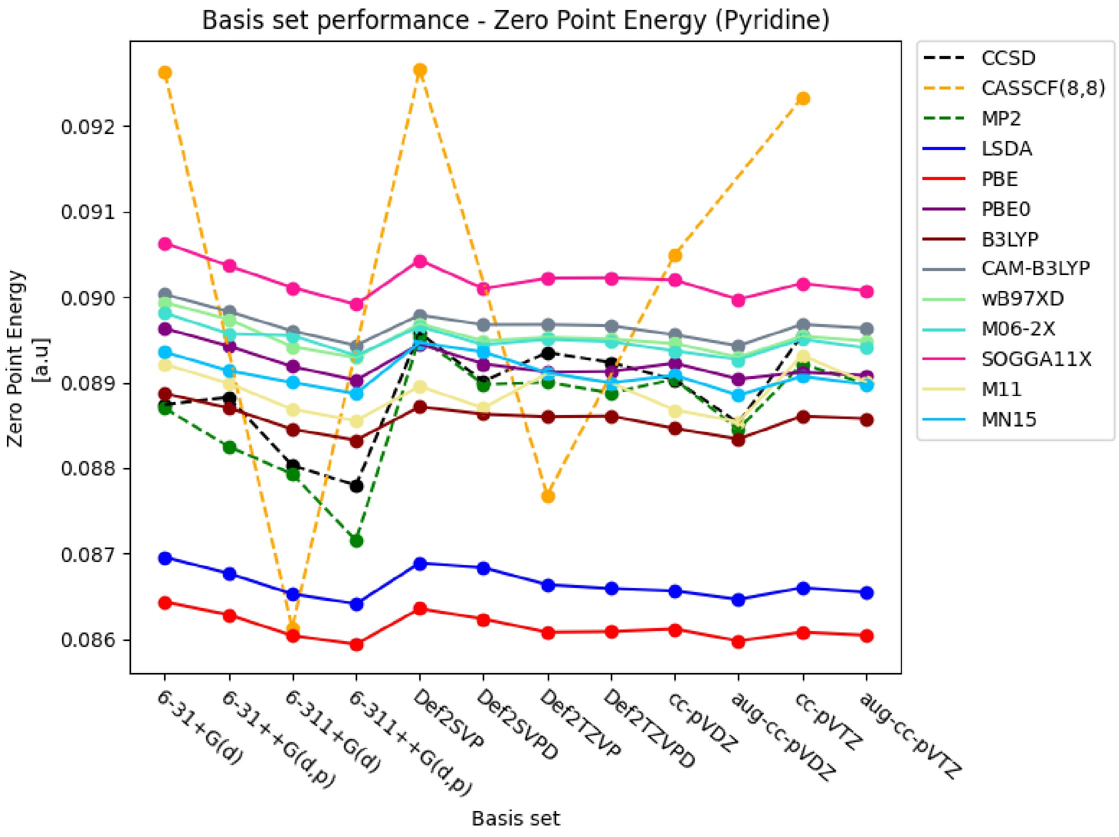
Basis set performance - Zero Point Energy (Pyridine)
Zero Point Energy [a.u]
0.092
0.091
0.090
0.089
0.088
0.087
0.086
Basis set
6-31+G(d)
6-31++G(d,p)
6-311+G(d)
6-311++G(d,p)
Def2SVP
Def2SVPD
Def2TZVP
Def2TZVPD
cc-pVDZ
aug-cc-pVDZ
cc-pVTZ
aug-cc-pVTZ
CCSD
CASSCF(8,8)
MP2
LSDA
PBE
PBE0
B3LYP
CAM-B3LYP
wB97XD
M06-2X
SOGGA11X
M11
MN15

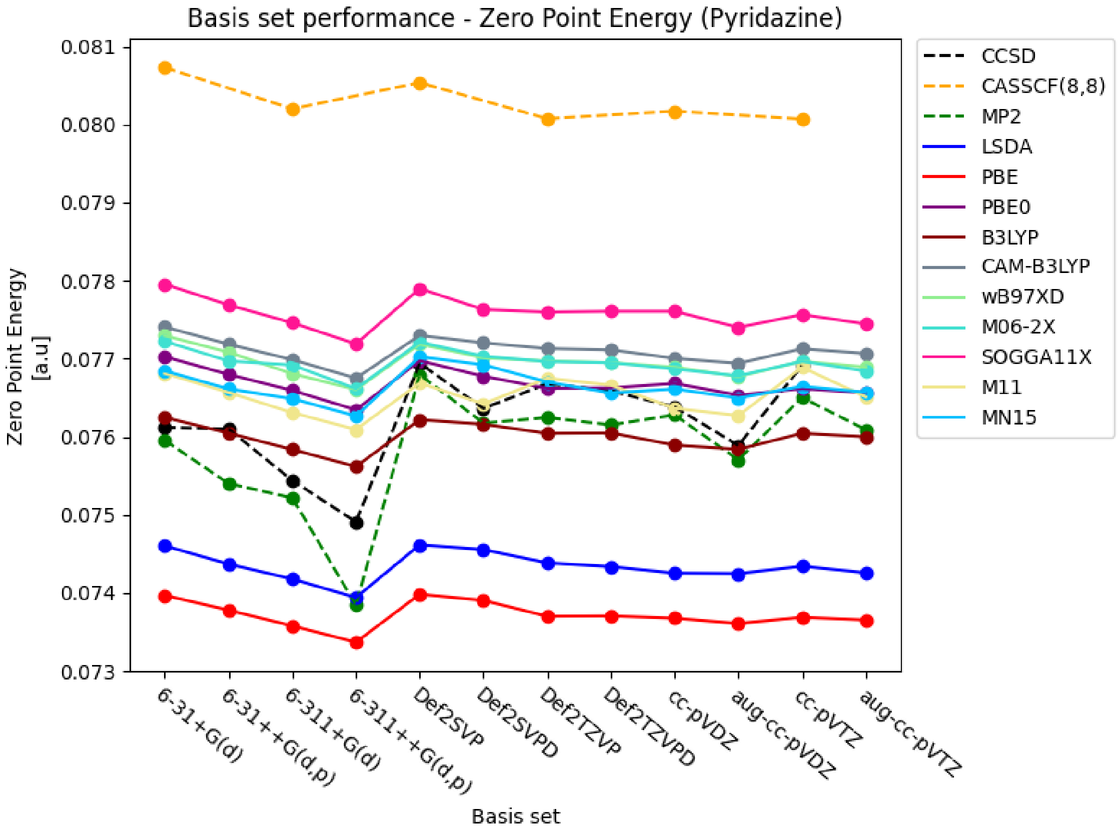
Basis set performance - Zero Point Energy (Pyridazine)
Zero Point Energy [a.u]
0.081
0.080
0.079
0.078
0.077
0.076
0.075
0.074
0.073
Basis set
6-31+G(d)
6-31++G(d,p)
6-311+G(d)
6-311++G(d,p)
Def2SVP
Def2SVPD
Def2TZVP
Def2TZVPD
cc-pVDZ
aug-cc-pVDZ
cc-pVTZ
aug-cc-pVTZ
CCSD
CASSCF(8,8)
MP2
LSDA
PBE
PBE0
B3LYP
CAM-B3LYP
wB97XD
M06-2X
SOGGA11X
M11
MN15

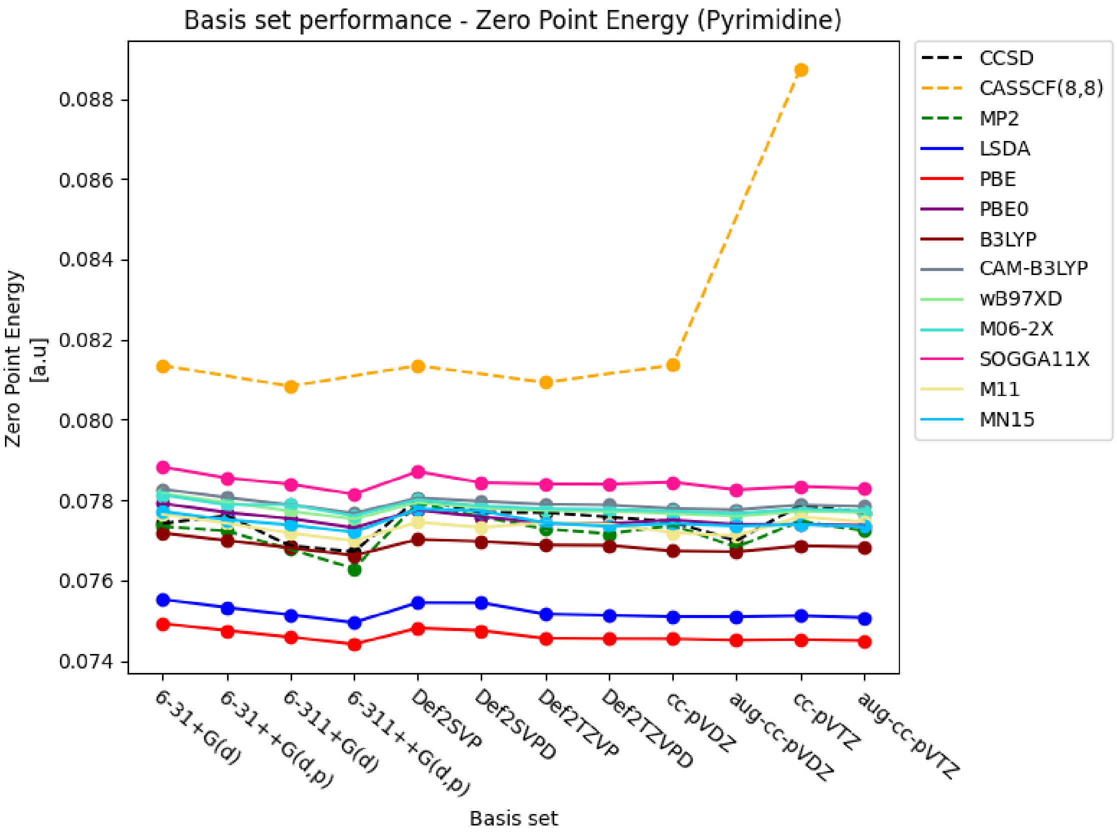
Basis set performance - Zero Point Energy (Pyrimidine)
Zero Point Energy [a.u]
Basis set
0.074
0.076
0.078
0.080
0.082
0.084
0.086
0.088
6-31+G(d)
6-31++G(d,p)
6-311+G(d)
6-311++G(d,p)
Def2SVP
Def2SVPD
Def2TZVP
Def2TZVPD
cc-pVDZ
aug-cc-pVDZ
cc-pVTZ
aug-cc-pVTZ
CCSD
CASSCF(8,8)
MP2
LSDA
PBE
PBE0
B3LYP
CAM-B3LYP
wB97XD
M06-2X
SOGGA11X
M11
MN15

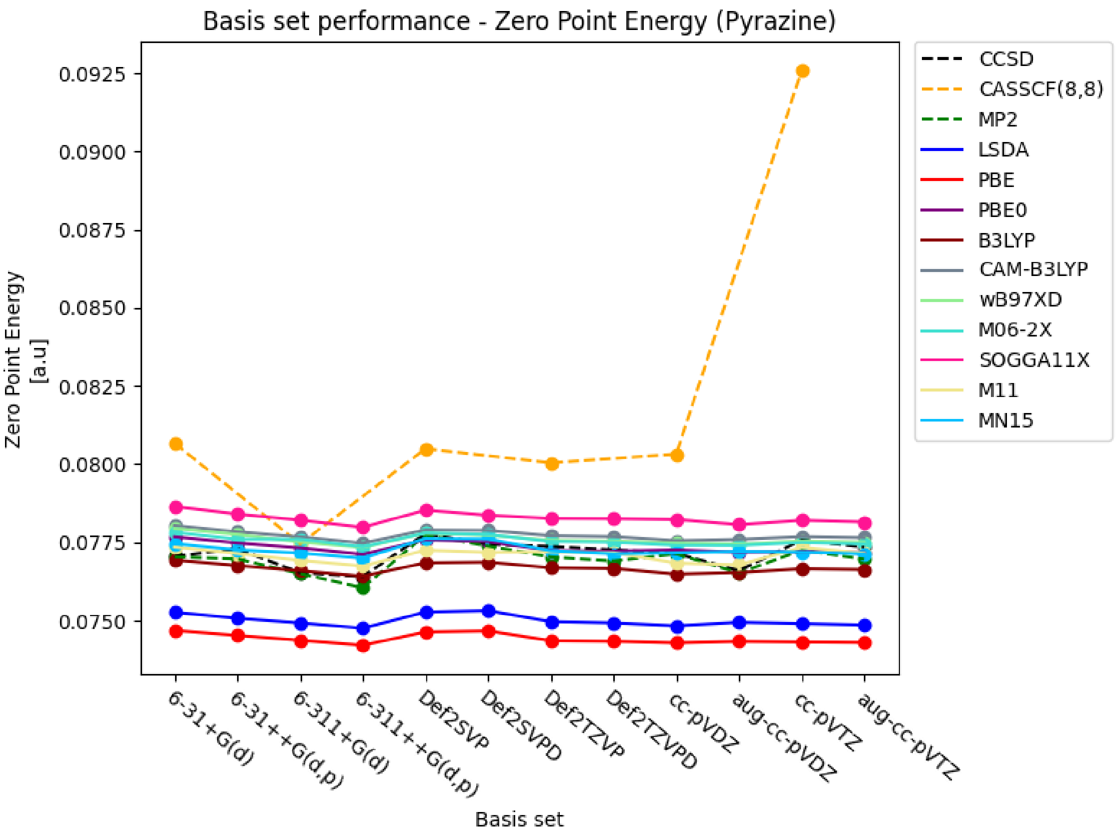
Basis set performance - Zero Point Energy (Pyrazine)
Zero Point Energy [a.u]
Basis set
0.0750
0.0775
0.0800
0.0825
0.0850
0.0875
0.0900
0.0925
6-31+G(d)
6-31++G(d,p)
6-311+G(d)
6-311++G(d,p)
Def2SVP
Def2SVPD
Def2TZVP
Def2TZVPD
cc-pVDZ
aug-cc-pVDZ
cc-pVTZ
aug-cc-pVTZ
CCSD
CASSCF(8,8)
MP2
LSDA
PBE
PBE0
B3LYP
CAM-B3LYP
wB97XD
M06-2X
SOGGA11X
M11
MN15

C.8 Sum of Electronic Energy and ZPE

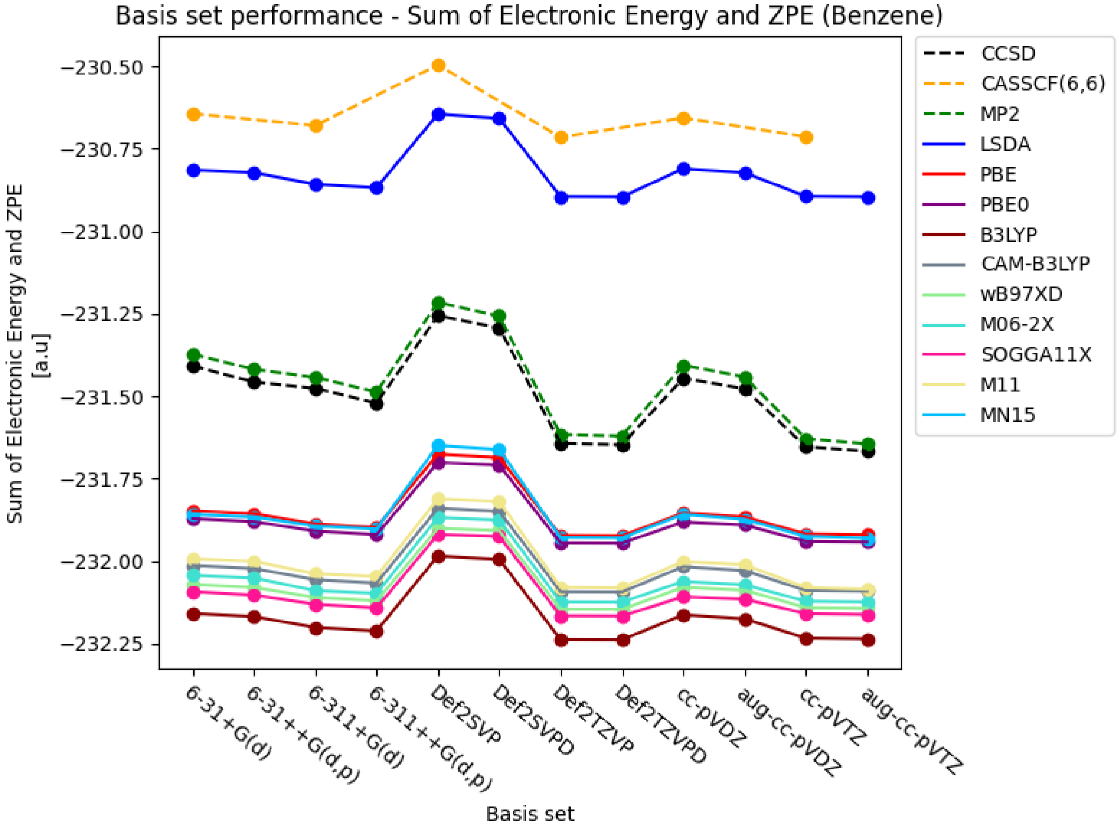

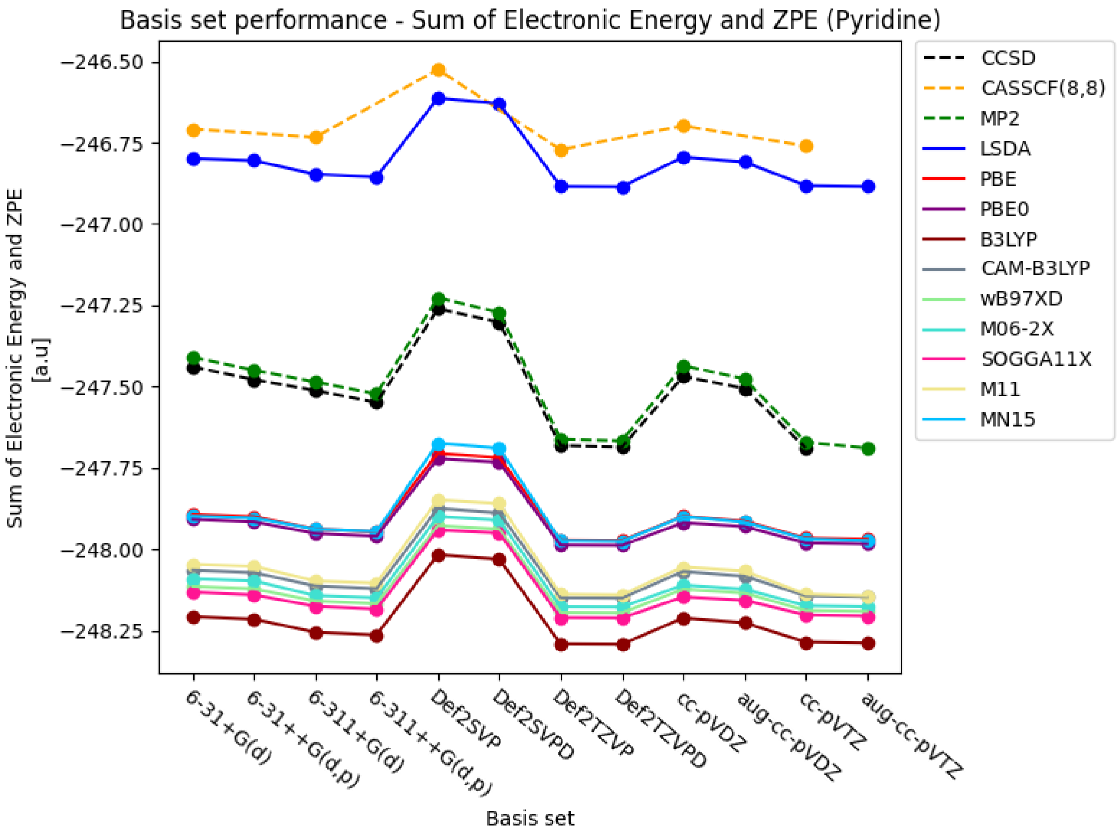
Basis set performance - Sum of Electronic Energy and ZPE (Pyridine)
Sum of Electronic Energy and ZPE [a.u]
Basis set
−246.50
−246.75
−247.00
−247.25
−247.50
−247.75
−248.00
−248.25
6-31+G(d)
6-31++G(d,p)
6-311+G(d)
6-311++G(d,p)
Def2SVP
Def2SVPD
Def2TZVP
Def2TZVPD
cc-pVDZ
aug-cc-pVDZ
cc-pVTZ
aug-cc-pVTZ
CCSD
CASSCF(8,8)
MP2
LSDA
PBE
PBE0
B3LYP
CAM-B3LYP
wB97XD
M06-2X
SOGGA11X
M11
MN15

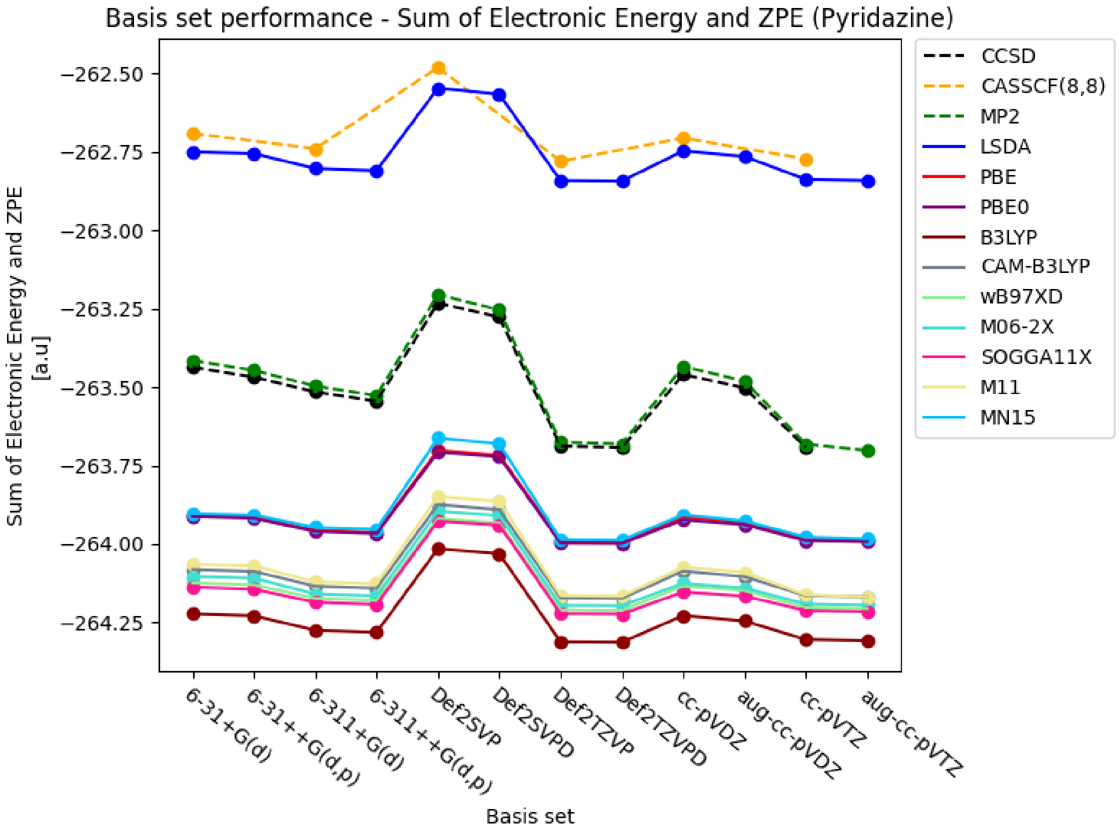
Basis set performance - Sum of Electronic Energy and ZPE (Pyridazine)
Sum of Electronic Energy and ZPE [a.u]
Basis set
−262.50
−262.75
−263.00
−263.25
−263.50
−263.75
−264.00
−264.25
6-31+G(d)
6-31++G(d,p)
6-311+G(d)
6-311++G(d,p)
Def2SVP
Def2SVPD
Def2TZVP
Def2TZVPD
cc-pVDZ
aug-cc-pVDZ
cc-pVTZ
aug-cc-pVTZ
CCSD
CASSCF(8,8)
MP2
LSDA
PBE
PBE0
B3LYP
CAM-B3LYP
wB97XD
M06-2X
SOGGA11X
M11
MN15

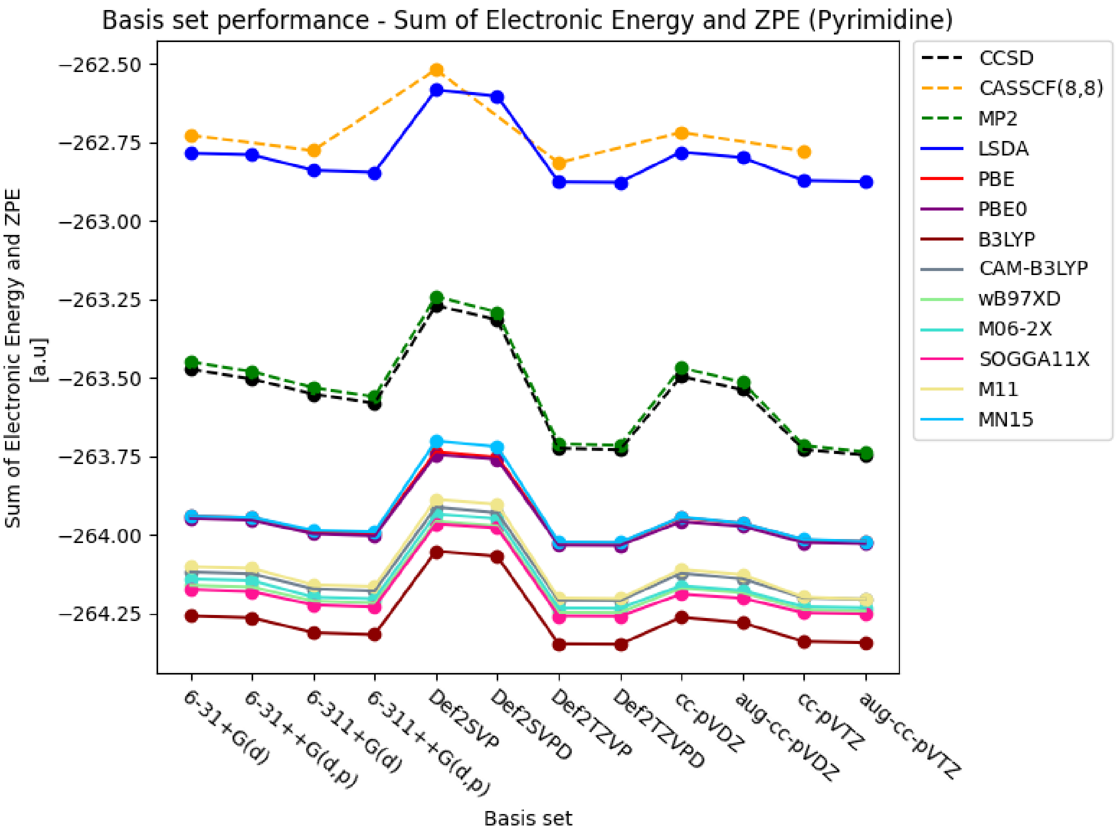

Basis set performance - Sum of Electronic Energy and ZPE (Pyrimidine)
Sum of Electronic Energy and ZPE [a.u]
−262.50
−262.75
−263.00
−263.25
−263.50
−263.75
−264.00
−264.25
6-31+G(d)
6-31++G(d,p)
6-311+G(d)
6-311++G(d,p)
Def2SVP
Def2SVPD
Def2TZVP
Def2TZVPD
cc-pVDZ
aug-cc-pVDZ
cc-pVTZ
aug-cc-pVTZ
Basis set
CCSD
CASSCF(8,8)
MP2
LSDA
PBE
PBE0
B3LYP
CAM-B3LYP
wB97XD
M06-2X
SOGGA11X
M11
MN15

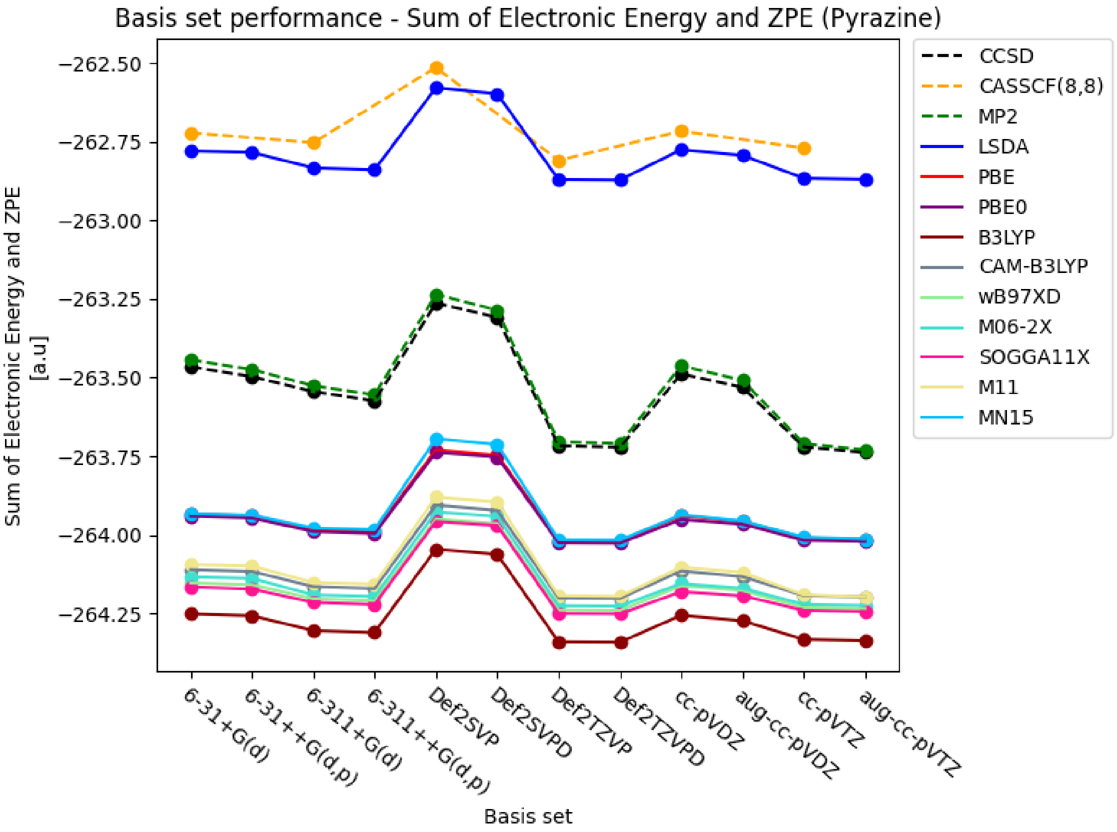

Basis set performance - Sum of Electronic Energy and ZPE (Pyrazine)
Sum of Electronic Energy and ZPE [a.u]
−262.50
−262.75
−263.00
−263.25
−263.50
−263.75
−264.00
−264.25
6-31+G(d)
6-31++G(d,p)
6-311+G(d)
6-311++G(d,p)
Def2SVP
Def2SVPD
Def2TZVP
Def2TZVPD
cc-pVDZ
aug-cc-pVDZ
cc-pVTZ
aug-cc-pVTZ
Basis set
CCSD
CASSCF(8,8)
MP2
LSDA
PBE
PBE0
B3LYP
CAM-B3LYP
wB97XD
M06-2X
SOGGA11X
M11
MN15

D Benchmark Plots

All figures with relative errors from the 'Benchmarking of DFT Functionals' section are listed in the following. They are arranged similar as the basis set performance plots from the previous section; the relative errors for all five molecules are listed together for each property. The relative differences are calculated with respect to the corresponding property obtained at CCSD level of theory. The electronic structure methods are listed along the horizontal axis, and the basis sets are listed along the vertical axis. The associated molecule is specified in parentheses on top of each figure. The colorbar indicates the size of the relative error; the color red indicates an overestimation of the property, whereas blue indicates an underestimation.

D.1 HOMA Index

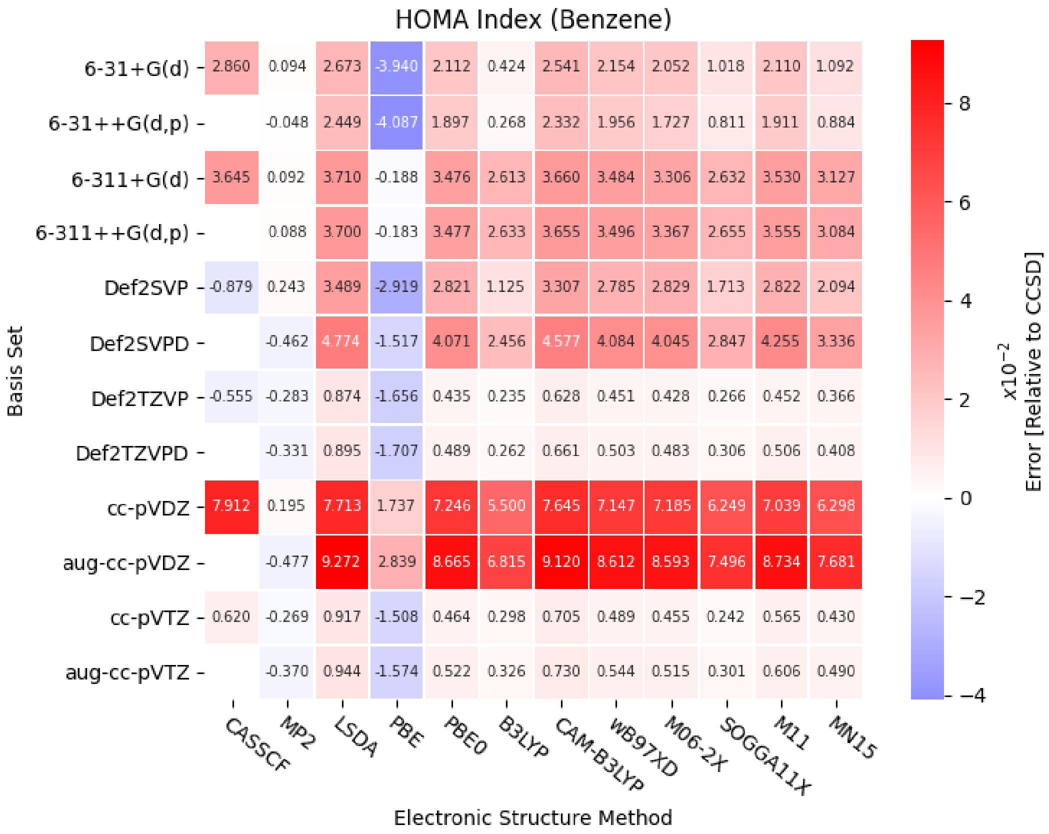

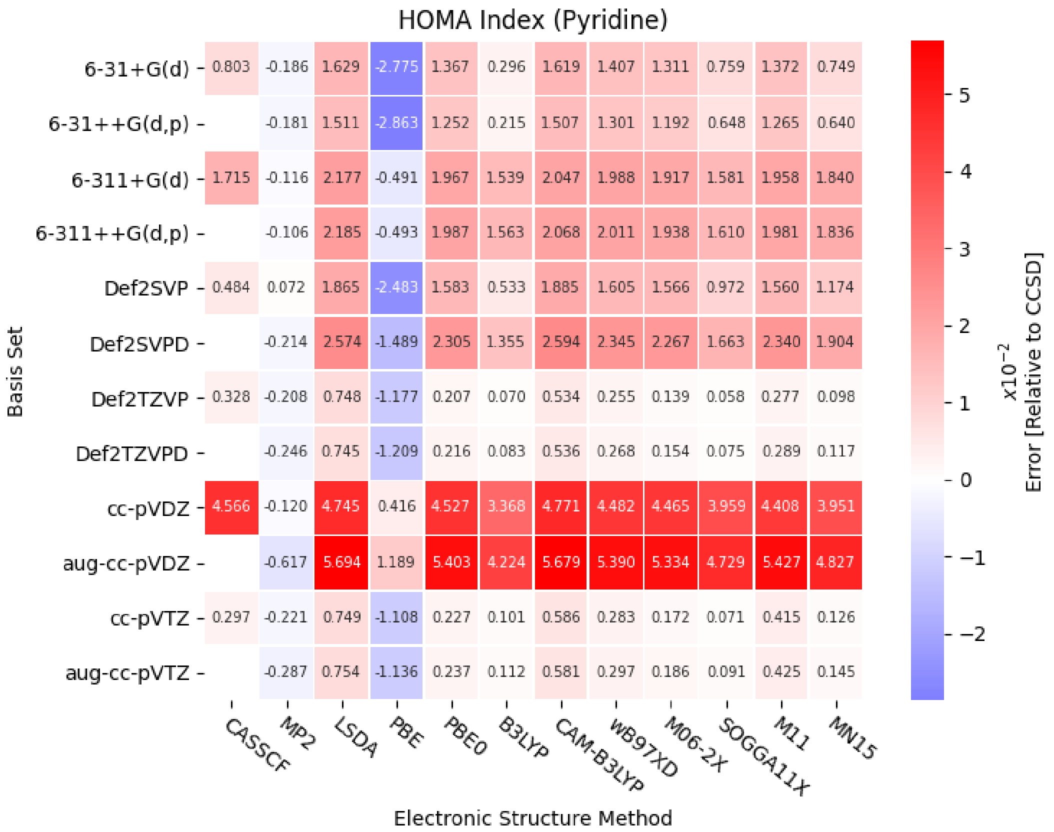
HOMA Index (Pyridine)
Basis Set
Electronic Structure Method
x10^−2 Error [Relative to CCSD]
CASSCF MP2 LSDA PBE PBE0 B3LYP CAM-B3LYP wB97XD M06-2X SOGGA11X M11 MN15
6-31+G(d) 0.803 -0.186 1.629 -2.775 1.367 0.296 1.619 1.407 1.311 0.759 1.372 0.749
6-31++G(d,p) -0.181 1.511 -2.863 1.252 0.215 1.507 1.301 1.192 0.648 1.265 0.640
6-311+G(d) 1.715 -0.116 2.177 -0.491 1.967 1.539 2.047 1.988 1.917 1.581 1.958 1.840
6-311++G(d,p) -0.106 2.185 -0.493 1.987 1.563 2.068 2.011 1.938 1.610 1.981 1.836
Def2SVP 0.484 0.072 1.865 -2.483 1.583 0.533 1.885 1.605 1.566 0.972 1.560 1.174
Def2SVPD -0.214 2.574 -1.489 2.305 1.355 2.594 2.345 2.267 1.663 2.340 1.904
Def2TZVP 0.328 -0.208 0.748 -1.177 0.207 0.070 0.534 0.255 0.139 0.058 0.277 0.098
Def2TZVPD -0.246 0.745 -1.209 0.216 0.083 0.536 0.268 0.154 0.075 0.289 0.117
cc-pVDZ 4.566 -0.120 4.745 0.416 4.527 3.368 4.771 4.482 4.465 3.959 4.408 3.951
aug-cc-pVDZ -0.617 5.694 1.189 5.403 4.224 5.679 5.390 5.334 4.729 5.427 4.827
cc-pVTZ 0.297 -0.221 0.749 -1.108 0.227 0.101 0.586 0.283 0.172 0.071 0.415 0.126
aug-cc-pVTZ -0.287 0.754 -1.136 0.237 0.112 0.581 0.297 0.186 0.091 0.425 0.145
5 4 3 2 1 0 −1 −2

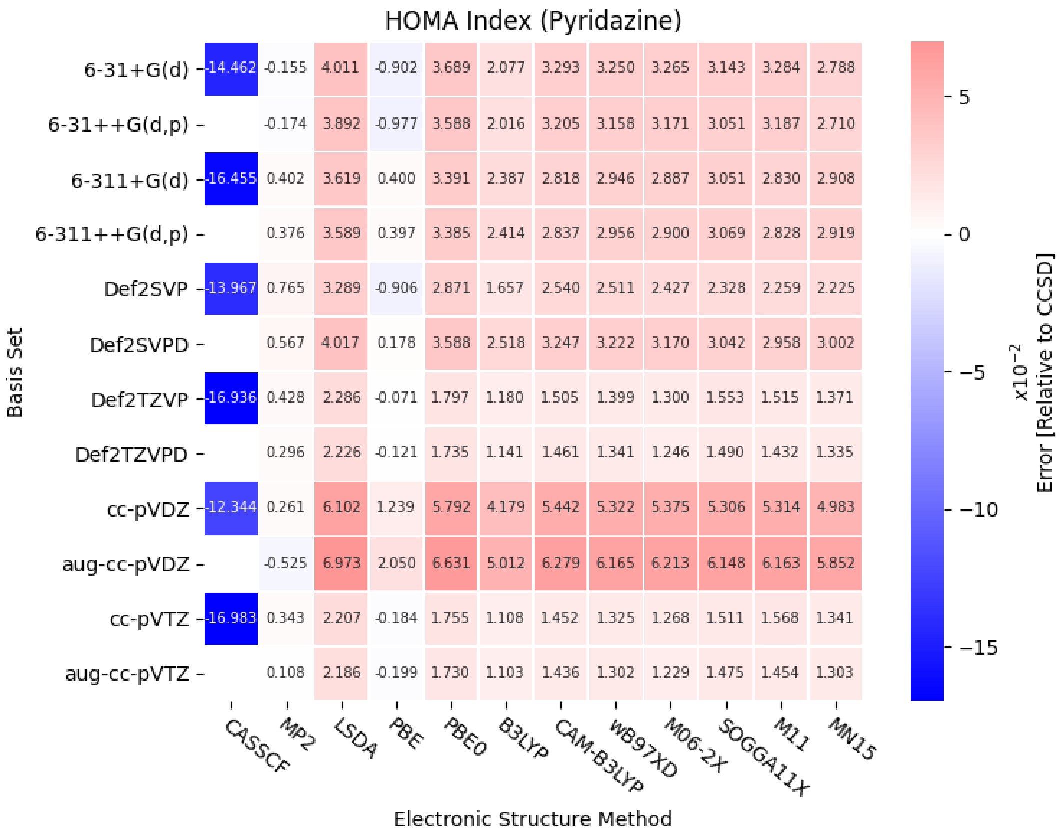
HOMA Index (Pyridazine)
Basis Set
Electronic Structure Method
x10^−2 Error [Relative to CCSD]
CASSCF MP2 LSDA PBE PBE0 B3LYP CAM-B3LYP wB97XD M06-2X SOGGA11X M11 MN15
6-31+G(d) -14.462 -0.155 4.011 -0.902 3.689 2.077 3.293 3.250 3.265 3.143 3.284 2.788
6-31++G(d,p) -0.174 3.892 -0.977 3.588 2.016 3.205 3.158 3.171 3.051 3.187 2.710
6-311+G(d) -16.455 0.402 3.619 0.400 3.391 2.387 2.818 2.946 2.887 3.051 2.830 2.908
6-311++G(d,p) 0.376 3.589 0.397 3.385 2.414 2.837 2.956 2.900 3.069 2.828 2.919
Def2SVP -13.967 0.765 3.289 -0.906 2.871 1.657 2.540 2.511 2.427 2.328 2.259 2.225
Def2SVPD 0.567 4.017 0.178 3.588 2.518 3.247 3.222 3.170 3.042 2.958 3.002
Def2TZVP -16.936 0.428 2.286 -0.071 1.797 1.180 1.505 1.399 1.300 1.553 1.515 1.371
Def2TZVPD 0.296 2.226 -0.121 1.735 1.141 1.461 1.341 1.246 1.490 1.432 1.335
cc-pVDZ -12.344 0.261 6.102 1.239 5.792 4.179 5.442 5.322 5.375 5.306 5.314 4.983
aug-cc-pVDZ -0.525 6.973 2.050 6.631 5.012 6.279 6.165 6.213 6.148 6.163 5.852
cc-pVTZ -16.983 0.343 2.207 -0.184 1.755 1.108 1.452 1.325 1.268 1.511 1.568 1.341
aug-cc-pVTZ 0.108 2.186 -0.199 1.730 1.103 1.436 1.302 1.229 1.475 1.454 1.303
5 0 −5 −10 −15

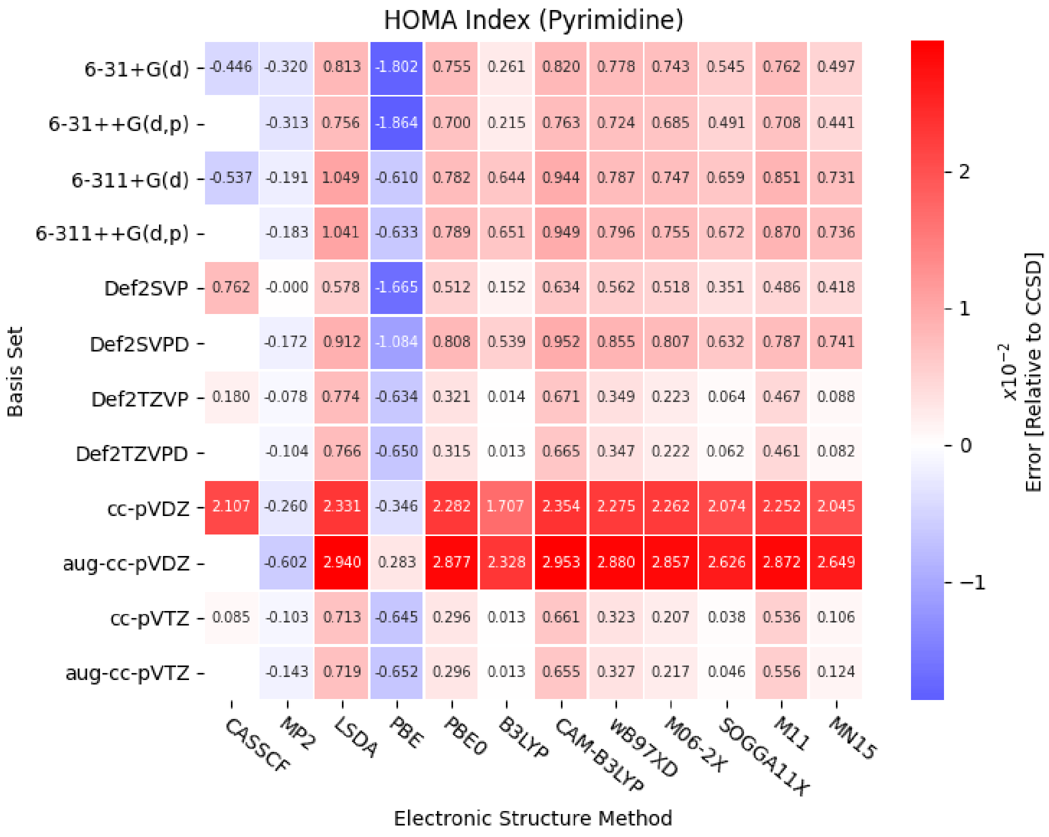
HOMA Index (Pyrimidine)
Basis Set
Electronic Structure Method
CASSCF MP2 LSDA PBE PBE0 B3LYP CAM-B3LYP wB97XD M06-2X SOGGA11X M11 MN15
6-31+G(d) -0.446 -0.320 0.813 -1.802 0.755 0.261 0.820 0.778 0.743 0.545 0.762 0.497
6-31++G(d,p) -0.313 0.756 -1.864 0.700 0.215 0.763 0.724 0.685 0.491 0.708 0.441
6-311+G(d) -0.537 -0.191 1.049 -0.610 0.782 0.644 0.944 0.787 0.747 0.659 0.851 0.731
6-311++G(d,p) -0.183 1.041 -0.633 0.789 0.651 0.949 0.796 0.755 0.672 0.870 0.736
Def2SVP 0.762 -0.000 0.578 -1.665 0.512 0.152 0.634 0.562 0.518 0.351 0.486 0.418
Def2SVPD -0.172 0.912 -1.084 0.808 0.539 0.952 0.855 0.807 0.632 0.787 0.741
Def2TZVP 0.180 -0.078 0.774 -0.634 0.321 0.014 0.671 0.349 0.223 0.064 0.467 0.088
Def2TZVPD -0.104 0.766 -0.650 0.315 0.013 0.665 0.347 0.222 0.062 0.461 0.082
cc-pVDZ 2.107 -0.260 2.331 -0.346 2.282 1.707 2.354 2.275 2.262 2.074 2.252 2.045
aug-cc-pVDZ -0.602 2.940 0.283 2.877 2.328 2.953 2.880 2.857 2.626 2.872 2.649
cc-pVTZ 0.085 -0.103 0.713 -0.645 0.296 0.013 0.661 0.323 0.207 0.038 0.536 0.106
aug-cc-pVTZ -0.143 0.719 -0.652 0.296 0.013 0.655 0.327 0.217 0.046 0.556 0.124
2
1
0
−1
$x10^{-2}$
Error [Relative to CCSD]

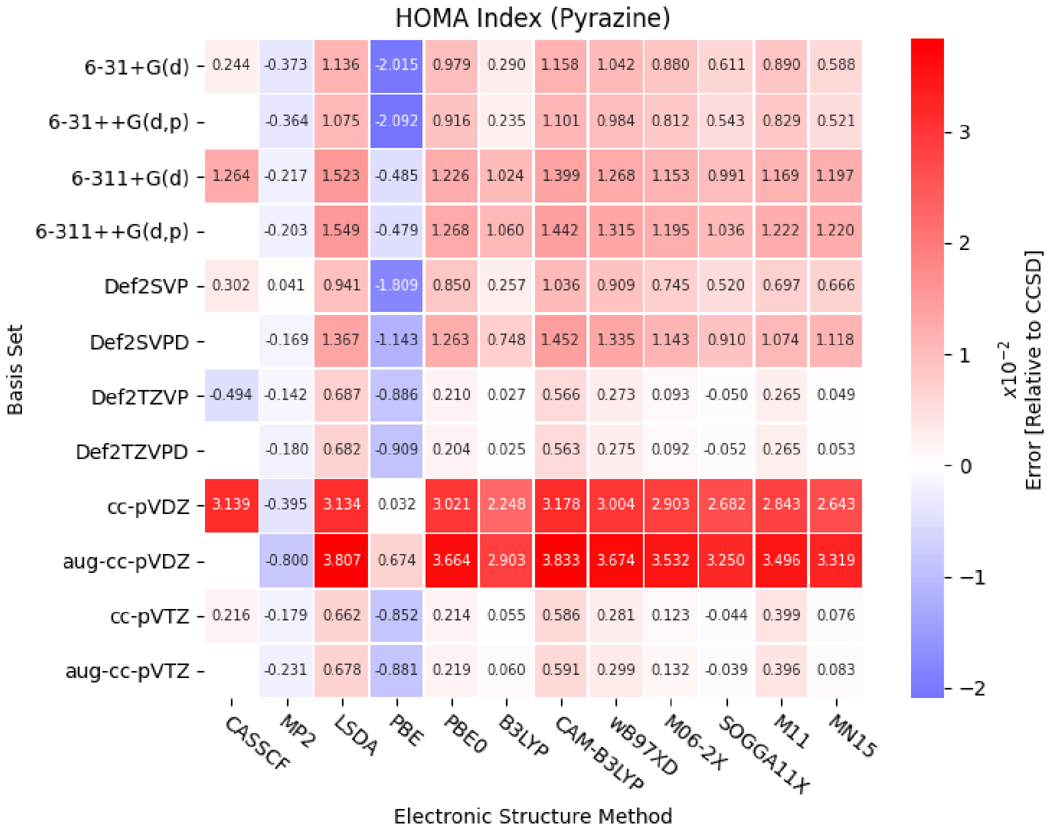
HOMA Index (Pyrazine)
Basis Set
Electronic Structure Method
CASSCF MP2 LSDA PBE PBE0 B3LYP CAM-B3LYP wB97XD M06-2X SOGGA11X M11 MN15
6-31+G(d) 0.244 -0.373 1.136 -2.015 0.979 0.290 1.158 1.042 0.880 0.611 0.890 0.588
6-31++G(d,p) -0.364 1.075 -2.092 0.916 0.235 1.101 0.984 0.812 0.543 0.829 0.521
6-311+G(d) 1.264 -0.217 1.523 -0.485 1.226 1.024 1.399 1.268 1.153 0.991 1.169 1.197
6-311++G(d,p) -0.203 1.549 -0.479 1.268 1.060 1.442 1.315 1.195 1.036 1.222 1.220
Def2SVP 0.302 0.041 0.941 -1.809 0.850 0.257 1.036 0.909 0.745 0.520 0.697 0.666
Def2SVPD -0.169 1.367 -1.143 1.263 0.748 1.452 1.335 1.143 0.910 1.074 1.118
Def2TZVP -0.494 -0.142 0.687 -0.886 0.210 0.027 0.566 0.273 0.093 -0.050 0.265 0.049
Def2TZVPD -0.180 0.682 -0.909 0.204 0.025 0.563 0.275 0.092 -0.052 0.265 0.053
cc-pVDZ 3.139 -0.395 3.134 0.032 3.021 2.248 3.178 3.004 2.903 2.682 2.843 2.643
aug-cc-pVDZ -0.800 3.807 0.674 3.664 2.903 3.833 3.674 3.532 3.250 3.496 3.319
cc-pVTZ 0.216 -0.179 0.662 -0.852 0.214 0.055 0.586 0.281 0.123 -0.044 0.399 0.076
aug-cc-pVTZ -0.231 0.678 -0.881 0.219 0.060 0.591 0.299 0.132 -0.039 0.396 0.083
3
2
1
0
−1
−2
$x10^{-2}$
Error [Relative to CCSD]

D.2 PDI

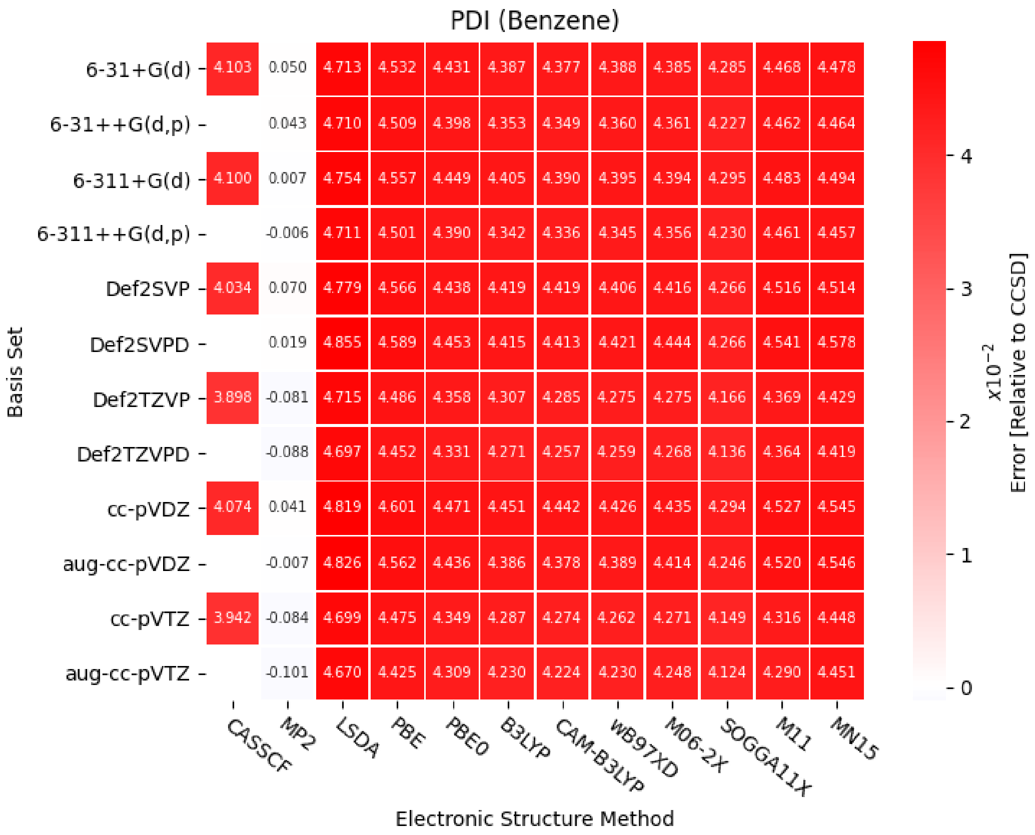
PDI (Benzene)
Basis Set
Electronic Structure Method
x10^-2 Error [Relative to CCSD]
6-31+G(d)
6-31++G(d,p)
6-311+G(d)
6-311++G(d,p)
Def2SVP
Def2SVPD
Def2TZVP
Def2TZVPD
cc-pVDZ
aug-cc-pVDZ
cc-pVTZ
aug-cc-pVTZ
CASSCF
MP2
LSDA
PBE
PBE0
B3LYP
CAM-B3LYP
wB97XD
M06-2X
SOGGA11X
M11
MN15

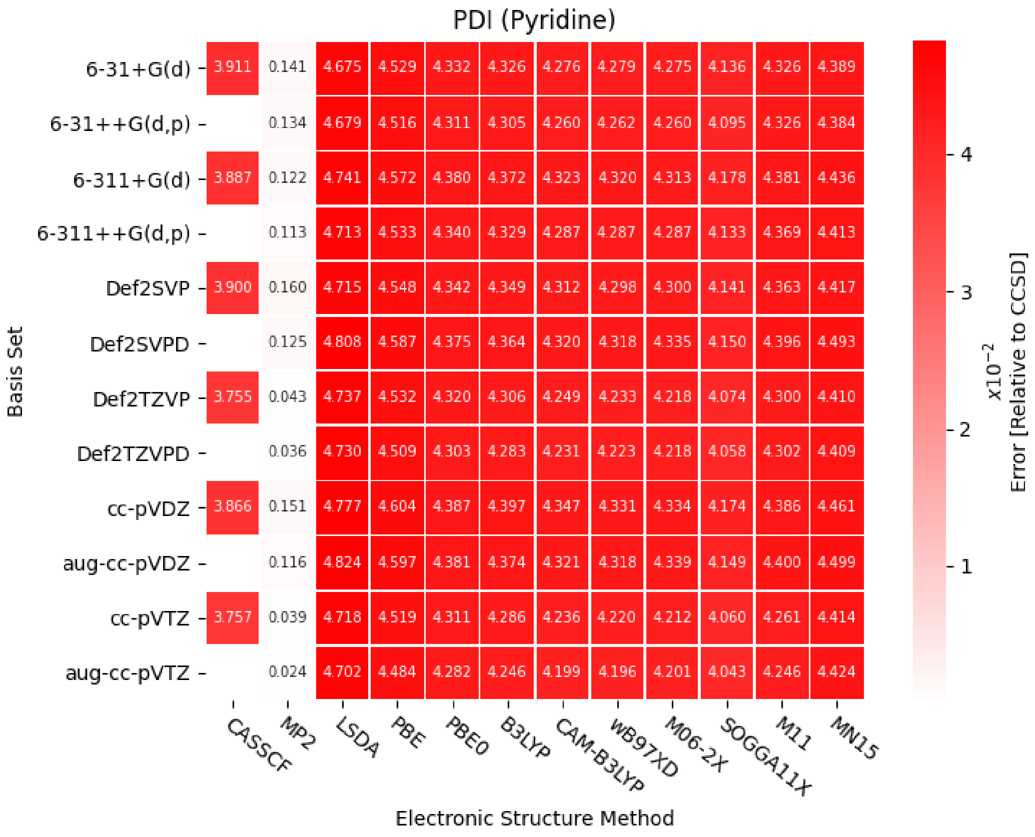
PDI (Pyridine)
Basis Set
6-31+G(d) 3.911 0.141 4.675 4.529 4.332 4.326 4.276 4.279 4.275 4.136 4.326 4.389
6-31++G(d,p) 0.134 4.679 4.516 4.311 4.305 4.260 4.262 4.260 4.095 4.326 4.384
6-311+G(d) 3.887 0.122 4.741 4.572 4.380 4.372 4.323 4.320 4.313 4.178 4.381 4.436
6-311++G(d,p) 0.113 4.713 4.533 4.340 4.329 4.287 4.287 4.287 4.133 4.369 4.413
Def2SVP 3.900 0.160 4.715 4.548 4.342 4.349 4.312 4.298 4.300 4.141 4.363 4.417
Def2SVPD 0.125 4.808 4.587 4.375 4.364 4.320 4.318 4.335 4.150 4.396 4.493
Def2TZVP 3.755 0.043 4.737 4.532 4.320 4.306 4.249 4.233 4.218 4.074 4.300 4.410
Def2TZVPD 0.036 4.730 4.509 4.303 4.283 4.231 4.223 4.218 4.058 4.302 4.409
cc-pVDZ 3.866 0.151 4.777 4.604 4.387 4.397 4.347 4.331 4.334 4.174 4.386 4.461
aug-cc-pVDZ 0.116 4.824 4.597 4.381 4.374 4.321 4.318 4.339 4.149 4.400 4.499
cc-pVTZ 3.757 0.039 4.718 4.519 4.311 4.286 4.236 4.220 4.212 4.060 4.261 4.414
aug-cc-pVTZ 0.024 4.702 4.484 4.282 4.246 4.199 4.196 4.201 4.043 4.246 4.424
CASSCF MP2 LSDA PBE PBE0 B3LYP CAM-B3LYP wB97XD M06-2X SOGGA11X M11 MN15
Electronic Structure Method
1 2 3 4
$x10^{-2}$
Error [Relative to CCSD]

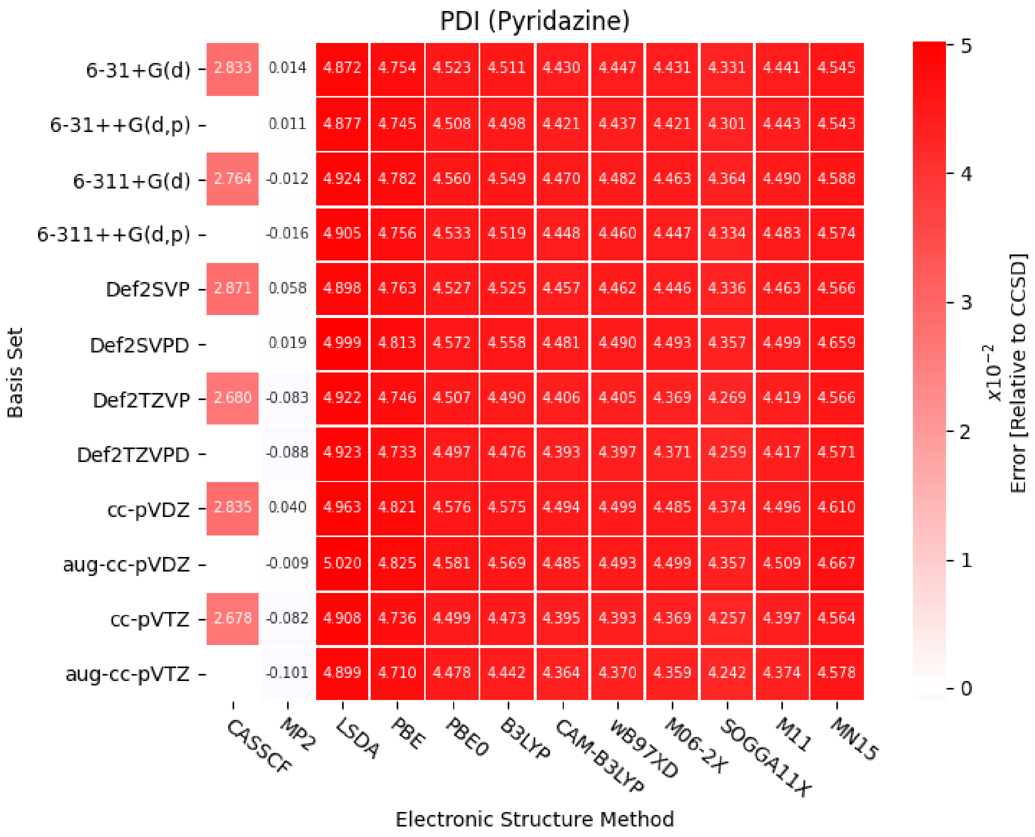
PDI (Pyridazine)
Basis Set
6-31+G(d) 2.833 0.014 4.872 4.754 4.523 4.511 4.430 4.447 4.431 4.331 4.441 4.545
6-31++G(d,p) 0.011 4.877 4.745 4.508 4.498 4.421 4.437 4.421 4.301 4.443 4.543
6-311+G(d) 2.764 -0.012 4.924 4.782 4.560 4.549 4.470 4.482 4.463 4.364 4.490 4.588
6-311++G(d,p) -0.016 4.905 4.756 4.533 4.519 4.448 4.460 4.447 4.334 4.483 4.574
Def2SVP 2.871 0.058 4.898 4.763 4.527 4.525 4.457 4.462 4.446 4.336 4.463 4.566
Def2SVPD 0.019 4.999 4.813 4.572 4.558 4.481 4.490 4.493 4.357 4.499 4.659
Def2TZVP 2.680 -0.083 4.922 4.746 4.507 4.490 4.406 4.405 4.369 4.269 4.419 4.566
Def2TZVPD -0.088 4.923 4.733 4.497 4.476 4.393 4.397 4.371 4.259 4.417 4.571
cc-pVDZ 2.835 0.040 4.963 4.821 4.576 4.575 4.494 4.499 4.485 4.374 4.496 4.610
aug-cc-pVDZ -0.009 5.020 4.825 4.581 4.569 4.485 4.493 4.499 4.357 4.509 4.667
cc-pVTZ 2.678 -0.082 4.908 4.736 4.499 4.473 4.395 4.393 4.369 4.257 4.397 4.564
aug-cc-pVTZ -0.101 4.899 4.710 4.478 4.442 4.364 4.370 4.359 4.242 4.374 4.578
CASSCF MP2 LSDA PBE PBE0 B3LYP CAM-B3LYP wB97XD M06-2X SOGGA11X M11 MN15
Electronic Structure Method
0 1 2 3 4 5
$x10^{-2}$
Error [Relative to CCSD]

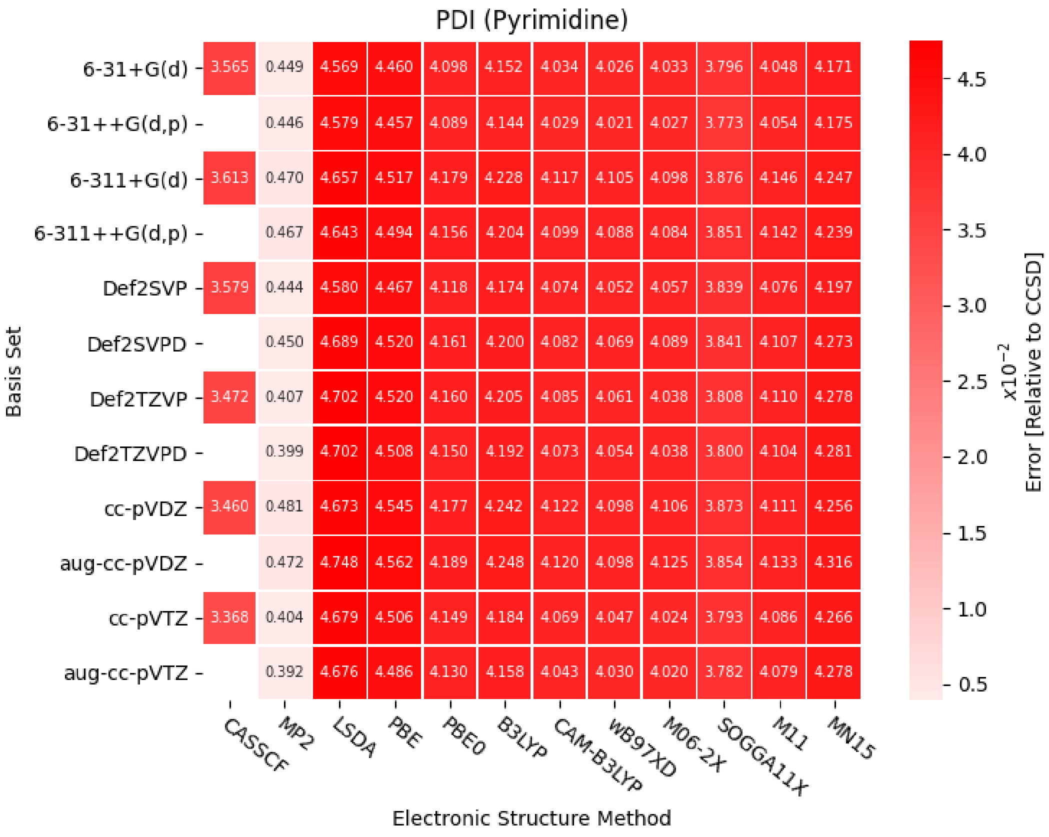
PDI (Pyrimidine)
Basis Set
6-31+G(d) 3.565 0.449 4.569 4.460 4.098 4.152 4.034 4.026 4.033 3.796 4.048 4.171
6-31++G(d,p) 0.446 4.579 4.457 4.089 4.144 4.029 4.021 4.027 3.773 4.054 4.175
6-311+G(d) 3.613 0.470 4.657 4.517 4.179 4.228 4.117 4.105 4.098 3.876 4.146 4.247
6-311++G(d,p) 0.467 4.643 4.494 4.156 4.204 4.099 4.088 4.084 3.851 4.142 4.239
Def2SVP 3.579 0.444 4.580 4.467 4.118 4.174 4.074 4.052 4.057 3.839 4.076 4.197
Def2SVPD 0.450 4.689 4.520 4.161 4.200 4.082 4.069 4.089 3.841 4.107 4.273
Def2TZVP 3.472 0.407 4.702 4.520 4.160 4.205 4.085 4.061 4.038 3.808 4.110 4.278
Def2TZVPD 0.399 4.702 4.508 4.150 4.192 4.073 4.054 4.038 3.800 4.104 4.281
cc-pVDZ 3.460 0.481 4.673 4.545 4.177 4.242 4.122 4.098 4.106 3.873 4.111 4.256
aug-cc-pVDZ 0.472 4.748 4.562 4.189 4.248 4.120 4.098 4.125 3.854 4.133 4.316
cc-pVTZ 3.368 0.404 4.679 4.506 4.149 4.184 4.069 4.047 4.024 3.793 4.086 4.266
aug-cc-pVTZ 0.392 4.676 4.486 4.130 4.158 4.043 4.030 4.020 3.782 4.079 4.278
CASSCF MP2 LSDA PBE PBE0 B3LYP CAM-B3LYP wB97XD M06-2X SOGGA11X M11 MN15
Electronic Structure Method
4.5 4.0 3.5 3.0 2.5 2.0 1.5 1.0 0.5
$x10^{-2}$
Error [Relative to CCSD]

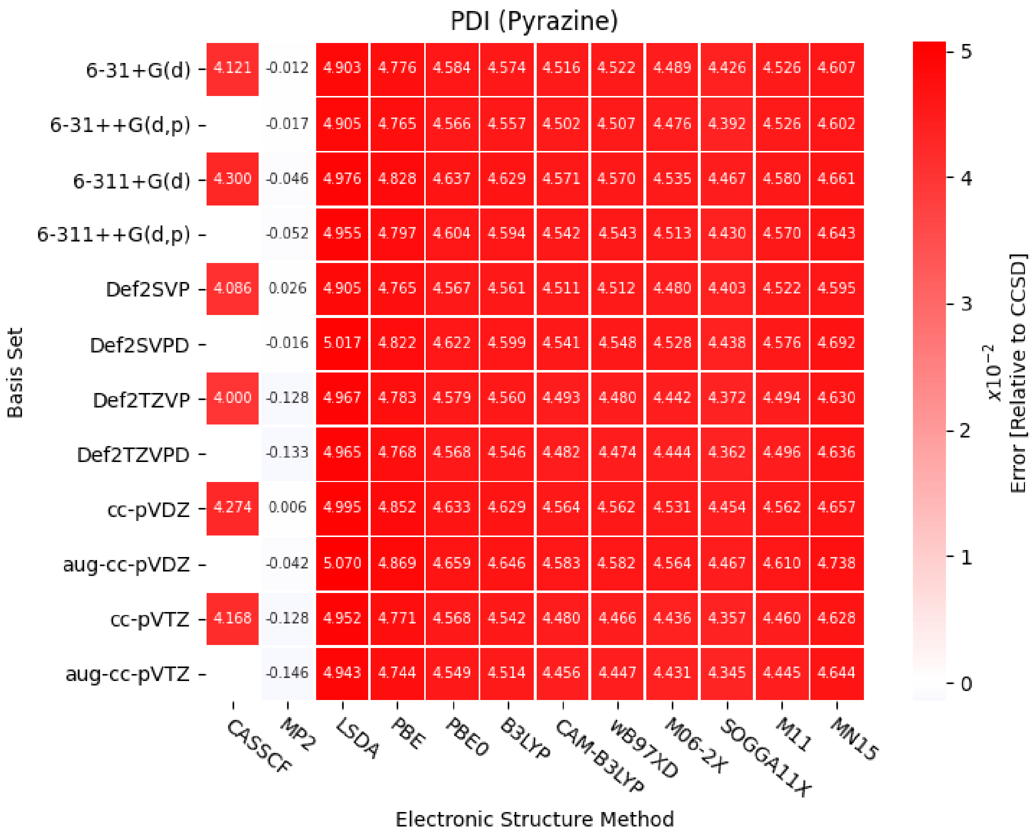
PDI (Pyrazine)
Basis Set
6-31+G(d) 4.121 -0.012 4.903 4.776 4.584 4.574 4.516 4.522 4.489 4.426 4.526 4.607
6-31++G(d,p) -0.017 4.905 4.765 4.566 4.557 4.502 4.507 4.476 4.392 4.526 4.602
6-311+G(d) 4.300 -0.046 4.976 4.828 4.637 4.629 4.571 4.570 4.535 4.467 4.580 4.661
6-311++G(d,p) -0.052 4.955 4.797 4.604 4.594 4.542 4.543 4.513 4.430 4.570 4.643
Def2SVP 4.086 0.026 4.905 4.765 4.567 4.561 4.511 4.512 4.480 4.403 4.522 4.595
Def2SVPD -0.016 5.017 4.822 4.622 4.599 4.541 4.548 4.528 4.438 4.576 4.692
Def2TZVP 4.000 -0.128 4.967 4.783 4.579 4.560 4.493 4.480 4.442 4.372 4.494 4.630
Def2TZVPD -0.133 4.965 4.768 4.568 4.546 4.482 4.474 4.444 4.362 4.496 4.636
cc-pVDZ 4.274 0.006 4.995 4.852 4.633 4.629 4.564 4.562 4.531 4.454 4.562 4.657
aug-cc-pVDZ -0.042 5.070 4.869 4.659 4.646 4.583 4.582 4.564 4.467 4.610 4.738
cc-pVTZ 4.168 -0.128 4.952 4.771 4.568 4.542 4.480 4.466 4.436 4.357 4.460 4.628
aug-cc-pVTZ -0.146 4.943 4.744 4.549 4.514 4.456 4.447 4.431 4.345 4.445 4.644
CASSCF MP2 LSDA PBE PBE0 B3LYP CAM-B3LYP wB97XD M06-2X SOGGA11X M11 MN15
Electronic Structure Method
5 4 3 2 1 0
$x10^{-2}$
Error [Relative to CCSD]

D.3 MCI

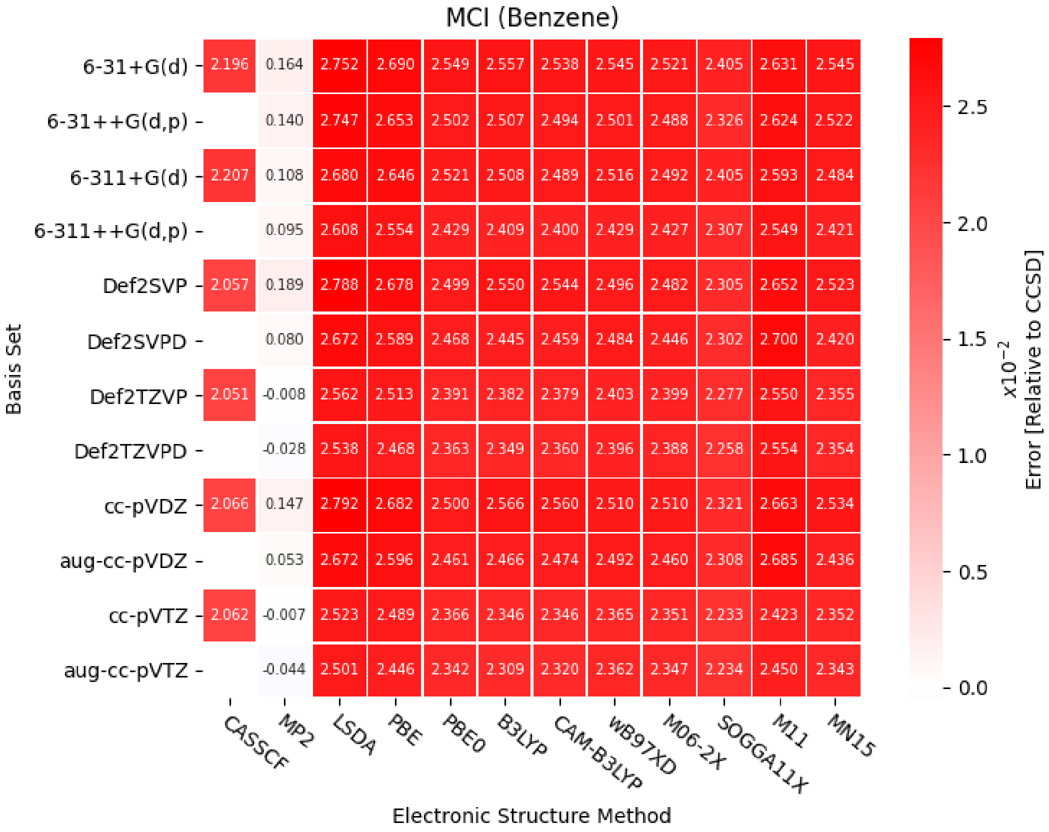
MCI (Benzene)
Basis Set
Electronic Structure Method
CASSCF MP2 LSDA PBE PBE0 B3LYP CAM-B3LYP wB97XD M06-2X SOGGA11X M11 MN15
6-31+G(d) 2.196 0.164 2.752 2.690 2.549 2.557 2.538 2.545 2.521 2.405 2.631 2.545
6-31++G(d,p) 0.140 2.747 2.653 2.502 2.507 2.494 2.501 2.488 2.326 2.624 2.522
6-311+G(d) 2.207 0.108 2.680 2.646 2.521 2.508 2.489 2.516 2.492 2.405 2.593 2.484
6-311++G(d,p) 0.095 2.608 2.554 2.429 2.409 2.400 2.429 2.427 2.307 2.549 2.421
Def2SVP 2.057 0.189 2.788 2.678 2.499 2.550 2.544 2.496 2.482 2.305 2.652 2.523
Def2SVPD 0.080 2.672 2.589 2.468 2.445 2.459 2.484 2.446 2.302 2.700 2.420
Def2TZVP 2.051 -0.008 2.562 2.513 2.391 2.382 2.379 2.403 2.399 2.277 2.550 2.355
Def2TZVPD -0.028 2.538 2.468 2.363 2.349 2.360 2.396 2.388 2.258 2.554 2.354
cc-pVDZ 2.066 0.147 2.792 2.682 2.500 2.566 2.560 2.510 2.510 2.321 2.663 2.534
aug-cc-pVDZ 0.053 2.672 2.596 2.461 2.466 2.474 2.492 2.460 2.308 2.685 2.436
cc-pVTZ 2.062 -0.007 2.523 2.489 2.366 2.346 2.346 2.365 2.351 2.233 2.423 2.352
aug-cc-pVTZ -0.044 2.501 2.446 2.342 2.309 2.320 2.362 2.347 2.234 2.450 2.343
2.5
2.0
1.5
1.0
0.5
0.0
x10^{-2}
Error [Relative to CCSD]

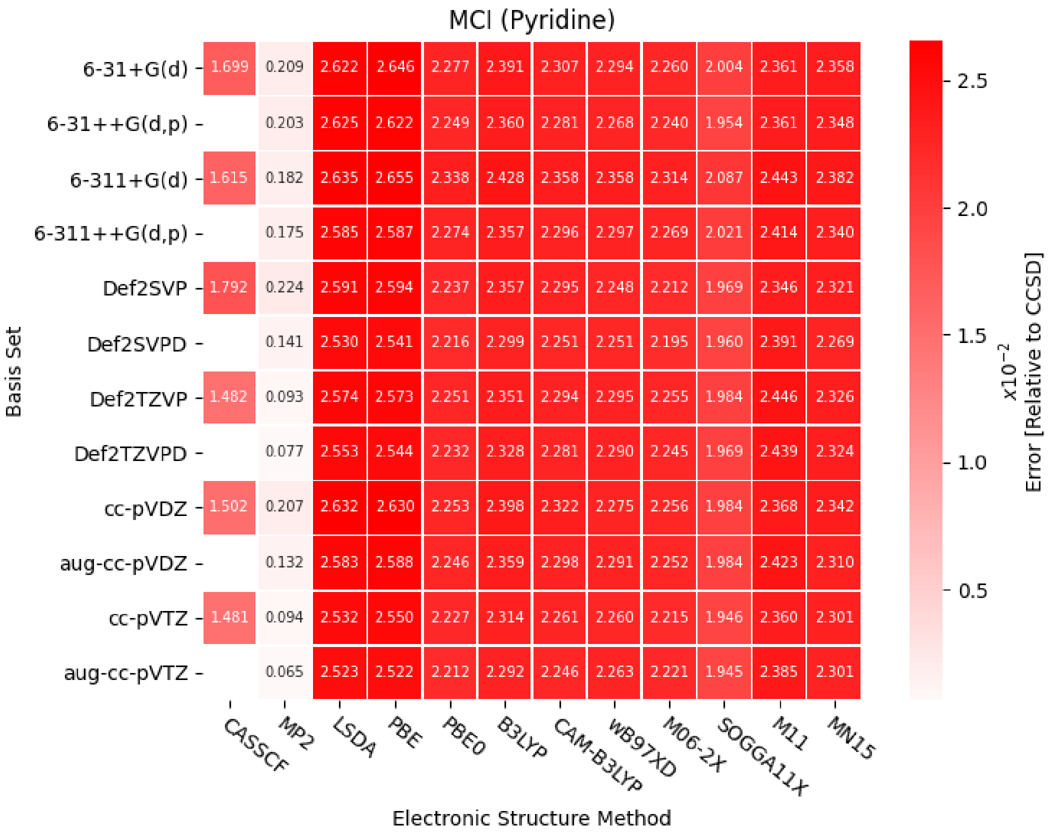
MCI (Pyridine)
6-31+G(d) 1.699 0.209 2.622 2.646 2.277 2.391 2.307 2.294 2.260 2.004 2.361 2.358
6-31++G(d,p) 0.203 2.625 2.622 2.249 2.360 2.281 2.268 2.240 1.954 2.361 2.348
6-311+G(d) 1.615 0.182 2.635 2.655 2.338 2.428 2.358 2.358 2.314 2.087 2.443 2.382
6-311++G(d,p) 0.175 2.585 2.587 2.274 2.357 2.296 2.297 2.269 2.021 2.414 2.340
Def2SVP 1.792 0.224 2.591 2.594 2.237 2.357 2.295 2.248 2.212 1.969 2.346 2.321
Def2SVPD 0.141 2.530 2.541 2.216 2.299 2.251 2.251 2.195 1.960 2.391 2.269
Def2TZVP 1.482 0.093 2.574 2.573 2.251 2.351 2.294 2.295 2.255 1.984 2.446 2.326
Def2TZVPD 0.077 2.553 2.544 2.232 2.328 2.281 2.290 2.245 1.969 2.439 2.324
cc-pVDZ 1.502 0.207 2.632 2.630 2.253 2.398 2.322 2.275 2.256 1.984 2.368 2.342
aug-cc-pVDZ 0.132 2.583 2.588 2.246 2.359 2.298 2.291 2.252 1.984 2.423 2.310
cc-pVTZ 1.481 0.094 2.532 2.550 2.227 2.314 2.261 2.260 2.215 1.946 2.360 2.301
aug-cc-pVTZ 0.065 2.523 2.522 2.212 2.292 2.246 2.263 2.221 1.945 2.385 2.301
Basis Set
CASSCF MP2 LSDA PBE PBE0 B3LYP CAM-B3LYP wB97XD M06-2X SOGGA11X M11 MN15
Electronic Structure Method
2.5 2.0 1.5 1.0 0.5
$x10^{-2}$
Error [Relative to CCSD]

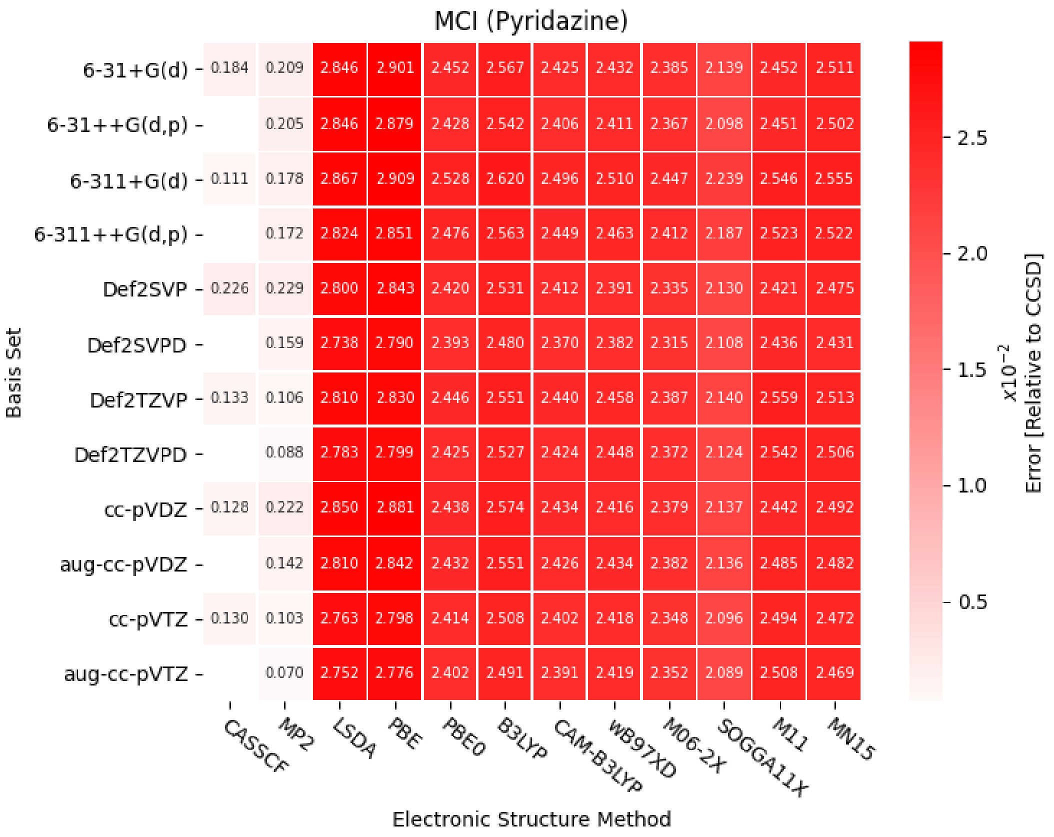
MCI (Pyridazine)
6-31+G(d) 0.184 0.209 2.846 2.901 2.452 2.567 2.425 2.432 2.385 2.139 2.452 2.511
6-31++G(d,p) 0.205 2.846 2.879 2.428 2.542 2.406 2.411 2.367 2.098 2.451 2.502
6-311+G(d) 0.111 0.178 2.867 2.909 2.528 2.620 2.496 2.510 2.447 2.239 2.546 2.555
6-311++G(d,p) 0.172 2.824 2.851 2.476 2.563 2.449 2.463 2.412 2.187 2.523 2.522
Def2SVP 0.226 0.229 2.800 2.843 2.420 2.531 2.412 2.391 2.335 2.130 2.421 2.475
Def2SVPD 0.159 2.738 2.790 2.393 2.480 2.370 2.382 2.315 2.108 2.436 2.431
Def2TZVP 0.133 0.106 2.810 2.830 2.446 2.551 2.440 2.458 2.387 2.140 2.559 2.513
Def2TZVPD 0.088 2.783 2.799 2.425 2.527 2.424 2.448 2.372 2.124 2.542 2.506
cc-pVDZ 0.128 0.222 2.850 2.881 2.438 2.574 2.434 2.416 2.379 2.137 2.442 2.492
aug-cc-pVDZ 0.142 2.810 2.842 2.432 2.551 2.426 2.434 2.382 2.136 2.485 2.482
cc-pVTZ 0.130 0.103 2.763 2.798 2.414 2.508 2.402 2.418 2.348 2.096 2.494 2.472
aug-cc-pVTZ 0.070 2.752 2.776 2.402 2.491 2.391 2.419 2.352 2.089 2.508 2.469
Basis Set
CASSCF MP2 LSDA PBE PBE0 B3LYP CAM-B3LYP wB97XD M06-2X SOGGA11X M11 MN15
Electronic Structure Method
2.5 2.0 1.5 1.0 0.5
$x10^{-2}$
Error [Relative to CCSD]

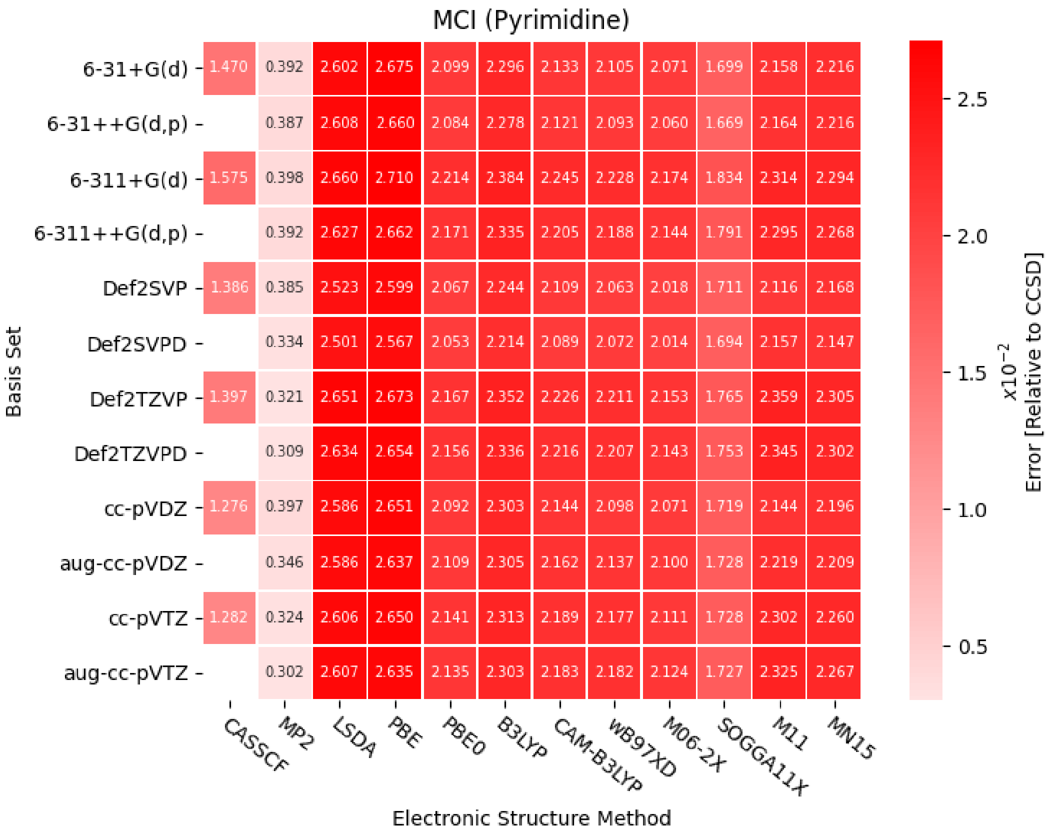
MCI (Pyrimidine)
Basis Set
Electronic Structure Method
CASSCF MP2 LSDA PBE PBE0 B3LYP CAM-B3LYP wB97XD M06-2X SOGGA11X M11 MN15
6-31+G(d) 1.470 0.392 2.602 2.675 2.099 2.296 2.133 2.105 2.071 1.699 2.158 2.216
6-31++G(d,p) 0.387 2.608 2.660 2.084 2.278 2.121 2.093 2.060 1.669 2.164 2.216
6-311+G(d) 1.575 0.398 2.660 2.710 2.214 2.384 2.245 2.228 2.174 1.834 2.314 2.294
6-311++G(d,p) 0.392 2.627 2.662 2.171 2.335 2.205 2.188 2.144 1.791 2.295 2.268
Def2SVP 1.386 0.385 2.523 2.599 2.067 2.244 2.109 2.063 2.018 1.711 2.116 2.168
Def2SVPD 0.334 2.501 2.567 2.053 2.214 2.089 2.072 2.014 1.694 2.157 2.147
Def2TZVP 1.397 0.321 2.651 2.673 2.167 2.352 2.226 2.211 2.153 1.765 2.359 2.305
Def2TZVPD 0.309 2.634 2.654 2.156 2.336 2.216 2.207 2.143 1.753 2.345 2.302
cc-pVDZ 1.276 0.397 2.586 2.651 2.092 2.303 2.144 2.098 2.071 1.719 2.144 2.196
aug-cc-pVDZ 0.346 2.586 2.637 2.109 2.305 2.162 2.137 2.100 1.728 2.219 2.209
cc-pVTZ 1.282 0.324 2.606 2.650 2.141 2.313 2.189 2.177 2.111 1.728 2.302 2.260
aug-cc-pVTZ 0.302 2.607 2.635 2.135 2.303 2.183 2.182 2.124 1.727 2.325 2.267
2.5 2.0 1.5 1.0 0.5
x10^{-2}
Error [Relative to CCSD]

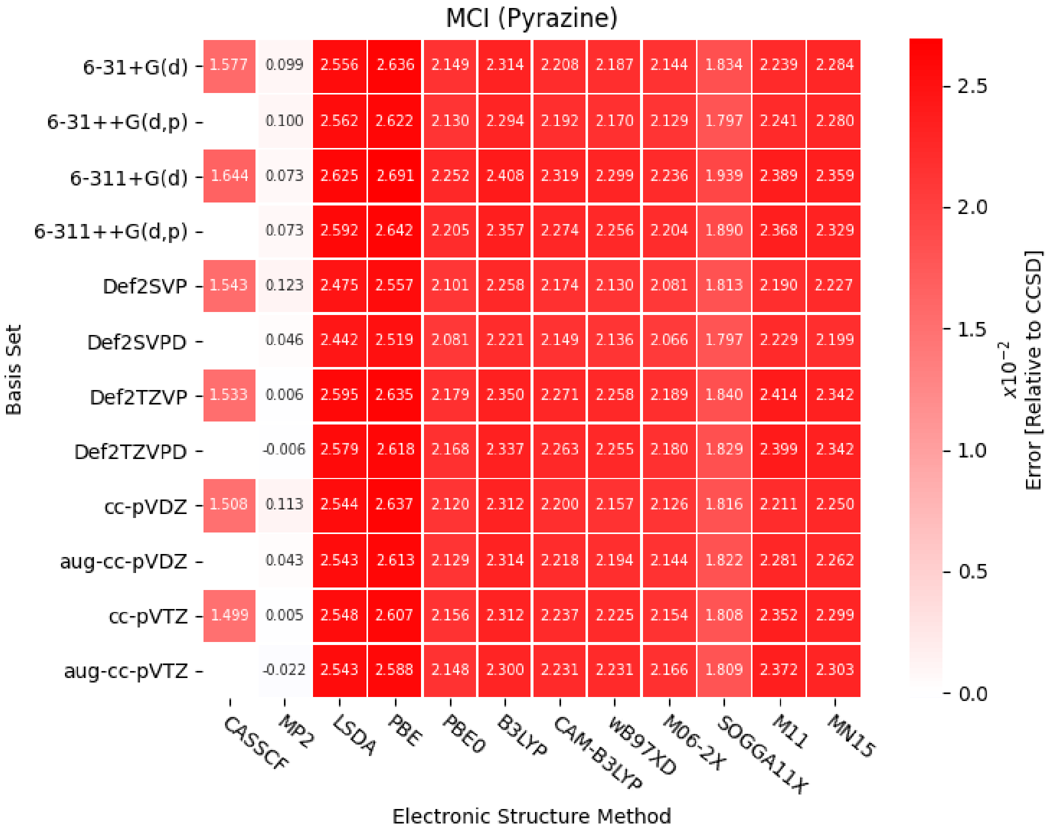
MCI (Pyrazine)
Basis Set
Electronic Structure Method
CASSCF MP2 LSDA PBE PBE0 B3LYP CAM-B3LYP wB97XD M06-2X SOGGA11X M11 MN15
6-31+G(d) 1.577 0.099 2.556 2.636 2.149 2.314 2.208 2.187 2.144 1.834 2.239 2.284
6-31++G(d,p) 0.100 2.562 2.622 2.130 2.294 2.192 2.170 2.129 1.797 2.241 2.280
6-311+G(d) 1.644 0.073 2.625 2.691 2.252 2.408 2.319 2.299 2.236 1.939 2.389 2.359
6-311++G(d,p) 0.073 2.592 2.642 2.205 2.357 2.274 2.256 2.204 1.890 2.368 2.329
Def2SVP 1.543 0.123 2.475 2.557 2.101 2.258 2.174 2.130 2.081 1.813 2.190 2.227
Def2SVPD 0.046 2.442 2.519 2.081 2.221 2.149 2.136 2.066 1.797 2.229 2.199
Def2TZVP 1.533 0.006 2.595 2.635 2.179 2.350 2.271 2.258 2.189 1.840 2.414 2.342
Def2TZVPD -0.006 2.579 2.618 2.168 2.337 2.263 2.255 2.180 1.829 2.399 2.342
cc-pVDZ 1.508 0.113 2.544 2.637 2.120 2.312 2.200 2.157 2.126 1.816 2.211 2.250
aug-cc-pVDZ 0.043 2.543 2.613 2.129 2.314 2.218 2.194 2.144 1.822 2.281 2.262
cc-pVTZ 1.499 0.005 2.548 2.607 2.156 2.312 2.237 2.225 2.154 1.808 2.352 2.299
aug-cc-pVTZ -0.022 2.543 2.588 2.148 2.300 2.231 2.231 2.166 1.809 2.372 2.303
2.5 2.0 1.5 1.0 0.5 0.0
x10^{-2}
Error [Relative to CCSD]

D.4 AV1245 Index

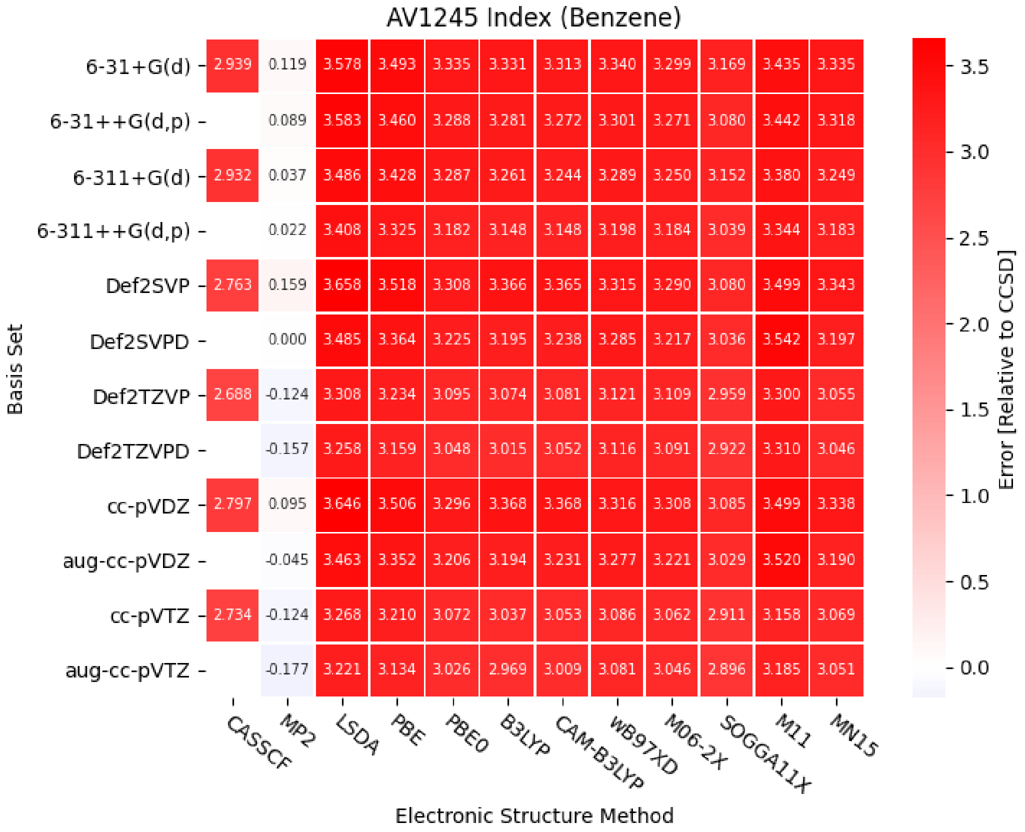

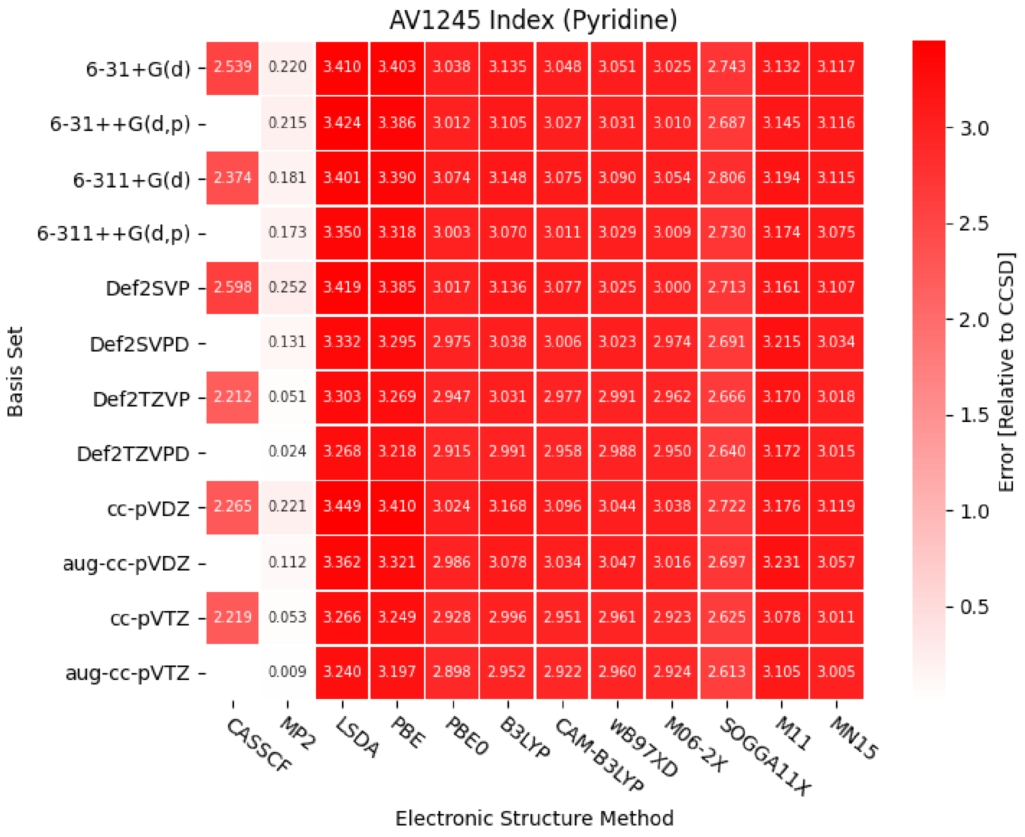
AV1245 Index (Pyridine)
Basis Set
6-31+G(d) 2.539 0.220 3.410 3.403 3.038 3.135 3.048 3.051 3.025 2.743 3.132 3.117
6-31++G(d,p) 0.215 3.424 3.386 3.012 3.105 3.027 3.031 3.010 2.687 3.145 3.116
6-311+G(d) 2.374 0.181 3.401 3.390 3.074 3.148 3.075 3.090 3.054 2.806 3.194 3.115
6-311++G(d,p) 0.173 3.350 3.318 3.003 3.070 3.011 3.029 3.009 2.730 3.174 3.075
Def2SVP 2.598 0.252 3.419 3.385 3.017 3.136 3.077 3.025 3.000 2.713 3.161 3.107
Def2SVPD 0.131 3.332 3.295 2.975 3.038 3.006 3.023 2.974 2.691 3.215 3.034
Def2TZVP 2.212 0.051 3.303 3.269 2.947 3.031 2.977 2.991 2.962 2.666 3.170 3.018
Def2TZVPD 0.024 3.268 3.218 2.915 2.991 2.958 2.988 2.950 2.640 3.172 3.015
cc-pVDZ 2.265 0.221 3.449 3.410 3.024 3.168 3.096 3.044 3.038 2.722 3.176 3.119
aug-cc-pVDZ 0.112 3.362 3.321 2.986 3.078 3.034 3.047 3.016 2.697 3.231 3.057
cc-pVTZ 2.219 0.053 3.266 3.249 2.928 2.996 2.951 2.961 2.923 2.625 3.078 3.011
aug-cc-pVTZ 0.009 3.240 3.197 2.898 2.952 2.922 2.960 2.924 2.613 3.105 3.005
CASSCF MP2 LSDA PBE PBE0 B3LYP CAM-B3LYP wB97XD M06-2X SOGGA11X M11 MN15
Electronic Structure Method
Error [Relative to CCSD]
3.0 2.5 2.0 1.5 1.0 0.5

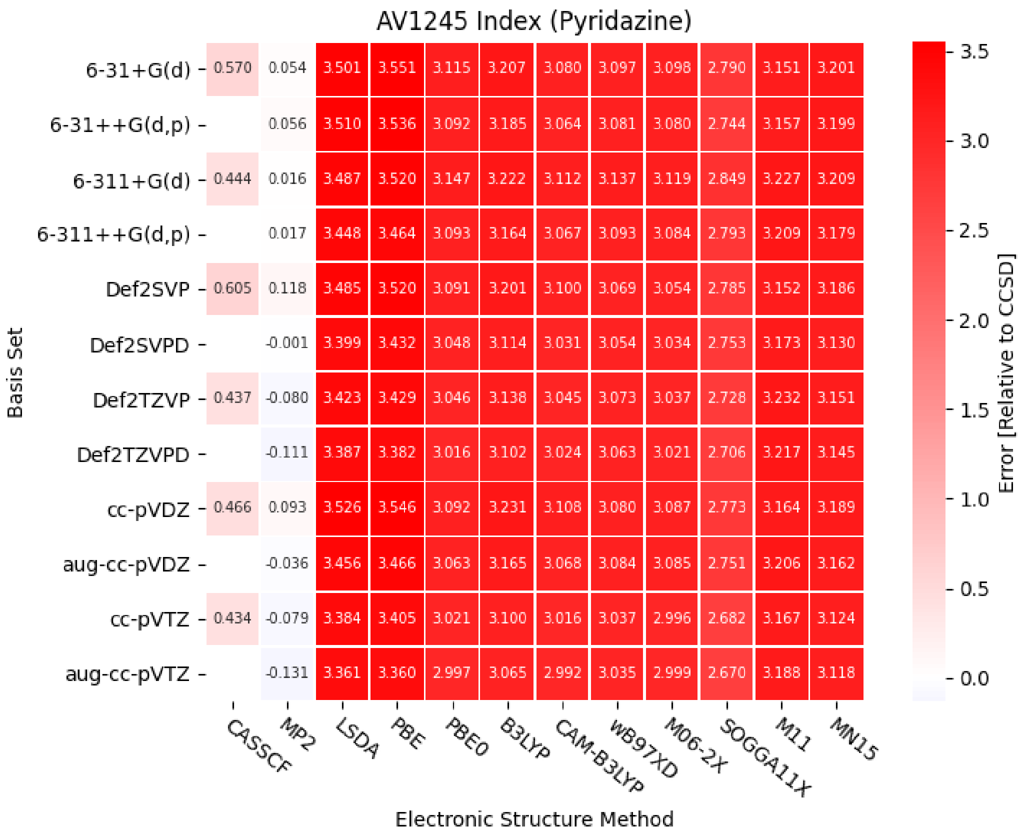
AV1245 Index (Pyridazine)
Basis Set
6-31+G(d) 0.570 0.054 3.501 3.551 3.115 3.207 3.080 3.097 3.098 2.790 3.151 3.201
6-31++G(d,p) 0.056 3.510 3.536 3.092 3.185 3.064 3.081 3.080 2.744 3.157 3.199
6-311+G(d) 0.444 0.016 3.487 3.520 3.147 3.222 3.112 3.137 3.119 2.849 3.227 3.209
6-311++G(d,p) 0.017 3.448 3.464 3.093 3.164 3.067 3.093 3.084 2.793 3.209 3.179
Def2SVP 0.605 0.118 3.485 3.520 3.091 3.201 3.100 3.069 3.054 2.785 3.152 3.186
Def2SVPD -0.001 3.399 3.432 3.048 3.114 3.031 3.054 3.034 2.753 3.173 3.130
Def2TZVP 0.437 -0.080 3.423 3.429 3.046 3.138 3.045 3.073 3.037 2.728 3.232 3.151
Def2TZVPD -0.111 3.387 3.382 3.016 3.102 3.024 3.063 3.021 2.706 3.217 3.145
cc-pVDZ 0.466 0.093 3.526 3.546 3.092 3.231 3.108 3.080 3.087 2.773 3.164 3.189
aug-cc-pVDZ -0.036 3.456 3.466 3.063 3.165 3.068 3.084 3.085 2.751 3.206 3.162
cc-pVTZ 0.434 -0.079 3.384 3.405 3.021 3.100 3.016 3.037 2.996 2.682 3.167 3.124
aug-cc-pVTZ -0.131 3.361 3.360 2.997 3.065 2.992 3.035 2.999 2.670 3.188 3.118
CASSCF MP2 LSDA PBE PBE0 B3LYP CAM-B3LYP wB97XD M06-2X SOGGA11X M11 MN15
Electronic Structure Method
Error [Relative to CCSD]
3.5 3.0 2.5 2.0 1.5 1.0 0.5 0.0

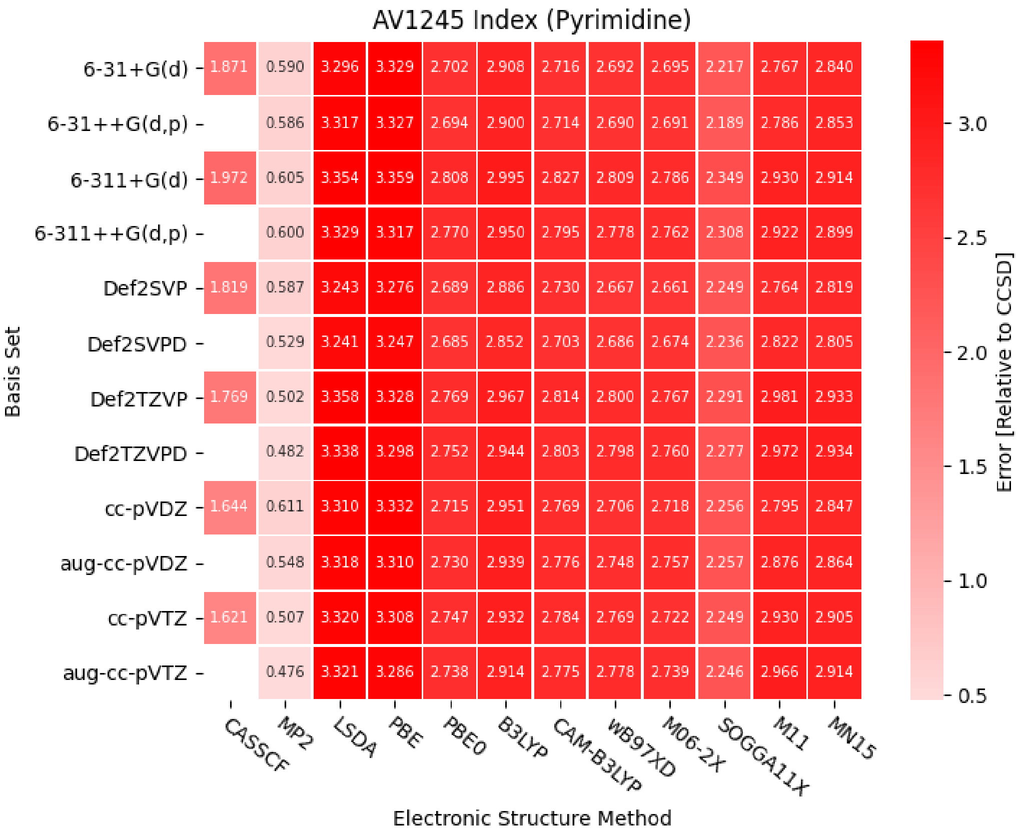
AV1245 Index (Pyrimidine)
Basis Set
Electronic Structure Method
Error [Relative to CCSD]
CASSCF MP2 LSDA PBE PBE0 B3LYP CAM-B3LYP wB97XD M06-2X SOGGA11X M11 MN15
6-31+G(d) 1.871 0.590 3.296 3.329 2.702 2.908 2.716 2.692 2.695 2.217 2.767 2.840
6-31++G(d,p) 0.586 3.317 3.327 2.694 2.900 2.714 2.690 2.691 2.189 2.786 2.853
6-311+G(d) 1.972 0.605 3.354 3.359 2.808 2.995 2.827 2.809 2.786 2.349 2.930 2.914
6-311++G(d,p) 0.600 3.329 3.317 2.770 2.950 2.795 2.778 2.762 2.308 2.922 2.899
Def2SVP 1.819 0.587 3.243 3.276 2.689 2.886 2.730 2.667 2.661 2.249 2.764 2.819
Def2SVPD 0.529 3.241 3.247 2.685 2.852 2.703 2.686 2.674 2.236 2.822 2.805
Def2TZVP 1.769 0.502 3.358 3.328 2.769 2.967 2.814 2.800 2.767 2.291 2.981 2.933
Def2TZVPD 0.482 3.338 3.298 2.752 2.944 2.803 2.798 2.760 2.277 2.972 2.934
cc-pVDZ 1.644 0.611 3.310 3.332 2.715 2.951 2.769 2.706 2.718 2.256 2.795 2.847
aug-cc-pVDZ 0.548 3.318 3.310 2.730 2.939 2.776 2.748 2.757 2.257 2.876 2.864
cc-pVTZ 1.621 0.507 3.320 3.308 2.747 2.932 2.784 2.769 2.722 2.249 2.930 2.905
aug-cc-pVTZ 0.476 3.321 3.286 2.738 2.914 2.775 2.778 2.739 2.246 2.966 2.914
0.5 1.0 1.5 2.0 2.5 3.0

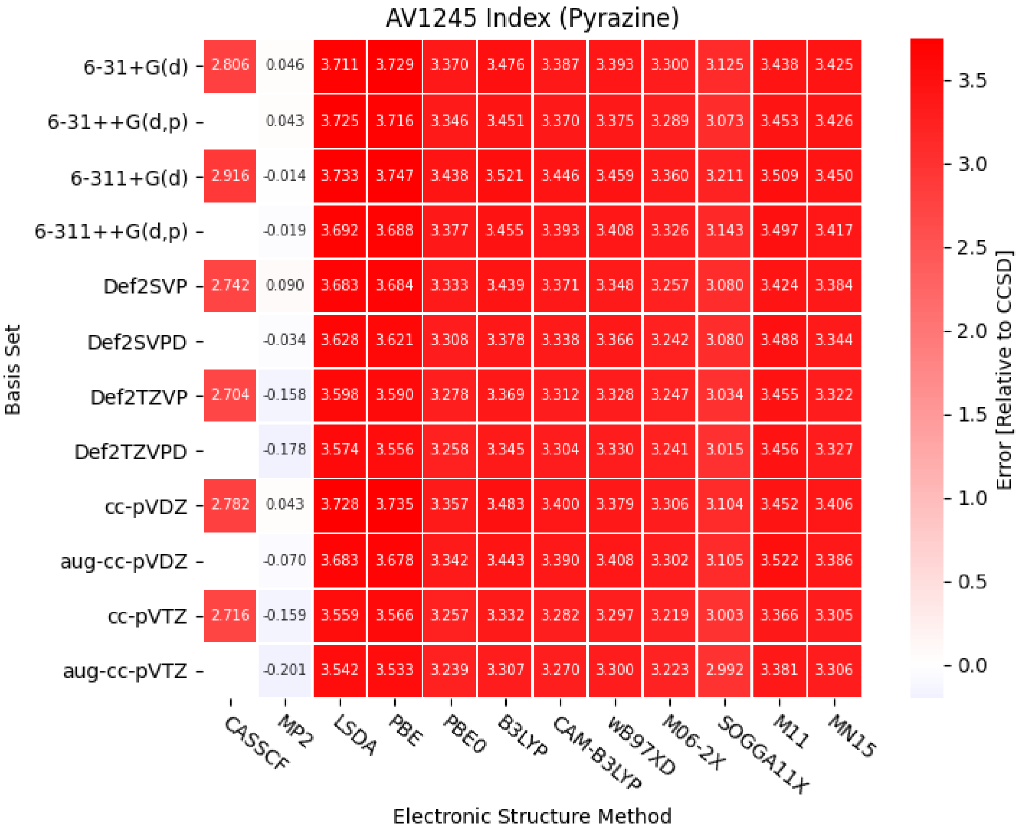
AV1245 Index (Pyrazine)
Basis Set
Electronic Structure Method
Error [Relative to CCSD]
CASSCF MP2 LSDA PBE PBE0 B3LYP CAM-B3LYP wB97XD M06-2X SOGGA11X M11 MN15
6-31+G(d) 2.806 0.046 3.711 3.729 3.370 3.476 3.387 3.393 3.300 3.125 3.438 3.425
6-31++G(d,p) 0.043 3.725 3.716 3.346 3.451 3.370 3.375 3.289 3.073 3.453 3.426
6-311+G(d) 2.916 -0.014 3.733 3.747 3.438 3.521 3.446 3.459 3.360 3.211 3.509 3.450
6-311++G(d,p) -0.019 3.692 3.688 3.377 3.455 3.393 3.408 3.326 3.143 3.497 3.417
Def2SVP 2.742 0.090 3.683 3.684 3.333 3.439 3.371 3.348 3.257 3.080 3.424 3.384
Def2SVPD -0.034 3.628 3.621 3.308 3.378 3.338 3.366 3.242 3.080 3.488 3.344
Def2TZVP 2.704 -0.158 3.598 3.590 3.278 3.369 3.312 3.328 3.247 3.034 3.455 3.322
Def2TZVPD -0.178 3.574 3.556 3.258 3.345 3.304 3.330 3.241 3.015 3.456 3.327
cc-pVDZ 2.782 0.043 3.728 3.735 3.357 3.483 3.400 3.379 3.306 3.104 3.452 3.406
aug-cc-pVDZ -0.070 3.683 3.678 3.342 3.443 3.390 3.408 3.302 3.105 3.522 3.386
cc-pVTZ 2.716 -0.159 3.559 3.566 3.257 3.332 3.282 3.297 3.219 3.003 3.366 3.305
aug-cc-pVTZ -0.201 3.542 3.533 3.239 3.307 3.270 3.300 3.223 2.992 3.381 3.306
0.0 0.5 1.0 1.5 2.0 2.5 3.0 3.5

D.5 FLU Index

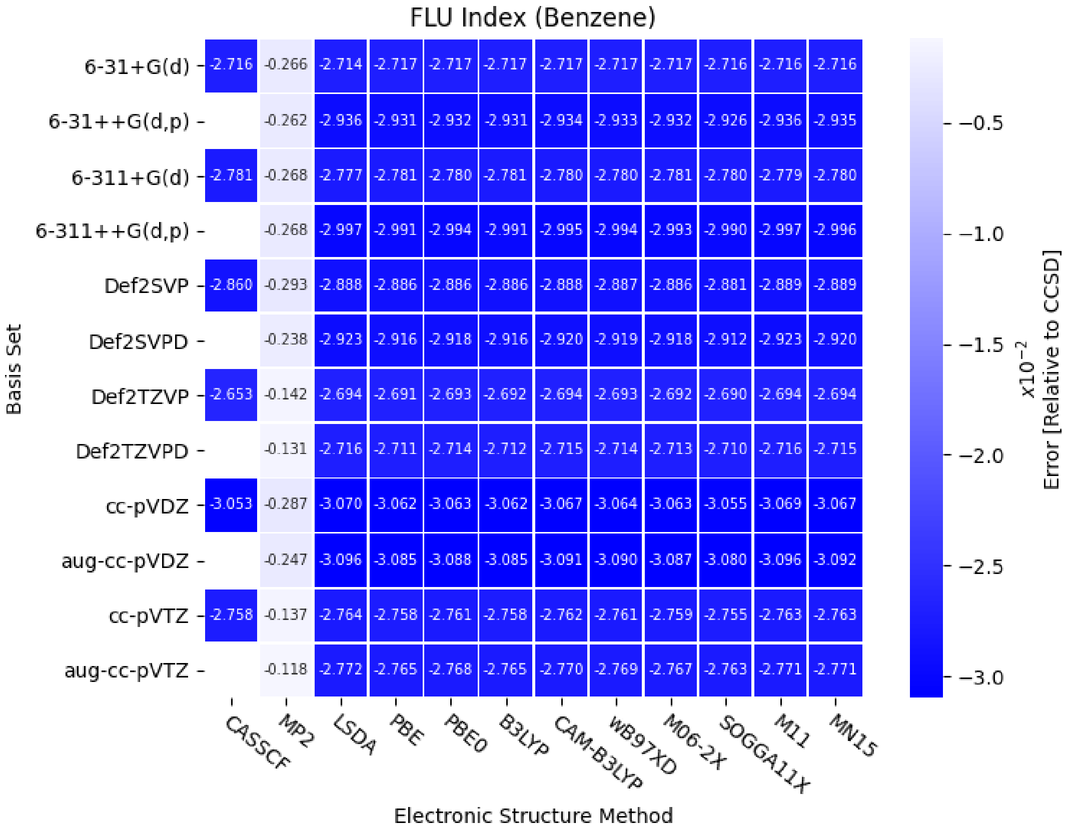
FLU Index (Benzene)
Basis Set
6-31+G(d)
6-31++G(d,p)
6-311+G(d)
6-311++G(d,p)
Def2SVP
Def2SVPD
Def2TZVP
Def2TZVPD
cc-pVDZ
aug-cc-pVDZ
cc-pVTZ
aug-cc-pVTZ
-2.716 -0.266 -2.714 -2.717 -2.717 -2.717 -2.717 -2.717 -2.717 -2.716 -2.716 -2.716
-0.262 -2.936 -2.931 -2.932 -2.931 -2.934 -2.933 -2.932 -2.926 -2.936 -2.935
-2.781 -0.268 -2.777 -2.781 -2.780 -2.781 -2.780 -2.780 -2.781 -2.780 -2.779 -2.780
-0.268 -2.997 -2.991 -2.994 -2.991 -2.995 -2.994 -2.993 -2.990 -2.997 -2.996
-2.860 -0.293 -2.888 -2.886 -2.886 -2.886 -2.888 -2.887 -2.886 -2.881 -2.889 -2.889
-0.238 -2.923 -2.916 -2.918 -2.916 -2.920 -2.919 -2.918 -2.912 -2.923 -2.920
-2.653 -0.142 -2.694 -2.691 -2.693 -2.692 -2.694 -2.693 -2.692 -2.690 -2.694 -2.694
-0.131 -2.716 -2.711 -2.714 -2.712 -2.715 -2.714 -2.713 -2.710 -2.716 -2.715
-3.053 -0.287 -3.070 -3.062 -3.063 -3.062 -3.067 -3.064 -3.063 -3.055 -3.069 -3.067
-0.247 -3.096 -3.085 -3.088 -3.085 -3.091 -3.090 -3.087 -3.080 -3.096 -3.092
-2.758 -0.137 -2.764 -2.758 -2.761 -2.758 -2.762 -2.761 -2.759 -2.755 -2.763 -2.763
-0.118 -2.772 -2.765 -2.768 -2.765 -2.770 -2.769 -2.767 -2.763 -2.771 -2.771
CASSCF
MP2
LSDA
PBE
PBE0
B3LYP
CAM-B3LYP
wB97XD
M06-2X
SOGGA11X
M11
MN15
Electronic Structure Method
−0.5
−1.0
−1.5
−2.0
−2.5
−3.0
x10^{-2}
Error [Relative to CCSD]

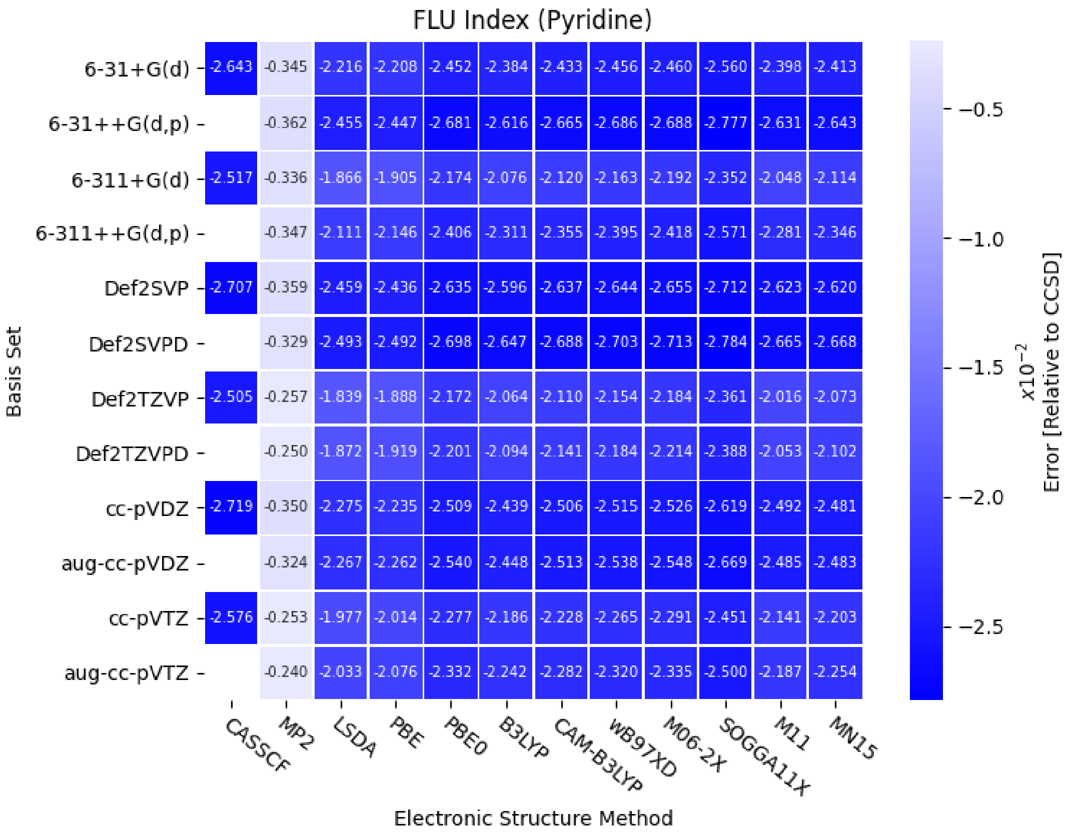
FLU Index (Pyridine)
Basis Set
6-31+G(d) -2.643 -0.345 -2.216 -2.208 -2.452 -2.384 -2.433 -2.456 -2.460 -2.560 -2.398 -2.413
6-31++G(d,p) -0.362 -2.455 -2.447 -2.681 -2.616 -2.665 -2.686 -2.688 -2.777 -2.631 -2.643
6-311+G(d) -2.517 -0.336 -1.866 -1.905 -2.174 -2.076 -2.120 -2.163 -2.192 -2.352 -2.048 -2.114
6-311++G(d,p) -0.347 -2.111 -2.146 -2.406 -2.311 -2.355 -2.395 -2.418 -2.571 -2.281 -2.346
Def2SVP -2.707 -0.359 -2.459 -2.436 -2.635 -2.596 -2.637 -2.644 -2.655 -2.712 -2.623 -2.620
Def2SVPD -0.329 -2.493 -2.492 -2.698 -2.647 -2.688 -2.703 -2.713 -2.784 -2.665 -2.668
Def2TZVP -2.505 -0.257 -1.839 -1.888 -2.172 -2.064 -2.110 -2.154 -2.184 -2.361 -2.016 -2.073
Def2TZVPD -0.250 -1.872 -1.919 -2.201 -2.094 -2.141 -2.184 -2.214 -2.388 -2.053 -2.102
cc-pVDZ -2.719 -0.350 -2.275 -2.235 -2.509 -2.439 -2.506 -2.515 -2.526 -2.619 -2.492 -2.481
aug-cc-pVDZ -0.324 -2.267 -2.262 -2.540 -2.448 -2.513 -2.538 -2.548 -2.669 -2.485 -2.483
cc-pVTZ -2.576 -0.253 -1.977 -2.014 -2.277 -2.186 -2.228 -2.265 -2.291 -2.451 -2.141 -2.203
aug-cc-pVTZ -0.240 -2.033 -2.076 -2.332 -2.242 -2.282 -2.320 -2.335 -2.500 -2.187 -2.254
CASSCF MP2 LSDA PBE PBE0 B3LYP CAM-B3LYP wB97XD M06-2X SOGGA11X M11 MN15
Electronic Structure Method
−0.5 −1.0 −1.5 −2.0 −2.5
x10^{-2}
Error [Relative to CCSD]

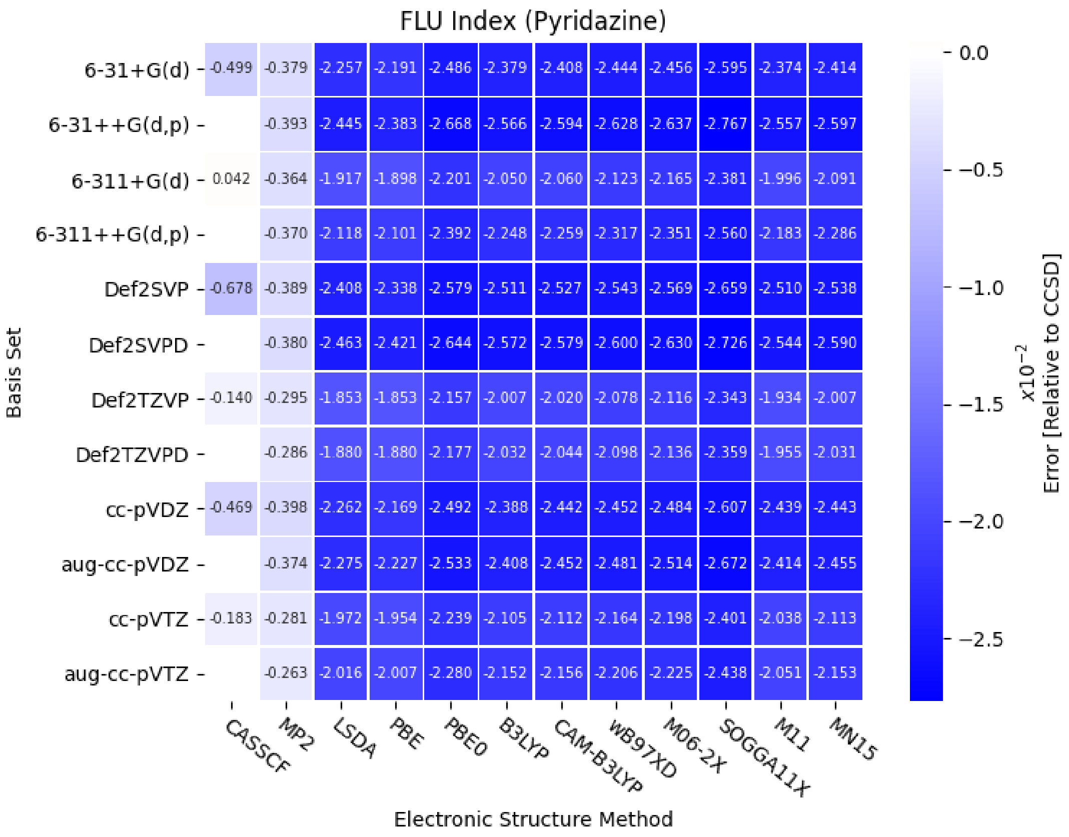
FLU Index (Pyridazine)
Basis Set
6-31+G(d) -0.499 -0.379 -2.257 -2.191 -2.486 -2.379 -2.408 -2.444 -2.456 -2.595 -2.374 -2.414
6-31++G(d,p) -0.393 -2.445 -2.383 -2.668 -2.566 -2.594 -2.628 -2.637 -2.767 -2.557 -2.597
6-311+G(d) 0.042 -0.364 -1.917 -1.898 -2.201 -2.050 -2.060 -2.123 -2.165 -2.381 -1.996 -2.091
6-311++G(d,p) -0.370 -2.118 -2.101 -2.392 -2.248 -2.259 -2.317 -2.351 -2.560 -2.183 -2.286
Def2SVP -0.678 -0.389 -2.408 -2.338 -2.579 -2.511 -2.527 -2.543 -2.569 -2.659 -2.510 -2.538
Def2SVPD -0.380 -2.463 -2.421 -2.644 -2.572 -2.579 -2.600 -2.630 -2.726 -2.544 -2.590
Def2TZVP -0.140 -0.295 -1.853 -1.853 -2.157 -2.007 -2.020 -2.078 -2.116 -2.343 -1.934 -2.007
Def2TZVPD -0.286 -1.880 -1.880 -2.177 -2.032 -2.044 -2.098 -2.136 -2.359 -1.955 -2.031
cc-pVDZ -0.469 -0.398 -2.262 -2.169 -2.492 -2.388 -2.442 -2.452 -2.484 -2.607 -2.439 -2.443
aug-cc-pVDZ -0.374 -2.275 -2.227 -2.533 -2.408 -2.452 -2.481 -2.514 -2.672 -2.414 -2.455
cc-pVTZ -0.183 -0.281 -1.972 -1.954 -2.239 -2.105 -2.112 -2.164 -2.198 -2.401 -2.038 -2.113
aug-cc-pVTZ -0.263 -2.016 -2.007 -2.280 -2.152 -2.156 -2.206 -2.225 -2.438 -2.051 -2.153
CASSCF MP2 LSDA PBE PBE0 B3LYP CAM-B3LYP wB97XD M06-2X SOGGA11X M11 MN15
Electronic Structure Method
0.0 −0.5 −1.0 −1.5 −2.0 −2.5
x10^{-2}
Error [Relative to CCSD]

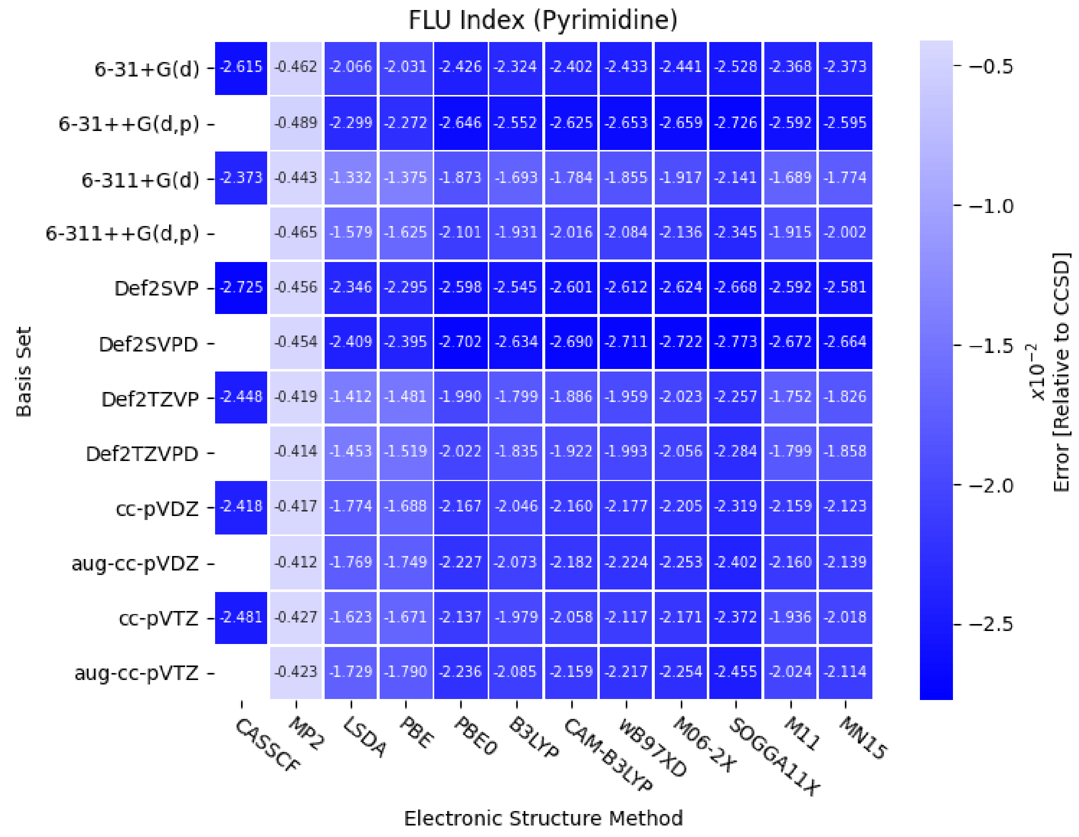
FLU Index (Pyrimidine)
Basis Set
6-31+G(d) -2.615 -0.462 -2.066 -2.031 -2.426 -2.324 -2.402 -2.433 -2.441 -2.528 -2.368 -2.373
6-31++G(d,p) -0.489 -2.299 -2.272 -2.646 -2.552 -2.625 -2.653 -2.659 -2.726 -2.592 -2.595
6-311+G(d) -2.373 -0.443 -1.332 -1.375 -1.873 -1.693 -1.784 -1.855 -1.917 -2.141 -1.689 -1.774
6-311++G(d,p) -0.465 -1.579 -1.625 -2.101 -1.931 -2.016 -2.084 -2.136 -2.345 -1.915 -2.002
Def2SVP -2.725 -0.456 -2.346 -2.295 -2.598 -2.545 -2.601 -2.612 -2.624 -2.668 -2.592 -2.581
Def2SVPD -0.454 -2.409 -2.395 -2.702 -2.634 -2.690 -2.711 -2.722 -2.773 -2.672 -2.664
Def2TZVP -2.448 -0.419 -1.412 -1.481 -1.990 -1.799 -1.886 -1.959 -2.023 -2.257 -1.752 -1.826
Def2TZVPD -0.414 -1.453 -1.519 -2.022 -1.835 -1.922 -1.993 -2.056 -2.284 -1.799 -1.858
cc-pVDZ -2.418 -0.417 -1.774 -1.688 -2.167 -2.046 -2.160 -2.177 -2.205 -2.319 -2.159 -2.123
aug-cc-pVDZ -0.412 -1.769 -1.749 -2.227 -2.073 -2.182 -2.224 -2.253 -2.402 -2.160 -2.139
cc-pVTZ -2.481 -0.427 -1.623 -1.671 -2.137 -1.979 -2.058 -2.117 -2.171 -2.372 -1.936 -2.018
aug-cc-pVTZ -0.423 -1.729 -1.790 -2.236 -2.085 -2.159 -2.217 -2.254 -2.455 -2.024 -2.114
CASSCF MP2 LSDA PBE PBE0 B3LYP CAM-B3LYP wB97XD M06-2X SOGGA11X M11 MN15
Electronic Structure Method
−0.5
−1.0
−1.5
−2.0
−2.5
x10^−2
Error [Relative to CCSD]

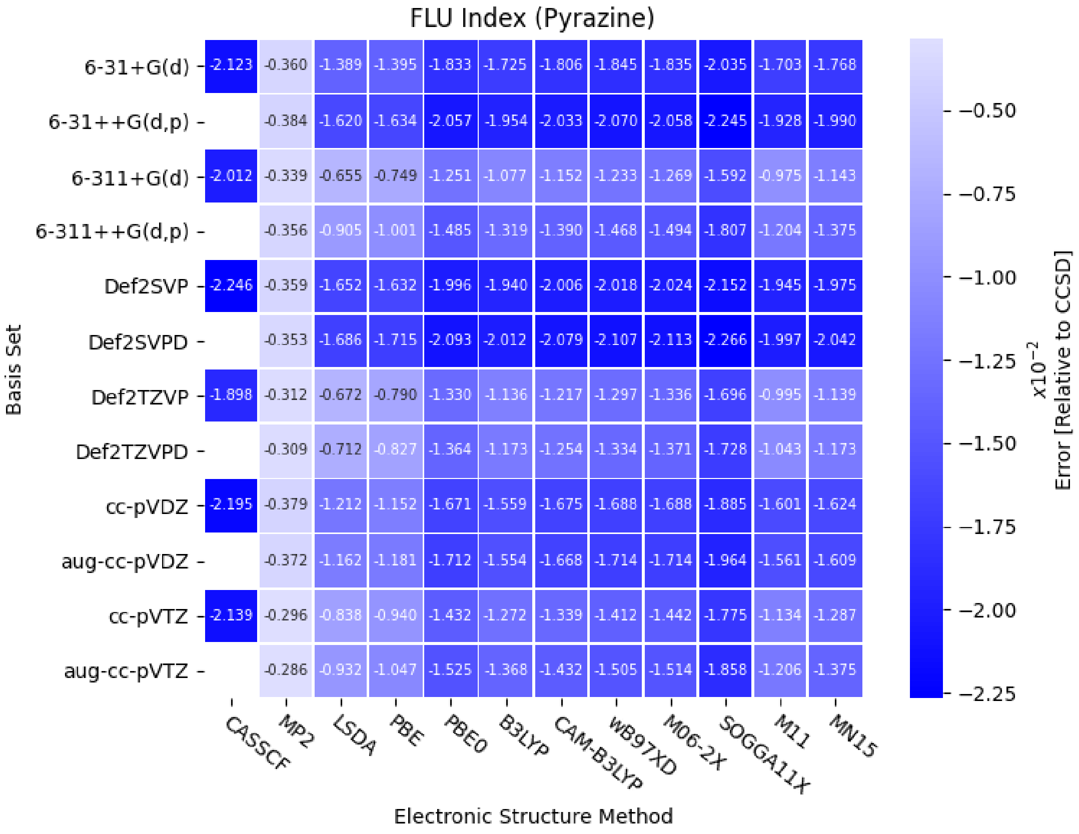
FLU Index (Pyrazine)
Basis Set
6-31+G(d) -2.123 -0.360 -1.389 -1.395 -1.833 -1.725 -1.806 -1.845 -1.835 -2.035 -1.703 -1.768
6-31++G(d,p) -0.384 -1.620 -1.634 -2.057 -1.954 -2.033 -2.070 -2.058 -2.245 -1.928 -1.990
6-311+G(d) -2.012 -0.339 -0.655 -0.749 -1.251 -1.077 -1.152 -1.233 -1.269 -1.592 -0.975 -1.143
6-311++G(d,p) -0.356 -0.905 -1.001 -1.485 -1.319 -1.390 -1.468 -1.494 -1.807 -1.204 -1.375
Def2SVP -2.246 -0.359 -1.652 -1.632 -1.996 -1.940 -2.006 -2.018 -2.024 -2.152 -1.945 -1.975
Def2SVPD -0.353 -1.686 -1.715 -2.093 -2.012 -2.079 -2.107 -2.113 -2.266 -1.997 -2.042
Def2TZVP -1.898 -0.312 -0.672 -0.790 -1.330 -1.136 -1.217 -1.297 -1.336 -1.696 -0.995 -1.139
Def2TZVPD -0.309 -0.712 -0.827 -1.364 -1.173 -1.254 -1.334 -1.371 -1.728 -1.043 -1.173
cc-pVDZ -2.195 -0.379 -1.212 -1.152 -1.671 -1.559 -1.675 -1.688 -1.688 -1.885 -1.601 -1.624
aug-cc-pVDZ -0.372 -1.162 -1.181 -1.712 -1.554 -1.668 -1.714 -1.714 -1.964 -1.561 -1.609
cc-pVTZ -2.139 -0.296 -0.838 -0.940 -1.432 -1.272 -1.339 -1.412 -1.442 -1.775 -1.134 -1.287
aug-cc-pVTZ -0.286 -0.932 -1.047 -1.525 -1.368 -1.432 -1.505 -1.514 -1.858 -1.206 -1.375
CASSCF MP2 LSDA PBE PBE0 B3LYP CAM-B3LYP wB97XD M06-2X SOGGA11X M11 MN15
Electronic Structure Method
−0.50
−0.75
−1.00
−1.25
−1.50
−1.75
−2.00
−2.25
x10^−2
Error [Relative to CCSD]

D.6 Electronic Energy

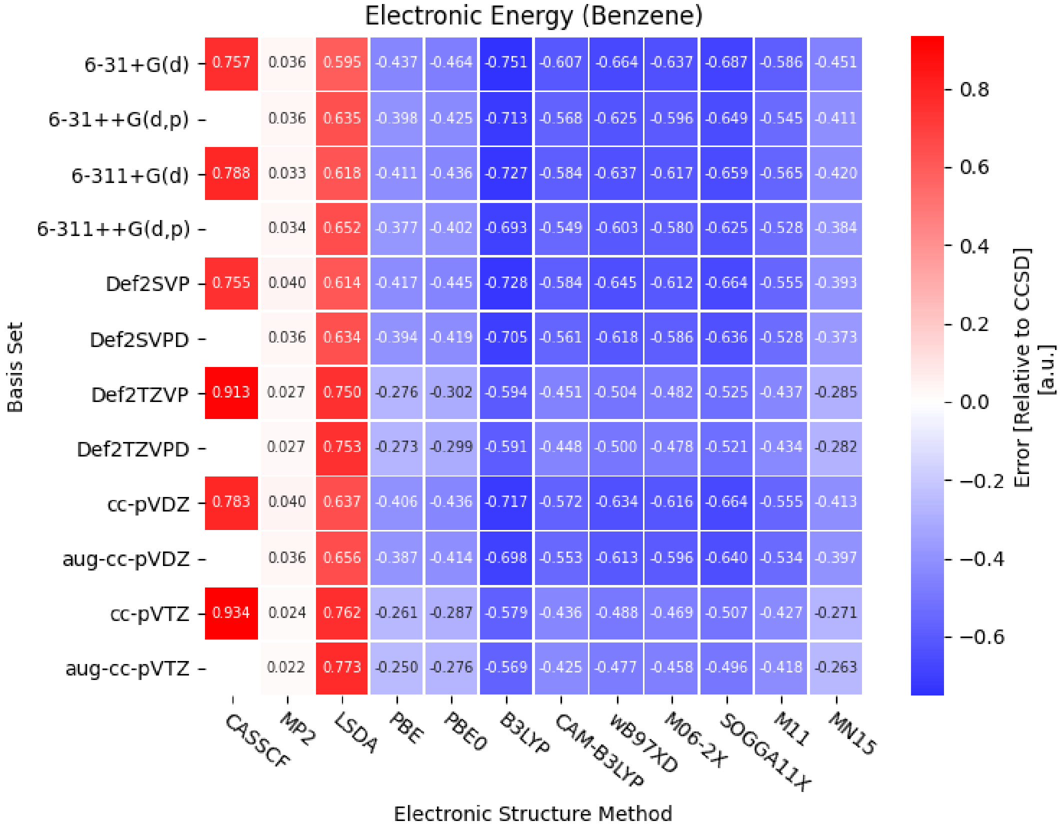

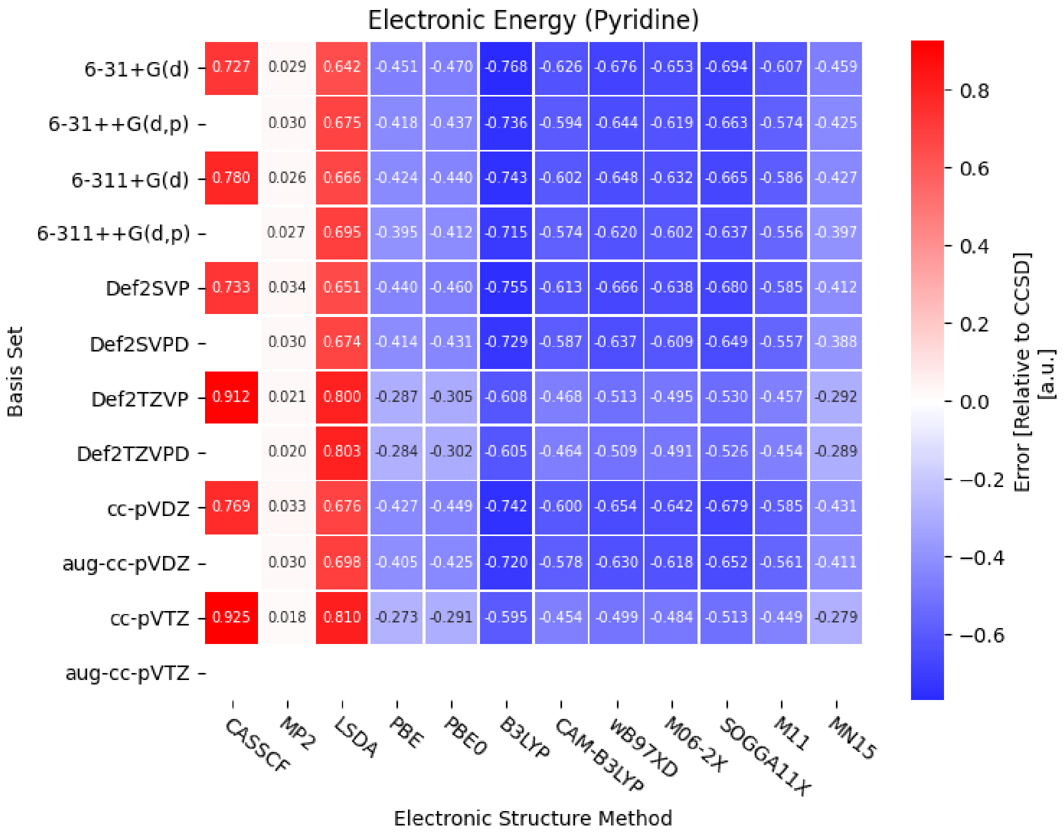
Electronic Energy (Pyridine)
Basis Set
6-31+G(d) 0.727 0.029 0.642 -0.451 -0.470 -0.768 -0.626 -0.676 -0.653 -0.694 -0.607 -0.459
6-31++G(d,p) 0.030 0.675 -0.418 -0.437 -0.736 -0.594 -0.644 -0.619 -0.663 -0.574 -0.425
6-311+G(d) 0.780 0.026 0.666 -0.424 -0.440 -0.743 -0.602 -0.648 -0.632 -0.665 -0.586 -0.427
6-311++G(d,p) 0.027 0.695 -0.395 -0.412 -0.715 -0.574 -0.620 -0.602 -0.637 -0.556 -0.397
Def2SVP 0.733 0.034 0.651 -0.440 -0.460 -0.755 -0.613 -0.666 -0.638 -0.680 -0.585 -0.412
Def2SVPD 0.030 0.674 -0.414 -0.431 -0.729 -0.587 -0.637 -0.609 -0.649 -0.557 -0.388
Def2TZVP 0.912 0.021 0.800 -0.287 -0.305 -0.608 -0.468 -0.513 -0.495 -0.530 -0.457 -0.292
Def2TZVPD 0.020 0.803 -0.284 -0.302 -0.605 -0.464 -0.509 -0.491 -0.526 -0.454 -0.289
cc-pVDZ 0.769 0.033 0.676 -0.427 -0.449 -0.742 -0.600 -0.654 -0.642 -0.679 -0.585 -0.431
aug-cc-pVDZ 0.030 0.698 -0.405 -0.425 -0.720 -0.578 -0.630 -0.618 -0.652 -0.561 -0.411
cc-pVTZ 0.925 0.018 0.810 -0.273 -0.291 -0.595 -0.454 -0.499 -0.484 -0.513 -0.449 -0.279
aug-cc-pVTZ
CASSCF MP2 LSDA PBE PBE0 B3LYP CAM-B3LYP wB97XD M06-2X SOGGA11X M11 MN15
Electronic Structure Method
0.8 0.6 0.4 0.2 0.0 −0.2 −0.4 −0.6
Error [Relative to CCSD] [a.u.]

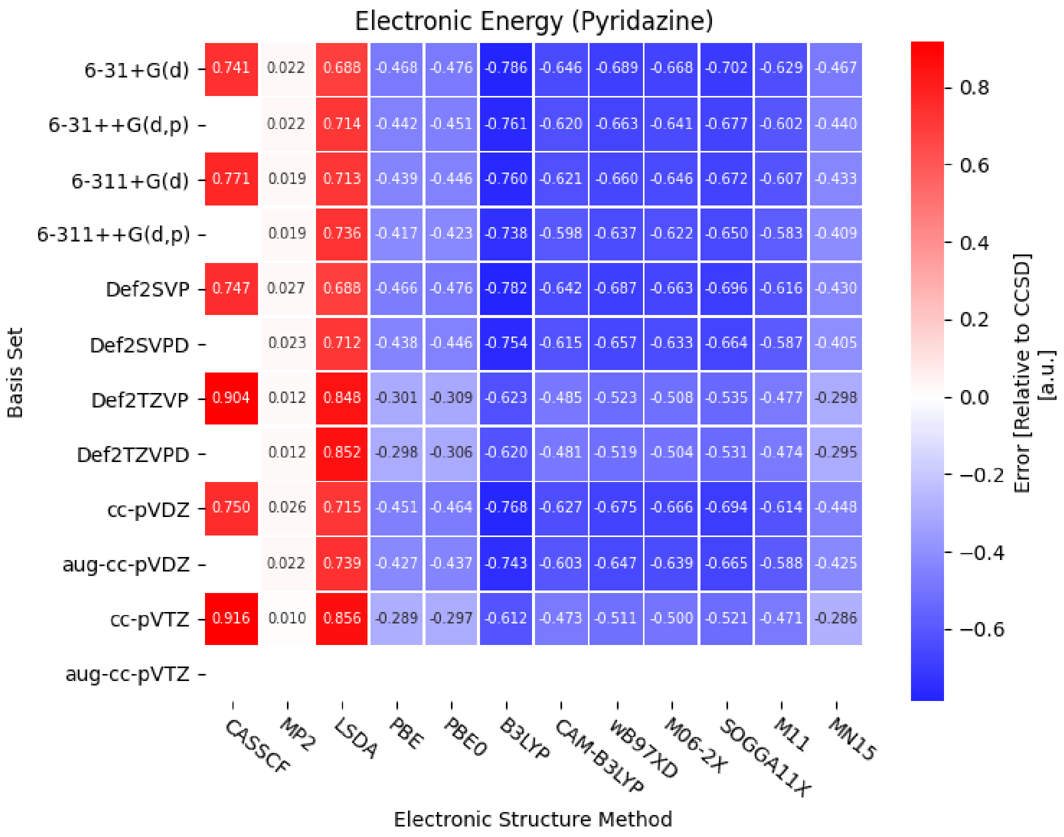
Electronic Energy (Pyridazine)
Basis Set
6-31+G(d) 0.741 0.022 0.688 -0.468 -0.476 -0.786 -0.646 -0.689 -0.668 -0.702 -0.629 -0.467
6-31++G(d,p) 0.022 0.714 -0.442 -0.451 -0.761 -0.620 -0.663 -0.641 -0.677 -0.602 -0.440
6-311+G(d) 0.771 0.019 0.713 -0.439 -0.446 -0.760 -0.621 -0.660 -0.646 -0.672 -0.607 -0.433
6-311++G(d,p) 0.019 0.736 -0.417 -0.423 -0.738 -0.598 -0.637 -0.622 -0.650 -0.583 -0.409
Def2SVP 0.747 0.027 0.688 -0.466 -0.476 -0.782 -0.642 -0.687 -0.663 -0.696 -0.616 -0.430
Def2SVPD 0.023 0.712 -0.438 -0.446 -0.754 -0.615 -0.657 -0.633 -0.664 -0.587 -0.405
Def2TZVP 0.904 0.012 0.848 -0.301 -0.309 -0.623 -0.485 -0.523 -0.508 -0.535 -0.477 -0.298
Def2TZVPD 0.012 0.852 -0.298 -0.306 -0.620 -0.481 -0.519 -0.504 -0.531 -0.474 -0.295
cc-pVDZ 0.750 0.026 0.715 -0.451 -0.464 -0.768 -0.627 -0.675 -0.666 -0.694 -0.614 -0.448
aug-cc-pVDZ 0.022 0.739 -0.427 -0.437 -0.743 -0.603 -0.647 -0.639 -0.665 -0.588 -0.425
cc-pVTZ 0.916 0.010 0.856 -0.289 -0.297 -0.612 -0.473 -0.511 -0.500 -0.521 -0.471 -0.286
aug-cc-pVTZ
CASSCF MP2 LSDA PBE PBE0 B3LYP CAM-B3LYP wB97XD M06-2X SOGGA11X M11 MN15
Electronic Structure Method
0.8 0.6 0.4 0.2 0.0 −0.2 −0.4 −0.6
Error [Relative to CCSD] [a.u.]

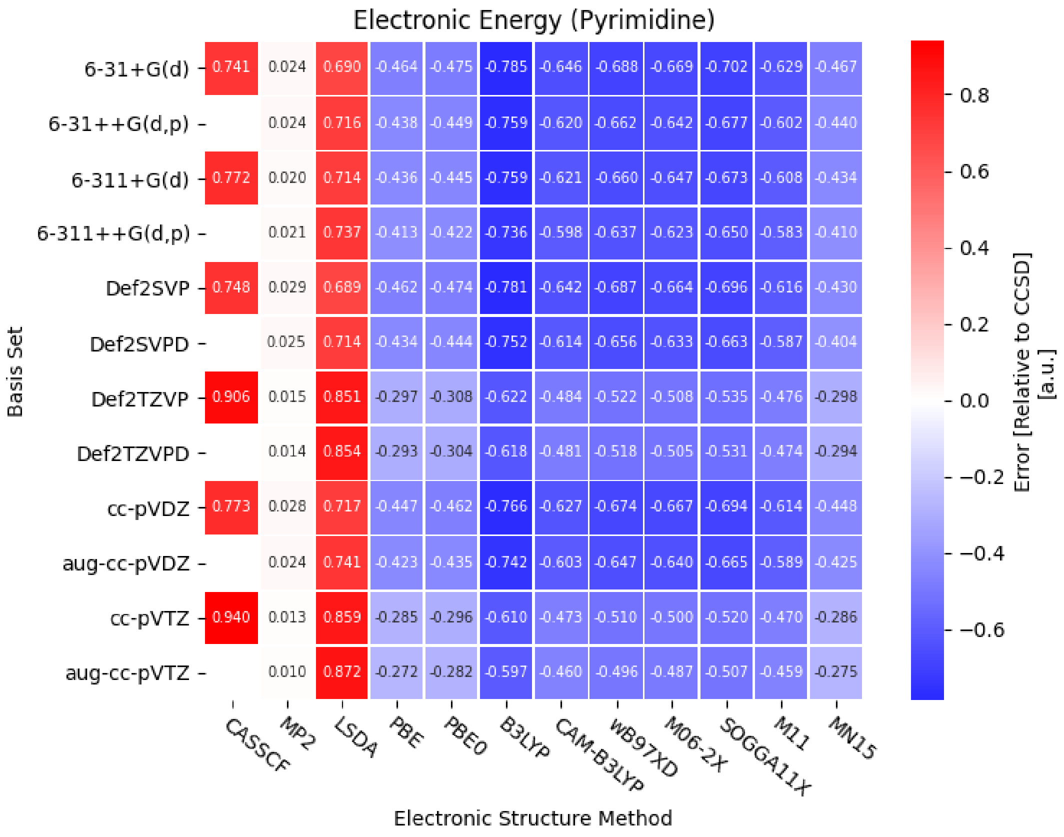

Electronic Energy (Pyrimidine)
Basis Set
6-31+G(d)
6-31++G(d,p)
6-311+G(d)
6-311++G(d,p)
Def2SVP
Def2SVPD
Def2TZVP
Def2TZVPD
cc-pVDZ
aug-cc-pVDZ
cc-pVTZ
aug-cc-pVTZ
CASSCF
MP2
LSDA
PBE
PBE0
B3LYP
CAM-B3LYP
wB97XD
M06-2X
SOGGA11X
M11
MN15
Electronic Structure Method
Error [Relative to CCSD] [a.u.]
0.8
0.6
0.4
0.2
0.0
−0.2
−0.4
−0.6

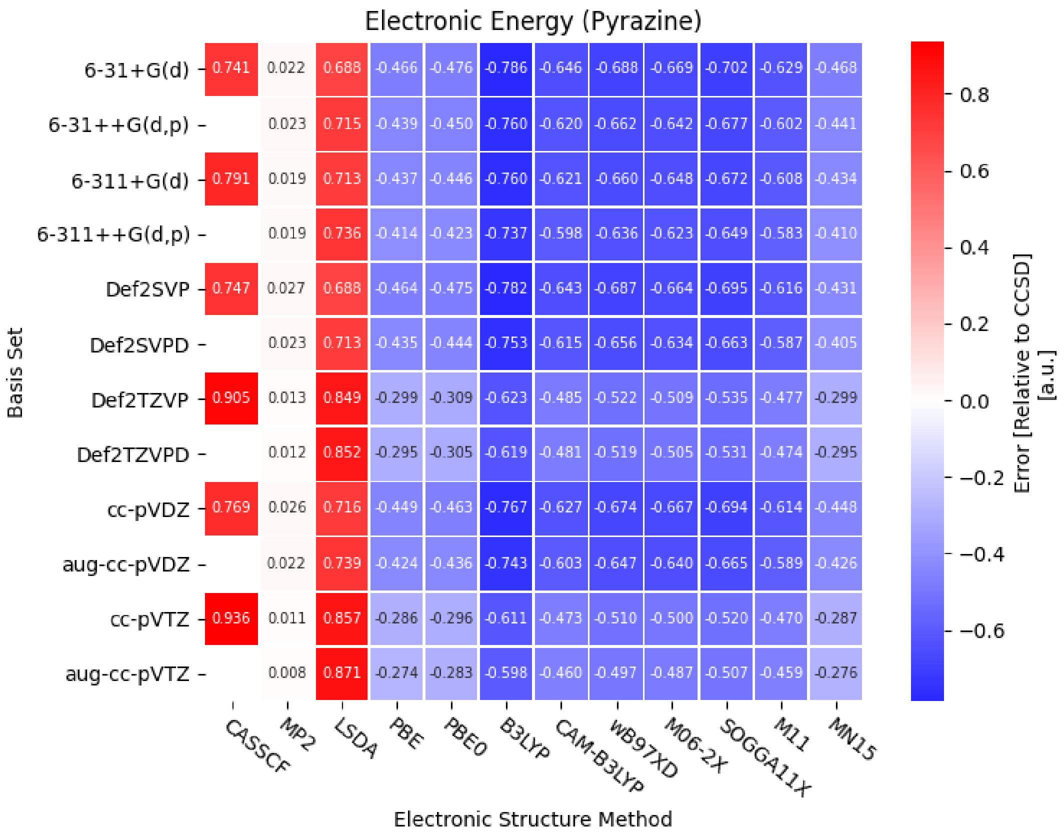

Electronic Energy (Pyrazine)
Basis Set
6-31+G(d)
6-31++G(d,p)
6-311+G(d)
6-311++G(d,p)
Def2SVP
Def2SVPD
Def2TZVP
Def2TZVPD
cc-pVDZ
aug-cc-pVDZ
cc-pVTZ
aug-cc-pVTZ
CASSCF
MP2
LSDA
PBE
PBE0
B3LYP
CAM-B3LYP
wB97XD
M06-2X
SOGGA11X
M11
MN15
Electronic Structure Method
Error [Relative to CCSD] [a.u.]
0.8
0.6
0.4
0.2
0.0
−0.2
−0.4
−0.6

D.7 Zero Point Energy

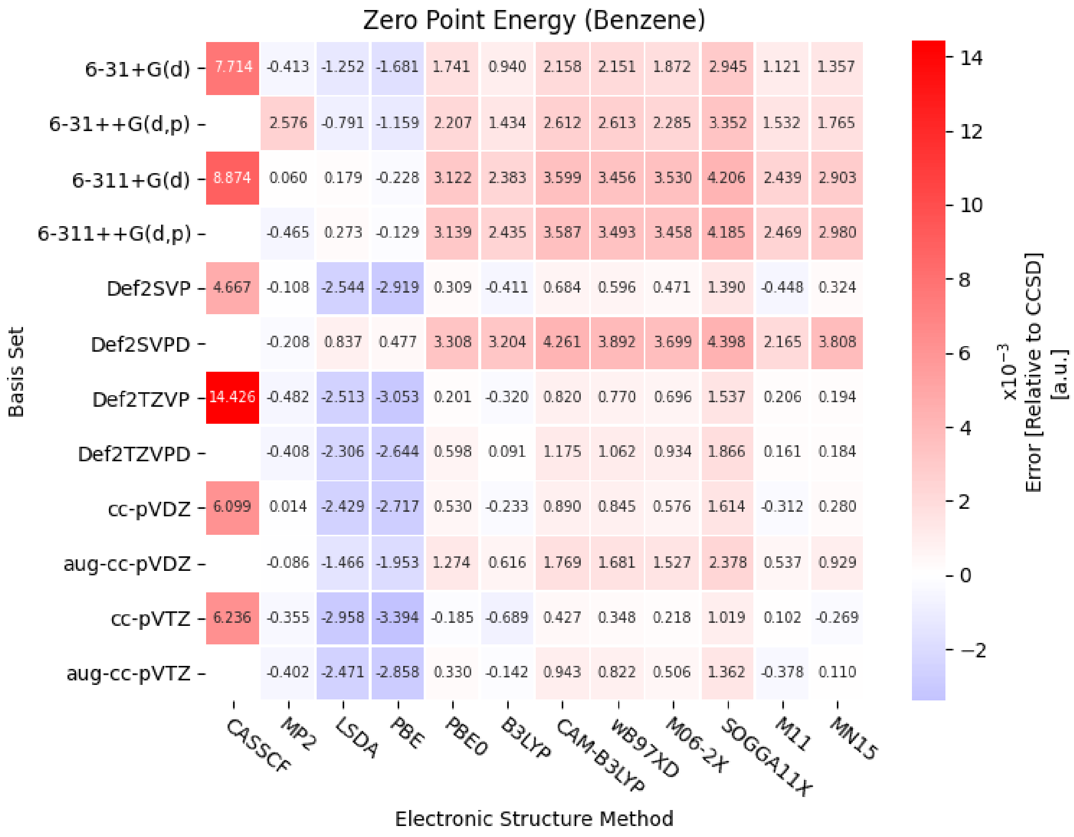

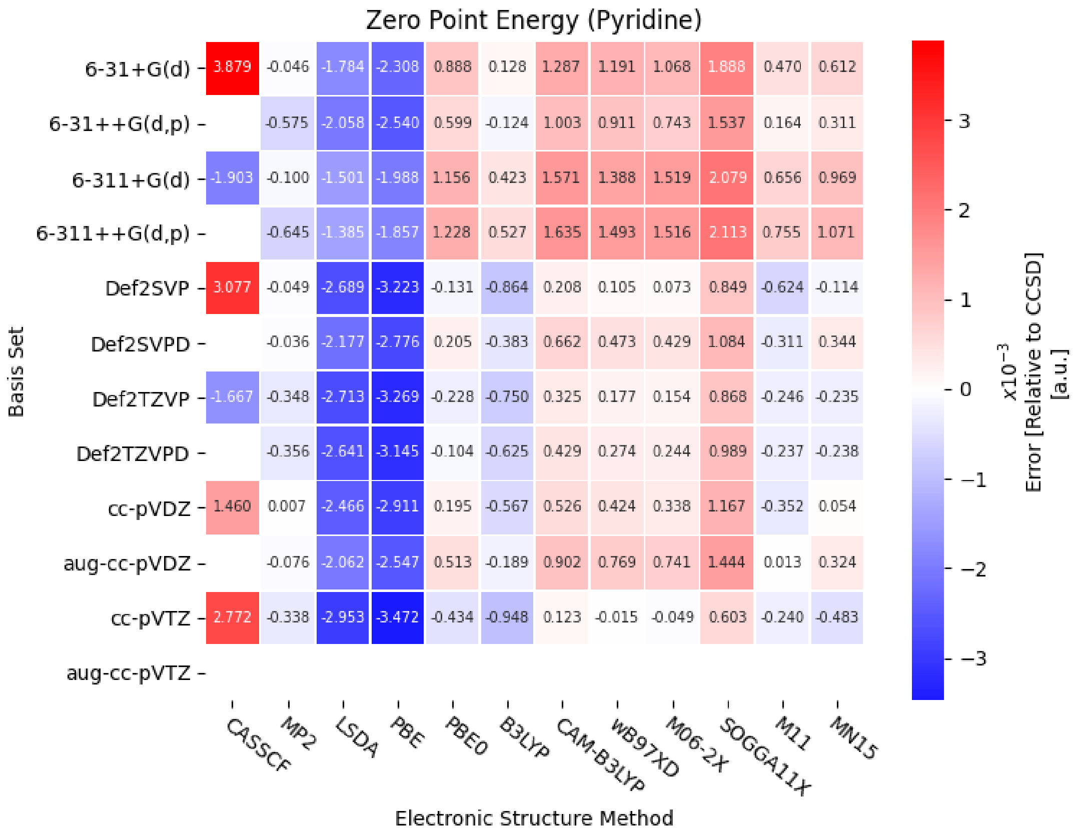
Zero Point Energy (Pyridine)
6-31+G(d) 3.879 -0.046 -1.784 -2.308 0.888 0.128 1.287 1.191 1.068 1.888 0.470 0.612
6-31++G(d,p) -0.575 -2.058 -2.540 0.599 -0.124 1.003 0.911 0.743 1.537 0.164 0.311
6-311+G(d) -1.903 -0.100 -1.501 -1.988 1.156 0.423 1.571 1.388 1.519 2.079 0.656 0.969
6-311++G(d,p) -0.645 -1.385 -1.857 1.228 0.527 1.635 1.493 1.516 2.113 0.755 1.071
Def2SVP 3.077 -0.049 -2.689 -3.223 -0.131 -0.864 0.208 0.105 0.073 0.849 -0.624 -0.114
Def2SVPD -0.036 -2.177 -2.776 0.205 -0.383 0.662 0.473 0.429 1.084 -0.311 0.344
Def2TZVP -1.667 -0.348 -2.713 -3.269 -0.228 -0.750 0.325 0.177 0.154 0.868 -0.246 -0.235
Def2TZVPD -0.356 -2.641 -3.145 -0.104 -0.625 0.429 0.274 0.244 0.989 -0.237 -0.238
cc-pVDZ 1.460 0.007 -2.466 -2.911 0.195 -0.567 0.526 0.424 0.338 1.167 -0.352 0.054
aug-cc-pVDZ -0.076 -2.062 -2.547 0.513 -0.189 0.902 0.769 0.741 1.444 0.013 0.324
cc-pVTZ 2.772 -0.338 -2.953 -3.472 -0.434 -0.948 0.123 -0.015 -0.049 0.603 -0.240 -0.483
aug-cc-pVTZ
Basis Set
CASSCF MP2 LSDA PBE PBE0 B3LYP CAM-B3LYP wB97XD M06-2X SOGGA11X M11 MN15
Electronic Structure Method
3 2 1 0 −1 −2 −3
x10^{-3}
Error [Relative to CCSD]
[a.u.]

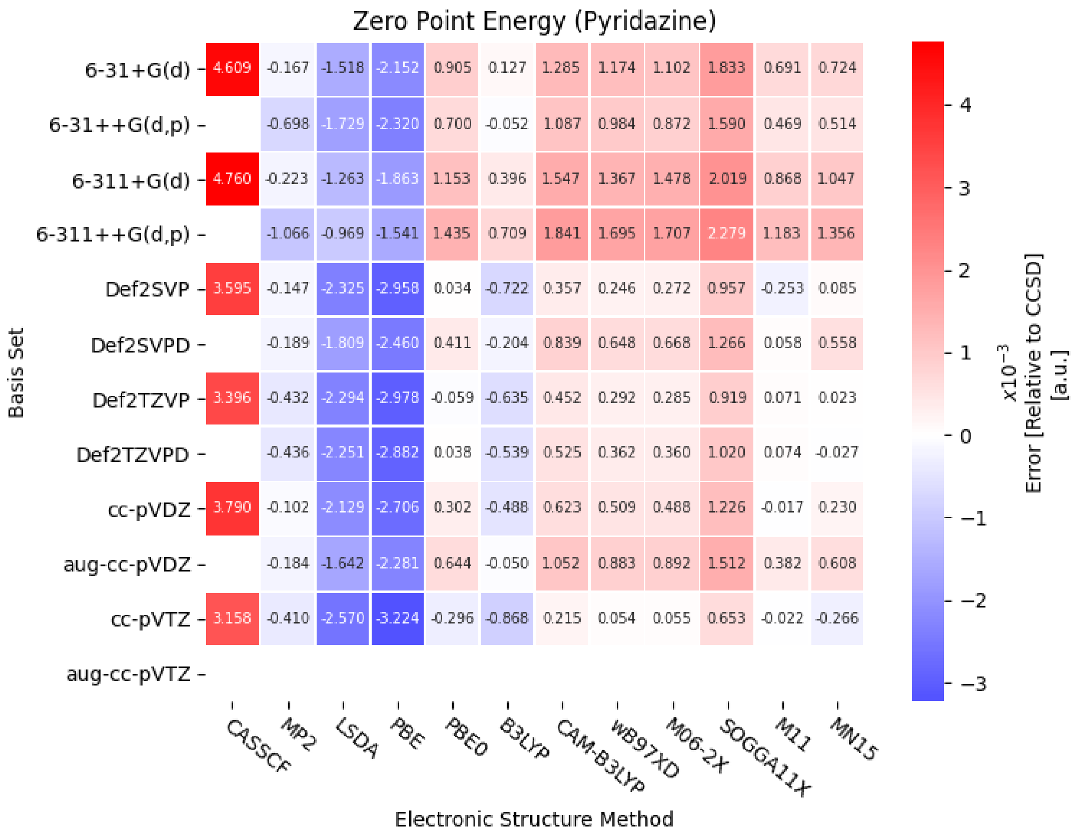
Zero Point Energy (Pyridazine)
6-31+G(d) 4.609 -0.167 -1.518 -2.152 0.905 0.127 1.285 1.174 1.102 1.833 0.691 0.724
6-31++G(d,p) -0.698 -1.729 -2.320 0.700 -0.052 1.087 0.984 0.872 1.590 0.469 0.514
6-311+G(d) 4.760 -0.223 -1.263 -1.863 1.153 0.396 1.547 1.367 1.478 2.019 0.868 1.047
6-311++G(d,p) -1.066 -0.969 -1.541 1.435 0.709 1.841 1.695 1.707 2.279 1.183 1.356
Def2SVP 3.595 -0.147 -2.325 -2.958 0.034 -0.722 0.357 0.246 0.272 0.957 -0.253 0.085
Def2SVPD -0.189 -1.809 -2.460 0.411 -0.204 0.839 0.648 0.668 1.266 0.058 0.558
Def2TZVP 3.396 -0.432 -2.294 -2.978 -0.059 -0.635 0.452 0.292 0.285 0.919 0.071 0.023
Def2TZVPD -0.436 -2.251 -2.882 0.038 -0.539 0.525 0.362 0.360 1.020 0.074 -0.027
cc-pVDZ 3.790 -0.102 -2.129 -2.706 0.302 -0.488 0.623 0.509 0.488 1.226 -0.017 0.230
aug-cc-pVDZ -0.184 -1.642 -2.281 0.644 -0.050 1.052 0.883 0.892 1.512 0.382 0.608
cc-pVTZ 3.158 -0.410 -2.570 -3.224 -0.296 -0.868 0.215 0.054 0.055 0.653 -0.022 -0.266
aug-cc-pVTZ
Basis Set
CASSCF MP2 LSDA PBE PBE0 B3LYP CAM-B3LYP wB97XD M06-2X SOGGA11X M11 MN15
Electronic Structure Method
4 3 2 1 0 −1 −2 −3
x10^{-3}
Error [Relative to CCSD]
[a.u.]

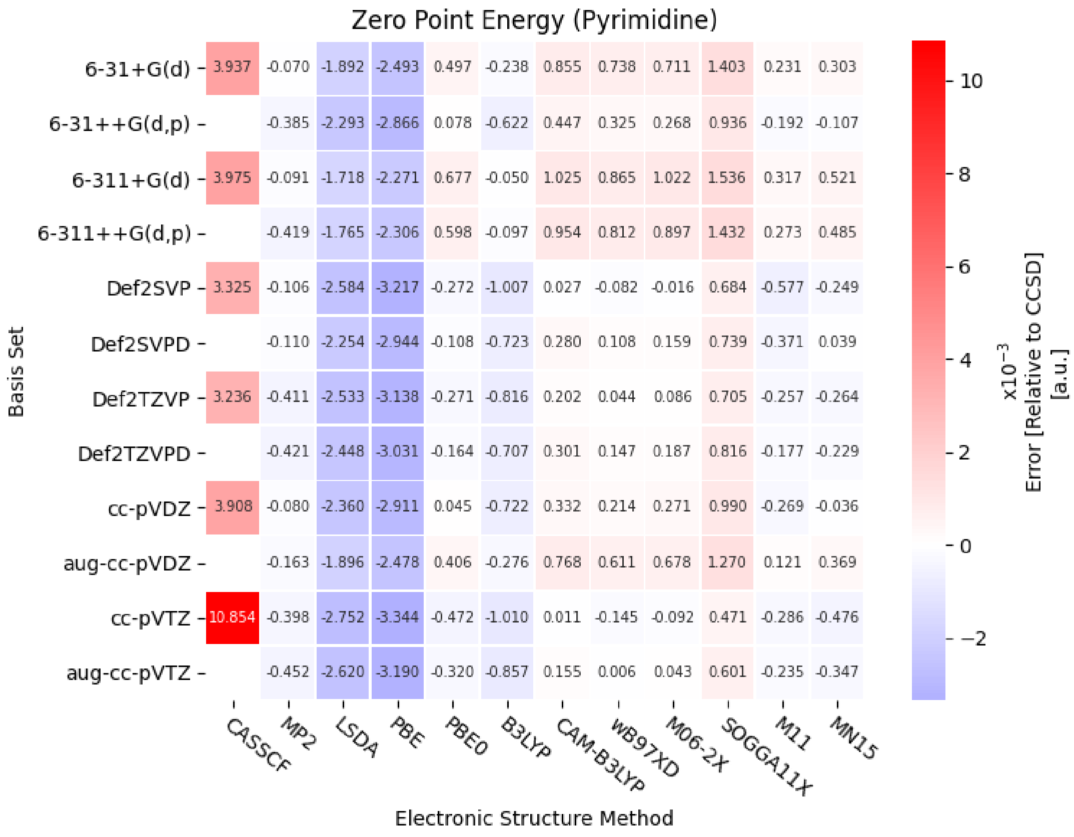
Zero Point Energy (Pyrimidine)
6-31+G(d) 3.937 -0.070 -1.892 -2.493 0.497 -0.238 0.855 0.738 0.711 1.403 0.231 0.303
6-31++G(d,p) -0.385 -2.293 -2.866 0.078 -0.622 0.447 0.325 0.268 0.936 -0.192 -0.107
6-311+G(d) 3.975 -0.091 -1.718 -2.271 0.677 -0.050 1.025 0.865 1.022 1.536 0.317 0.521
6-311++G(d,p) -0.419 -1.765 -2.306 0.598 -0.097 0.954 0.812 0.897 1.432 0.273 0.485
Def2SVP 3.325 -0.106 -2.584 -3.217 -0.272 -1.007 0.027 -0.082 -0.016 0.684 -0.577 -0.249
Def2SVPD -0.110 -2.254 -2.944 -0.108 -0.723 0.280 0.108 0.159 0.739 -0.371 0.039
Def2TZVP 3.236 -0.411 -2.533 -3.138 -0.271 -0.816 0.202 0.044 0.086 0.705 -0.257 -0.264
Def2TZVPD -0.421 -2.448 -3.031 -0.164 -0.707 0.301 0.147 0.187 0.816 -0.177 -0.229
cc-pVDZ 3.908 -0.080 -2.360 -2.911 0.045 -0.722 0.332 0.214 0.271 0.990 -0.269 -0.036
aug-cc-pVDZ -0.163 -1.896 -2.478 0.406 -0.276 0.768 0.611 0.678 1.270 0.121 0.369
cc-pVTZ 10.854 -0.398 -2.752 -3.344 -0.472 -1.010 0.011 -0.145 -0.092 0.471 -0.286 -0.476
aug-cc-pVTZ -0.452 -2.620 -3.190 -0.320 -0.857 0.155 0.006 0.043 0.601 -0.235 -0.347
Basis Set
CASSCF MP2 LSDA PBE PBE0 B3LYP CAM-B3LYP wB97XD M06-2X SOGGA11X M11 MN15
Electronic Structure Method
10
8
6
4
2
0
−2
x10^{-3}
Error [Relative to CCSD]
[a.u.]

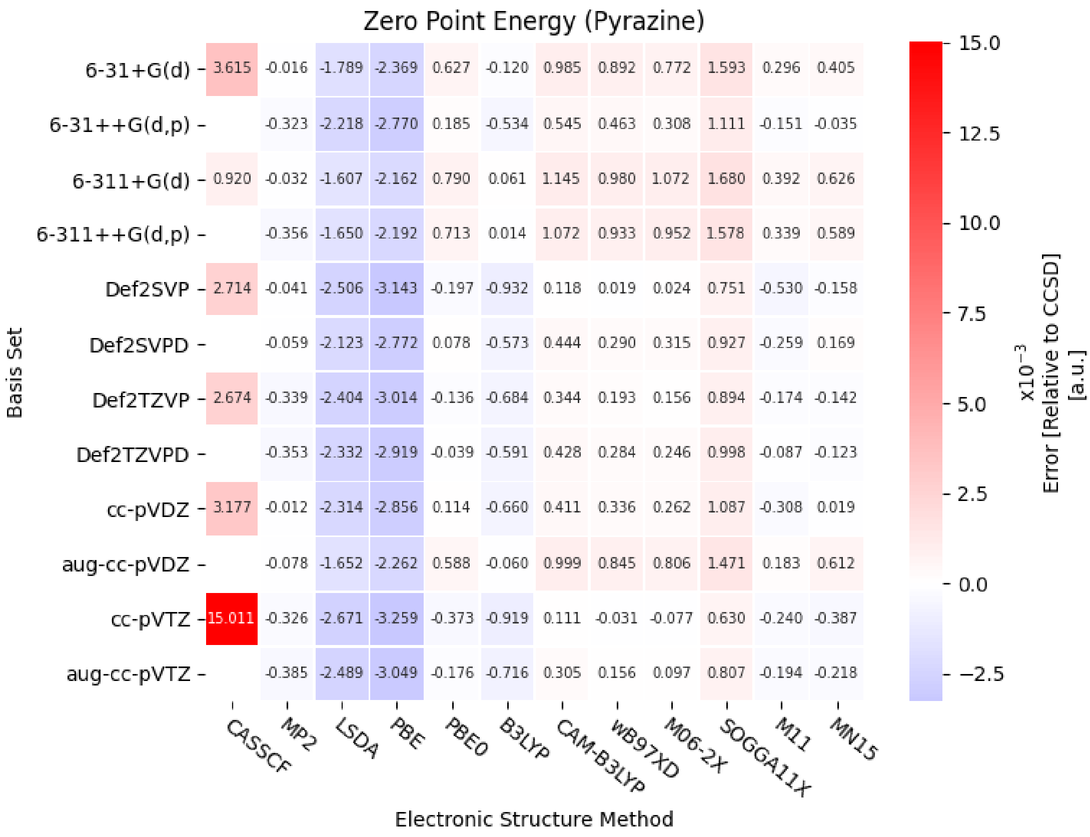
Zero Point Energy (Pyrazine)
6-31+G(d) 3.615 -0.016 -1.789 -2.369 0.627 -0.120 0.985 0.892 0.772 1.593 0.296 0.405
6-31++G(d,p) -0.323 -2.218 -2.770 0.185 -0.534 0.545 0.463 0.308 1.111 -0.151 -0.035
6-311+G(d) 0.920 -0.032 -1.607 -2.162 0.790 0.061 1.145 0.980 1.072 1.680 0.392 0.626
6-311++G(d,p) -0.356 -1.650 -2.192 0.713 0.014 1.072 0.933 0.952 1.578 0.339 0.589
Def2SVP 2.714 -0.041 -2.506 -3.143 -0.197 -0.932 0.118 0.019 0.024 0.751 -0.530 -0.158
Def2SVPD -0.059 -2.123 -2.772 0.078 -0.573 0.444 0.290 0.315 0.927 -0.259 0.169
Def2TZVP 2.674 -0.339 -2.404 -3.014 -0.136 -0.684 0.344 0.193 0.156 0.894 -0.174 -0.142
Def2TZVPD -0.353 -2.332 -2.919 -0.039 -0.591 0.428 0.284 0.246 0.998 -0.087 -0.123
cc-pVDZ 3.177 -0.012 -2.314 -2.856 0.114 -0.660 0.411 0.336 0.262 1.087 -0.308 0.019
aug-cc-pVDZ -0.078 -1.652 -2.262 0.588 -0.060 0.999 0.845 0.806 1.471 0.183 0.612
cc-pVTZ 15.011 -0.326 -2.671 -3.259 -0.373 -0.919 0.111 -0.031 -0.077 0.630 -0.240 -0.387
aug-cc-pVTZ -0.385 -2.489 -3.049 -0.176 -0.716 0.305 0.156 0.097 0.807 -0.194 -0.218
Basis Set
CASSCF MP2 LSDA PBE PBE0 B3LYP CAM-B3LYP wB97XD M06-2X SOGGA11X M11 MN15
Electronic Structure Method
15.0
12.5
10.0
7.5
5.0
2.5
0.0
−2.5
x10^{-3}
Error [Relative to CCSD]
[a.u.]

D.8 Sum of Electronic Energy and ZPE

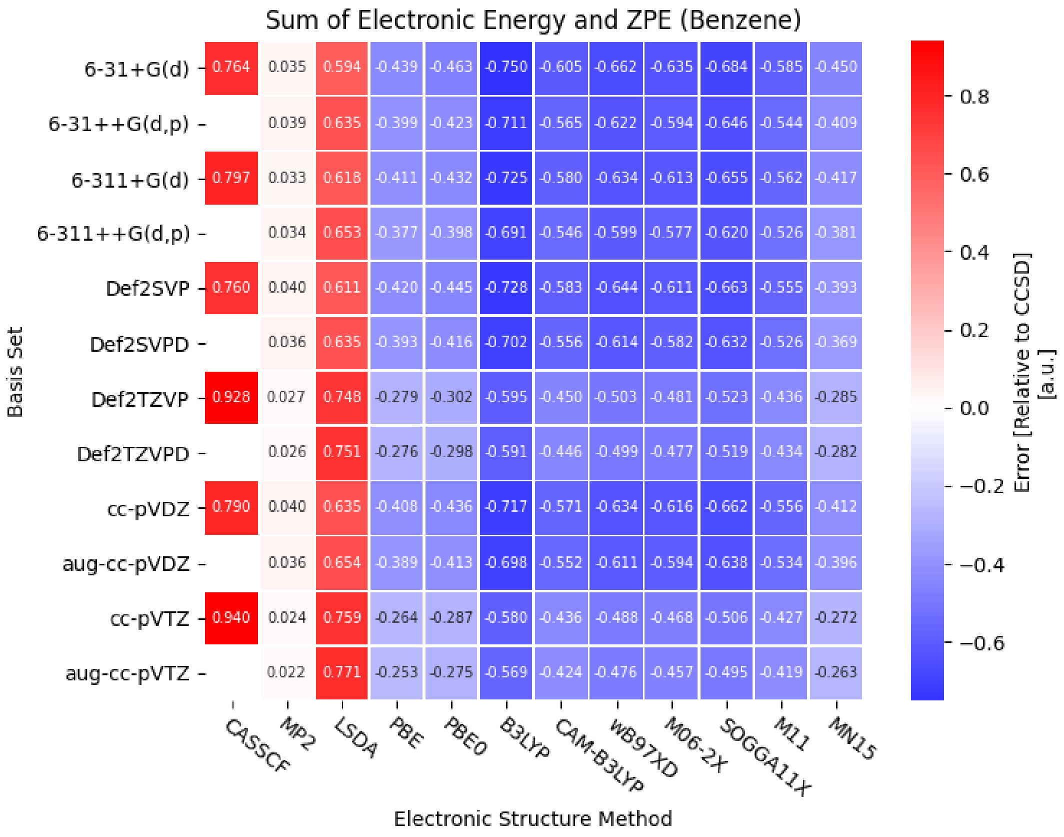

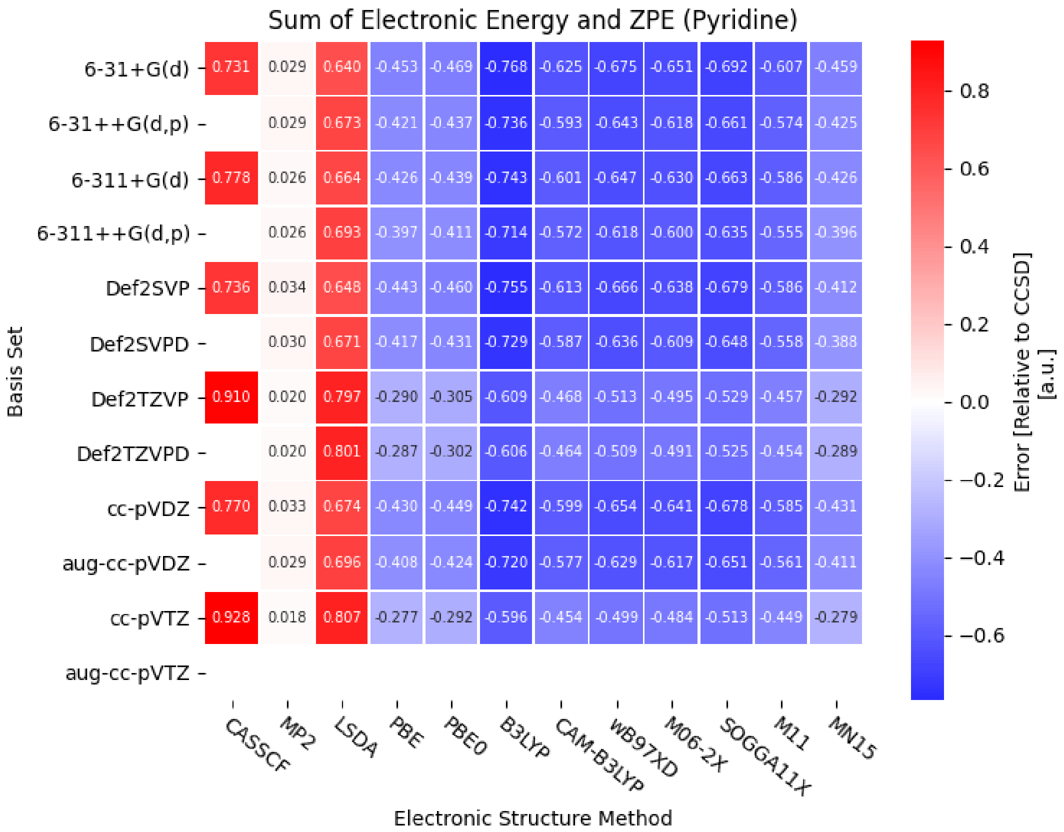
Sum of Electronic Energy and ZPE (Pyridine)
Basis Set
6-31+G(d)
6-31++G(d,p)
6-311+G(d)
6-311++G(d,p)
Def2SVP
Def2SVPD
Def2TZVP
Def2TZVPD
cc-pVDZ
aug-cc-pVDZ
cc-pVTZ
aug-cc-pVTZ
0.731 0.029 0.640 -0.453 -0.469 -0.768 -0.625 -0.675 -0.651 -0.692 -0.607 -0.459
0.029 0.673 -0.421 -0.437 -0.736 -0.593 -0.643 -0.618 -0.661 -0.574 -0.425
0.778 0.026 0.664 -0.426 -0.439 -0.743 -0.601 -0.647 -0.630 -0.663 -0.586 -0.426
0.026 0.693 -0.397 -0.411 -0.714 -0.572 -0.618 -0.600 -0.635 -0.555 -0.396
0.736 0.034 0.648 -0.443 -0.460 -0.755 -0.613 -0.666 -0.638 -0.679 -0.586 -0.412
0.030 0.671 -0.417 -0.431 -0.729 -0.587 -0.636 -0.609 -0.648 -0.558 -0.388
0.910 0.020 0.797 -0.290 -0.305 -0.609 -0.468 -0.513 -0.495 -0.529 -0.457 -0.292
0.020 0.801 -0.287 -0.302 -0.606 -0.464 -0.509 -0.491 -0.525 -0.454 -0.289
0.770 0.033 0.674 -0.430 -0.449 -0.742 -0.599 -0.654 -0.641 -0.678 -0.585 -0.431
0.029 0.696 -0.408 -0.424 -0.720 -0.577 -0.629 -0.617 -0.651 -0.561 -0.411
0.928 0.018 0.807 -0.277 -0.292 -0.596 -0.454 -0.499 -0.484 -0.513 -0.449 -0.279
CASSCF
MP2
LSDA
PBE
PBE0
B3LYP
CAM-B3LYP
wB97XD
M06-2X
SOGGA11X
M11
MN15
Electronic Structure Method
0.8
0.6
0.4
0.2
0.0
−0.2
−0.4
−0.6
Error [Relative to CCSD] [a.u.]

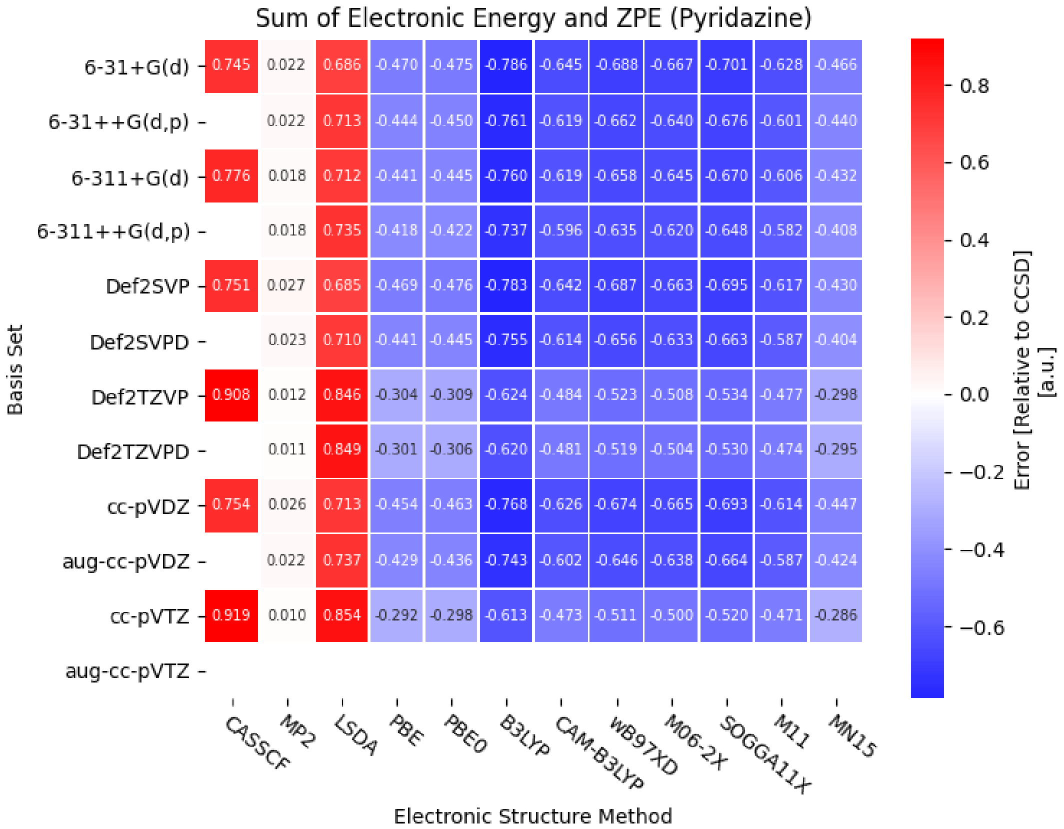
Sum of Electronic Energy and ZPE (Pyridazine)
Basis Set
6-31+G(d)
6-31++G(d,p)
6-311+G(d)
6-311++G(d,p)
Def2SVP
Def2SVPD
Def2TZVP
Def2TZVPD
cc-pVDZ
aug-cc-pVDZ
cc-pVTZ
aug-cc-pVTZ
0.745 0.022 0.686 -0.470 -0.475 -0.786 -0.645 -0.688 -0.667 -0.701 -0.628 -0.466
0.022 0.713 -0.444 -0.450 -0.761 -0.619 -0.662 -0.640 -0.676 -0.601 -0.440
0.776 0.018 0.712 -0.441 -0.445 -0.760 -0.619 -0.658 -0.645 -0.670 -0.606 -0.432
0.018 0.735 -0.418 -0.422 -0.737 -0.596 -0.635 -0.620 -0.648 -0.582 -0.408
0.751 0.027 0.685 -0.469 -0.476 -0.783 -0.642 -0.687 -0.663 -0.695 -0.617 -0.430
0.023 0.710 -0.441 -0.445 -0.755 -0.614 -0.656 -0.633 -0.663 -0.587 -0.404
0.908 0.012 0.846 -0.304 -0.309 -0.624 -0.484 -0.523 -0.508 -0.534 -0.477 -0.298
0.011 0.849 -0.301 -0.306 -0.620 -0.481 -0.519 -0.504 -0.530 -0.474 -0.295
0.754 0.026 0.713 -0.454 -0.463 -0.768 -0.626 -0.674 -0.665 -0.693 -0.614 -0.447
0.022 0.737 -0.429 -0.436 -0.743 -0.602 -0.646 -0.638 -0.664 -0.587 -0.424
0.919 0.010 0.854 -0.292 -0.298 -0.613 -0.473 -0.511 -0.500 -0.520 -0.471 -0.286
CASSCF
MP2
LSDA
PBE
PBE0
B3LYP
CAM-B3LYP
wB97XD
M06-2X
SOGGA11X
M11
MN15
Electronic Structure Method
0.8
0.6
0.4
0.2
0.0
−0.2
−0.4
−0.6
Error [Relative to CCSD] [a.u.]

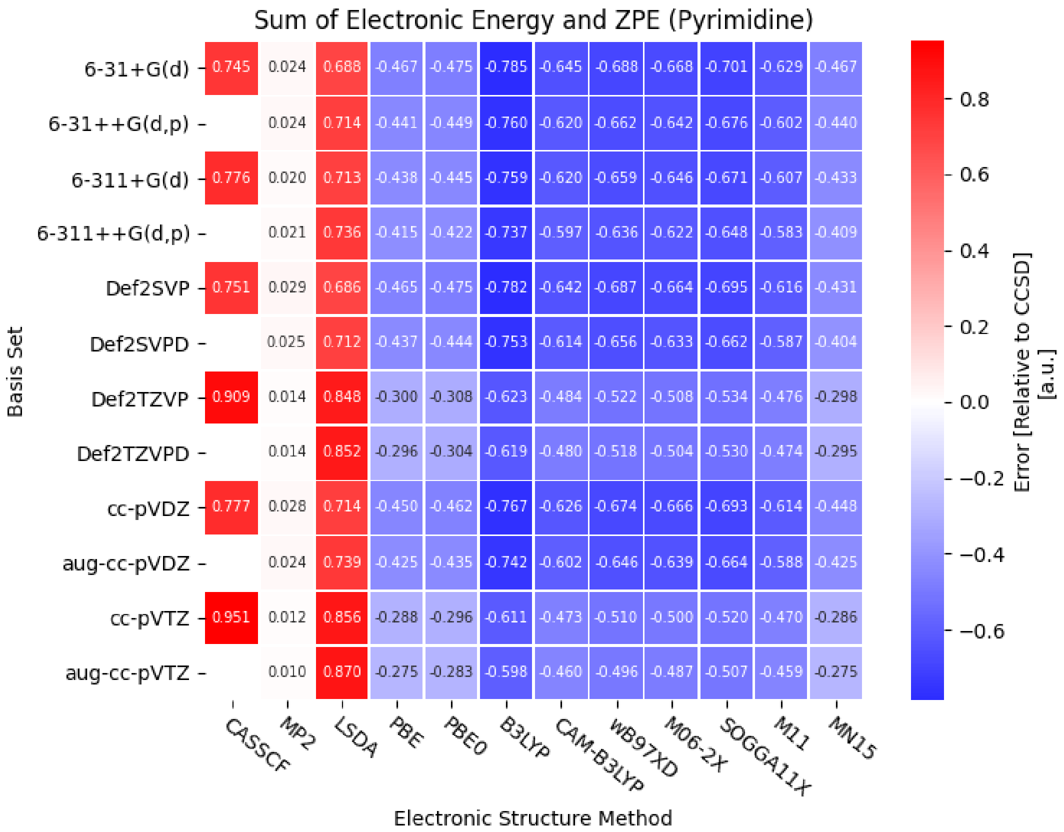
Sum of Electronic Energy and ZPE (Pyrimidine)
Basis Set
Electronic Structure Method
Error [Relative to CCSD] [a.u.]
CASSCF MP2 LSDA PBE PBE0 B3LYP CAM-B3LYP wB97XD M06-2X SOGGA11X M11 MN15
6-31+G(d) 0.745 0.024 0.688 -0.467 -0.475 -0.785 -0.645 -0.688 -0.668 -0.701 -0.629 -0.467
6-31++G(d,p) 0.024 0.714 -0.441 -0.449 -0.760 -0.620 -0.662 -0.642 -0.676 -0.602 -0.440
6-311+G(d) 0.776 0.020 0.713 -0.438 -0.445 -0.759 -0.620 -0.659 -0.646 -0.671 -0.607 -0.433
6-311++G(d,p) 0.021 0.736 -0.415 -0.422 -0.737 -0.597 -0.636 -0.622 -0.648 -0.583 -0.409
Def2SVP 0.751 0.029 0.686 -0.465 -0.475 -0.782 -0.642 -0.687 -0.664 -0.695 -0.616 -0.431
Def2SVPD 0.025 0.712 -0.437 -0.444 -0.753 -0.614 -0.656 -0.633 -0.662 -0.587 -0.404
Def2TZVP 0.909 0.014 0.848 -0.300 -0.308 -0.623 -0.484 -0.522 -0.508 -0.534 -0.476 -0.298
Def2TZVPD 0.014 0.852 -0.296 -0.304 -0.619 -0.480 -0.518 -0.504 -0.530 -0.474 -0.295
cc-pVDZ 0.777 0.028 0.714 -0.450 -0.462 -0.767 -0.626 -0.674 -0.666 -0.693 -0.614 -0.448
aug-cc-pVDZ 0.024 0.739 -0.425 -0.435 -0.742 -0.602 -0.646 -0.639 -0.664 -0.588 -0.425
cc-pVTZ 0.951 0.012 0.856 -0.288 -0.296 -0.611 -0.473 -0.510 -0.500 -0.520 -0.470 -0.286
aug-cc-pVTZ 0.010 0.870 -0.275 -0.283 -0.598 -0.460 -0.496 -0.487 -0.507 -0.459 -0.275
0.8 0.6 0.4 0.2 0.0 −0.2 −0.4 −0.6

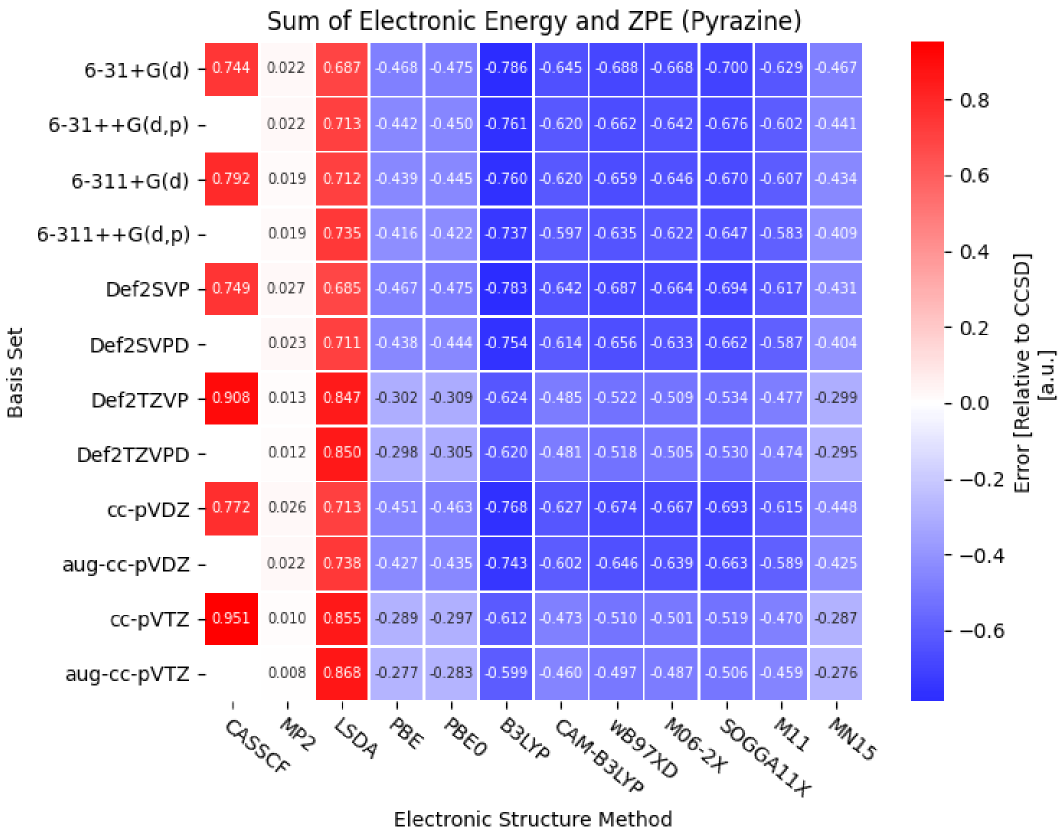
Sum of Electronic Energy and ZPE (Pyrazine)
Basis Set
Electronic Structure Method
Error [Relative to CCSD] [a.u.]
CASSCF MP2 LSDA PBE PBE0 B3LYP CAM-B3LYP wB97XD M06-2X SOGGA11X M11 MN15
6-31+G(d) 0.744 0.022 0.687 -0.468 -0.475 -0.786 -0.645 -0.688 -0.668 -0.700 -0.629 -0.467
6-31++G(d,p) 0.022 0.713 -0.442 -0.450 -0.761 -0.620 -0.662 -0.642 -0.676 -0.602 -0.441
6-311+G(d) 0.792 0.019 0.712 -0.439 -0.445 -0.760 -0.620 -0.659 -0.646 -0.670 -0.607 -0.434
6-311++G(d,p) 0.019 0.735 -0.416 -0.422 -0.737 -0.597 -0.635 -0.622 -0.647 -0.583 -0.409
Def2SVP 0.749 0.027 0.685 -0.467 -0.475 -0.783 -0.642 -0.687 -0.664 -0.694 -0.617 -0.431
Def2SVPD 0.023 0.711 -0.438 -0.444 -0.754 -0.614 -0.656 -0.633 -0.662 -0.587 -0.404
Def2TZVP 0.908 0.013 0.847 -0.302 -0.309 -0.624 -0.485 -0.522 -0.509 -0.534 -0.477 -0.299
Def2TZVPD 0.012 0.850 -0.298 -0.305 -0.620 -0.481 -0.518 -0.505 -0.530 -0.474 -0.295
cc-pVDZ 0.772 0.026 0.713 -0.451 -0.463 -0.768 -0.627 -0.674 -0.667 -0.693 -0.615 -0.448
aug-cc-pVDZ 0.022 0.738 -0.427 -0.435 -0.743 -0.602 -0.646 -0.639 -0.663 -0.589 -0.425
cc-pVTZ 0.951 0.010 0.855 -0.289 -0.297 -0.612 -0.473 -0.510 -0.501 -0.519 -0.470 -0.287
aug-cc-pVTZ 0.008 0.868 -0.277 -0.283 -0.599 -0.460 -0.497 -0.487 -0.506 -0.459 -0.276
0.8 0.6 0.4 0.2 0.0 −0.2 −0.4 −0.6

E Internal Coordinate Representations

The internal coordinate representation of the five molecules are listed in the following:

Benzene

X
X 1 1.0
C 1 cx 2 90.0
C 1 cx 2 90.0 3 60.0
C 1 cx 2 90.0 4 60.0
C 1 cx 2 90.0 5 60.0
C 1 cx 2 90.0 6 60.0
C 1 cx 2 90.0 7 60.0
H 1 hx 2 90.0 3 0.0
H 1 hx 2 90.0 4 0.0
H 1 hx 2 90.0 5 0.0
H 1 hx 2 90.0 6 0.0
H 1 hx 2 90.0 7 0.0
H 1 hx 2 90.0 8 0.0

cx = 1.30
hx = 2.30

Pyridazine

X
X 1 1.0
N 1 cx 2 90.0
N 1 cx 2 90.0 3 60.0
C 1 cx 2 90.0 4 60.0
C 1 cx 2 90.0 5 60.0
C 1 cx 2 90.0 6 60.0
C 1 cx 2 90.0 7 60.0
H 1 hx 2 90.0 5 0.0
H 1 hx 2 90.0 6 0.0
H 1 hx 2 90.0 7 0.0
H 1 hx 2 90.0 8 0.0

cx = 1.30
hx = 2.30

Pyridine

c
c 1 cc2
c 2 cc3 1 ccc3
n 3 nc4 2 ncc4 1 dih4
c 4 cn5 3 cnc5 2 dih5
c 5 cc6 4 ccn6 3 dih6
h 2 hc7 1 hcc7 3 dih7
h 3 hc8 2 hcc8 4 dih8
h 5 hc9 4 hcn9 3 dih9
h 6 hc10 5 hcc10 4 dih10
h 1 hc11 6 hcc11 2 dih11

cc2 = 1.400000
cc3 = 1.400000
ccc3 = 120.000
nc4 =1.400000
ncc4 = 120.000
dih4 = 0.000
cn5 = 1.400000
cnc5 = 120.000
dih5 = 0.000
cc6 = 1.400000
ccn6 = 120.000
dih6 = 0.000
hc7 = 1.089000
hcc7 = 120.000
dih7 = 180.000
hc8 = 1.089000
hcc8 = 120.000
dih8 = 180.000
hc9 = 1.089000
hcn9 = 120.000
dih9 = 180.000
hc10 = 1.089000
hcc10 = 120.000
dih10 = 180.000
hc11 = 1.070000
hcc11 = 120.000
dih11 = 180.000

Pyrimidine

n
c 1 cn2
c 2 cc3 1 ccn3
c 3 cc4 2 ccc4 1 dih4
n 4 nc5 3 ncc5 2 dih5
c 5 cn6 4 cnc6 3 dih6
h 2 hc7 1 hcn7 3 dih7
h 3 hc8 2 hcc8 4 dih8
h 4 hc9 3 hcc9 5 dih9
h 6 hc10 5 hcn10 4 dih10

cn2 = 1.400000
cc3 = 1.400000
ccn3 = 120.000
cc4 = 1.400000
ccc4 = 120.000
dih4 = 0.000
nc5 = 1.400000
ncc5 = 120.000
dih5 = 0.000
cn6 = 1.400000
cnc6 = 120.000
dih6 = 0.000
hc7 = 1.089000
hcn7 = 120.000
dih7 = 180.000
hc8 = 1.089000
hcc8 = 120.000
dih8 = 180.000
hc9 = 1.089000
hcc9 = 120.000
dih9 = 180.000
hc10 = 1.089000
hcn10 = 120.000
dih10 = 180.000

Pyrazine

n
c 1 cn2
c 2 cc3 1 ccn3
n 3 nc4 2 ncc4 1 dih4
c 4 cn5 3 cnc5 2 dih5
c 5 cc6 4 ccn6 3 dih6
h 2 hc7 1 hcn7 3 dih7
h 3 hc8 2 hcc8 4 dih8
h 5 hc9 4 hcn9 3 dih9
h 6 hc10 5 hcc10 4 dih10

cn2 = 1.400000
cc3 = 1.400000
ccn3 = 120.000
nc4 = 1.400000
ncc4 = 120.000
dih4 = 0.000
cn5 = 1.400000
cnc5 = 120.000
dih5 = 0.000
cc6 = 1.400000
ccn6 = 120.000
dih6 = 0.000
hc7 = 1.089000
hcn7 = 120.000
dih7 = 180.000
hc8 = 1.089000
hcc8 = 120.000
dih8 = 180.000
hc9 = 1.089000
hcn9 = 120.000
dih9 = 180.000
hc10 = 1.089000
hcc10 = 120.000
dih10 = 180.000

F Route Section and Calculation Setup

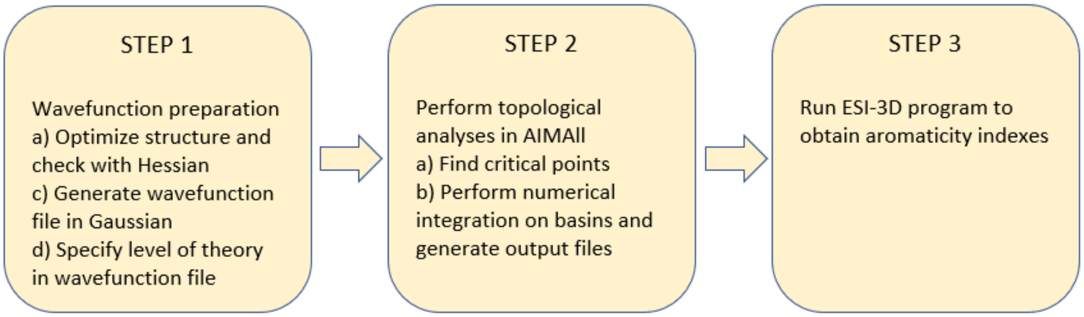

Scheme S1: Flowchart of computational approach.

Generic route section for the calculations in Gaussian 16:

```
%chk=checkpoint_file.chk
# opt freq electronic_structure_method/basis_set density=current
output=wfx

Title Card

0 1
Internal_coordinate_representation

wavefunction_file.wfx
```

Calculation setup for the wavefunction partition in the AIMAll software package:

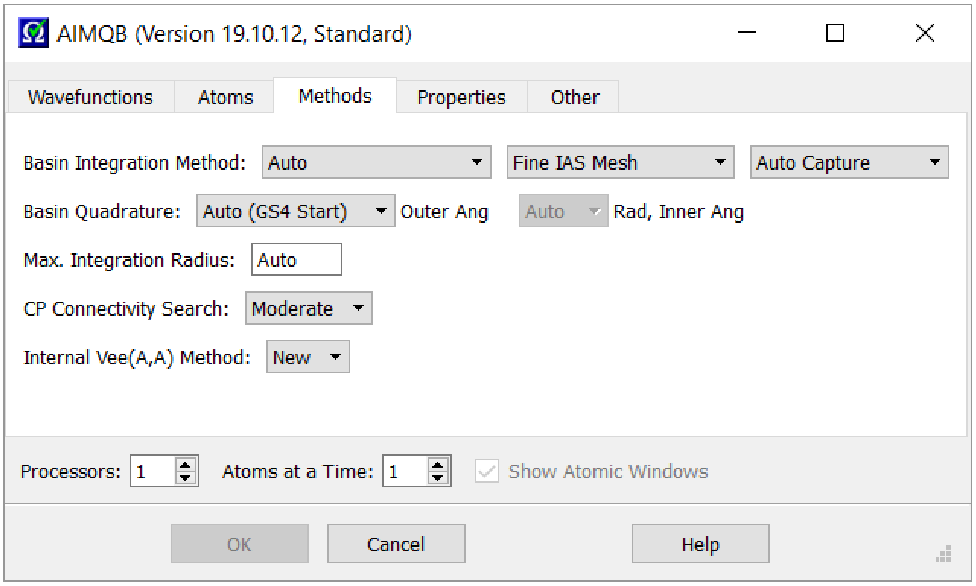

Generic input file (bad.in) for the ESI-3D calculations:

```
$TITLE
Title Card
$FULLOUT
$READWFN
wavefunction_file.wfx
$RING
    1 rings found
    6 members of 1th ring
    1   2   3   4   5   6
!$MULTPART
6
1 2 3 4 5 6
$AV124
$AV1245
```
